# Ionic Liquid Catalysts for Poly(ethylene terephthalate) Glycolysis: Use of Structure Activity Relationships to Combine Activity with Biodegradability

**DOI:** 10.1021/acssuschemeng.4c08491

**Published:** 2025-01-16

**Authors:** Lorenzo Pedrini, Chiara Zappelli, Stephen J. Connon

**Affiliations:** School of Chemistry, Trinity Biomedical Sciences Institute, Trinity College Dublin, 152-160 Pearse St., Dublin 2, Ireland

**Keywords:** ionic liquids, glycolysis, poly(ethylene terephthalate), green chemistry, recycling

## Abstract

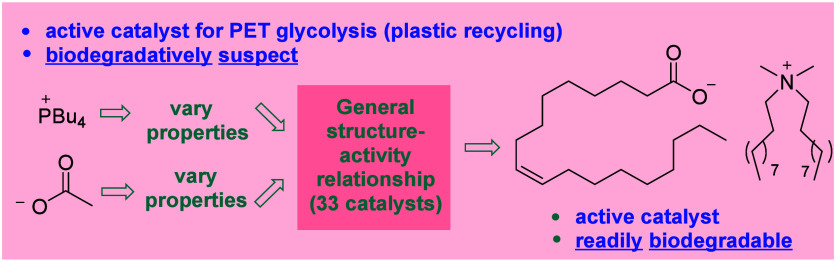

The chemical recycling of poly(ethylene terephthalate) (PET) plastic by catalytic glycolysis is an enabling technology for the circular economy that is attracting burgeoning academic and commercial interest. Ionic liquids are emerging as a versatile catalyst class for this transformation, yet general strategies for how both high activity and biodegradability can be incorporated into catalyst design have not yet emerged. Beginning with an active literature catalyst incorporating a phosphonium cation of concern from a biodegradability standpoint, a structure activity relationship study involving 33 systematically varied ionic liquid catalysts was undertaken, which highlighted (*inter alia*) the contribution of cation lipophilicity to activity and identified the hydrocinnamate and benzoate counteranions as highly serviceable. This allowed the design of a superior, high-activity catalyst, which remained of biodegradative concern. Subsequently, the structure–activity relationships and general principles uncovered in this study informed a biodegradability/activity-guided approach to catalyst design, leading to the development of three highly active catalysts that were either known to be readily biodegradable or comprised biodegradable anions and cations. All three significantly outperformed a benchmark cholinium ion-based glycolysis catalyst at low catalyst loadings of 1 mol %.

## Abstract 2

A structure−activity relationship study has allowed the design of highly active yet biodegradable ionic catalysts for PET plastic chemical recycling.

## Introduction

It has been estimated that without intervention, by 2050 there will be 12 trillion kg of plastic either in landfills or polluting the natural world.^1^ Poly(ethylene terephthalate) (PET, **1**) is a (petroleum-derived) condensation polymer found in (*inter alia*) beverage bottles, 'polyester' fibers, and food packaging. In 2021, 24.2 billion kg of PET was synthesized,^2^ which is more than the estimated mass of all wild land mammals.^3^ Production and supply chain greenhouse gas emissions (kg CO_2_e/kg polymer) associated with PET are the largest of the major plastic types;^4,5^ however, the material is eminently recyclable. The primary method of PET recycling is mechanical in nature; this is currently associated with superior environmental and economic metrics,^6^ however, it requires a clean and sorted PET stream, and without more resource-intensive treatments to remove contaminants and partially remediate thermal degradation/chain-scission during reprocessing,^7^ it produces recycled PET of an inferior quality to inexpensive virgin PET,^8^ leading to decreased circularity and downcycling.^5,9^ Chemical recycling of PET is an emerging complementary technology involving the solvolytic depolymerization of the plastic to monomers, which can then be purified and repolymerized to virgin PET. Unlike its mechanical variant, chemical recycling is potentially almost infinitely circular, amenable to mixed waste streams/colored PET waste and is an enabling technology for the upcycling of PET waste.^9−14^ Aminolytic, hydrolytic, methanolytic, and glycolytic processes have been developed,^9−13^ of which the latter has received the most commercial^14,15^ and academic^9−14^ attention due to the high boiling point of ethylene glycol (which allows high temperature depolymerization at atmospheric pressure in the presence of a catalyst) and the utility of the product (BHET, **2**), which can be either directly repolymerized to PET or transformed into upcycled materials.^9^ Glycolysis has also recently been found to outperform methanolysis, virgin PET, and enzymatic hydrolysis from an environmental impact perspective.^6,14^

**Figure 1:**
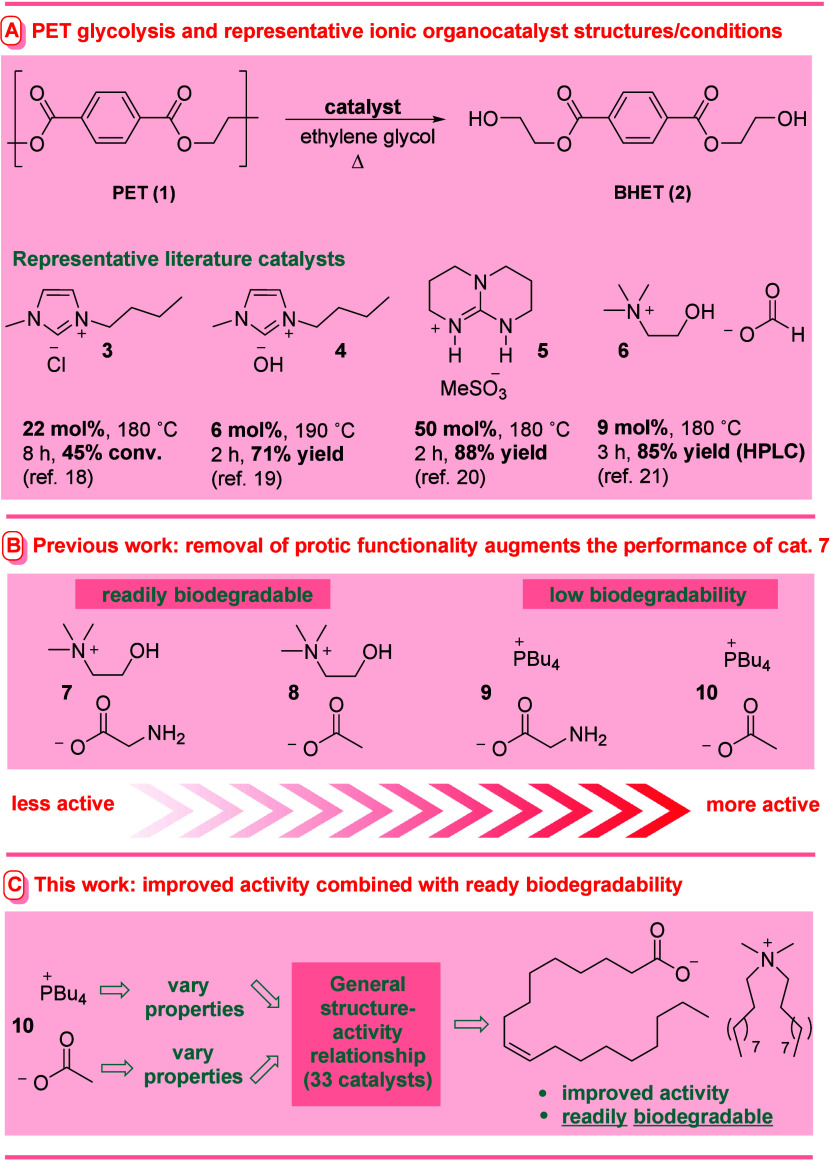
Catalytic PET glycolysis using ionic organocatalysts.

Several catalyst classes have been investigated as promoters of PET glycolysis, including organocatalysts, metal salts, heterogeneous systems, and biocatalysts.^9−14^ Among the most intensely studied catalyst systems is ionic liquids (ILs)^16,17^ due, in the main, to their low vapor pressure, tunable properties and high thermal stability. A recent comprehensive review detailed the performance of 121 distinct ILs in PET glycolysis.^17^ Of these, 63% contained at least one transition metal ion, which raises issues related to product metal contamination, and 37% were organocatalysts, representing a diverse array of anion–cation combinations. Of these, over 50% contained either a cholinium cation or an amino-acid-based anion (or both), which reflects the drive by researchers to design more biodegradable catalysts. In general, loadings using IL organocatalysts are higher than the most active metal-based systems, typically 5–20 mol or wt %. Considering the first IL organocatalysts for PET glycolysis were reported in 2009^18^ (and that one of the key advantages associated with their use is the ready exchangeability of anions, allowing the rapid alteration of catalyst properties; representative examples **3**–**6** are depicted in Figure 1A^19−21^); it is somewhat surprising that while limited endeavor to modify either anion basicity^19,21−23^ or cation polarity^24^ has been made within related catalyst systems, a general consensus regarding the IL organocatalyst structure–activity relationship (SAR) has not emerged.

This is possibly related to the variety of PET sources and reaction conditions (catalyst:PET:ethylene glycol ratios) and reaction temperatures employed in different studies, which complicate the comparative analysis. In a key study, D’Anna and co-workers^25^ found cholinium glycinate (**7**) to be superior to a range of evaluated related cholinium-ion based systems. We recently determined^26^ that removal of the biodegradable cholinium ion and/or polar functionality associated with **7** led to more active (yet ultimately less biodegradable due to the use of tetraalkylphosphonium ions^27^) catalysts (**8**–**10**, Figure 1B). The ultimate goal of a PET glycolysis catalyst design is application on an industrial scale, where the replacement of a biodegradable catalyst for a more active, yet less biodegradable system could complicate operations from a sustainability perspective. It was clear that in order to design more active systems that retain biodegradability, insight into the individual contribution of the catalyst ions to overall IL catalyst activity was required. Herein we report the results of a SAR study aimed at the elucidation of these contributions, which allowed the design/selection of biodegradable catalysts of considerably superior activity to **7** (Figure 1C).

## Results and Discussion

**Scheme 1:**
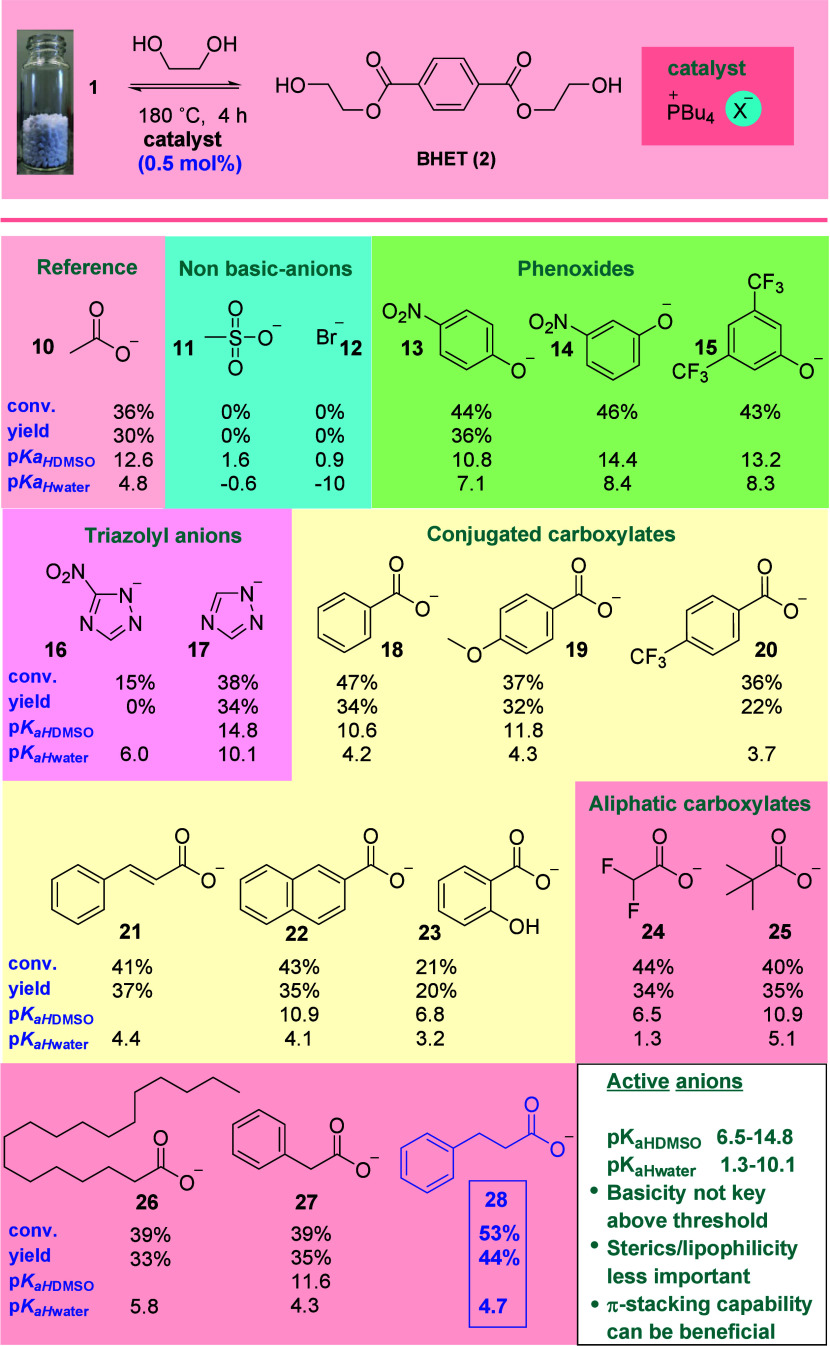
Influence of the Anion on the Performance of Tetrabutylphosphonium Ion-Based IL Catalysts

The study commenced with the glycolysis of commercial PET pellets at 180 °C in ethylene glycol (1:4 wt/wt) for 4 h (Scheme 1). All yield and conversion data represent a minimum of two repeated experiments with a maximum deviation of 3.5% from the mean (more usually 2.5%). In order to allow superior structures to be distinguished from less active counterparts, a highly challenging catalyst loading of 0.5 mol % was selected. Under these conditions, the reference catalyst from our previous study (*i.e*., **10**) promoted the reaction with 36% conversion and 30% yield of isolated, pure **2** after aqueous recrystallization. Subsequently, a library of glycolysis catalysts featuring the retention of the tetrabutylphosphonium cation and the systematic variation of the properties of the anion were synthesized and evaluated under identical conditions (Scheme 1). Nonbasic anions **11** and **12** failed to catalyze the reaction, while use of phenoxide-based catalysts bearing electron-withdrawing functionality (*i.e.*, **13**–**15**, the parent phenoxide catalyst decomposed) led to higher conversion relative to **10**. Interestingly, variation of phenoxide basicity over 3 orders of magnitude failed to affect the outcome significantly. The basic and nucleophilic triazolyl anion **16** proved more efficacious than its less basic nitro-substituted counterpart **17**, however, superiority over the phenoxides was not apparent.

In an attempt to improve the affinity of the anion for the heterogeneous, aromatic-ring-decorated PET surface, several conjugated carboxylate-based catalysts were also investigated. The benzoate-derived system **18** promoted the formation of **2** with higher conversion and marginally improved yield than catalyst **10**, however, either modulation of the anion’s electronic characteristics (*i.e*., **19** and **20**) or extension of the π-system (*i.e.*, **21** and **22**) failed to appreciably augment performance further. Consistent with our previous findings,^25^ the installation of H-bond donating functionality (*i.e.,***23**) was particularly deleterious. Instructively, less basic, bulkier, and more lipophilic-aliphatic analogues of **10** (*i.e.*, **24**–**26**, respectively) all exhibited increased efficacy, yet not of a magnitude indicating that these properties made crucial contributions to activity. Finally, returning to the concept of designing for affinity with the PET surface, variants of **10** incorporating aromatic rings attached via a saturated linker (*i.e.,***27** and **28**) were prepared and evaluated. The hydrocinnamate-based salt **28** was identified as a superior promoter compared to all other library members.

From the results depicted in Scheme 1, much regarding the contribution of the anion to the activity in these catalyst systems can be gleaned.

1. Somewhat contrary to two literature reports (using distinct cations),^19,22^ basicity was clearly not a key factor once above a threshold in these systems (*i.e.*, **11** and **12** were inactive, yet the basicities of other active catalysts which promoted the formation of **2** with similar yield varied over 8 orders of magnitude). Catalyst **24** is over 10^6^ times less basic than **10** in DMSO, yet it possesses similar activity.
2. Anion steric bulk and lipophilicity could be dramatically increased without damaging activity (*i.e.*, catalysts **10**, **26**, and **27**).
3. H-bond donating functionality in proximity to the base was not advantageous (*i.e.*, **23**).
4. Aromatic functionality in the anion was desirable, especially if linked to a carboxylate via a linker (*i.e.*, **13**–**15**, **18**, **21**, **22**, **27**, and **28**).

**Scheme 2:**
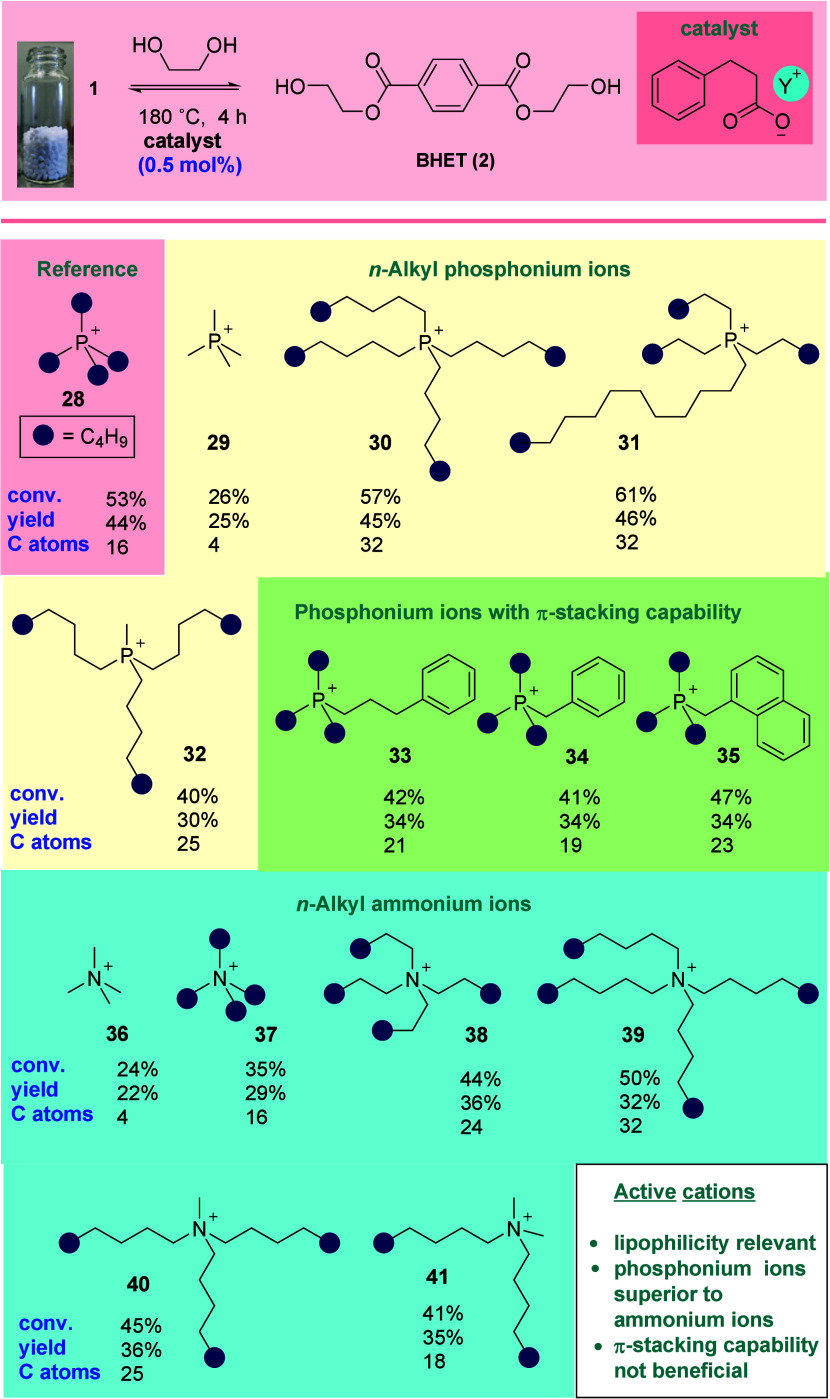
Influence of the Cation on the Performance of Hydrocinnamate Ion-Based IL Catalysts

In a similar fashion, the influence of the cation was next probed through the synthesis of a library of catalysts that share the hydrocinnamate ion, but incorporate systematically varied cations (Scheme 2). These were evaluated in the glycolysis of PET under identical conditions to those utilized in the study of anions. Here the importance of cation lipophilicity^28^ was immediately apparent; catalyst **29**, incorporating a small tetramethylphosphonium cation, was strikingly inferior to the reference catalyst **28**. Subsequent augmentation of the cation size (and lipohilicity, *i.e.*, **30**–**32**) led to improvements in conversion and marginal gains in product yield relative to **28**. The installation of aromatic functionality via a tether produced significantly less-active phosphonium-based catalysts (**25**–**33**), presumably due to increased association with the hydrocinnamate anion. Ammonium-based cations **36**–**41** were found to be less useful than phosphonium counterparts; however, the same trends with regards to the relationship between cation size and activity were discernible.

**Scheme 3:**
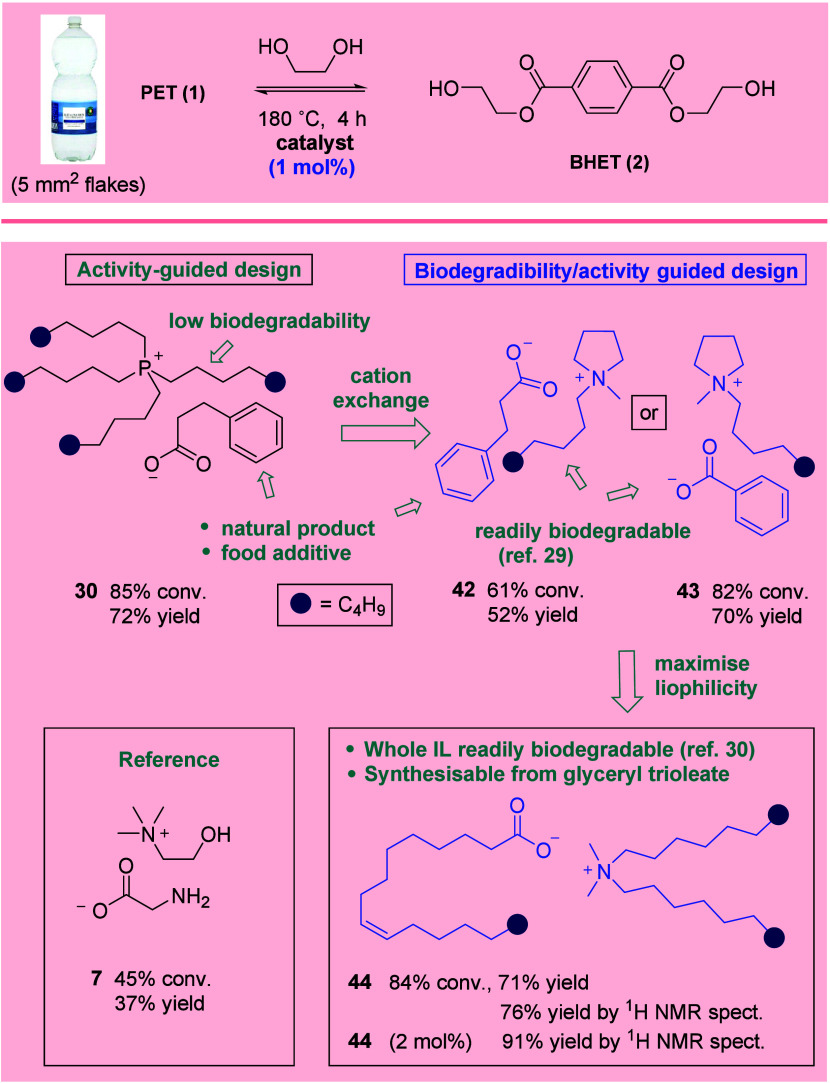
Design/Selection of Novel Biodegradable Active IL Catalysts

The question now became one of leveraging the emerging SAR toward the design of biodegradable yet highly active catalysts. In order to better reflect a real-world recycling scenario, commercial PET pellets were exchanged for 5 mm × 5 mm flakes cut from water bottles purchased from a local supermarket. These were glycolysed at 180 °C in the presence of 1 mol % catalyst. Catalyst **30**, identified as highly efficacious in the activity-guided studies outlined in Schemes 1 and 2, proved capable of mediating the formation of **2** in high yield, even at this extraordinarily low loading for an IL-based system (Scheme 3). While hydrocinnamic acid is a common metabolite and food additive (sweetner, antioxidant) of little biodegradatory concern, tetraalkylphosphonium ions are notoriously poor substrates for biodegradation.^27^ A substitute cation lipophilic enough to drive high catalyst activity (and devoid of polar/H-bond-donating or -accepting functionality) while also being known to be readily biodegradable was required. This presented a challenge, as the majority of known biodegradable cations are not highly lipophilic and incorporate polar/protic moieties.^26^ It appears that, in the systems under study, cation biodegradability and cation contribution to activity are generally antagonistic. Eventually, a literature search led to the *N*-methyl, *N*-octylpyrrolidinium cation, the chloride salt of which was found by Stolte *et al*. to be readily biodegradable (100% biotic deg., 28 d).^29^ The combination of this cation with either previously identified hydrocinnamate or benzoate anions gave rise to highly active catalysts (**42** and **43**, respectively; note: sodium benzoate is used as a positive control in biodegradation assays^29^) comprising both biodegradable anions and cations. It is interesting to note that here, the benzoate catalyst proved more active than its hydrocinnamate analogue. Further mining of the literature allowed the identification of the highly lipophilic yet demonstrably biodegradable IL **44** (87% biotic deg., 28 d)^30^ comprising both cation and anion functionality expected (Schemes 1 and 2) to engender high catalyst activity. The material was synthesized and excellent catalytic activity was confirmed. All three catalysts (*i.e*., **42**–**44**) were considerably superior to cholinium glycinate (**7**).

In all experiments outlined above, yields of **2** are isolated after crystallization under scrupulously identical conditions, which results in some loss of **2** remaining in the mother liquor. To demonstrate the true efficacy of catalyst **44**, the glycolysis experiment was repeated at 2 mol % loading, resulting in a 91% yield of **2**, determined by ^1^H NMR spectroscopy using (*E*)-stilbene as an internal standard. While the oleic acid and didodecylammonium bromide used to prepare **44** are not expensive, we note that **44** (and similar fatty acid derived catalysts) could be conceivably obtained from the saponification and ion exchange of inexpensive and sustainably sourced triglycerides (*e.g*., from olive oil). Recycling of **44** was not efficient due to partial degradation: 20% of **44** remains post-glycolysis, determined by quantitative ^1^H NMR spectroscopic analysis, see SI (similar to catalyst **7**). Either immobilization of the catalyst on a suitable support or catalyst structural modification to minimize degradation could be considered.

## Conclusion

In summary, beginning with a previously studied catalyst cholinium glycinate (**7**) and its biodegradatory suspect yet more active analogue **10**, a SAR study provided valuable and somewhat unexpected insight regarding the contributions the anion and cation make to catalyst efficacy. Anion basicity in tetrabutylphosphonium-based catalysts proved largely inconsequential above a threshold, while anion bulk and lipophilicity also appeared to be of minor importance. It seems clear that once polar protic functionality is avoided, considerable variation in anion structure is compatible with high catalyst activity. The incorporation of potentially π-stacking functionality was advantageous.

We had previously shown the cholinium ion to be suboptimal from an efficacy perspective:^25^ here it was found that when combined with a hydrocinnamate counterion, that simple ammonium and (in particular) phosphonium cation lipophilicity is of considerable importance. The use of small relatively polar cations resulted in poor catalyst performance, while larger, more lipophilic homologues brought about more active systems (an SAR which it is now clear represents a challenge associated with the design active yet biodegradable IL-based PET glycolysis catalysts). This approach allowed the design of a highly active IL catalyst, **30** capable of operating at loadings considerably lower than those associated with IL catalysts in the literature. The SAR insight garnered during these studies allowed the initial activity-guided design paradigm to evolve into a biodegradability/activity-based approach, which facilitated the development of two new glycolysis catalysts (**42** and **43**) comprising biodegradable anions and cations (with structural characteristics expected to be compatible with high catalytic effiacy), and one known, readily biodegradable IL **44** with similar desirable properties. All three exhibited catalyst activity far in excess of that associated with cholinium glycinate (**7**) at 1 mol % loadings. Further studies to explore the mode of action of these systems and the potential of a biodegradability/activity-guided approach are underway.

## Supporting Information Available

Materials, experimental procedures, characterization data, NMR spectra (PDF)
