# Supplementary material for: Ionic Liquid Catalysts for Poly(ethylene terephthalate) Glycolysis: Use of Structure Activity Relationships to Combine Activity with Biodegradability
Source: ACS Sustain Chem Eng. 2025 Jan 16;13(4):1424–30. doi: 10.1021/acssuschemeng.4c08491 (PMC11795626; doi:10.1021/acssuschemeng.4c08491)
Supplement: Supplementary file 1 — sc4c08491_si_001.pdf [file sc4c08491_si_001.pdf]

Electronic Supplementary Information

Ionic liquid catalysts for poly(ethylene terephthalate) glycolysis: use of structure activity relationships to combine activity with biodegradability

Lorenzo Pedrini, Chiara Zappelli and Stephen J. Connon

School of Chemistry, Trinity Biomedical Sciences Institute,

Trinity College Dublin, 152-160 Pearse Street,

Dublin 2, Ireland

E-mail: connons@tcd.ie.

Number of pages: 163

Number of figures: 4

Number of tables: 1

Table of Contents

1 General 3
2 General procedure for PET glycolysis 4
2.1 General procedure A for PET glycolysis 4
2.2 General procedure B for PET glycolysis - yield determined by ^{1}H NMR spectroscopy . 7
3 Catalyst decomposition assessment. 9
3.1 Didecyldimethylammonium oleate (**44**) decomposition assessment. 9
3.2 Recyclability assessment of catalytic activity of **44** 11
3.3 Choline glycinate (**7**) decomposition assessment. 12
4 Synthesis of ionic catalysts: procedures 13
5 pK_a data associated with Scheme 1 – literature data 53
6 References 54
7 NMR Spectra 58

1 General

Proton Nuclear Magnetic Resonance (NMR) spectra were recorded on Bruker DPX 400 MHz, Bruker Avance II 600 MHz and Agilent 400DD2 spectrometers, using $CDCl_3$, DMSO-d_6 and D_2O as solvents, and referenced relative to residual $CHCl_3$ (δ_H = 7.26 ppm, δ_C = 77.16 ppm), DMSO (δ_H = 2.50 ppm, δ_C = 39.52 ppm) or D_2O (δ_H = 4.79 ppm). Chemical shifts are reported in ppm and coupling constants (*J*) in Hertz. Carbon NMR spectra were recorded on the same instruments (100 MHz and 150 MHz respectively) with total proton decoupling. Phosphorus NMR spectra were recorded on the Bruker DPX400 or Agilent 400DD2 machine (162 MHz). Fluorine NMR spectra were recorded on the Bruker DPX400 or Agilent 400DD2 machine (376 MHz). HSQC, HMBC, TOCSY, NOE, EXSY and ROESY NMR experiments were used to aid assignment of NMR peaks when required. Infrared spectra were obtained on a Perkin Elmer Spectrum 100 FT-IR spectrometer equipped with a universal ATR sampling accessory. ESI mass spectra were acquired using a Waters Micromass LCT- time of flight mass spectrometer (TOF), interfaced to a Waters 2690 HPLC. The instrument was operated in either positive or negative mode as required. Agilent tuning mix APCI-TOF was used to calibrate the system. Flash chromatography was carried out using gel, particle size 0.035-0.075 mm. TLC analysis was performed on precoated 60 F_{254} slides and visualized by either UV irradiation, $KMnO_4$, ninhydrin staining or phosphomolybdic acid staining as appropriate. Anhydrous acetonitrile was obtained from "pure solv MD-4EN" solvent purification system. Glycolysis experiments were accomplished with Radleys Carousel 12 Plus Reaction Station. Polyethylene terephthalate (granule size 3-5 mm) was purchased from Goodfellow Cambridge Limited and was used as provided. Clear polyethylene terephthalate bottles were purchased from a local supermarket, washed with water and soap and dried overnight. Ethylene glycol (anhydrous) was purchased from Sigma Aldrich – 324558. Anion exchange resin Amberlite-OH IRN78 (Supelco, strongly basic, total exchange capacity (OH) 1.25 eq/L) was purchased from Sigma Aldrich. Unless otherwise noted, all commercially available compounds were used as provided, without any further purification. Choline glycinate (**7**) was prepared following a literature procedure.[1]

2 General procedure for PET glycolysis

2.1 General procedure A for PET glycolysis

An oven dried carousel flask was charged with polyethylene terephthalate (pellets or flakes, 1.0000 g ± 0.0050 g), ethylene glycol (3.60 mL) and catalyst (0.5 mol%).[a] The flask was placed under a nitrogen atmosphere (balloon), and the reaction was stirred for 4 h at 180 °C.[b] The flask was removed from the carousel and was left to cool for 5 min. The reaction mixture was then diluted with deionised water (400 mL), and the resulting aqueous mixture was vigorously stirred for 20 min. Utilising a Bucker funnel the mixture was filtered and the pre-weighed filter paper was dried overnight in a vacuum oven at 60 °C to determine levels of unreacted PET residue and oligomers. The filtrate was concentrated in *vacuo* to a total of 50 mL and cooled to 4 °C overnight to enhance crystallisation. The resulting crystals were filtered and dried overnight in a vacuum oven at 60 °C.

[a]Mol% of catalyst is related to the number of moles of monomeric units (5.2 mmol) present in 1 g of polymer.

[b]Temperature was measured in an adjacent carousel flask filled with the equivalent amount of ethylene glycol and equipped with a thermometer.

Note: all yield and conversion data derive from duplicate experiments agreeing within 5%.

Synthesis *bis*(2-hydroxyethyl)terephthalate (BHET) from PET pellets

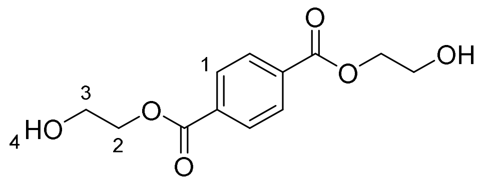

BHET was synthesised using General procedure A, polyethylene terephthalate pellets (1.0000 g ± 0.0050 g), ethylene glycol (3.60 mL) and **28** (0.0128 g, 0.026 mmol, 0.5 mol%).[a] BHET was isolated as a white solid (0.59 g, 45%). M.p. 108.0-109.5 °C (lit.,[2] M.p. 106-109 °C).

[a]Mol% of catalyst is related to the number of moles of monomeric units (5.2 mmol) present in 1 g of polymer.

Spectral data for this compound were consistent with those in literature.[2]

δ_H (400 MHz, DMSO-d_6): 8.12 (s, 4H, *H*-1), 4.95 (t, 2H, *J* 5.6, *H*-4), 4.32 (t, 4H, *J* 4.8, *H*-2), 3.77-3.67 (m, 4H, *H*-3) ppm.

δ_C (100 MHz, DMSO-d_6): 165.2, 133.8, 129.5, 67.0, 59.0 ppm.

Synthesis *bis*(2-hydroxyethyl)terephthalate (BHET) from PET flakes

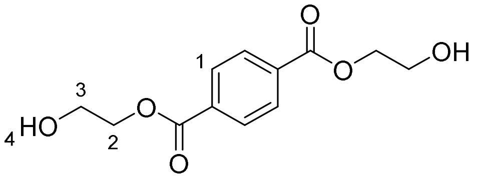

BHET was synthesised using the General procedure A, polyethylene terephthalate flakes (1.0000 g ± 0.0050 g, 5 mm x 5 mm), ethylene glycol (3.60 mL) and **44** (0.0316 g, 0.052 mmol, 1 mol%).[a] BHET was isolated as a white solid (0.95 g, 72%). M.p. 108.0-109.5 °C (lit.,[2] M.p. 106-109 °C).

[a]Mol% of catalyst is related to the number of moles of monomeric units (5.2 mmol) present in 1 g of polymer.

Spectral data for this compound were consistent with those in literature.[2]

δ_H (400 MHz, DMSO-d_6): 8.12 (s, 4H, *H*-1), 4.95 (t, 2H, *J* 5.6, *H*-4), 4.32 (t, 4H, *J* 4.8, *H*-2), 3.77-3.67 (m, 4H, *H*-3) ppm.

δ_C (100 MHz, DMSO-d_6): 165.2, 133.8, 129.5, 67.0, 59.0 ppm.

2.2 General procedure B for PET glycolysis - yield determined by ^{1}H NMR spectroscopy

An oven dried carousel flask was charged with polyethylene terephthalate flakes (1.0000 g ± 0.0050 g, 5 mm x 5 mm), ethylene glycol (3.6 mL, 4 g) and the appropriate loading of **44**.[a] The flask was placed under a nitrogen atmosphere (balloon), and the reaction was stirred for 4 h at 180 °C. The flask was removed from the carousel and was left to cool for 5 min. The reaction mixture was transferred in a 50 mL beaker, the internal standard was added ((*E*)-stilbene, 2 mmol, 0.3605 g) and the mixture was diluted by adding 15 mL of dimethylsulfoxide (DMSO). The cloudy suspension was vigorously stirred while keeping the plate temperature to 50 °C until a clear solution resulted. A sample of the reaction mixture (~ 3 drops) was diluted to ca. 1 mL with DMSO-d_6. The obtained sample was analysed by qH{^{13}C}NMR to determine BHET yield (89%).[b,c]

[a]Mol% of catalyst is related to the number of moles of monomeric units (5.2 mmol) present in 1 g of polymer.

[b]qH{^{13}C}NMR, 400 MHz, D1 = 15 s, GARP-mediated ^{13}C decoupling.[3]

[c]Yield value was calculated by subtraction of the resonance associated with the soluble oligomeric fraction at 4.68 ppm (4H) from the integral of the -CH_2- resonance at 3.72 ppm (4H).[4]

Note: all yield and conversion data derive from duplicate experiments agreeing within 5%.

Resonances relevant for yield calculation:

A	-	3.72 ppm	BHET (4H) + BHET dimer (4H)		
B	-	4.68 ppm	BHET dimer (4H)		
C	-	7.38 ppm	(*E*)-stilbene (4H)	-	set reference integration to 2.00

$$\%yield = \frac{(int.A - int.B)(C\ mmol)}{theoretical\ mmol} * 100 = \frac{(int.A - int.B)(1\ mmol)}{5.2\ mmol} * 100$$

Synthesis *bis*(2-hydroxyethyl)terephthalate (BHET) from PET flakes - yield determined by ^{1}H NMR spectroscopy.

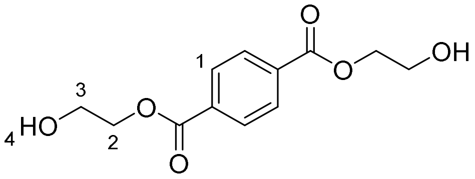

BHET was synthesised using General procedure B, polyethylene terephthalate flakes (1.0000 g ± 0.0050 g, 5 mm x 5 mm), ethylene glycol (3.60 mL) and **44** (0.0632 g, 0.104 mmol, 2 mol%).[a] The obtained sample was analysed by qH{^{13}C}NMR to determine the BHET yield (91%).

[a]Mol% of catalyst is related to the number of moles of monomeric units (5.2 mmol) present in 1 g of polymer.

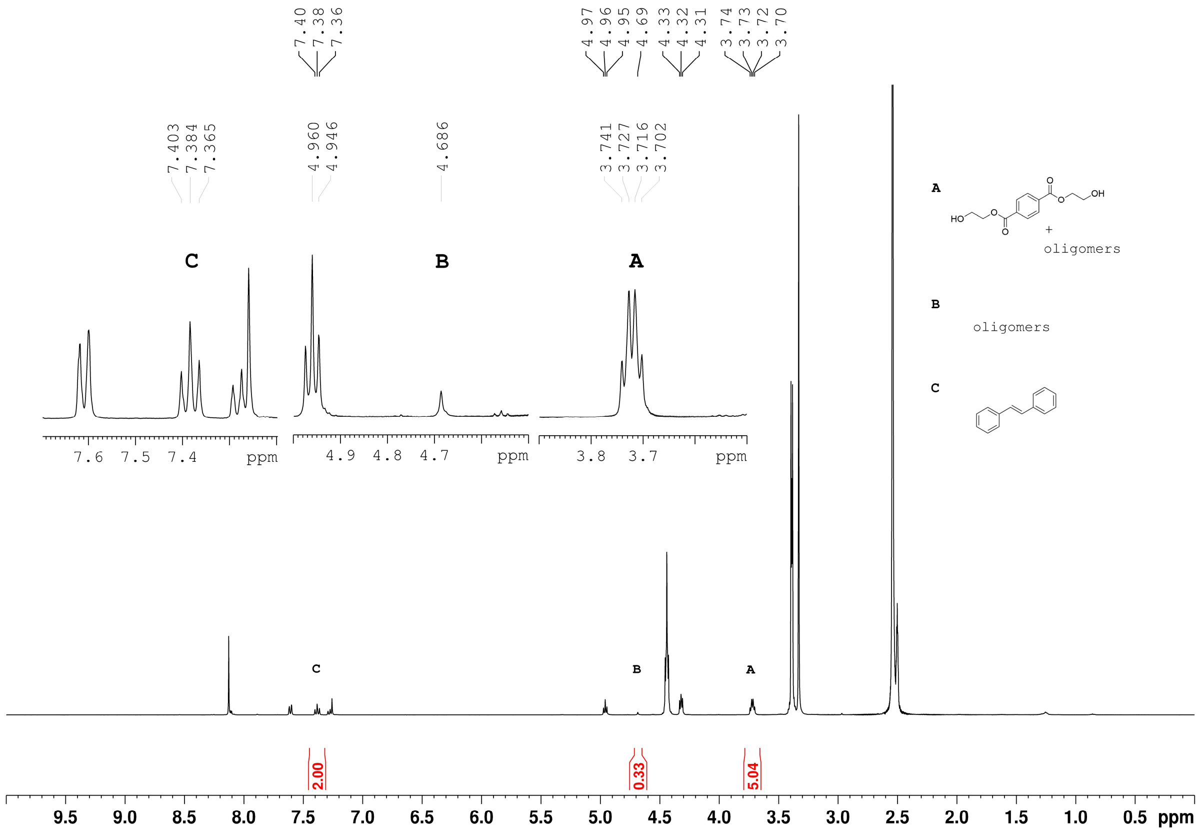

Figure S1. qH{^{13}C}NMR (400 MHz, DMSO-*d$_6$*, D1 = 15 s, GARP-mediated ^{13}C decoupling) of PET:EG (1:4, *wt:wt*) experiment for NMR yield calculation, internal standard (*E*)-stilbene.

3 Catalyst decomposition assessment.

3.1 Didecyldimethylammonium oleate (44) decomposition assessment.

A small volume carousel tube was charged with **44** (0.0079 g, 0.013 mmol) and ethylene glycol (1 g, 0.9 mL).[a] The mixture was stirred at 180 °C for 1 h, 2 h, 3 h and 4 h (separate experiments for each time point). Ethylene glycol was evaporated *in vacuo* at 100 °C. The residue was taken up $CDCl_3$ and analysed by ^{1}H NMR spectroscopy (Figure S2).

The decomposition of **44** was studied quantitatively under the reaction conditions using a carousel tube charged with **44** (0.0316 g, 0.052 mmol) and ethylene glycol (4 g, 3.6 mL).[a] Decomposition led to 20% (0.010 mmol) of the initial amount of **44** being detected by quantitative ^{1}H NMR spectroscopic analysis with an internal standard (*E*-stilbene, 0.0094g, 0.052 mmol) (Figure S3).

[a] Catalyst concentration is identical to that employed in the experiments outlined in Section 2 (1 mol% experiment).

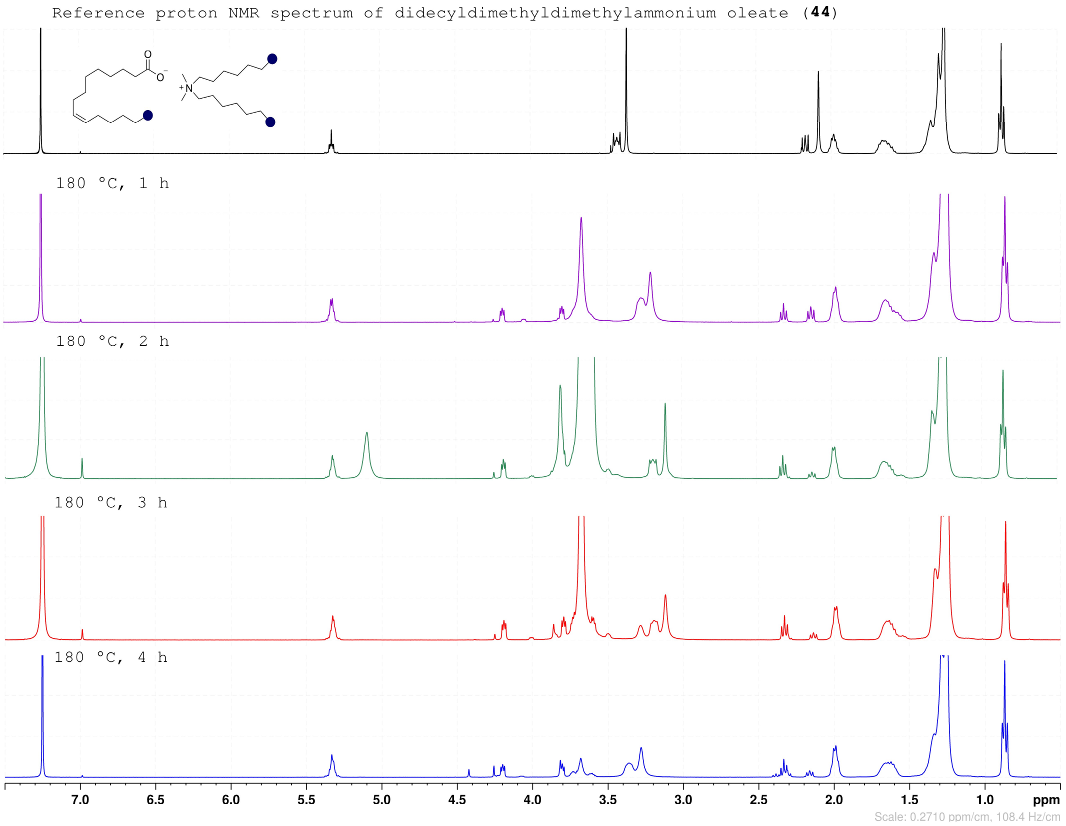

Figure S2: Decomposition assessment of 44 by ^{1}H NMR (400 MHz) in $CDCl_3$.

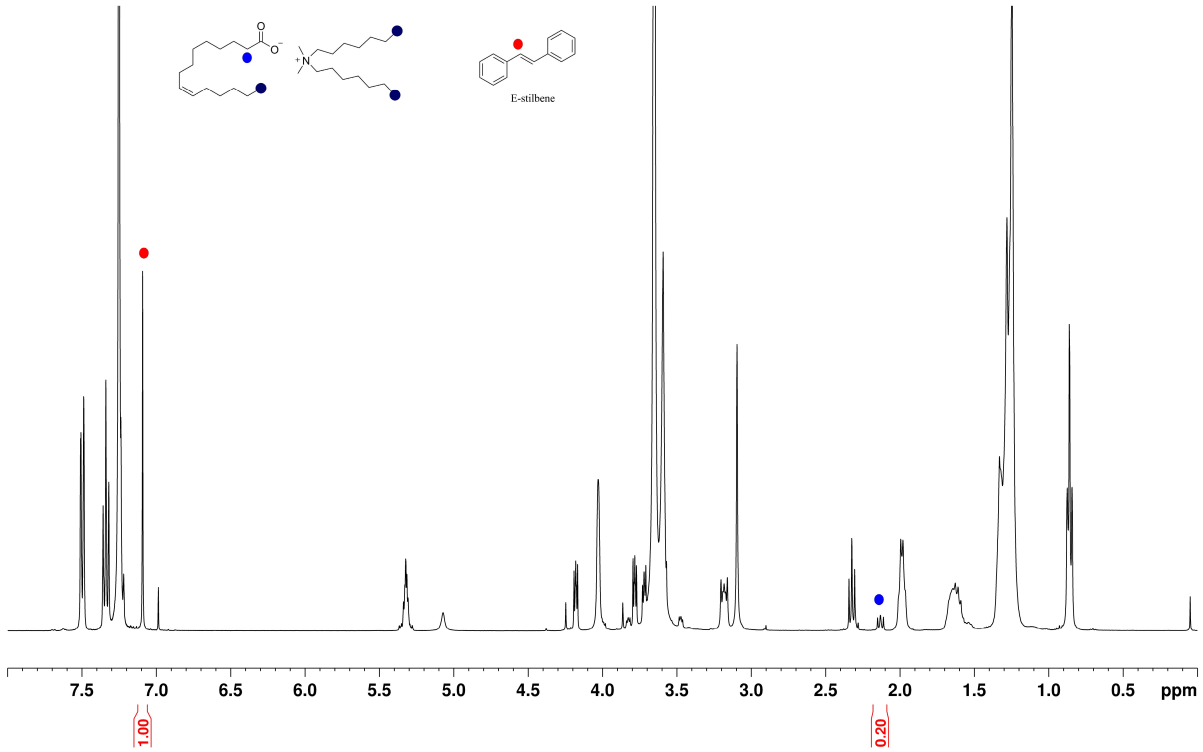

Figure S3: Decomposition of 44 by quantitative ^{1}H NMR (400 MHz) spectroscopic analysis in ($CDCl_3$) using equimolar amounts of (E)-stilbene as the internal standard.

3.2 Recyclability assessment of catalytic activity of 44

A carousel tube was charged with **44** (0.0316 g, 0.052 mmol, 1 mol%) and ethylene glycol (3.60 mL).[a] The resulting mixture was stirred for 4 h at 180 °C. Ethylene glycol was evaporated *in vacuo* at 100 °C. The residue was diluted with ethylene glycol (3.60 mL) and polyethylene terephthalate flakes (1.0000 g ± 0.0050 g, 5 mm x 5 mm) were added to the mixture. BHET was synthesised using the General procedure A. BHET was isolated as a white solid (0.70 g, 53%). M.p. 105.0-107.5 °C (lit.,[2] M.p. 106-109 °C).

[a]Mol% of catalyst is related to the number of moles of monomeric units (5.2 mmol) present in 1 g of polymer.

3.3 Choline glycinate (7) decomposition assessment.

A small volume carousel tube was charged with **7** (0.0023 g, 0.013 mmol) and ethylene glycol (0.9 mL).[a] The mixture was stirred at 180 °C for 4 h. Ethylene glycol was evaporated *in vacuo* at 100 °C. The residue was taken up in D_2O and analysed by ^1H NMR spectroscopy.

[a] Catalyst concentration is identical to that employed in the experiments outlined in Section 2 (1 mol% experiment).

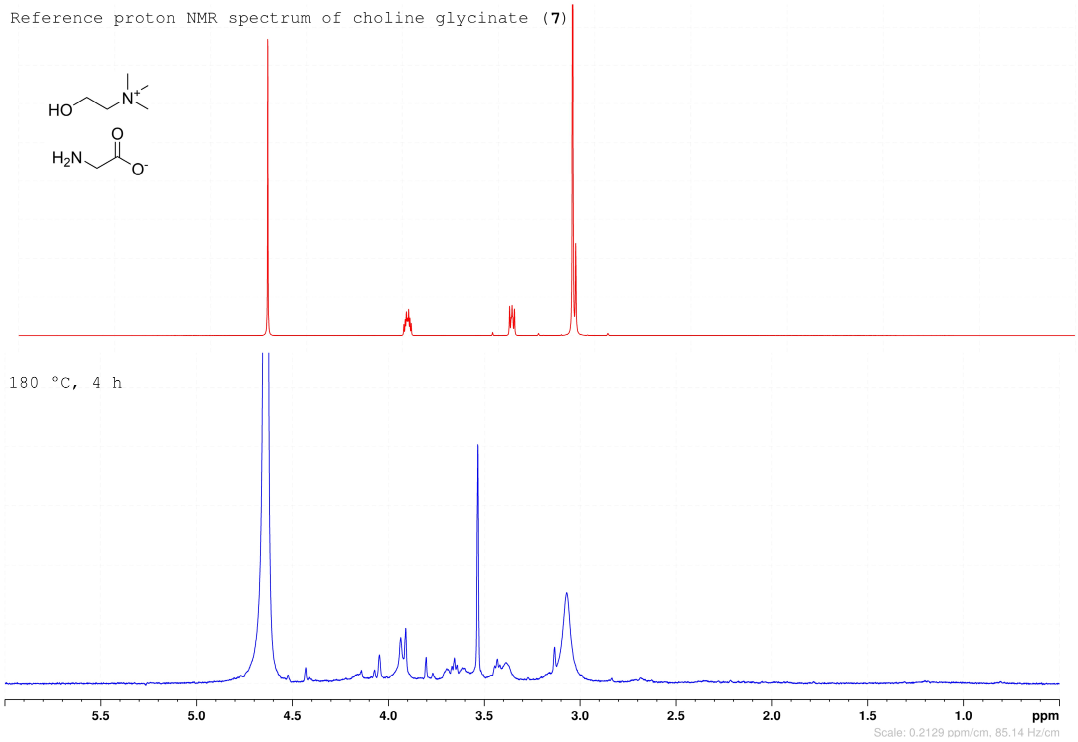

Figure S4: Decomposition assessment of 7 by ^1H NMR (400 MHz) in D_2O.

4 Synthesis of ionic catalysts: procedures

Tetrabutylphosphonium acetate (10)

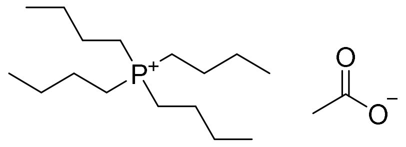

A 100 mL round-bottomed flask was charged with MeOH (50 mL). Tetrabutylphosphonium hydroxide in H_2O (40 wt.%, 3.50 mL, 5.0 mmol) was added dropwise to the solution and the resulting mixture was stirred for 5 min. Acetic acid (0.29 mL, 5.0 mmol) was added dropwise and the resulting solution was stirred for 16 h. The solvent was removed under reduced pressure and the product was dried under vacuum for 8 h at 60 °C to yield **10** as a hygroscopic colourless gum (1.54 g, 98%).

Spectral data for this compound were consistent with those in literature.[5]

δ_H (400 MHz, $CDCl_3$):	2.46-2.34 (m, 8H), 1.91 (s, 3H), 1.54-1.42 (m, 16H), 0.93 (t, 12H, *J* 7.0) ppm.
δ_C (100 MHz, $CDCl_3$):	176.8, 25.6, 24.0 (d, *J* 15.3), 23.8 (d, *J* 4.8), 18.6 (d, *J* 47.4), 13.5 ppm.
HRMS (*m/z* – ESI$^+$):	Found: 259.2552 (M^+) $C_{16}H_{36}P^+$ Requires: 259.2549.

Tetrabutylphosphonium mesylate (11)

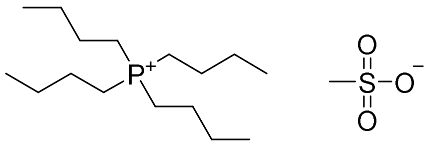

A 50 mL round-bottomed flask was charged with MeOH (25 mL). Tetrabutylphosphonium hydroxide in H_2O (40 wt.%, 1.75 mL, 2.5 mmol) was added dropwise to the solution and the resulting mixture was stirred for 5 min. Methanesulfonic acid (0.16 mL, 2.5 mmol) was added dropwise and the resulting solution was stirred for 16 h. The solvent was removed under reduced pressure and the product was dried under vacuum for 8 h at 60 °C to yield **11** as a hygroscopic white solid (0.89 g, 99%).[a]

Spectral data for this compound were consistent with those in literature.[6,7]

[a]The hygroscopic solid was not amenable to melting point determination in air.

δ_H (400 MHz, DMSO-d_6):	2.29 (s, 3H), 2.24-2.12 (m, 8H), 1.54-1.34 (m, 16H), 0.92 (t, 12H, *J* 7.1) ppm.
δ_C (100 MHz, DMSO-d_6):	39.8, 23.3 (d, *J* 15.5), 22.6 (d, *J* 4.4), 17.3 (d, *J* 47.6), 13.2 ppm.
HRMS (*m/z* – ESI$^+$):	Found: 259.2549 (M^+) $C_{16}H_{36}P^+$ Requires: 259.2549.
ν_{max} (neat)/cm^{-1}:	2957, 2932, 2871, 1466, 1379, 1317, 1189, 1038, 919, 808, 761, 725.

Tetrabutylphosphonium 4-nitrophenolate (13)

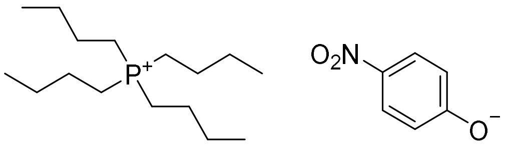

A 50 mL round-bottomed flask was charged with 4-nitrophenol (0.35 g, 2.5 mmol) and MeOH (25 mL). Tetrabutylphosphonium hydroxide in H_2O (40 wt.%, 1.75 mL, 2.5 mmol) was added dropwise to the solution and the resulting mixture was stirred for 16 h. The solvent was removed under reduced pressure and the product was dried under vacuum for 8 h at 60 °C to yield **13** as a hygroscopic yellow solid (0.98 g, 99%).[a]

[a]The hygroscopic solid was not amenable to melting point determination in air.

δ_H (400 MHz, $CDCl_3$):	7.93 (d, 2H, *J* 9.5), 6.24 (d, 2H, *J* 9.5), 2.18-2.03 (m, 8H), 1.53-1.38 (m, 16H), 0.95 (t, 12H, *J* 6.9) ppm.
δ_C (100 MHz, $CDCl_3$):	180.7, 130.3, 128.03, 119.6, 23.9 (d, *J* 15.2), 23.6 (d, *J* 4.8), 18.6 (d, *J* 47.5), 13.4 ppm.
δ_P (162 MHz, $CDCl_3$):	33.1 ppm.
HRMS (*m/z* – ESI$^+$):	Found: 259.2555 (M^+) $C_{16}H_{36}P^+$ Requires: 259.2549.
ν_{max} (neat)/cm^{-1}:	2961, 2928, 2872, 1584, 1527, 1463, 1385, 1255, 1156, 1098, 998, 909, 847, 760, 709.

Tetrabutylphosphonium 3-nitrophenlate (14)

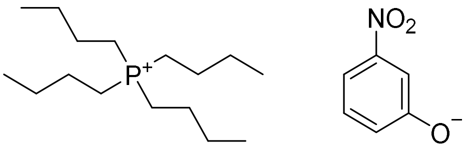

A 50 mL round-bottomed flask was charged with 3-nitrophenol (0.35 g, 2.5 mmol) and MeOH (25 mL). Tetrabutylphosphonium hydroxide in H_2O (40 wt.%, 1.75 mL, 2.5 mmol) was added dropwise to the solution and the resulting mixture was stirred for 16 h. The solvent was removed under reduced pressure and the product was dried under vacuum for 8 h at 60 °C to yield **14** as a hygroscopic dark red solid (0.99 g, 99%).[a]

[a]The hygroscopic solid was not amenable to melting point determination in air.

$\delta_{H\{C\}}$ (400 MHz, DMSO-*d6*):	6.82 (app. t, 1H), 6.62 (app. t, 1H), 6.58 (dd, 1H, *J* 7.7, 2.3), 6.34 (dd, 1H, *J* 8.3, 2.1), 2.23-2.11 (m, 8H), 1.52-1.34 (m, 16H), 0.91 (t, 12H, *J* 7.1) ppm.
δ_C (100 MHz, DMSO-*d6*):	172.4, 149.7, 128.4, 126.8, 111.2, 99.9, 23.4 (d, *J* 15.6), 22.6 (d, *J* 4.4), 17.3 (d, *J* 47.5), 13.3 ppm.
δ_P (162 MHz, DMSO-*d6*):	33.8 ppm.
HRMS (*m/z* – ESI$^+$):	Found: 259.2555 (M^+) $C_{16}H_{36}P^+$ Requires: 259.2549.
ν_{max} (neat)/cm^{-1}:	2960, 2930, 2872, 1603, 1499, 1464, 1436, 1330, 1309, 1282, 1236, 1202, 1099, 1071, 983, 909, 849, 816, 736, 674.

Tetrabutylphosphonium 3,5-bis(trifluoromethyl)phenolate (15)

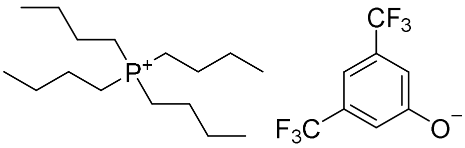

A 50 mL round-bottomed flask was charged with MeOH (25 mL). Tetrabutylphosphonium hydroxide in H_2O (40 wt.%, 1.75 mL, 2.5 mmol) was added dropwise to the solution and the resulting mixture was stirred for 5 min. 3,5-*bis*(trifluoromethyl)phenol (0.40 mL, 2.5 mmol) was added dropwise and the resulting solution was stirred for 16 h. The solvent was removed under reduced pressure and the product was dried under vacuum for 8 h at 60 °C to yield **15** as an off-white gum (1.21 g, 99%).

$\delta_{H\{C\}}$ (400 MHz, DMSO-d_6):	6.40 (s, 2H), 6.20 (s, 1H), 2.26-2.11 (m, 8H), 1.54-1.34 (m, 16H), 0.92 (t, 12H, *J* 7.1) ppm.
δ_C (100 MHz, DMSO-d_6):	171.8, 130.4 (q, *J* 30.1), 124.8 (q, *J* 272.5), 118.0 (app. d, *J* 2.5), 96.4 (br. s), 23.4 (d, *J* 15.6), 22.6 (d, *J* 4.4), 17.3 (d, *J* 47.8), 13.2 ppm.
δ_P (162 MHz, DMSO-d_6):	33.8 ppm.
δ_F (376 MHz, DMSO-d_6):	61.6 ppm.
HRMS (*m/z* – ESI^+):	Found: 259.2550 (M^+) $C_{16}H_{36}P^+$ Requires: 259.2549.
ν_{max} (neat)/cm^{-1}:	3659, 2980, 2971, 2935, 2880, 1582, 1479, 1402, 1268, 1155, 1107, 983, 862, 839, 811, 726, 704, 678.

Tetrabutylphosphonium nitrotriazolate (16)

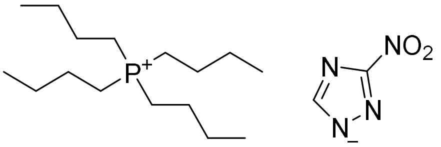

A 50 mL round-bottomed flask was charged with 3-nitro-1,2,4-triazole (0.29 g, 2.5 mmol) and MeOH (25 mL). Tetrabutylphosphonium hydroxide in H_2O (40 wt.%, 1.75 mL, 2.5 mmol) was added dropwise to the solution and the resulting mixture was stirred for 16 h. The solvent was removed under reduced pressure and the product was dried under vacuum for 8 h at 60 °C to yield **16** as a hygroscopic off-white solid (0.93 g, 94%).[a]

[a]The hygroscopic solid was not amenable to melting point determination in air.

δ_H (400 MHz, DMSO-*d6*):	7.79 (s, 1H), 2.24-2.10 (m, 8H), 1.52-1.34 (m, 16H), 0.91 (t, *J* 7.0) ppm.
δ_C (100 MHz, DMSO-*d6*):	165.6, 151.2, 23.4 (d, *J* 15.6), 22.6 (d, *J* 4.4), 17.3 (d, *J* 47.6), 13.3 ppm.
δ_P (162 MHz, DMSO-*d6*):	33.8 ppm.
HRMS (*m/z* – ESI$^+$):	Found: 259.2550 (M^+) $C_{16}H_{36}P^+$ Requires: 259.2549.
ν_{max} (neat)/cm^{-1}:	3089, 2960, 2931, 2873, 1510, 1466, 1376, 1350, 1280, 1149, 1097, 1057, 977, 906, 830, 720, 665.

Tetrabutylphosphonium triazolate (17)

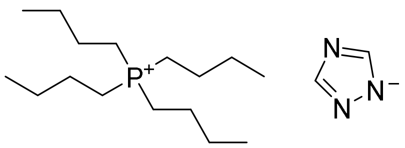

A 50 mL round-bottomed flask was charged with 1,2,4-triazole (0.17 g, 2.5 mmol) and MeOH (25 mL). Tetrabutylphosphonium hydroxide in H_2O (40 wt.%, 1.75 mL, 2.5 mmol) was added dropwise to the solution and the resulting mixture was stirred for 16 h. The solvent was removed under reduced pressure and the product was dried under vacuum for 8 h at 60 °C to yield **17** as a hygroscopic white solid (0.81 g, 99%).[a]

Spectral data for this compound were consistent with those in literature.[8]

[a]The hygroscopic solid was not amenable to melting point determination in air.

δ_H (400 MHz, DMSO-*d*$_6$):	7.59 (s, 2H), 2.25-2.11 (m, 8H), 1.53-1.34 (m, 16H), 0.91 (t, 12H, *J* 7.0) ppm.
δ_C (100 MHz, DMSO-*d*$_6$):	148.8, 23.3 (d, *J* 15.6), 22.6 (d, *J* 4.4), 17.3 (d, *J* 47.6), 13.2 (d, *J* 0.6) ppm.
HRMS (*m/z* – ESI^+):	Found: 259.2552 (M^+) $C_{16}H_{36}P^+$ Requires: 259.2549.

Tetrabutylphosphonium benzoate (18)

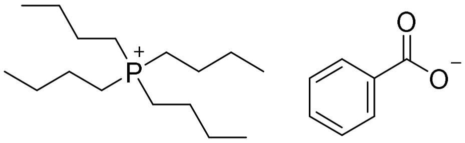

A 100 mL round-bottomed flask was charged with benzoic acid (0.61 g, 2.5 mmol) and MeOH (50 mL). Tetrabutylphosphonium hydroxide in H_2O (40 wt.%, 1.75 mL, 2.5 mmol) was added dropwise to the solution and the resulting mixture was stirred for 16 h. The solvent was removed under reduced pressure and the product was dried under vacuum for 8 h at 60 °C to yield **18** as a hygroscopic off-white solid (1.86 g, 98%).[a]

Spectral data for this compound were consistent with those in literature.[9]

[a]The hygroscopic solid was not amenable to melting point determination in air.

δ_H (400 MHz, $CDCl_3$):	8.09-8.03 (m, 2H), 2.30-7.27 (m overlapped $CDCl_3$, 3H), 2.44-2.33 (m, 8H), 1.53-1.43 (m, 16H), 0.93 (t, 12H, *J* 6.9) ppm.
δ_C (100 MHz, $CDCl_3$):	171.7, 140.8, 129.5, 128.6, 127.2, 24.0 (d, *J* 15.2), 23.9 (d, *J* 4.9), 18.8 (d, *J* 47.3), 13.5 (d, *J* 0.6) ppm.
HRMS (*m/z* – ESI^+):	Found: 259.2551 (M^+) $C_{16}H_{36}P^+$ Requires: 259.2549.

Tetrabutylphosphonium 4-methoxybenzoate (19)

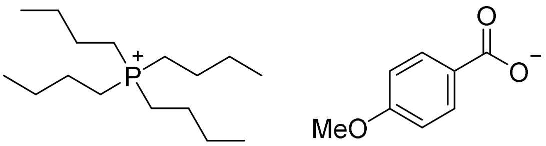

A 50 mL round-bottomed flask was charged with 4-methoxybenzoic acid (0.38 g, 2.5 mmol) and MeOH (25 mL). Tetrabutylphosphonium hydroxide in H_2O (40 wt.%, 1.75 mL, 2.5 mmol) was added dropwise to the solution and the resulting mixture was stirred for 16 h. The solvent was removed under reduced pressure and the product was dried under vacuum for 8 h at 60 °C to yield **19** as a hygroscopic off-white solid (1.01 g, 99%).[a]

[a]The hygroscopic solid was not amenable to melting point determination in air.

δ_H (400 MHz, DMSO-d_6):	7.72 (d, 2H, *J* 8.7), 6.73 (m, 2H, *J* 8.7), 3.72 (s, 3H), 2.23-2.12 (m, 8H), 1.52-1.34 (m, 16H), 0.91 (t, *J* 7.1) ppm.
δ_C (100 MHz, DMSO-d_6):	167.7, 159.1, 135.1, 130.3, 111.8, 23.3 (d, *J* 15.6), 22.6 (d, *J* 4.4), 17.3 (d, *J* 47.6), 13.2 ppm.
δ_P (162 MHz, DMSO-d_6):	33.8 ppm.
HRMS (*m/z* – ESI^+):	Found: 259.2553 (M^+) $C_{16}H_{36}P^+$ Requires: 259.2549.
ν_{max} (neat)/cm^{-1}:	2957, 2930, 2872, 1599, 1563, 1500, 1465, 1347, 1239, 1091, 1027, 853, 829, 786, 695.

Tetrabutylphosphonium 4-trifluoromethylbenzoate (20)

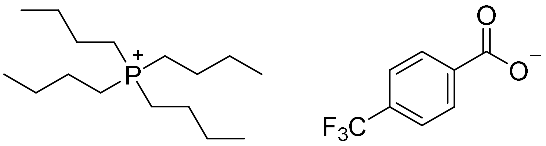

A 50 mL round-bottomed flask was charged with 4-trifluoromethylbenzoic acid (0.48 g, 2.5 mmol) and MeOH (25 mL). Tetrabutylphosphonium hydroxide in H_2O (40 wt.%, 1.75 mL, 2.5 mmol) was added dropwise to the solution and the resulting mixture was stirred for 16 h. The solvent was removed under reduced pressure and the product was dried under vacuum for 8 h at 60 °C to yield **20** as a hygroscopic off-white solid (1.05 g, 94%).[a]

[a]The hygroscopic solid was not amenable to melting point determination in air.

$\delta_{H\{C\}}$ (600 MHz, DMSO-*d*$_6$):	7.96 (d, 2H, *J* 7.8), 7.92 (d, 2H, *J* 7.8), 2.21-2.14 (m, 8H), 1.50-1.35 (m, 16H), 0.91 (m, 8H) ppm.
δ_C (150 MHz, DMSO-*d*$_6$):	166.5, 46.2, 129.4, 128.1 (q, *J* 31.1), 12.7 (q, *J* 271.8), 123.9 (q, *J* 3.4), 23.4 (d, *J* 5.6), 22.7 (d, *J* 4.5), 17.3 (d, *J* 47.7), 13.3 ppm.
δ_P (162 MHz, DMSO-*d*$_6$):	33.8 ppm.
δ_F (377 MHz, DMSO-*d*$_6$):	60.5 ppm.
HRMS (*m/z* – ESI$^+$):	Found: 259.2551 (M^+) $C_{16}H_{36}P^+$ Requires: 259.2549.
ν_{max} (neat)/cm^{-1}:	3466, 3265, 2961, 2926, 2871, 1610, 1570, 1466, 1371, 1320, 1140, 1092, 1061, 1016, 907, 871, 835, 788, 706.

Tetrabutylphosphonium cinnamate (21)

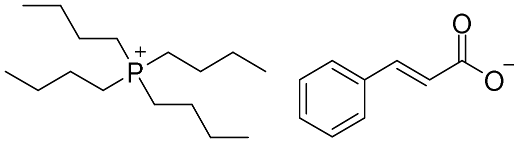

A 50 mL round-bottomed flask was charged with (*E*)-cinnamic acid (0.37 g, 2.5 mmol) and MeOH (25 mL). Tetrabutylphosphonium hydroxide in H_2O (40 wt.%, 1.75 mL, 2.5 mmol) was added dropwise to the solution and the resulting mixture was stirred for 16 h. The solvent was removed under reduced pressure and the product was dried under vacuum for 8 h at 60 °C to yield **21** as a hygroscopic pale yellow solid (1.01 g, 99%).[a]

[a]The hygroscopic solid was not amenable to melting point determination in air.

δ_H (400 MHz, DMSO-d_6):	7.45-7.40 (m, 2H), 7.34-7.28 (m, 2H), 7.25-7.19 (m, 1H), 6.97 (d, 1H, *J* 15.9), 6.32 (d, 1H, *J* 15.9), 2.26-2.11 (m, 8H), 1.53-1.34 (m, 16H), 0.92 (t, 12H, *J* 7.1) ppm.
δ_C (100 MHz, DMSO-d_6):	168.6, 137.2, 133.8, 132.1, 128.5, 127.4, 126.6, 23.4 (d, *J* 15.6), 22.6 (d, *J* 4.3), 17.3 (d, *J* 47.5), 13.3 ppm.
δ_P (162 MHz, DMSO-d_6):	33.8 ppm.
HRMS (*m/z* – ESI$^+$):	Found: 259.2552 (M^+) $C_{16}H_{36}P^+$ Requires: 259.2549.
ν_{max} (neat)/cm^{-1}:	3026, 2955, 2930, 2909, 2869, 1636, 1572, 1465, 1444, 1353, 1234, 1096, 981, 777, 714.

Tetrabutylphosphonium 2-naphthoate (22)

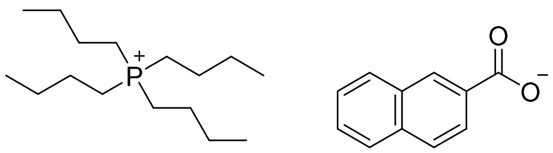

A 50 mL round-bottomed flask was charged with 2-naphthoic acid (0.43 g, 2.5 mmol) and MeOH (25 mL). Tetrabutylphosphonium hydroxide in H_2O (40 wt.%, 1.75 mL, 2.5 mmol) was added dropwise to the solution and the resulting mixture was stirred for 16 h. The solvent was removed under reduced pressure and the product was dried under vacuum for 8 h at 60 °C to yield **22** as a hygroscopic white solid (1.01 g, 99%).[a]

[a]The hygroscopic solid was not amenable to melting point determination in air.

δ_H (400 MHz, DMSO-d_6):	8.30 (s, 1H), 7.99 (d, 1H, *J* 8.4), 7.92-7.80 (m, 2H), 7.71 (d, 1H, *J* 8.4), 7.47-7.39 (m, 2H), 2.25-2.10 (m, 8H), 1.53-1.32 (m, 16H), 0.90 (t, 12H, *J* 7.0) ppm.
δ_C (100 MHz, DMSO-d_6):	167.8, 139.9, 133.1, 132.6, 128.4, 127.7, 127.7, 127.2, 125.8, 125.4, 125.2, 23.3 (d, *J* 15.6), 22.6 (d, *J* 4.4), 17.3 (d, *J* 47.6), 13.3 ppm.
δ_P (162 MHz, DMSO-d_6):	33.8 ppm.
HRMS (*m/z* – ESI$^+$):	Found: 259.2547 (M^+) $C_{16}H_{36}P^+$ Requires: 259.2549.
ν_{max} (neat)/cm^{-1}:	3056, 2958, 2930, 2902, 2871, 1628, 1600, 1590, 1565, 1462, 1416, 1337, 1097, 909, 872, 798, 773.

Tetrabutylphosphonium salicylate (23)

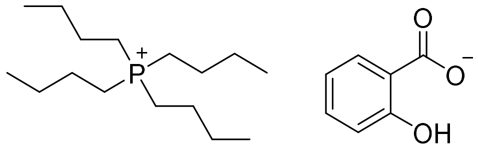

A 50 mL round-bottomed flask was charged with salicylic acid (0.35 g, 2.5 mmol) and MeOH (25 mL). Tetrabutylphosphonium hydroxide in H_2O (40 wt.%, 1.75 mL, 2.5 mmol) was added dropwise to the solution and the resulting mixture was stirred for 16 h. The solvent was removed under reduced pressure and the product was dried under vacuum for 8 h at 60 °C to yield **23** as an hygroscopic white solid (0.99 g, 99%).[a]

Spectral data for this compound were consistent with those in literature.[10]

[a]The hygroscopic solid was not amenable to melting point determination in air.

δ_H (400 MHz, DMSO-d_6):	7.63 (d, 1H, *J* 7.6), 7.12-7.06 (m, 1H), 6.60-6.50 (m, 2H), 2.25-2.11 (m, 8H), 1.53-1.33 (m, 16H), 0.91 (t, 12H, *J* 7.0) ppm.
δ_C (100 MHz, DMSO-d_6):	171.1, 163.2, 131.0, 129.8, 120.8, 115.7, 115.5, 23.4 (d, *J* 15.8), 22.6 (d, *J* 4.5), 17.3 (d, *J* 47.7), 13.3 ppm.
HRMS (*m/z* – ESI$^+$):	Found: 259.2548 (M^+) $C_{16}H_{36}P^+$ Requires: 259.2549.

Tetrabutylphosphonium difluoroacetate (24)

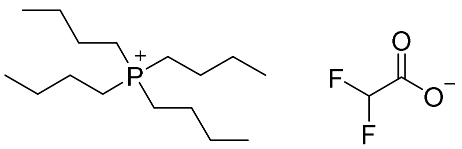

A 100 mL round-bottomed flask was charged with MeOH (50 mL). Tetrabutylphosphonium hydroxide in H_2O (40 wt.%, 3.50 mL, 5.0 mmol) was added dropwise to the solution and the resulting mixture was stirred for 5 min. Difluoroacetic acid (0.32 mL, 5.0 mmol) was added dropwise and the resulting solution was stirred for 16 h. The solvent was removed under reduced pressure and the product was dried under vacuum for 8 h at 60 °C to yield **24** as a colourless gum (1.77 g, 99%).

δ_H (400 MHz, DMSO-d_6):	5.47 (t, 1H, *J* 56.8), 2.25-2.10 (m, 8H), 1.52-1.32 (m, 16H), 0.90 (t, 12H, *J* 7.0) ppm.
δ_C (100 MHz, DMSO-d_6):	164.2, 110.6 (t, *J* 251.4), 23.4 (d, *J* 15.6), 22.6 (d, *J* 4.5), 17.3 (d, *J* 47.7), 13.3 ppm.
δ_P (162 MHz, DMSO-d_6):	33.8 ppm.
δ_F (376 MHz, DMSO-d_6):	-120.9 (d, *J* 56.9) ppm.
HRMS (*m/z* – ESI$^+$):	Found: 259.2549 (M^+) $C_{16}H_{36}P^+$ Requires: 259.2549.
v_{max} (neat)/cm^{-1}:	2960, 2933, 2874, 1647, 1466, 1410, 1299, 1100, 1032, 797.

Tetrabutylphosphonium pivalate (25)

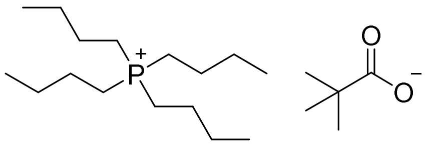

A 50 mL round-bottomed flask was charged with pivalic acid (0.26 g, 2.5 mmol) and MeOH (25 mL). Tetrabutylphosphonium hydroxide in H_2O (40 wt.%, 1.75 mL, 2.5 mmol) was added dropwise to the solution and the resulting mixture was stirred for 16 h. The solvent was removed under reduced pressure and the product was dried under vacuum for 8 h at 60 °C to yield **25** as an off-white gum (0.86 g, 96%).[a]

δ_H (400 MHz, DMSO-d_6):	2.26-2.13 (m, 8H), 1.54-1.34 (m, 16H), 0.95-0.88 (m, 21H) ppm.
δ_C (100 MHz, DMSO-d_6):	179.8, 39.0, 29.7, 23.8 (d, *J* 15.6), 23.1 (d, *J* 13.7), 17.8 (d, *J* 47.6), 13.7 ppm.
δ_P (162 MHz, DMSO-d_6):	33.8 ppm.
HRMS (*m/z* – ESI$^+$):	Found: 259.2551 (M^+) $C_{16}H_{36}P^+$ Requires: 259.2549.
ν_{max} (neat)/cm^{-1}:	2960, 2935, 2873, 1567, 1476, 1466, 1389, 1336, 1219, 1097, 971, 866, 786, 724.

Tetrabutylphosphonium stearate (26)

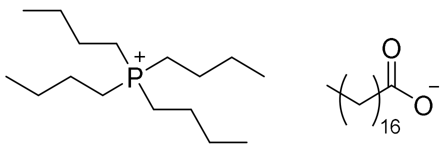

A 50 mL round-bottomed flask was charged with stearic acid (0.71 g, 2.5 mmol) and MeOH (25 mL). Tetrabutylphosphonium hydroxide in H_2O (40 wt.%, 1.75 mL, 2.5 mmol) was added dropwise to the solution and the resulting mixture was stirred for 16 h. The solvent was removed under reduced pressure and the product was dried under vacuum for 8 h at 60 °C to yield **26** as a colourless oil (1.35 g, 99%).

δ_H (400 MHz, $CDCl_3$): 2.52-2.40 (m, 8H), 2.18-2.12 (m, 2H), 1.66-1.43 (m, 18H), 1.34-1.18 (m, 28H), 0.96 (t, 16H, *J* 7.0), 0.87 (t, 3H, *J* 6.8) ppm.

δ_C (100 MHz, $CDCl_3$): 179.7, 39.7, 32.0, 30.3, 29.9-29.7 (10C coalesced resonances), 29.5, 27.5, 24.2 (d, *J* 15.4), 24.0 (d, *J* 4.8), 22.8, 18.8 (d, *J* 47.3), 14.2, 13.6 ppm.

δ_P (162 MHz, $CDCl_3$): 33.1 ppm.

HRMS (*m/z* – ESI^+): Found: 259.2560 (M^+) $C_{16}H_{36}P^+$ Requires: 259.2549.

ν_{max} (neat)/cm^{-1}: 2957, 2923, 2853, 1575, 1463, 1378, 1306, 1236, 1097, 914, 812, 721.

Tetrabutylphosphonium phenylacetate (27)

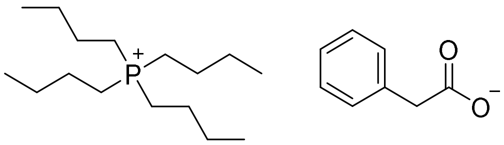

A 50 mL round-bottomed flask was charged with phenylacetic acid (0.34 g, 2.5 mmol) and MeOH (25 mL). Tetrabutylphosphonium hydroxide in H_2O (40 wt.%, 1.75 mL, 2.5 mmol) was added dropwise to the solution and the resulting mixture was stirred for 16 h. The solvent was removed under reduced pressure and the product was dried under vacuum for 8 h at 60 °C to yield **27** as a hygroscopic white solid (1.35 g, 99%).

δ_H (400 MHz, DMSO-d_6):	7.16 (app d, 4H), 7.09-7.02 (m, 1H), 3.09 (s, 2H), 2.24-2.12 (m, 8H), 1.53-1.34 (m, 16H), 0.92 (t, 12H, *J* 7.1) ppm.
δ_C (100 MHz, DMSO-d_6):	171.8, 141.0, 129.2, 127.3, 124.3, 47.3, 23.4 (d, *J* 15.6), 22.6 (d, *J* 4.4), 17.3 (d, *J* 47.6), 13.3 ppm.
δ_P (162 MHz, DMSO-d_6):	33.8 ppm.
HRMS (*m/z* – ESI$^+$):	Found: 259.2547 (M^+) $C_{16}H_{36}P^+$ Requires: 259.2549.
ν_{max} (neat)/cm^{-1}:	2958, 2931, 2872, 1591, 1465, 1340, 1243, 1196, 1096, 911, 724, 697.

Tetrabutylphosphonium hydrocinnamate (28)

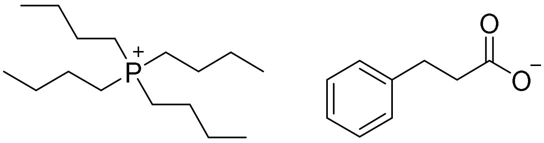

A 50 mL round-bottomed flask was charged with hydrocinnamic acid (0.38 g, 2.5 mmol) and MeOH (25 mL). Tetrabutylphosphonium hydroxide in H_2O (40 wt.%, 1.75 mL, 2.5 mmol) was added dropwise to the solution and the resulting mixture was stirred for 16 h. The solvent was removed under reduced pressure and the product was dried under vacuum for 8 h at 60 °C to yield **28** as an off-white gum (1.35 g, 98 %).

$\delta_{H\{C\}}$ (400 MHz, DMSO-*d₆*):	7.23-7.13 (m, 4H), 7.12-7.06 (m, 1H), 2.72-2.66 (m, 2H), 2.26-2.15 (m, 8H), 2.05-1.99 (m, 2H), 1.52-1.33 (m, 16H), 0.91 (t, 12H, *J* 7.1) ppm.
δ_C (100 MHz, DMSO-d_6):	173.6, 144.1, 128.1, 127.9, 124.9, 41.2, 33.2, 23.4 13.3 (d, *J* 15.7), 22.7 (d, *J* 4.4), 17.3 (d, *J* 47.6) ppm.
δ_P (162 MHz, DMSO-d_6):	33.8 ppm.
HRMS (*m/z* – ESI$^+$):	Found: 259.2553 (M^+) $C_{16}H_{36}P^+$ Requires: 259.2549.
ν_{max} (neat)/cm^{-1}:	3024, 2956, 2930, 2871, 1577, 1452, 1360, 1097, 998, 918, 754, 703.

Tetramethylphosphonium hydrocinnamate (29)

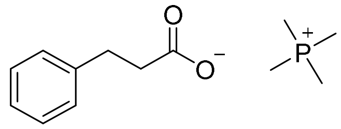

To a 50 mL round-bottomed flask, tetramethylphosphonium bromide (0.28 g, 1.6 mmol) and MeOH (20 mL) were added. Amberlite-OH (bed volume ≈ 5 mL, 6 meq of total exchange capacity)[a] was added to the solution and the flask was shaken for 16 h on an orbital shaker. The resulting solution was passed through a plug of Amberlite-OH (bed volume ≈ 5 mL, 6 meq of total exchange capacity). Hydrocinnamic acid (0.24 g, 1.6 mmol) was added, and the resulting solution was stirred for 16 h. The solvent was removed under reduced pressure and the product was dried under vacuum for 8 h at 60 °C to yield **29** as a colourless gum (0.38 g, 98%).

[a]Bed volume (mL) is measured in a burette or graduated cylinder as packed resin beads soaked in methanol.

δ_H (400 MHz, DMSO-d_6):	7.24-7.13 (m, 4H), 7.09 (t, 1H, *J* 7.0), 2.72-2.66 (m, 2H), 2.07-2.00 (m, 2H), 1.84 (d, 12H, *J* 15.3) ppm.
δ_C (100 MHz, DMSO-d_6):	173.9, 144.0, 28.1, 127.9, 125.0, 41.1, 33.1, 8.8 (d, *J* 55.3) ppm.
δ_P (162 MHz, DMSO-d_6):	25.5 ppm.
HRMS (*m/z* – ESI$^+$):	Found: 91.0671 (M^+) $C_4H_{12}P^+$ Requires: 91.0668.
ν_{max} (neat)/cm^{-1}:	3293, 2990, 2915, 1564, 1495, 1427, 1383, 1302, 982, 753, 776, 701.

Tetraoctylphosphonium hydrocinnamate (30)

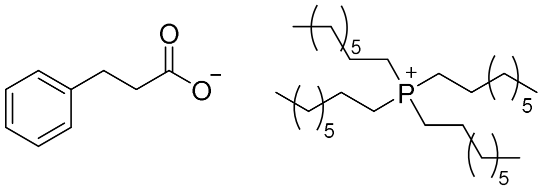

To a 50 mL round-bottomed flask, tetraoctylphosphonium bromide (0.56 g, 1.0 mmol) and MeOH (10 mL) were added. Amberlite-OH (bed volume ≈ 2.5 mL, 3 meq of total exchange capacity)[a] was added to the solution and the flask was shaken for 16 h on an orbital shaker. The resulting solution was passed through a plug of Amberlite-OH (bed volume ≈ 2.5 mL, 3 meq of total exchange capacity). Hydrocinnamic acid (0.15 g, 1.0 mmol) was added, and the resulting solution was stirred for 16 h. The solvent was removed under reduced pressure and the product was dried under vacuum for 8 h at 60 °C to yield **30** as a colourless oil (0.58 g, 87%).

[a]Bed volume (mL) is measured in a burette or graduated cylinder as packed resin beads soaked in methanol.

δ_H (400 MHz, DMSO-*d*$_6$):	7.24-7.13 (m, 4H), 7.09 (t, 1H, *J* 7.0), 2.72-2.65 (m, 2H), 2.25-2.12 (m, 8H), 2.06-2.00 (m, 2H), 1.53-1.18 (m, 48H), 0.86 (t, 12H, *J* 6.8) ppm.
δ_C (100 MHz, DMSO-*d*$_6$):	173.6, 144.1, 128.1, 127.9, 124.9, 41.1, 33.2, 31.2, 30.0 (d, *J* 15.1), 28.4, 28.1, 22.1, 20.5 (d, *J* 4.4), 17.4 (d, *J* 47.3), 13.9 ppm.
δ_P (162 MHz, DMSO-*d*$_6$):	33.8 ppm.
HRMS (*m/z* – ESI$^+$):	Found: 483.5051 (M$^+$) $C_{32}H_{68}P^+$ Requires: 483.5053.
ν_{max} (neat)/cm^{-1}:	2924, 2855, 1578, 1459, 1375, 1115, 1031, 721, 698.

Trihexyltetradecylphosphonium hydrocinnamate (31)

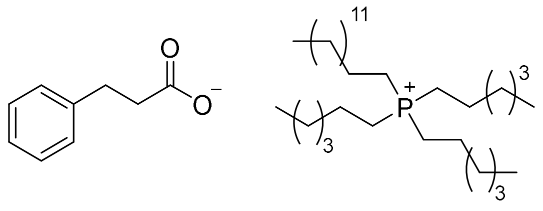

To a 50 mL round-bottomed flask, trihexyltetradecylphosphonium bromide (0.56 g, 1.0 mmol) and MeOH (10 mL) were added. Amberlite-OH (bed volume ≈ 2.5 mL, 3 meq of total exchange capacity)[a] was added to the solution and the flask was shaken for 16 h on an orbital shaker. The resulting solution was passed through a plug of Amberlite-OH (bed volume ≈ 2.5 mL, 3 meq of total exchange capacity). Hydrocinnamic acid (0.15 g, 1.0 mmol) was added, and the resulting solution was stirred for 16 h. The solvent was removed under reduced pressure and the product was dried under vacuum for 8 h at 60 °C to yield **31** as a colourless oil (0.58 g, 87%).

[a]Bed volume (mL) is measured in a burette or graduated cylinder as packed resin beads soaked in methanol.

δ_H (400 MHz, DMSO-d_6): 7.24-7.13 (m, 4H), 7.09 (t, 1H, *J* 7.0), 2.78-2.65 (m, 2H), 2.25-2.10 (m, 8H), 2.09-2.00 (m, 2H), 1.54-1.16 (m, 48H), 0.93-0.81 (m, 12H) ppm.

δ_C (100 MHz, DMSO-d_6): 173.7, 143.9, 128.1, 127.9, 125.0, 40.8, 33.0, 31.3, 30.4, 29.9 (d, *J* 15.2), 29.7 (d, *J* 15.2), 29.0, 29.0, 29.0, 29.0, 28.9, 28.7, 28.6, 28.1, 22.1, 21.8, 20.5 (d, *J* 4.6), 20.4 (d, *J* 4.4), 17.5 (d, *J* 47.5), 17.4 (d, *J* 47.2), 13.9, 13.8 ppm.

δ_P (162 MHz, DMSO-d_6): 33.7 ppm.

HRMS (*m/z* – ESI$^+$): Found: 483.5054 (M^+) $C_{32}H_{68}P^+$ Requires: 483.5053.

ν_{max} (neat)/cm^{-1}: 2924, 2854, 1577, 1459, 1376, 1111, 993, 720, 698.

Methyltrioctylphosphonium iodide (S1)

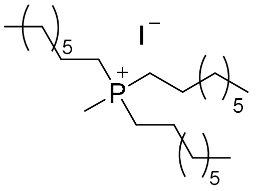

An oven dried 25 mL round bottomed flask was charged with anhydrous acetonitrile (5 mL) and tributylphosphine (2.23 g, 5 mmol). The emulsion was kept under an argon atmosphere, stirred vigorously for 5 min, and cooled to 0 °C. Iodomethane (0.34 mL, 5.5 mmol) was added dropwise to the emulsion and the resulting mixture was stirred at room temperature for 1 h. The reaction mixture was concentrated under reduced pressure and the resulting oil is washed with Et_2O (5 x 10 mL) maintaining vigorous stirring for 2 min, Et_2O is then decanted off the mixture. The resulting oil was dried under vacuum for 8 h at 60 °C to yield **S1** as a colourless oil (2.31 g, 90%).

Spectral data for this compound were consistent with those in literature.[11]

δ_H (400 MHz, $CDCl_3$):	2.48-2.36 (m, 6H), 2.09 (d, 3H, *J* 13.3), 1.60-1.40 (m, 12H), 1.36-1.17 (m, 24H), 0.85 (t, 9H, *J* 6.6) ppm.
δ_P (162 MHz, $CDCl_3$):	31.2 ppm.
HRMS (*m/z* – ESI$^+$):	Found: 385.3960 (M^+) $C_{25}H_{54}P^+$ Requires: 385.3958.
ν_{max} (neat)/cm^{-1}:	2955, 2923, 2855, 1460, 1378, 1306, 1098, 1033, 906, 770, 719.

Methyltrioctylphosphonium hydrocinnamate (32)

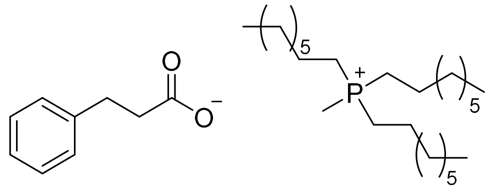

To a 50 mL round-bottomed flask, **S1** (0.23 g, 0.5 mmol) and MeOH (10 mL) were added. Amberlite-OH (bed volume ≈ 2.5 mL, 3 meq of total exchange capacity)[a] was added to the solution and the flask was shaken for 16 h on an orbital shaker. The resulting solution was passed through a plug of Amberlite-OH (bed volume ≈ 2.5 mL, 3 meq of total exchange capacity). Hydrocinnamic acid (0.08 g, 0.5 mmol) was added, and the resulting solution was stirred for 16 h. The solvent was removed under reduced pressure and the product was dried under vacuum for 8 h at 60 °C to yield **32** as a colourless oil (0.21 g, 77%).

[a]Bed volume (mL) is measured in a burette or graduated cylinder as packed resin beads soaked in methanol.

δ_H (400 MHz, DMSO-*d₆*):	7.24-7.13 (m, 4H), 7.12-7.06 (m, 1H), 2.71-2.65 (m, 2H), 2.20-2.09 (m, 6H), 2.05-1.99 (m, 2H), 1.77 (d, 3H, *J* 14.0), 1.51-1.22 (m, 36H), 0.87 (t, 9H, 6.8) ppm.
δ_C (100 MHz, DMSO-d_6):	173.7, 144.1, 128.2, 127.9, 125.0, 41.2, 33.2, 31.2, 30.0 (d, *J* 15.4), 28.4, 28.2, 22.1, 20.5 (d, *J* 4.7), 19.1 (d, *J* 49.0), 14.0, 3.2 (d, *J* 51.4) ppm.
δ_P (162 MHz, DMSO-d_6):	32.3 ppm.
HRMS (*m/z* – ESI$^+$):	Found: 385.3960 (M$^+$) $C_{25}H_{54}P^+$ Requires: 385.3958.
ν_{max} (neat)/cm^{-1}:	2924, 2855, 1577, 1459, 1375, 1311, 1031, 925, 748, 698.

Tributyl-3-phenylpropylophosphonium bromide (S2)

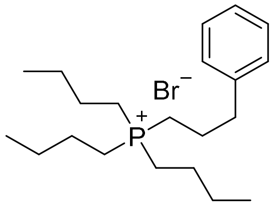

An oven dried 25 mL round bottomed flask was charged with anhydrous acetonitrile (5 mL) and tributylphosphine (1.01 g, 5 mmol). The emulsion was kept under an argon atmosphere, stirred vigorously for 5 min, and cooled to 0 °C. 1-Bromo-3-phenylpropane (0.68 mL, 4.5 mmol) was added dropwise to the emulsion and the resulting mixture was stirred at room temperature for 1 h. The reaction mixture was concentrated under reduced pressure and the resulting oil is washed with Et_2O (5 x 10 mL) maintaining vigorous stirring for 2 min, Et_2O is then decanted out of the mixture. The residue is purified by flash chromatography (CH_2Cl_2:MeOH, 9:1) and dried under vacuum for 8 h at 60 °C to yield **S2** as a hygroscopic white solid (0.41 g, 20%).

δ_H (400 MHz, $CDCl_3$):	7.34-7.27 (m, 2H), 7.25-7.16 (m, 3H), 2.81 (t, 2H, *J* 7.1), 2.52-2.34 (m, 8H), 1.95-1.83 (m, 2H), 1.53-1.36 (m, 12H), 0.93 (t, 9H, *J* 7.0) ppm.
δ_C (100 MHz, $CDCl_3$):	139.8, 128.9, 128.7, 126.8, 36.3 (d, *J* 15.4), 24.0 (d, *J* 15.3), 23.9, 23.8 (d, *J* 4.8), 19.2 (d, *J* 47.3), 18.5 (d, *J* 47.8), 13.6 ppm.
δ_P (162 MHz, $CDCl_3$):	33.1 ppm.
HRMS (*m/z* – ESI^+):	Found: 321.2709 (M^+) $C_{21}H_{38}P^+$ Requires: 321.2706.
ν_{max} (neat)/cm^{-1}:	3027, 2960, 2932, 2872, 2164, 1979, 1603, 1493, 1453, 1414, 1380, 1231, 1093, 912, 828, 751, 719, 701.

Tributyl-3-phenylpropylophosphonium hydrocinnamate (33)

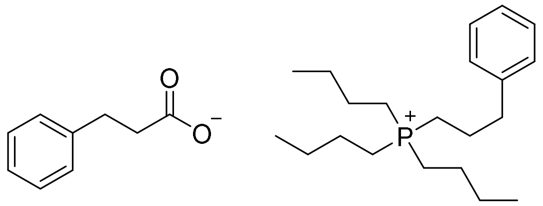

To a 50 mL round-bottomed flask, **S2** (0.5 g, 0.4 mmol) and MeOH (10 mL) were added. Amberlite-OH (bed volume ≈ 2.5 mL, 3 meq of total exchange capacity)[a] was added to the solution and the flask was shaken for 16 h on an orbital shaker. The resulting solution was passed through a plug of Amberlite-OH (bed volume ≈ 2.5 mL, 3 meq of total exchange capacity). Hydrocinnamic acid (0.06 g, 0.4 mmol) was added, and the resulting solution was stirred for 16 h. The solvent was removed under reduced pressure and the product was dried under vacuum for 8 h at 60 °C to yield **33** as a pale-yellow oil (0.17 g, 97%).

[a]Bed volume (mL) is measured in a burette or graduate cylinder as packed resin beads soaked in methanol.

δ_H (400 MHz, DMSO-*d₆*):	7.35-7.28 (m, 2H), 7.28-7.13 (m, 7H), 7.09 (t, 1H, *J* 7.0), 2.75-2.64 (m, 4H), 2.27-2.10 (m, 8H), 2.06-2.00 (m, 2H), 1.87-1.73 (m, 2H), 1.48-1.31 (m, 12H), 0.90 (t, 9H, *J* 7.0) ppm.
δ_C (100 MHz, DMSO-d_6):	173.6, 144.0, 140.5, 128.4, 128.4, 128.1, 127.9, 126.2, 124.9, 41.0, 35.8 (d, *J* 15.7), 33.1, 23.3 (d, *J* 15.7), 22.6 (d, *J* 4.4), 22.5 (d, *J* 3.6), 17.2 (d, *J* 47.7), 17.1 (d, *J* 47.9), 13.2 ppm.
δ_P (162 MHz, DMSO-d_6):	34.0 ppm.
HRMS (*m/z* – ESI$^+$):	Found: 321.2707 (M$^+$) $C_{21}H_{38}P^+$ Requires: 321.2706.
ν_{max} (neat)/cm^{-1}:	3026, 2958, 2931, 2872, 1574, 1495, 1454, 1375, 1098, 1078, 909, 749, 700.

Benzyltributylphosphonium bromide (S3)

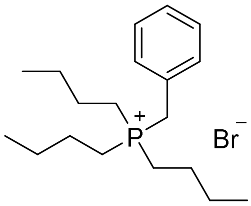

An oven dried 50 mL round bottomed flask was charged with anhydrous acetonitrile (10 mL) and tributylphosphine (2.02 g, 10 mmol). The emulsion was kept under an argon atmosphere, stirred vigorously for 5 min, and cooled to 0 °C. Benzyl bromide (1.54 g, 9 mmol) was added dropwise to the emulsion and the resulting mixture was stirred at room temperature for 16 h. The crude mixture was evaporated under reduced pression. Diethyl ether (30 mL) was added to initiate product crystallisation. The product was filtered and the resulting solid was washed with diethyl ether (3 x 20 mL) to yield **S3** as a white solid (3.28 g, 98 %). M.p. 143.0-144.0 °C. (Lit.,[11] 143 °C)

Spectral data for this compound were consistent with those in literature.[12]

δ_H (400 MHz, DMSO-d_6):	7.46-7.33 (m, 5H), 3.83 (d, *J* 15.5, 2H), 2.21-2.10 (m, 6H), 1.48-1.31 (m, 12H), 0.88 (t, *J* 7.0, 9H) ppm.
δ_C (100 MHz, DMSO-d_6):	130.0 (d, *J* 4.8), 129.3, 129.2 (d, *J* 3.1), 128.0 (d, *J* 3.4), 25.4 (d, *J* 44.5), 23.3 (d, *J* 15.7), 22.5 (d, *J* 4.5), 17.4 (d, *J* 47.1), 13.2 ppm.
HRMS (m/z – ESI$^+$):	Found: 293.2399 (M^+) $C_{19}H_{34}P^+$ Requires: 293.2393.
ν_{max} (neat)/cm^{-1}:	2959, 2931, 2895, 2867, 2797, 2165, 1979, 1602, 1495, 1457, 1410, 1379, 1229, 1097, 1080, 968, 919, 821, 701.

Benzyltributylphosphonium hydrocinnamate (34)

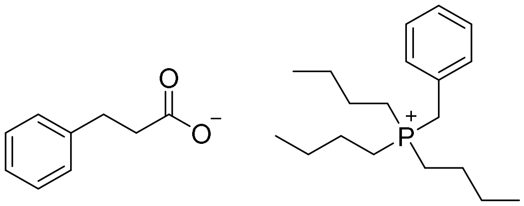

To a 50 mL round-bottomed flask, **S3** (0.38 g, 1 mmol) and MeOH (10 mL) were added. Amberlite-OH (bed volume ≈ 2.5 mL, 3 meq of total exchange capacity)[a] was added to the solution and the flask was shaken for 16 h on an orbital shaker. The resulting solution was passed through a plug of Amberlite-OH (bed volume ≈ 2.5 mL, 3 meq of total exchange capacity). Hydrocinnamic acid (0.15 g, 1.0 mmol) was added, and the resulting solution was stirred for 16 h. The solvent was removed under reduced pressure and the product was dried under vacuum for 8 h at 60 °C to yield **34** as a colourless gum (0.43 g, 97%).

[a]Bed volume (mL) is measured in a burette or graduated cylinder as packed resin beads soaked in methanol.

δ_H (400 MHz, DMSO-*d*$_6$):	7.47-7.32 (m, 5H), 7.24-7.13 (m, 4H), 7.12-7.05 (m, 1H), 3.83 (d, 2H, *J* 15.5), 2.73-2.64 (m, 2H), 2.22-2.08 (m, 6H), 2.07-2.00 (m, 2H), 1.48-1.29 (m, 12H), 0.88 (t, 9H, *J* 6.9) ppm.
δ_C (100 MHz, DMSO-*d*$_6$):	173.7, 144.0, 130.0 (d, *J* 5.0), 129.4 (d, *J* 8.3), 129.1 (d, *J* 2.9), 128.1, 127.9, 127.9, 124.9, 41.0, 33.1, 25.4 (d, *J* 44.4), 23.3 (d, *J* 15.7), 22.5 (d, *J* 4.6), 17.3 (d, *J* 46.9), 13.2 ppm.
δ_P (162 MHz, DMSO-*d*$_6$):	32.5 ppm.
HRMS (*m/z* – ESI$^+$):	Found: 293.2391 (M$^+$) $C_{19}H_{34}P^+$ Requires: 293.2393.
ν_{max} (neat)/cm^{-1}:	3025, 2958, 2930, 2872, 1572, 1458, 1371, 1096, 917, 829, 747, 699.

Tributyl(naphthalen-1-ylmethyl)phosphonium bromide (S4)

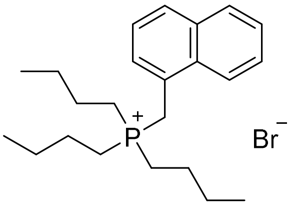

An oven dried 25 mL round bottomed flask was charged with anhydrous acetonitrile (3 mL) and tributylphosphine (1.01 g, 5 mmol). The emulsion was kept under an argon atmosphere, stirred vigorously for 5 min, and cooled to 0 °C. 1-(Bromomethyl)naphthalene (1.11 g, 4.5 mmol) was dissolved in anhydrous acetonitrile (2 mL), added dropwise to the emulsion and the resulting mixture was stirred at room temperature for 16 h. The crude mixture was evaporated under reduced pression. Diethyl ether (15 mL) was added to initiate product crystallisation. The product was filtered and the resulting solid was washed with diethyl ether (3 x 10 mL) to yield **S4** as a white solid (1.64 g, 86 %). M.p. 100.0-102.0 °C.

Spectral data for this compound were consistent with those in literature.[13]

δ_H (400 MHz, DMSO-d_6):	8.03-7.87 (m, 4H), 7.61-7.52 (m, 2H), 7.52-7.44 (m, 1H), 4.01 (d, 2H, *J* 15.5), 2.27-2.12 (m, 6H), 1.53-1.30 (m, 12H), 0.87 (t, 9H, *J* 7.2) ppm.
δ_C (100 MHz, DMSO-d_6):	132.9 (d, *J* 2.9), 132.2 (d, *J* 2.5), 128.9 (d, *J* 6.5), 128.8 (d, *J* 2.3), 127.7, 127.6 (d, *J* 3.9), 127.5, 126.9, 126.8, 126.5, 25.6 (d, *J* 44.2), 23.3 (d, *J* 15.7), 22.5 (d, *J* 4.5), 17.4 (d, *J* 46.8), 13.2 ppm.
δ_P (162 MHz, DMSO-d_6):	33.0 ppm.
HRMS (m/z – ESI$^+$):	Found: 343.2559 (M^+) $C_{23}H_{36}P^+$ Requires: 343.2549.
ν_{max} (neat)/cm^{-1}:	2957, 2929, 2871, 1465, 1094, 917, 861, 828, 744.

Tributyl(naphthalen-1-ylmethyl)phosphonium hydrocinnamate (35)

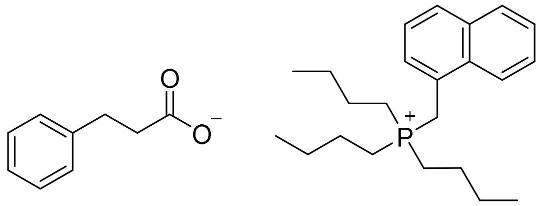

To a 50 mL round-bottomed flask, **S4** (0.42 g, 1 mmol) and MeOH (10 mL) were added. Amberlite-OH (bed volume ≈ 2.5 mL, 3 meq of total exchange capacity)[a] was added to the solution and the flask was shaken for 16 h on an orbital shaker. The resulting solution was passed through a plug of Amberlite-OH (bed volume ≈ 2.5 mL, 3 meq of total exchange capacity). Hydrocinnamic acid (0.15 g, 1.0 mmol) was added, and the resulting solution was stirred for 16 h. The solvent was removed under reduced pressure and the product was dried under vacuum for 8 h at 60 °C to yield **35** as a yellow gum (0.47 g, 96%).

[a]Bed volume (mL) is measured in a burette or graduated cylinder as packed resin beads soaked in methanol.

δ_H (400 MHz, DMSO-d_6): 8.00-7.87 (m, 4H), 7.61-7.52 (m, 2H), 7.49-7.43 (m, 1H), 7.23-7.13 (m, 4H), 7.12-7.06 (m, 1H), 3.99 (d, 2H, *J* 15.5), 2.73-2.65 (m, 2H), 2.25-2.12 (m, 6H), 2.08-2.00 (m, 2H), 1.51-1.31 (m, 12H), 0.87 (t, 9H, *J* 7.1) ppm.

δ_C (100 MHz, DMSO-d_6): 173.7, 144.0, 132.9 (d, *J* 3.2), 132.2 (d, *J* 2.5), 128.9 (d, *J* 6.7), 128.8 (d, *J* 2.5), 128.1, 127.9, 127.7-127.6 (m, 2C), 127.5 (d, *J* 1.3), 126.9 (d, *J* 8.9), 126.8 (d, *J* 1.0), 125.5 (d, *J* 1.7), 125.0, 40.9, 33.0, 25.6 (d, *J* 44.1), 23.3 (d, *J* 15.8), 22.5 (d, *J* 4.6), 17.3 (d, *J* 46.9), 13.2 (d, *J* 0.7) ppm.

δ_P (162 MHz, DMSO-d_6): 33.0 ppm.

HRMS (*m/z* – ESI^+): Found: 343.2550 (M^+) $C_{23}H_{36}P^+$ Requires: 343.2549.

ν_{max} (neat)/cm^{-1}: 2958, 2931, 2872, 1573, 1454, 1373, 1233, 1150, 1097, 1075, 958, 915, 829, 747, 699.

Tetramethylammonium hydrocinnamate (36)

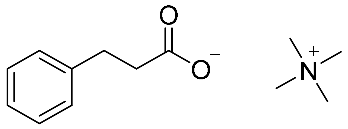

To a 100 mL round-bottomed flask, tetramethylammonium bromide (0.46 g, 5 mmol) and MeOH (50 mL) were added. Amberlite-OH (bed volume ≈ 12.5 mL, 15 meq of total exchange capacity)[a] was added to the solution and the flask was shaken for 16 h on an orbital shaker. The resulting solution was passed through a plug of Amberlite-OH (bed volume ≈ 12.5 mL, 15 meq of total exchange capacity). Hydrocinnamic acid (0.75 g, 5.0 mmol) was added, and the resulting solution was stirred for 16 h. The solvent was removed under reduced pressure and the product was dried under vacuum for 8 h at 60 °C to yield **36** as an off-white gum (1.06 g, 95%).

δ_H (400 MHz, D_2O):	7.42-7.24 (m, 5H), 3.16 (s, 12H), 2.94-2.87 (m, 2H), 2.54-2.46 (m, 2H) ppm.
δ_C (100 MHz, D_2O):	182.5, 142.0, 128.6, 128.3, 126.0, 55.1 (t, *J* 4.1),[b] 39.2, 31.9 ppm.
HRMS (*m/z* – ESI^+):	Found: 74.0962 (M^+) $C_4H_{12}N^+$ Requires: 74.0964.
ν_{max} (neat)/cm^{-1}:	3297, 3027, 2953, 1568, 1490, 1382, 1299, 1077, 1032, 951, 853, 752, 701.

[a]Bed volume (mL) is measured in a burette or graduated cylinder as packed resin beads soaked in methanol.

[b]The triplet originates from a spin-spin coupling between ^{14}N and ^{13}C.[14]

Tetrabutylammonium hydrocinnamate (37)

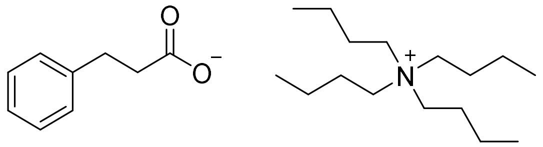

To a 50 mL round-bottomed flask, tetrabutylammonium chloride (0.42 g, 1 mmol) and MeOH (10 mL) were added. Amberlite-OH (bed volume ≈ 2.5 mL, 3 meq of total exchange capacity)[a] was added to the solution and the flask was shaken for 16 h on an orbital shaker. The resulting solution was passed through a plug of Amberlite-OH (bed volume ≈ 2.5 mL, 3 meq of total exchange capacity). Hydrocinnamic acid (0.15 g, 1.0 mmol) was added, and the resulting solution was stirred for 16 h. The solvent was removed under reduced pressure and the product was dried under vacuum for 8 h at 60 °C to yield **37** as a colourless gum (0.39 g, 99%).

[a]Bed volume (mL) is measured in a burette or graduated cylinder as packed resin beads soaked in methanol.

δ_H (400 MHz, DMSO-d_6):	7.24-7.13 (m, 4H), 7.12-7.06 (m, 1H), 3.21-3.12 (m, 8H), 2.78-2.64 (m, 2H), 2.05-1.98 (m, 2H), 1.63-1.49 (m, 8H), 1.37-1.24 (m, 8H), 0.93 (t, 12H, *J* 7.3) ppm.
δ_C (100 MHz, DMSO-d_6):	173.5, 114.1, 128.1, 127.9, 124.9, 57.5, 41.2, 33.2, 23.2, 19.2, 13.5 ppm.
HRMS (*m/z* – ESI$^+$):	Found: 242.2839 (M$^+$) $C_{16}H_{36}N^+$ Requires: 242.2842.
ν_{max} (neat)/cm^{-1}:	2959, 2873, 1582, 1493, 1463, 135, 1313, 1151, 1029, 897, 883, 760, 708.

Tetrahexylammonium hydrocinnamate (38)

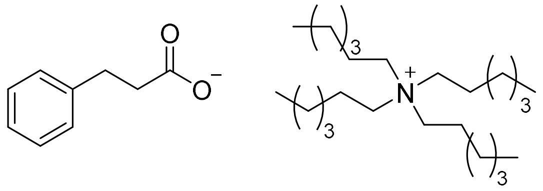

To a 50 mL round-bottomed flask, tetrahexylammonium bromide (0.44 g, 1 mmol) and MeOH (10 mL) were added. Amberlite-OH (bed volume ≈ 2.5 mL, 3 meq of total exchange capacity)[a] was added to the solution and the flask was shaken for 16 h on an orbital shaker. The resulting solution was passed through a plug of Amberlite-OH (bed volume ≈ 2.5 mL, 3 meq of total exchange capacity). Hydrocinnamic acid (0.15 g, 1.0 mmol) was added, and the resulting solution was stirred for 16 h. The solvent was removed under reduced pressure and the product was dried under vacuum for 8 h at 60 °C to yield **38** as a hygroscopic off-white gum (0.50 g, 99%).

[a]Bed volume (mL) is measured in a burette or graduated cylinder as packed resin beads soaked in methanol.

δ_H (400 MHz, DMSO-d_6):	7.24-7.13 (m, 4H), 7.12-7.06 (m, 1H), 3.22-3.10 (m, 8H), 2.71-2.64 (m, 2H), 2.05-1.98 (m, 2H), 1.63-1.51 (m, 8H), 1.35-1.22 (m, 24H), 0.88 (t, 12H, *J* 6.8) ppm.
δ_C (100 MHz, DMSO-d_6):	173.5, 144.1, 128.1, 127.9, 124.9, 57.6, 41.2, 33.2, 30.5, 25.4, 21.8, 20.9, 13.8 ppm.
HRMS (*m/z* – ESI$^+$):	Found: 354.4099 (M$^+$) $C_{24}H_{52}N^+$ Requires: 354.4094.
ν_{max} (neat)/cm^{-1}:	2955, 2927, 2859, 1580, 1491, 1466, 1372, 1147, 1050, 750, 728, 699.

Tetraoctylammonium hydrocinnamate (39)

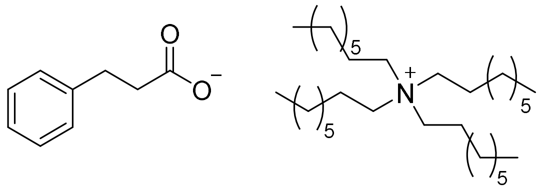

To a 50 mL round-bottomed flask, tetraoctylammonium bromide (0.55 g, 1 mmol) and MeOH (10 mL) were added. Amberlite-OH (bed volume ≈ 2.5 mL, 3 meq of total exchange capacity)[a] was added to the solution and the flask was shaken for 16 h on an orbital shaker. The resulting solution was passed through a plug of Amberlite-OH (bed volume ≈ 2.5 mL, 3 meq of total exchange capacity). Hydrocinnamic acid (0.15 g, 1.0 mmol) was added, and the resulting solution was stirred for 16 h. The solvent was removed under reduced pressure and the product was dried under vacuum for 8 h at 60 °C to yield **39** as a viscous transparent liquid (0.60 g, 95%).

[a]Bed volume (mL) is measured in a burette or graduated cylinder as packed resin beads soaked in methanol.

δ_H (400 MHz, DMSO-d_6):	7.24-7.13 (m, 4H), 7.12-7.06 (m, 1H), 3.22-3.09 (m, 8H), 2.62-2.64 (m, 2H), 2.05-1.98 (m, 2H), 1.64-1.48 (m, 8H), 1.38-1.17 (m, 40H), 0.87 (t, 12H, *J* 6.8) ppm.
δ_C (100 MHz, DMSO-d_6):	173.5, 144.1, 128.2, 127.9, 124.9, 57.5, 41.2, 33.2, 31.2, 28.4, 28.3, 25.7, 22.1, 20.9, 13.9 ppm.
HRMS (*m/z* – ESI$^+$):	Found: 466.5346 (M$^+$) $C_{32}H_{68}N^+$ Requires: 466.5346.
ν_{max} (neat)/cm^{-1}:	2924, 2855, 1578, 1490, 1467, 1376, 1075, 849, 723, 699.

Methyltrioctylammonium hydrocinnamate (40)

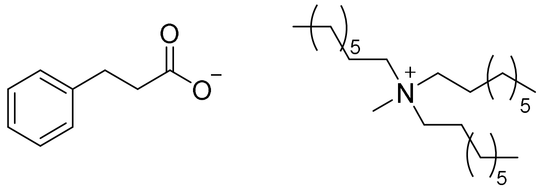

To a 50 mL round-bottomed flask, methyltrioctylammonium bromide (0.35 g, 1 mmol) and MeOH (10 mL) were added. Amberlite-OH (bed volume ≈ 2.5 mL, 3 meq of total exchange capacity)[a] was added to the solution and the flask was shaken for 16 h on an orbital shaker. The resulting solution was passed through a plug of Amberlite-OH (bed volume ≈ 2.5 mL, 3 meq of total exchange capacity). Hydrocinnamic acid (0.15 g, 1.0 mmol) was added, and the resulting solution was stirred for 16 h. The solvent was removed under reduced pressure and the product was dried under vacuum for 8 h at 60 °C to yield **40** as an off-white gum (0.40 g, 78%).

[a]Bed volume (mL) is measured in a burette or graduated cylinder as packed resin beads soaked in methanol.

δ_H (400 MHz, DMSO-*d*$_6$):	7.23-7.12 (m, 4H), 7.12-7.06 (m, 1H), 3.24-3.13 (m, 6H), 2.93 (s, 3H), 2.72-2.64 (m, 2H), 2.06-1.98 (m, 2H), 1.68-1.54 (m, 6H), 1.37-1.19 (m, 30H), 0.87 (t, 9H, *J* 6.8) ppm.
δ_C (100 MHz, DMSO-*d*$_6$):	173.5, 144.1, 128.1, 127.9, 124.9, 60.5, 47.5, 41.1, 33.2, 31.1, 28.4, 28.4, 25.7, 22.0, 21.3, 13.9 ppm.
HRMS (*m/z* – ESI$^+$):	Found: 368.4254 (M^+) $C_{25}H_{54}N^+$ Requires: 368.4251.
ν_{max} (neat)/cm^{-1}:	2924, 2855, 1578, 1463, 1375, 1075, 898, 723, 699.

***N*,*N*-dimethyl-*N*,*N*-diundecylammonium bromide (S5)**

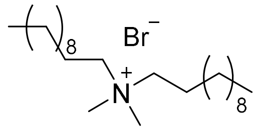

A 25 mL round bottomed flask was charged with anhydrous acetonitrile (2 mL), dimethylamine (2 mL, 4 mmol, 2.0 M solution in THF) and potassium carbonate (2.21 g, 16 mmol). 1-Bromoundecane (3.12 mL, 14 mmol) was diluted with anhydrous acetonitrile (2 mL) and added dropwise to the reaction mixture. The reaction vessel was fitted with a condenser and heated at reflux under nitrogen atmosphere (balloon). The crude reaction mixture was filtered, residual white solid was washed with acetonitrile (3 x 5 mL) and the filtrate was evaporated under reduced pressure. The resulting oil was purified by flash chromatography (CH_2Cl_2 to CH_2Cl_2/MeOH (9:1)) to yield **S5** as a white solid (1.14 g, 66 %). M.p. 159.5-161.0 °C.

Spectral data for this compound were consistent with those in literature.[13]

δH (400 MHz, $CDCl_3$):	3.57-3.46 (m, 4H), 3.41 (s, 6H), 1.74-1.63 (m overlapped H_2O, 4H), 1.43-1.20 (m, 32H), 0.87 (t, 6H, *J* 6.9) ppm
δC (100 MHz, $CDCl_3$):	64.0, 51.4, 32.0, 29.6, 29.6, 29.5, 29.4, 29.3, 26.4, 22.9, 22.8, 14.2 ppm
νmax (neat)/ cm^{-1}:	2951.8 (w), 2921.4 (s), 2853.3 (s), 1466.4 (m), 1455.8 (m), 889.7 (m), 720.8 (m).
HRMS (m/z – ESI^+):	Found: 354.4099 (M^+) $C_{24}H_{52}N^+$ Requires: 354.4094.
ν_{max} (neat)/cm^{-1}:	2921, 2853, 1467, 1409, 1378, 1053, 990, 962, 889, 721.

***N*,*N*-dimethyl-*N*,*N*-diundecylammonium hydrocinnamate (41)**

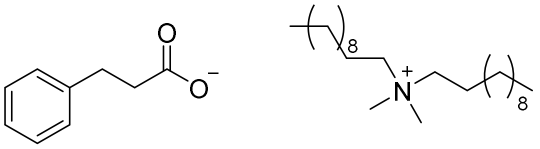

To a 50 mL round-bottomed flask, **S5** (0.22 g, 0.5 mmol) and MeOH (10 mL) were added. Amberlite-OH (bed volume ≈ 2.5 mL, 3 meq of total exchange capacity)[a] was added to the solution and the flask was shaken for 16 h on an orbital shaker. The resulting solution was passed through a plug of Amberlite-OH (bed volume ≈ 2.5 mL, 3 meq of total exchange capacity). Hydrocinnamic acid (0.08 g, 0.5 mmol) was added, and the resulting solution was stirred for 16 h. The solvent was removed under reduced pressure and the product was dried under vacuum for 8 h at 60 °C to yield **41** as a transparent viscous oil (0.25 g, 98%).

[a]Bed volume (mL) is measured in a burette or graduated cylinder as packed resin beads soaked in methanol.

δ_H (400 MHz, DMSO-d_6):	7.24-7.12 (m, 4H), 7.12-7.06 (m, 1H), 3.27-3.18 (m, 4H), 2.98 (s, 6H), 2.71-2.65 (m, 2H), 2.05-1.99 (m, 2H), 1.69-1.56 (m, 4H), 1.36-1.18 (m, 32H), 0.86 (t, 6H, *J* 6.8) ppm.
δ_C (100 MHz, DMSO-d_6):	173.5, 144.1, 128.1, 127.9, 124.9, 62.7, 49.9, 41.2, 33.2, 31.3, 28.9, 28.9, 28.7, 28.7, 28.4, 25.7, 22.1, 21.6, 13.9 ppm.
HRMS (*m/z* – ESI$^+$):	Found: 354.4097 (M^+) $C_{24}H_{52}N^+$ Requires: 354.4094.
ν_{max} (neat)/cm^{-1}:	3026, 2923, 2853, 1576, 1494, 1467, 1376, 1076, 922, 721, 698.

***N*-Methyl-*N*-octylpyrrolidinium bromide (S6)**

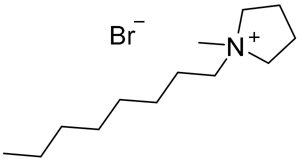

A 25 mL round bottomed flask was charged with anhydrous acetonitrile (6 mL) and *N*-methylpyrrolidine (1.04 mL, 10 mmol) and a stirring bar. 1-bromooctane (1.86 mL, 10.7 mmol) was added dropwise to the reaction mixture and the reaction heated for 16 h at reflux under nitrogen atmosphere (balloon). The crude reaction mixture was evaporated under reduced pressure to remove the residual solvent. The resulting oil was purified using a high vacuum distillation apparatus at 120 °C to remove traces of the starting materials. The residual oil was triturated using cold dichloromethane (10 mL) and evaporated under reduced pressure. The resulting crystals were dried under vacuum to obtain **S6** as a hygroscopic light brown solid (1.80 g, 65%).[a]

Spectral data for this compound were consistent with those in literature.[15]

[a]The hygroscopic solid was not amenable to melting point determination in air.

δ_H (400 MHz, $CDCl_3$):	3.93-3.75 (m, 4H), 3.69-3.60 (m, 2H), 3.31 (s, 3H), 2.35-2.25 (m, 4H), 1.81-1.70 (m, 2H, overlapped H_2O), 1.45-1.18 (m, 10H), 0.87 (t, 3H, *J* 6.9) ppm.
δ_C (100 MHz, $CDCl_3$):	64.5, 64.3, 48.8, 31.7, 29.3, 29.1, 26.5, 24.2, 22.7, 21.8, 14.1 ppm.
HRMS (*m/z* – ESI^+):	Found: 198.2218 (M^+) $C_{13}H_{28}N^+$ Requires: 198.2216.
ν_{max} (neat)/cm^{-1}:	3432, 2924, 2856, 1463, 1378, 1305, 1004, 932, 825, 724.

1-Methyl-1-octylpyrrolidinium hydrocinnamate (42)

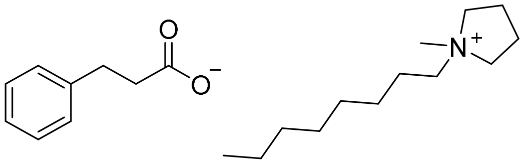

To a 50 mL round-bottomed flask, **S6** (0.14 g, 0.5 mmol) and MeOH (10 mL) were added. Amberlite-OH (bed volume ≈ 2.5 mL, 3 meq of total exchange capacity)[a] was added to the solution and the flask was shaken for 16 h on an orbital shaker. The resulting solution was passed through a plug of Amberlite-OH (bed volume ≈ 2.5 mL, 3 meq of total exchange capacity). Hydrocinnamic acid (0.08 g, 0.5 mmol) was added, and the resulting solution was stirred for 16 h. The solvent was removed under reduced pressure and the product was dried under vacuum for 8 h at 60 °C to yield **42** as an off-white gum (0.17 g, 99%).

[a]Bed volume (mL) is measured in a burette or graduated cylinder as packed resin beads soaked in methanol.

δ_H (400 MHz, $CDCl_3$):	7.24-7.15 (m, 4H), 7.07 (t, 1H), 3.79-3.69 (m, 2H), 3.68-3.56 (m, 2H), 3.50-3.42 (m, 2H), 3.16 (s, 3H), 2.97-2.90 (m, 2H), 2.49-2.42 (m, 2H), 2.23-2.08 (m, 4H), 1.72-1.59 (m, 2H), 1.38-1.14 (m, 10H), 0.83 (t, 3H, *J* 6.6) ppm.
δ_C (100 MHz, $CDCl_3$):	178.3, 143.9, 128.5, 128.1, 125.2, 64.0, 63.8, 48.2, 40.7, 33.5, 31.7, 29.2, 29.0, 26.5, 24.0, 22.6, 21.7, 14.1 ppm.
HRMS (*m/z* – ESI^+):	Found: 198.2219 (M^+) $C_{13}H_{28}N^+$ Requires: 198.2216.
ν_{max} (neat)/cm^{-1}:	3322, 3026, 2925, 2856, 1572, 1454, 1379, 1076, 1003, 932, 749, 699.

Methyloctylpyrrolidinium benzoate (43)

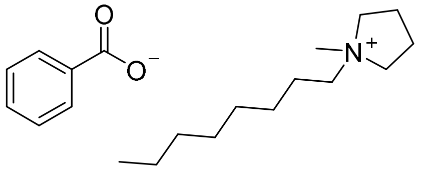

To a 50 mL round-bottomed flask, **S6** (0.14 g, 0.5 mmol) and MeOH (10 mL) were added. Amberlite-OH (bed volume ≈ 2.5 mL, 3 meq of total exchange capacity)[a] was added to the solution and the flask was shaken for 16 h on an orbital shaker. The resulting solution was passed through a plug of Amberlite-OH (bed volume ≈ 2.5 mL, 3 meq of total exchange capacity). Benzoic acid (0.06 g, 0.5 mmol) was added, and the resulting solution was stirred for 16 h. The solvent was removed under reduced pressure and the product was dried under vacuum for 8 h at 60 °C to yield **43** as an off-white gum (0.16 g, 99%).

[a]Bed volume (mL) is measured in a burette or graduated cylinder as packed resin beads soaked in methanol.

δ_H (400 MHz, $CDCl_3$):	8.11-8.04 (m, 2H), 7.32-7.27 (m, 3H), 3.92-3.82 (m, 2H), 3.79-3.68 (m, 2H), 3.58-3.50 (m, 2H), 3.28 (s, 3H), 2.29-2.16 (m, 4H), 1.75-1.64 (m, 2H), 1.35-1.18 (m, 10H), 0.86 (t, 3H, *J* 6.9) ppm.
δ_C (100 MHz, $CDCl_3$):	172.1, 139.9, 129.5, 129.3, 127.5, 64.3, 64.1, 48.5, 31.8, 29.3, 29.1, 26.5, 24.1, 22.7, 21.8, 14.2 ppm.
HRMS (*m/z* – ESI^+):	Found: 198.2215 (M^+) $C_{13}H_{28}N^+$ Requires: 198.2216.
ν_{max} (neat)/cm^{-1}:	3351, 2926, 2856, 1601, 1562, 1467, 1360, 1169, 1063, 1023, 933, 825, 716, 670.

Dimethyldidecylammonium oleate (44)

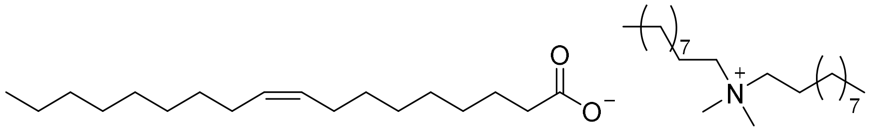

To a 50 mL round-bottomed flask, didecyldimethylammonium bromide (0.81 g, 2 mmol) and MeOH (10 mL) were added. Amberlite-OH (bed volume ≈ 5.0 mL, 6 meq of total exchange capacity)[a] was added to the solution and the flask was shaken for 16 h on an orbital shaker. The resulting solution was passed through a plug of Amberlite-OH (bed volume ≈ 5.0 mL, 6 meq of total exchange capacity). Oleic acid (0.64 mL, 2 mmol) was added, and the resulting solution was stirred for 16 h. The solvent was removed under reduced pressure and the product was dried under vacuum for 8 h at 60 °C to yield **44** as a white gum (1.19 g, 98%).

[a]Bed volume (mL) is measured in a burette or graduated cylinder as packed resin beads soaked in methanol.

Spectral data for this compound were consistent with those in literature.[16]

δ_H (400 MHz, $CDCl_3$):	5.38-5.27 (m, 2H), 3.48-3.40 (m, 4H), 3.38 (s, 6H), 2.21-2.15 (m, 2H), 2.03-1.94 (m, 4H), 1.73-1.56 (m, 6H), 1.42-1.18 (m, 48H), 0.91-0.83 (m, 9H) ppm.
δ_C (100 MHz, $CDCl_3$):	180.0, 130.1, 129.8, 63.7, 51.2, 39.5, 31.9, 31.8, 30.1, 29.9, 29.8, 29.7, 29.5, 29.5, 29.4, 29.4, 29.3, 29.3, 29.2, 29.2, 27.3, 27.3, 27.2, 26.3, 22.8, 22.7, 22.7, 14.1, 14.1 ppm.
HRMS (*m/z* – ESI^+):	Found: 326.3792 (M^+) $C_{22}H_{48}N^+$ Requires: 326.3781.
ν_{max} (neat)/cm^{-1}:	3314, 2921, 2852, 1575, 1467, 1383, 911, 881, 722.

5 pK$_a$ data associated with Scheme 1 – literature data

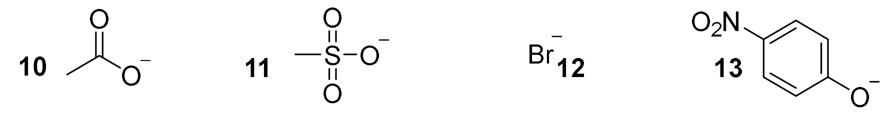

	10	11	12	13
p$Ka_{H\text{DMSO}}$	12.6 (Ref 17)	1.6 (Ref 19)	0.9 (Ref 21)	10.8 (Ref 23)
p$Ka_{H\text{water}}$	4.8 (Ref 18)	-0.6 (Ref 20)	-10 (Ref 22)	7.1 (Ref 24)

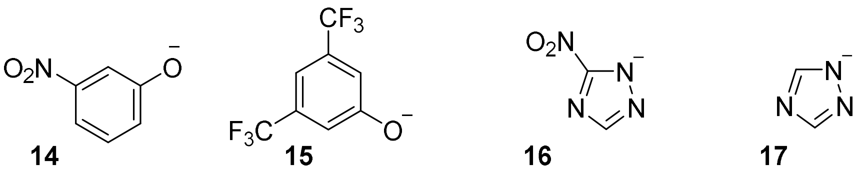

	14	15	16	17
p$K_{aH\text{DMSO}}$	14.4 (Ref 25)	13.2 (Ref 27)		14.8 (Ref 21)
p$K_{aH\text{water}}$	8.4 (Ref 26)	8.3 (Ref 27)	6.0 (Ref 28)	10.1 (Ref 29)

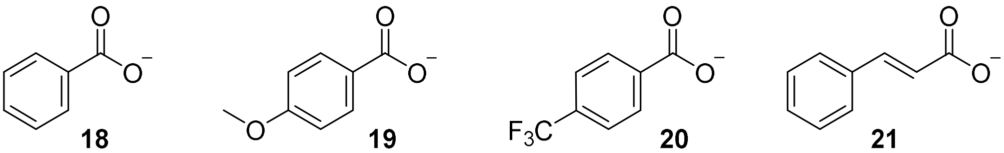

	18	19	20	21
p$K_{aH\text{DMSO}}$	10.6 (Ref 30)	11.8 (Ref 31)		
p$K_{aH\text{water}}$	4.2 (Ref 18)	4.3 (Ref 31)	3.7 (Ref 32)	4.4 (Ref 33)

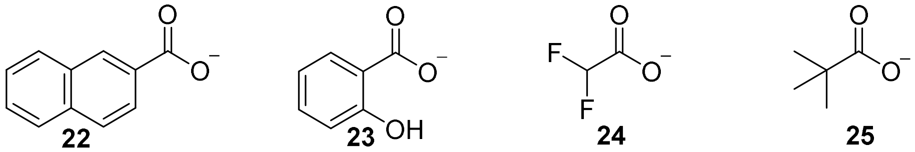

	22	23	24	25
p$K_{aH\text{DMSO}}$	10.9 (Ref 34)	6.8 (Ref 18)	6.5 (Ref 37)	10.9 (Ref 17)
p$K_{aH\text{water}}$	4.1 (Ref 35)	3.2 (Ref 36)	1.3 (Ref 38)	5.1 (Ref 39)

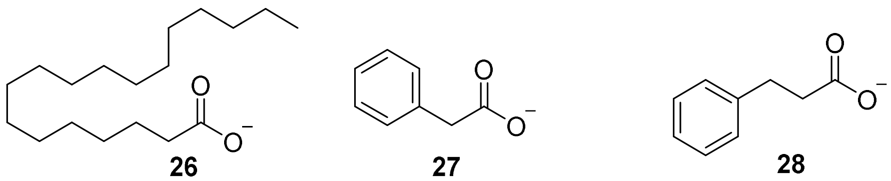

	26	27	28
p$K_{aH\text{DMSO}}$		11.6 (Ref 41)	
p$K_{aH\text{water}}$	5.8 (Ref 40)	4.3 (Ref 42)	4.7 (Ref 33)

Table S1. pK$_a$ data associated to Scheme 1 – literature data

6 References

1. Bura, D.; Pedrini, L.; Trujillo, C.; Connon, S. J. Cholinium-based ionic liquid catalysts for polyethylene terephthalate glycolysis: understanding the role of solvent and a reappraisal of the cation contribution. *RSC Sustain.* **2023**, *1*, 2197–2201.
2. Messa, F.; Paparella, A. N.; Perrone, S.; Salomone, A. Gas-Free Alkoxycarbonylation of Aryl Iodides in a Phosphonium-Based Deep Eutectic Solvent with $Mo(CO)_6$ as a Solid CO Source. *Org. Biomol. Chem.* **2023**, *21*, 5164-5170.
3. Fehér, Z.; Kiss, J.; Kisszékelyi, P.; Molnár, J.; Huszthy, P.; Kárpáti, L.; Kupai, J. Optimisation of PET Glycolysis by Applying Recyclable Heterogeneous Organocatalysts. *Green Chem.* **2022**, *24*, 8447–8459.
4. Bahadoor, A.; Brinkmann, A.; Melanson, J. E. 13C-Satellite Decoupling Strategies for Improving Accuracy in Quantitative Nuclear Magnetic Resonance. *Anal. Chem.* **2020**, *93*, 851-858.
5. Avila, J.; Lepre, L. F.; Santini, C. C.; Tiano, M.; Denis-Quanquin, S.; Chung Szeto, K.; Padua, A. A. H.; Costa Gomes, M. High-Performance Porous Ionic Liquids for Low-Pressure CO_2 Capture. *Angew. Chem.* **2021**, *133*, 12986-12992.
6. Kohno, Y.; Arai, H.; Saita, S.; Ohno, H. Material Design of Ionic Liquids to Show Temperature-Sensitive LCST-Type Phase Transition after Mixing with Water. *Aust. J. Chem.* **2011**, *64*, 1560-1567.
7. Barman, B. K.; Barman, S.; Roy, M. N. Inclusion Complexation between Tetrabutylphosphonium Methanesulfonate as Guest and α- and β-Cyclodextrin as Hosts Investigated by Physicochemical Methodology. *Mol. Liq.* **2018**, *264*, 80-87.
8. Gao, X.; Zhao, J.; Gao, Y.; Deng, Y.; Shi, Y.; He, J.; Li, Y. Benoxazolone-Based Ionic Liquid Catalyzed C–S Bond Construction for Synthesis of Benzothiazoles from 2-Aminothiophenols and CO_2 under Ambient Conditions. *New J. Chem.* **2023**, *47*, 17449-17455.
9. Gulotty, E. M.; Sanadhya, S.; Tucker, Z. D.; Moghaddam, S. S.; Ashfeld, B. L. Controlling Phase Separation Behavior of Thermo-Responsive Ionic Liquids through the Directed Distribution of Anionic Charge. *J. Mol. Liq.* **2022**, *360*, 119401.

10. Bousrez, G.; Renier, O.; Paterlini, V.; Smetana, V.; Mudring, A.-V. Magnetic, Photo- and Electroluminescent: Multifunctional Ionic Tb Complexes. *Inorg. Chem.* **2021**, *60*, 17487-17497.
11. Selva, M.; Fabris, M.; Lucchini, V.; Perosa, A.; Noè, M. The Reaction of Primary Aromatic Amines with Alkylene Carbonates for the Selective Synthesis of Bis-N-(2-Hydroxy)Alkylanilines: The Catalytic Effect of Phosphonium-Based Ionic Liquids. *Org. Biomol. Chem.* **2010**, *8*, 5187-5198.
12. Lichtin, N. N.; Wasserman, B.; Clougherty, E.; Wasserman, J.; Reardon, J. F. Ionization and Dissociation Equilibriums in Liquid Sulfur Dioxide. 12. The Behavior of Tetrahedral Ions. *J. Phys. Chem.* **1980**, *84*, 2946–2952.
13. Anderson, L. B.; Molloy, C.; Pedrini, L.; Martin, I. L.; Connon, S. J. Effect of phase transfer catalyst structure on the alkaline hydrolysis of poly(ethylene terephthalate). *Green. Chem*. **2024**, *26*, DOI: 10.1039/D4GC05070C
14. Taylor, M. J.; Calvert, D. J.; Hobbis, C. M. Carbon-13 to Nitrogen-14 Coupling Constants in Some Heterocyclic Cations Containing Quaternary Nitrogen Atoms. *Magn. Reson. Chem.* **1988**, *26*, 619-621.
15. Feliciano, J. A.; Leitgeb, A. J.; Schrank, C. L.; Allen, R. A.; Minbiole, K. P. C.; Wuest, W. M.; Carden, R. G. Trivalent Sulfonium Compounds (TSCs): Tetrahydrothiophene-Based Amphiphiles Exhibit Similar Antimicrobial Activity to Analogous Ammonium-Based Amphiphiles. *Bioorg. Med. Chem. Lett.* **2021**, 37, 127809.
16. Pernak, J.; Łęgosz, B.; Walkiewicz, F.; Klejdysz, T.; Borkowski, A.; Chrzanowski, Ł. Ammonium Ionic Liquids with Anions of Natural Origin. *RSC Adv.* **2015**, *5*, 65471-65480.
17. Bartnicka, H.; Bojanowska, I.; Kalinowski, M. Solvent Effect on the Dissociation Constants of Aliphatic Carboxylic Acids. *Aust. J. Chem.* **1991**, *44*, 1077.
18. Kolthoff, I. M.; Chantooni, M. K., Jr.; Bhowmik, S. Dissociation Constants of Uncharged and Monovalent Cation Acids in Dimethyl Sulfoxide. *J. Am. Chem. Soc.* **1968**, *90*, 23–28.
19. Bordwell, F. G.; Algrim, D. Nitrogen Acids. 1. Carboxamides and Sulfonamides. *J. Org. Chem.* **1976**, *41*, 2507–2508.
20. Klages, F.; Bott, K.; Hegenberg, P.; Jung, H. A. Aciditätsbestimmung Mit Hilfe Aliphatischer Diazoverbindungen. II. Die Aufstellung Einer Dynamischen Aciditätsreihe. *Chem. Ber.* **1965**, *98*, 3765–3776.

21. Bordwell, F. G. Equilibrium Acidities in Dimethyl Sulfoxide Solution. *Acc. Chem. Res.* **1988**, *21*, 456–463.
22. Pearson, R. G. Ionization Potentials and Electron Affinities in Aqueous Solution. *J. Am. Chem. Soc.* **1986**, *108*, 6109–6114.
23. Taft, R. W.; Bordwell, F. G. Structural and Solvent Effects Evaluated from Acidities Measured in Dimethyl Sulfoxide and in the Gas Phase. *Acc. Chem. Res.* **1988**, *21*, 463–469.
24. Li, C.; Hoffman, M. Z. One-Electron Redox Potentials of Phenols in Aqueous Solution. *The Journal of Physical Chemistry B* **1999**, *103*, 6653–6656.
25. Bordwell, F. G.; McCallum, R. J.; Olmstead, W. N. Acidities and Hydrogen Bonding of Phenols in Dimethyl Sulfoxide. *J. of Org. Chem.* **1984**, *49*, 1424–1427
26. Pearce, P. J.; Simkins, R. J. J. Acid Strengths of Some Substituted Picric Acids. *Can. J. Chem.* **1968**, *46*, 241–248.
27. Kütt, A.; Movchun, V.; Rodima, T.; Dansauer, T.; Rusanov, E. B.; Leito, I.; Kaljurand, I.; Koppel, J.; Pihl, V.; Koppel, I.; et al. Pentakis(Trifluoromethyl)Phenyl, a Sterically Crowded and Electron-Withdrawing Group: Synthesis and Acidity of Pentakis(Trifluoromethyl)Benzene, -Toluene, -Phenol, and -Aniline. *J. Org. Chem.* **2008**, *73*, 2607–2620.
28. Catalan, J.; Elguero, J. Basicity and Acidity of Azoles. *Adv. Heterocycl. Chem.* **1987**, 187–274.
29. Catalan, J.; Sanchez-Cabezudo, M.; de Paz, J. L. G.; Elguero, J. Acidity and Basicity of Azoles: Solvent Effects. *J. Mol. Struct.: THEOCHEM* **1988**, *166*, 415–420.
30. Moriyama, K.; Sugiue, T.; Saito, Y.; Katsuta, S.; Togo, H. 2,6-Bis(Amido)Benzoic Acid with Internal Hydrogen Bond as Brønsted Acid Catalyst for Friedel–Crafts Reaction of Indoles. *Adv. Synth. Catal.* **2015**, *357*, 2143–2149.
31. Jover, J.; Bosque, R.; Sales, J. QSPR Prediction of pKa for Benzoic Acids in Different Solvents. *QSAR Comb. Sci.* **2008**, *27*, 563–581.
32. Boiadjiev, S. E.; Lightner, D. A. Carboxylic Acid Ionization Constants by ^{19}F NMR Spectroscopy. *J. Phys. Org. Chem.* **1999**, *12*, 751–757.

33. Hoefnagel, A. J.; Hoefnagel, M. A.; Wepster, B. M. Substituent Effects. 6. Charged Groups: A Simple Extension of the Hammett Equation. *J. of Org. Chem.* **1978**, *43*, 4720–4745.
34. Pařík, P.; Wolfová, J.; Ludwig, M. Dissociation of Naphthoic Acids in Non-Aqueous Media. Comparison of Benzene and Naphthalene Skeletons. *Collect. Czech. Chem. Commun.* **2000**, *65*, 385–394.
35. Briggs, A. G.; Tickle, P.; Wilson, J. M. Acid-Base Equilibria of Some Naphthoic Acids, Studied Spectrophotometrically. *Spectrochim. Acta, Part A* **1970**, *26*, 1399–1402.
36. Ackermann, G.; Hesse, D.; Volland, P. Über Eisen(III)-Komplexe Mit Phenolen. IV. Säurekonstanten Einiger Polyphenole. *Z. anorg. Allg. Chem.* **1970**, *377*, 92–99.
37. Ritchie, C. D.; Lu, S. Proton Transfers among Oxygen and Nitrogen Acids and Bases in Dimethyl Sulfoxide Solution. *J. Am. Chem. Soc.* **1990**, *112*, 7748–7756.
38. Kurz, J. L.; Farrar, J. M. Entropies of Dissociation of Some Moderately Strong Acids. *J. Am. Chem. Soc.* **1969**, *91*, 6057–6062.
39. Jencks, W. P.; Gilchrist, M. Nonlinear Structure-Reactivity Correlations. The Reactivity of Nucleophilic Reagents toward Esters. *J. Am. Chem. Soc.* **1968**, *90*, 2622–2637.
40. Bowden, K.; Hardy, M.; Parkin, D. C. The Transmission of Polar Effects. Part V. The Kinetics of Esterification with Diazodiphenylmethane and the Ionization of Substituted Acetic and Propionic Acids in Several Solvents. *Can. J. Chem.* **1968**, *46*, 2929–2940.
41. Maran, F.; Celadon, D.; Severin, M. G.; Vianello, E. Electrochemical Determination of the pKa of Weak Acids in N,N-Dimethylformamide. *J. Am. Chem. Soc.* **1991**, *113*, 9320–9329.
42. Pascal, I.; Tarbell, D. S. The Kinetics of the Oxidation of a Mercaptan to the Corresponding Disulfide by Aqueous Hydrogen Peroxide. *J. Am. Chem. Soc.* **1957**, *79*, 6015–6020.

7 NMR Spectra

Product of the glycolysis reaction *bis*(2-hydroxyethyl)terephthalate (BHET, 2).

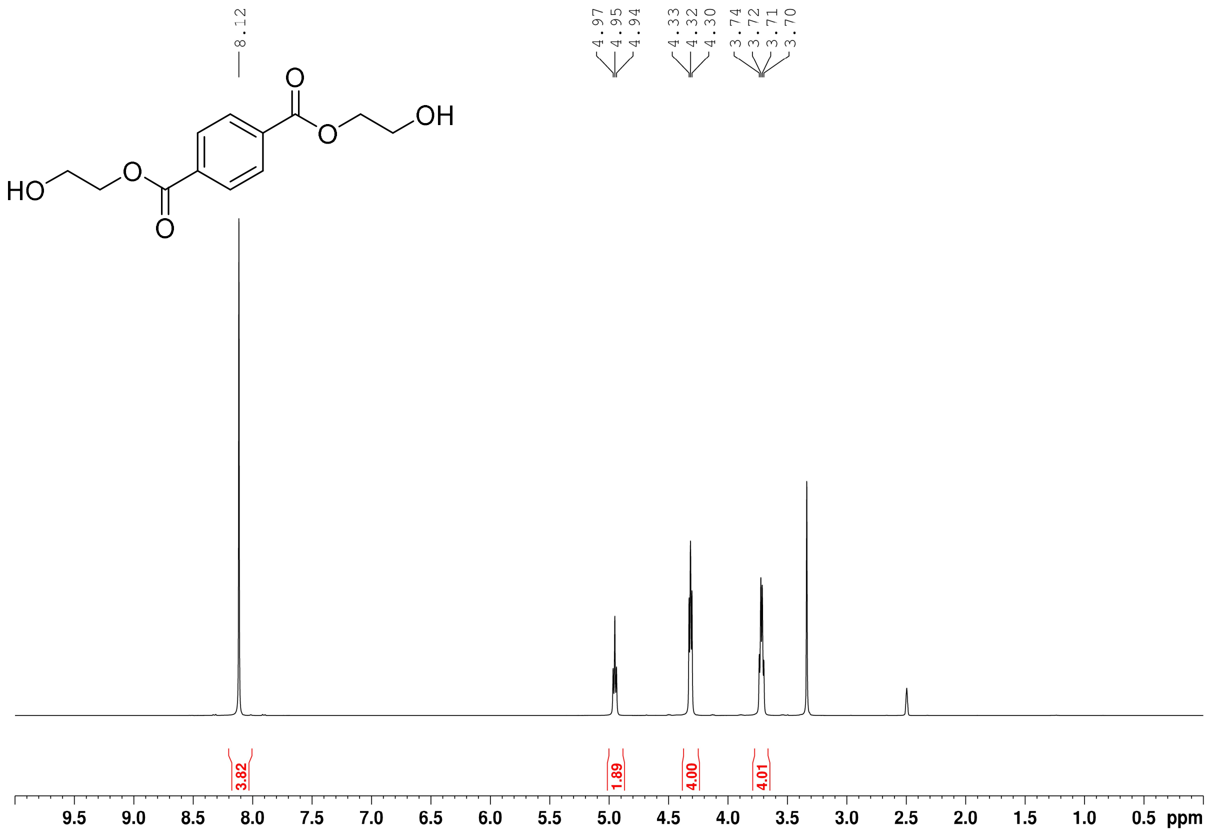

^{1}H NMR spectrum (400 MHz, DMSO-d_6) of **BHET (2)**.

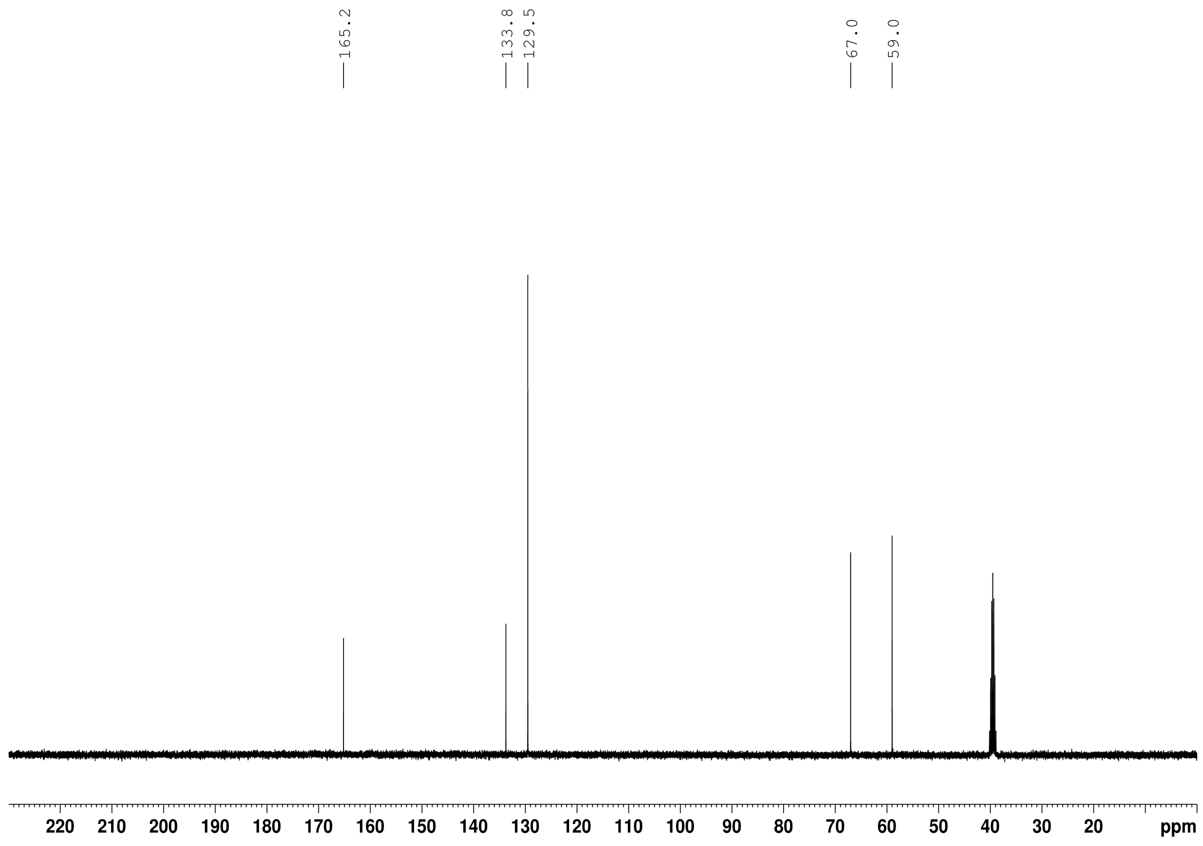

^{13}C NMR spectrum (100 MHz, DMSO-d_6) of **BHET (2).**

Tetrabutylphosphonium acetate (10)

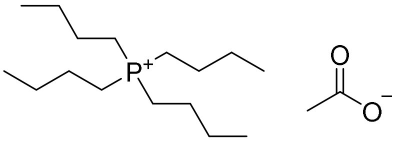

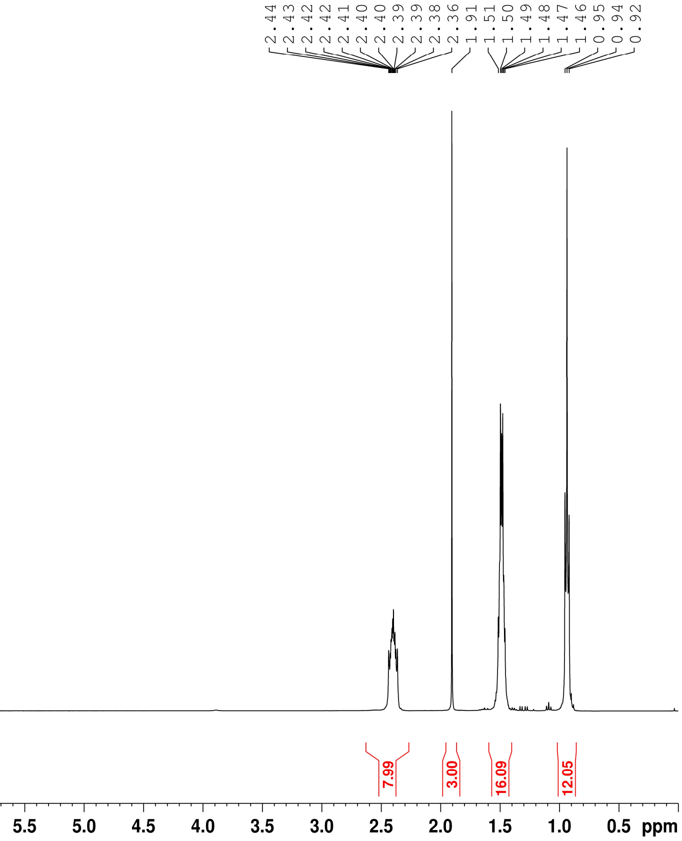

^{1}H NMR spectrum (400 MHz, $CDCl_3$) of **10**.

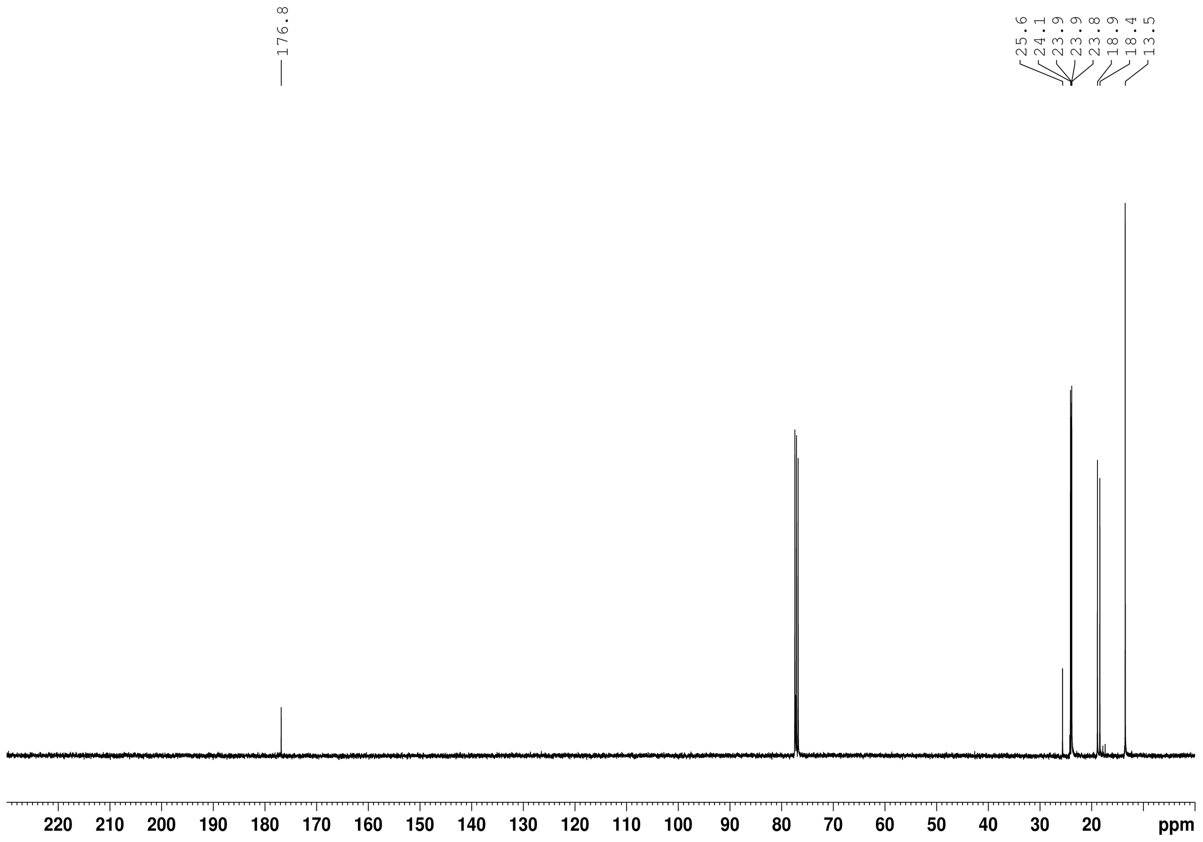

^{13}C NMR spectrum (100 MHz, $CDCl_3$) of **10**.

Tetrabutylphosphonium mesylate (11)

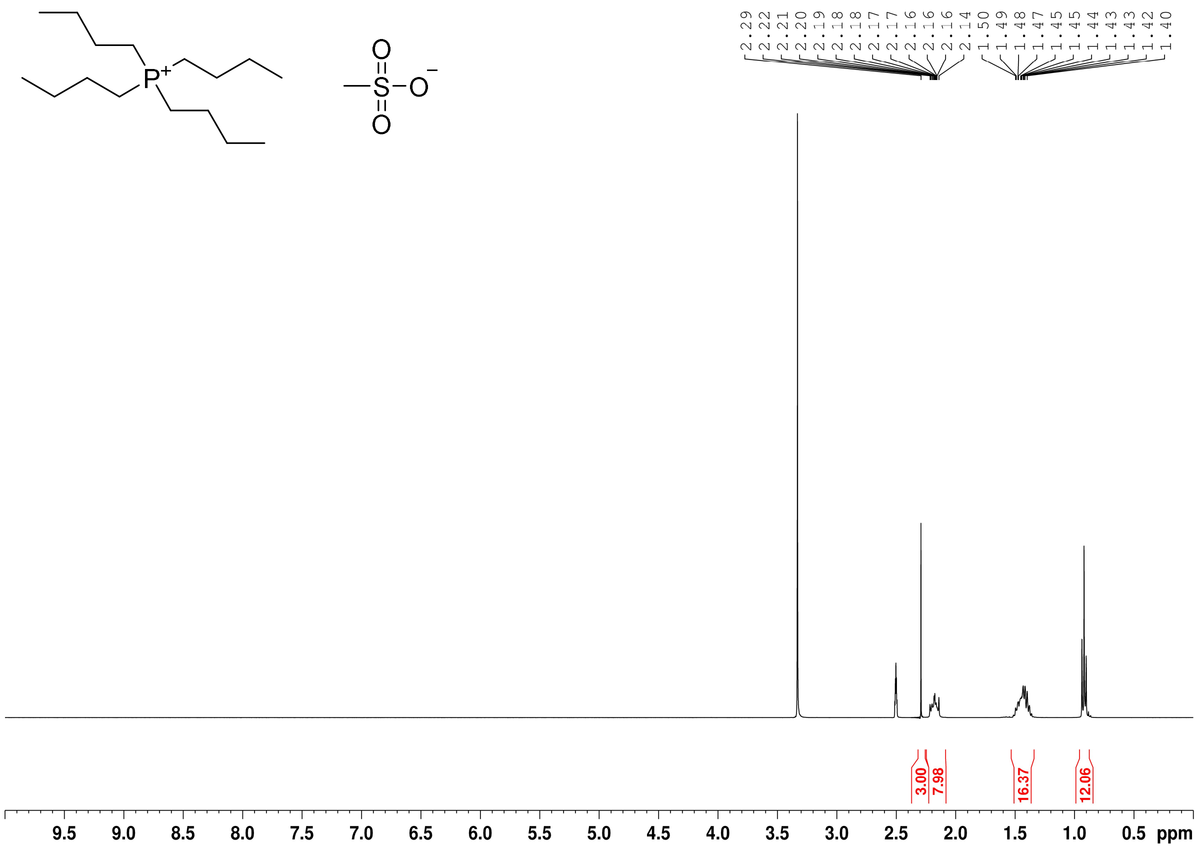

^{1}H NMR spectrum (400 MHz, DMSO-d_6) of **11**.

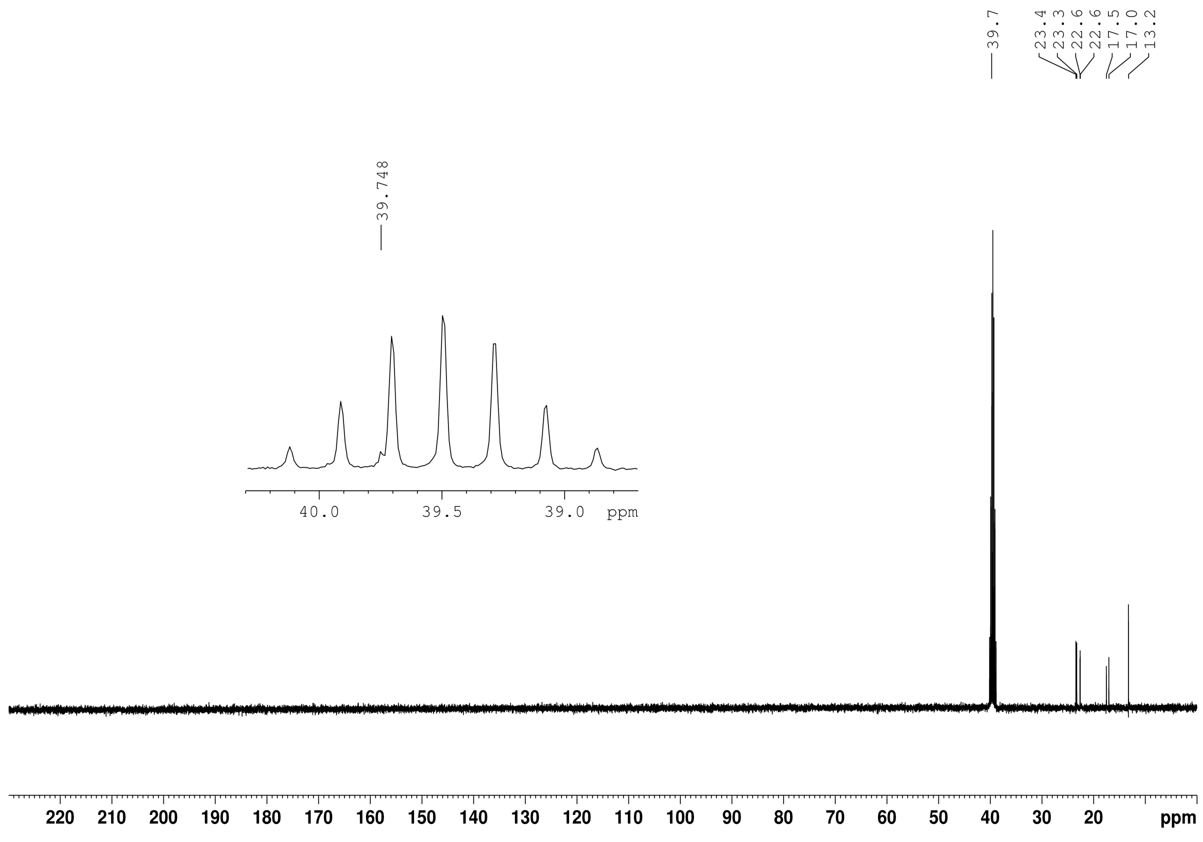

^{13}C NMR spectrum (100 MHz, DMSO-d_6) of **11**.

Tetrabutylphosphonium 4-nitrophenolate (13)

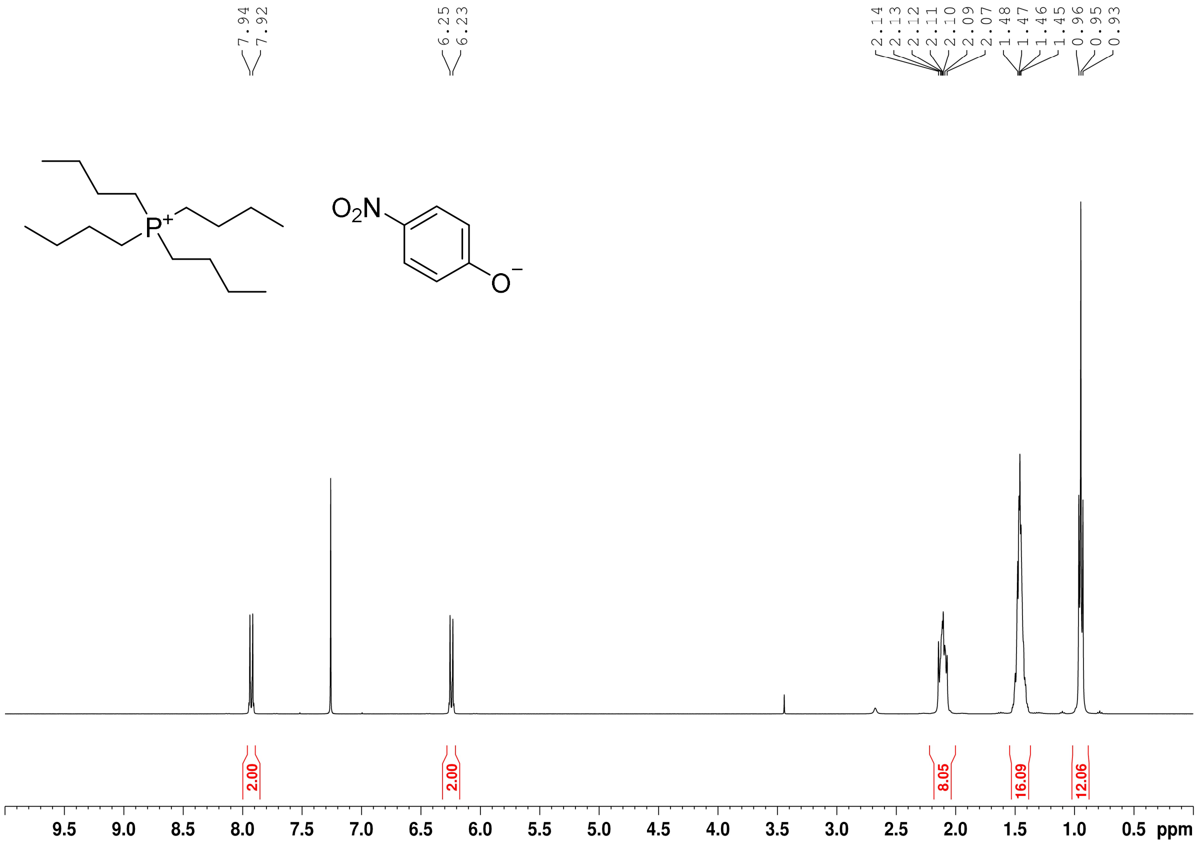

^{1}H NMR spectrum (400 MHz, $CDCl_3$) of **13**.

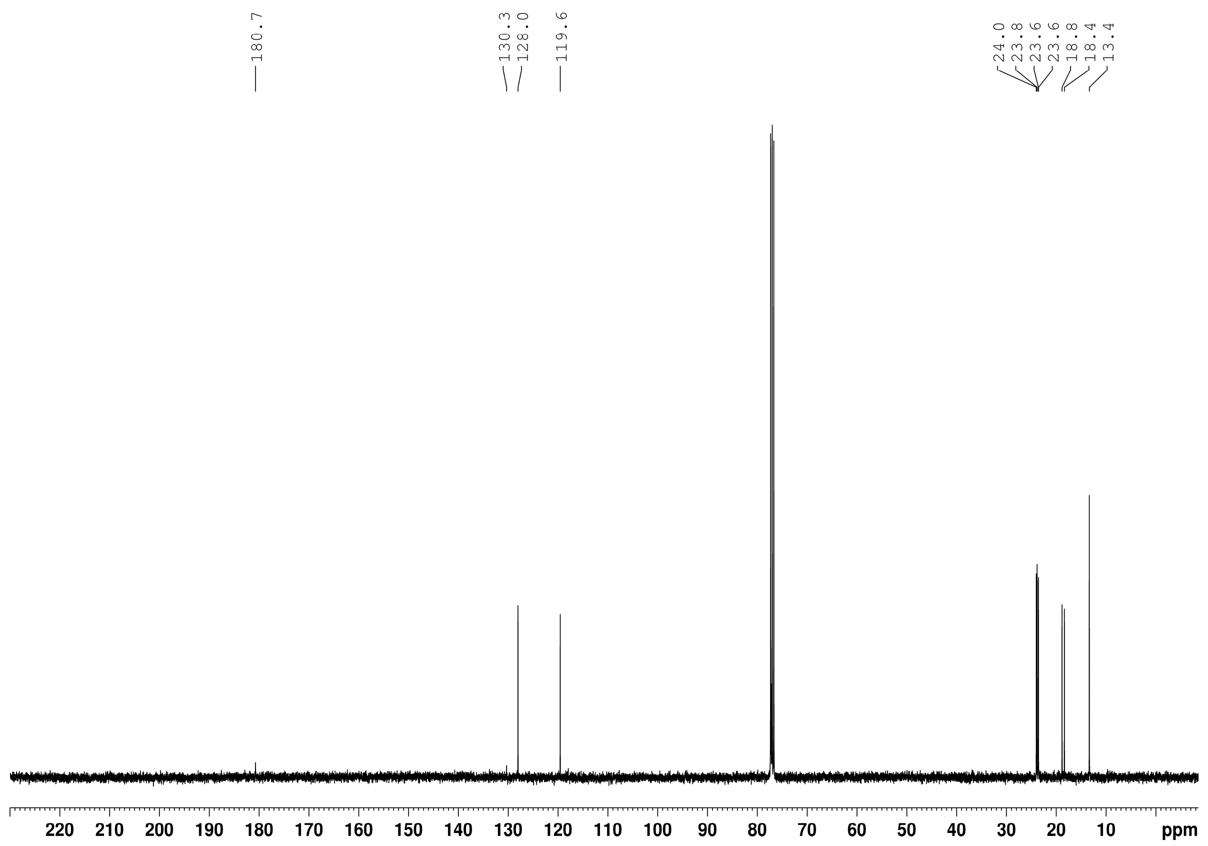

^{13}C NMR spectrum (100 MHz, $CDCl_3$) of **13**.

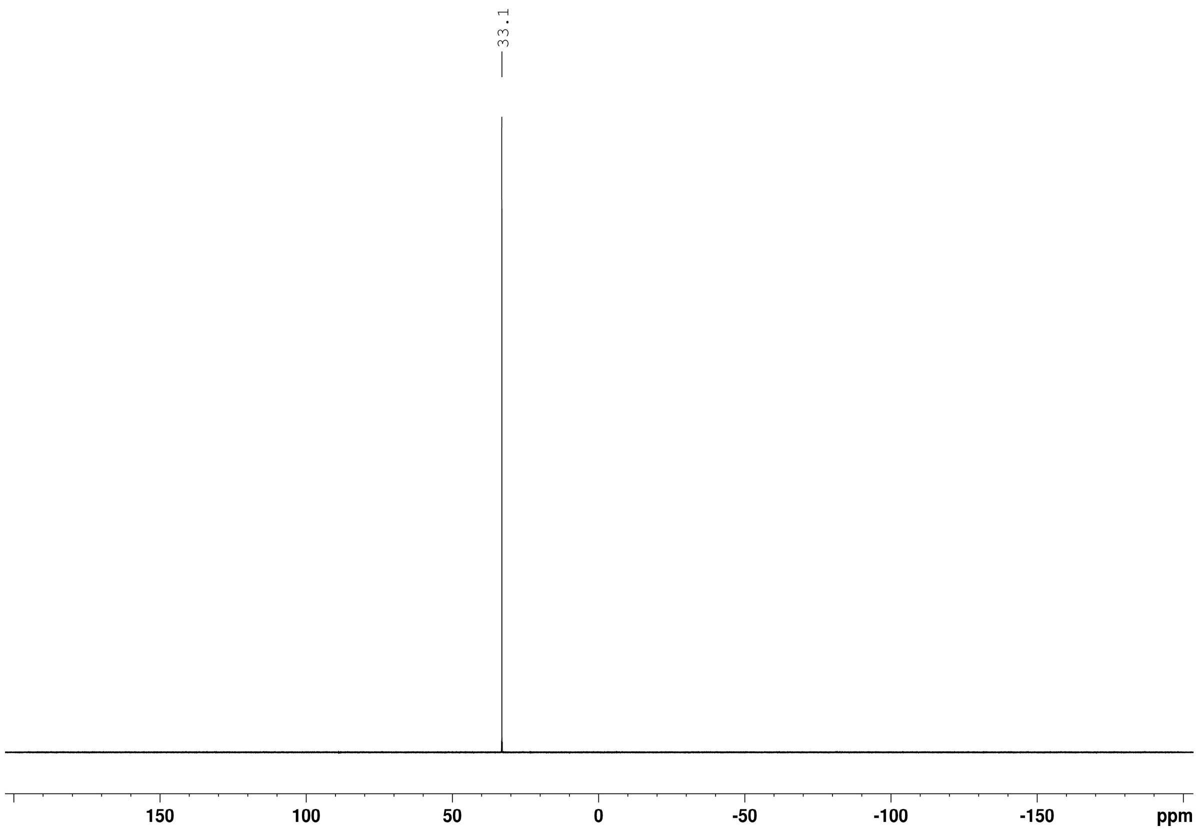

^{31}P NMR spectrum (162 MHz, $CDCl_3$) of **13**.

Tetrabutylphosphonium 3-nitrophenlate (14)

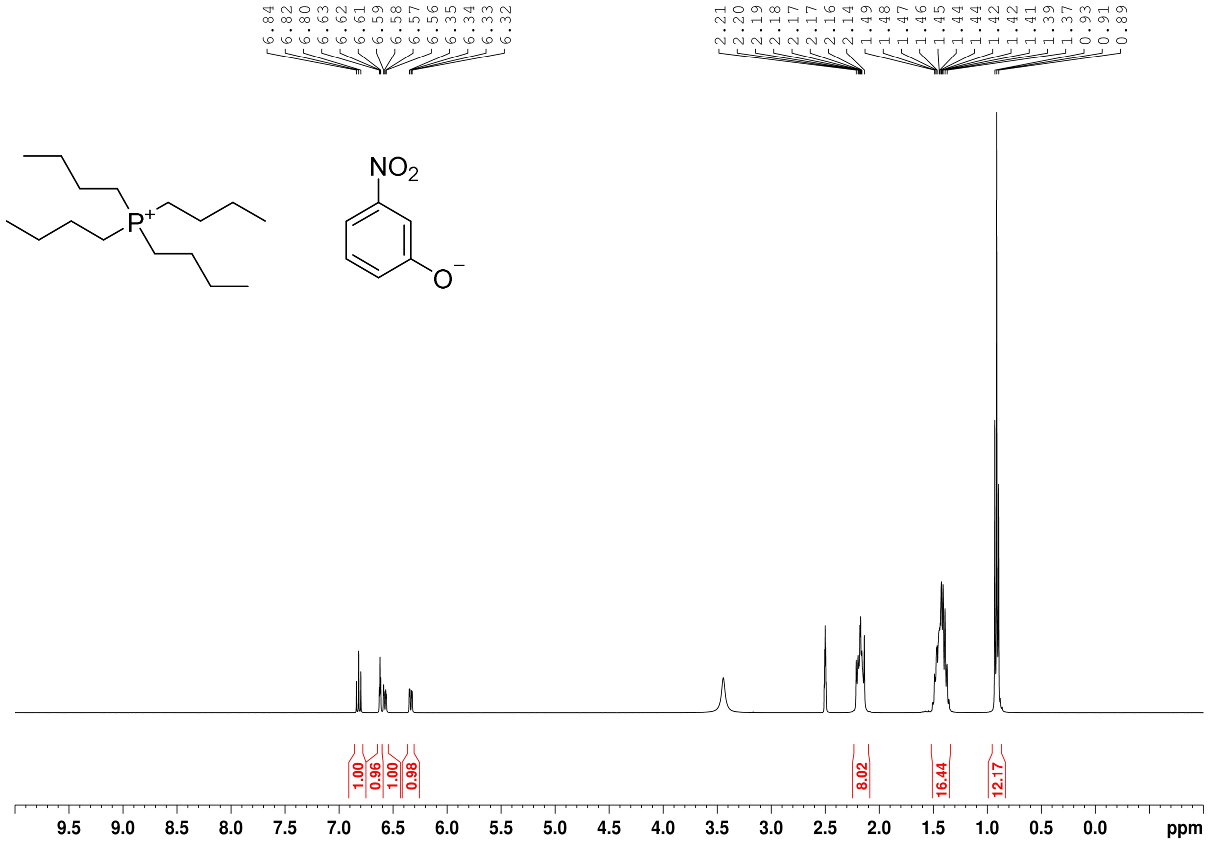

$^{1}H\{C\}$ NMR spectrum (400 MHz, DMSO-d_6) of **14**.

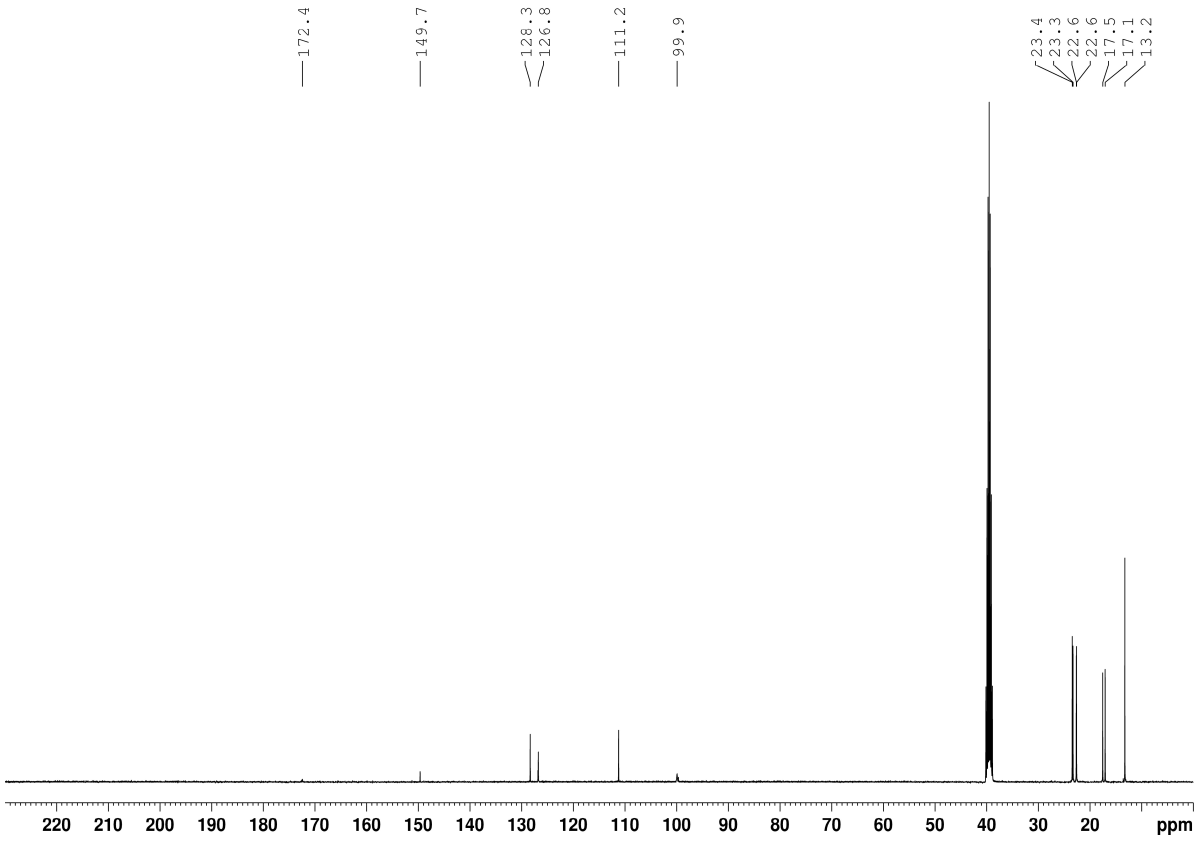

^{13}C NMR spectrum (100 MHz, DMSO-d_6) of **14**.

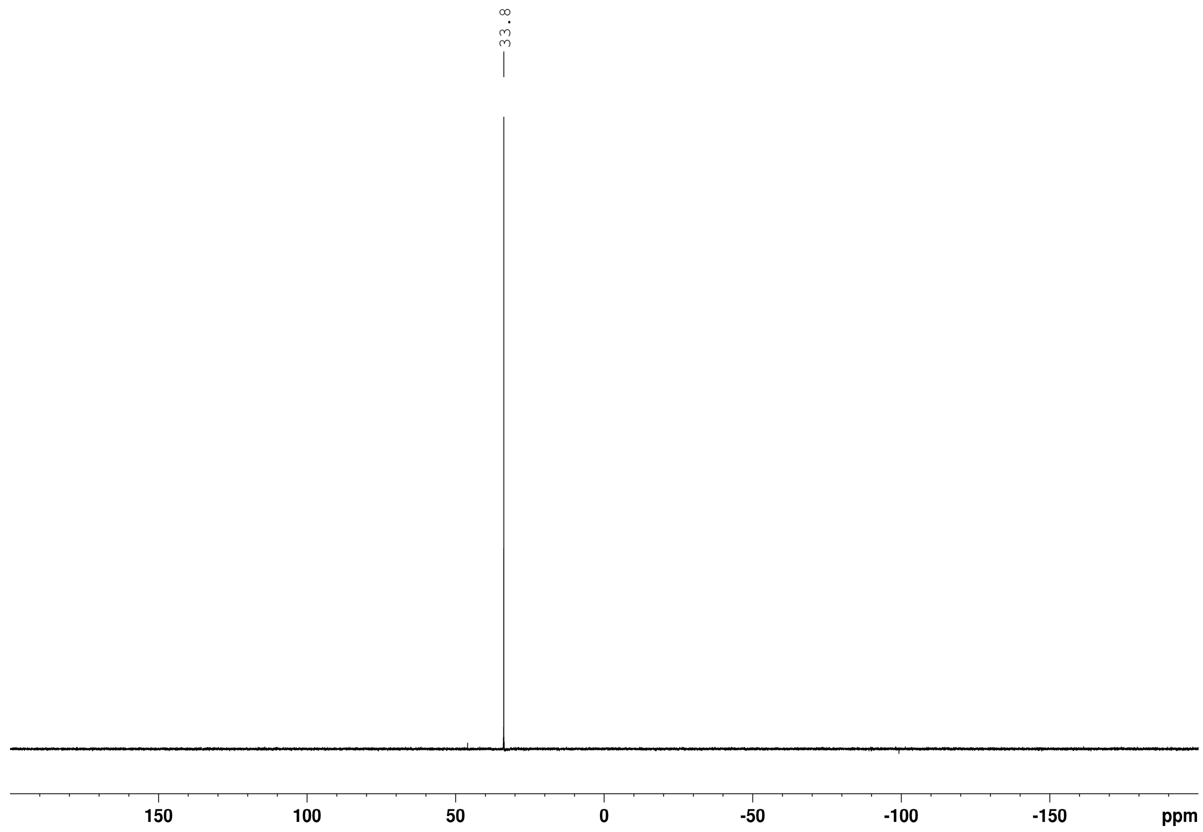

^{31}P NMR spectrum (162 MHz, DMSO-d_6) of **14**.

Tetrabutylphosphonium 3,5-bis(trifluoromethyl)phenolate (15)

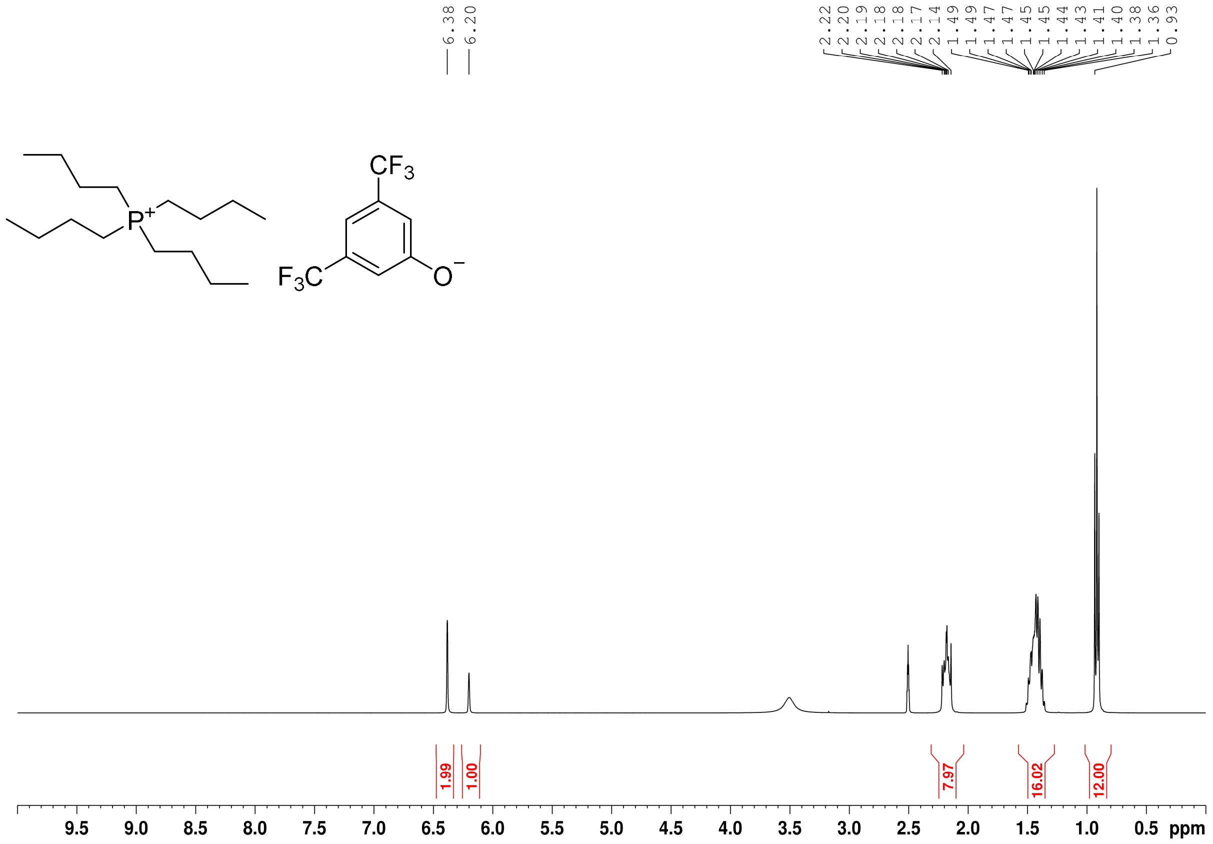

^{1}H NMR spectrum (400 MHz, DMSO-d_6) of **15**.

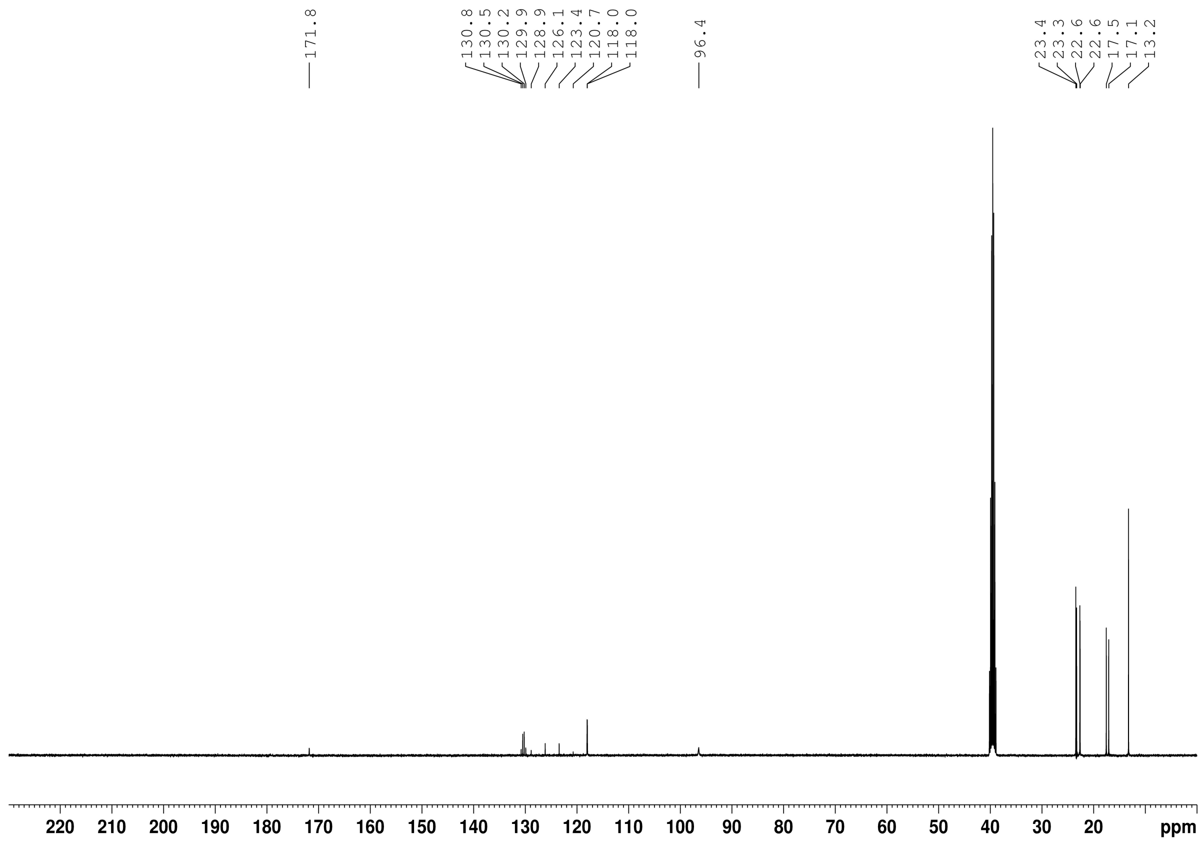

^{13}C NMR spectrum (100 MHz, DMSO-d_6) of **15**.

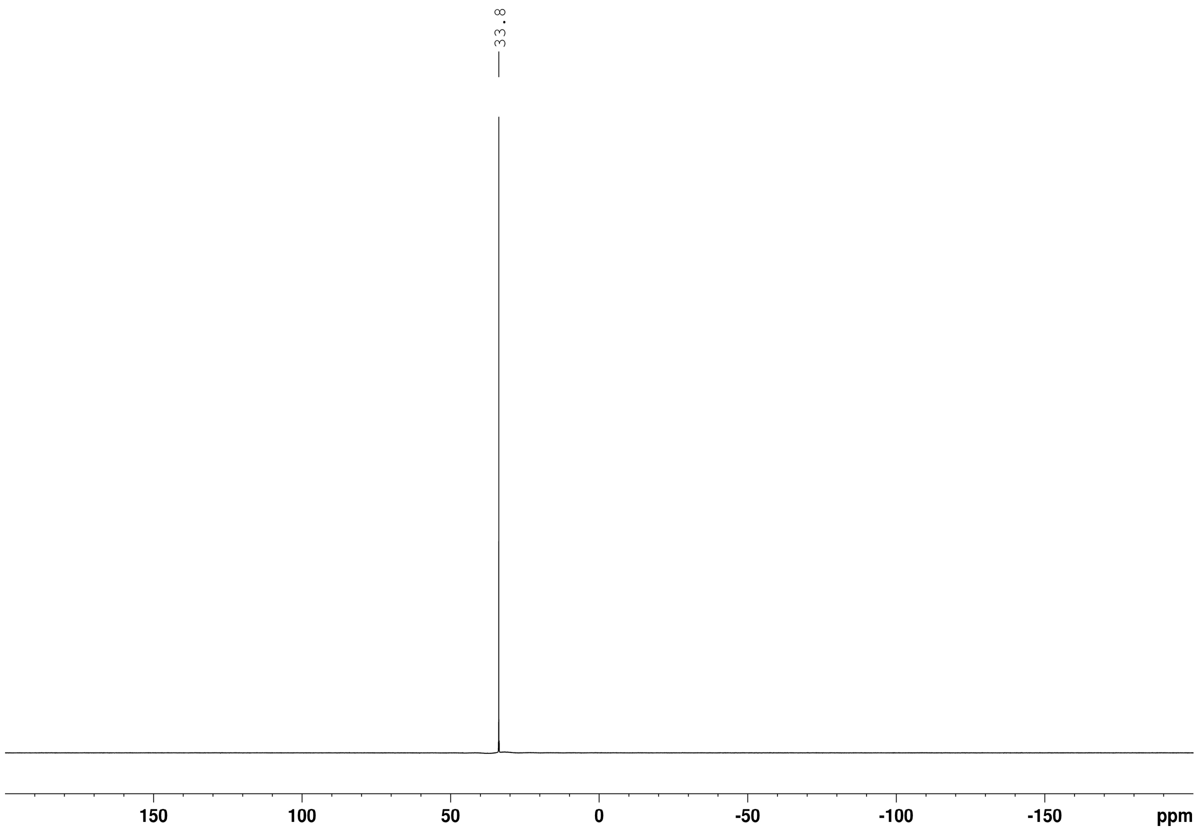

^{31}P NMR spectrum (162 MHz, DMSO-d_6) of **15**.

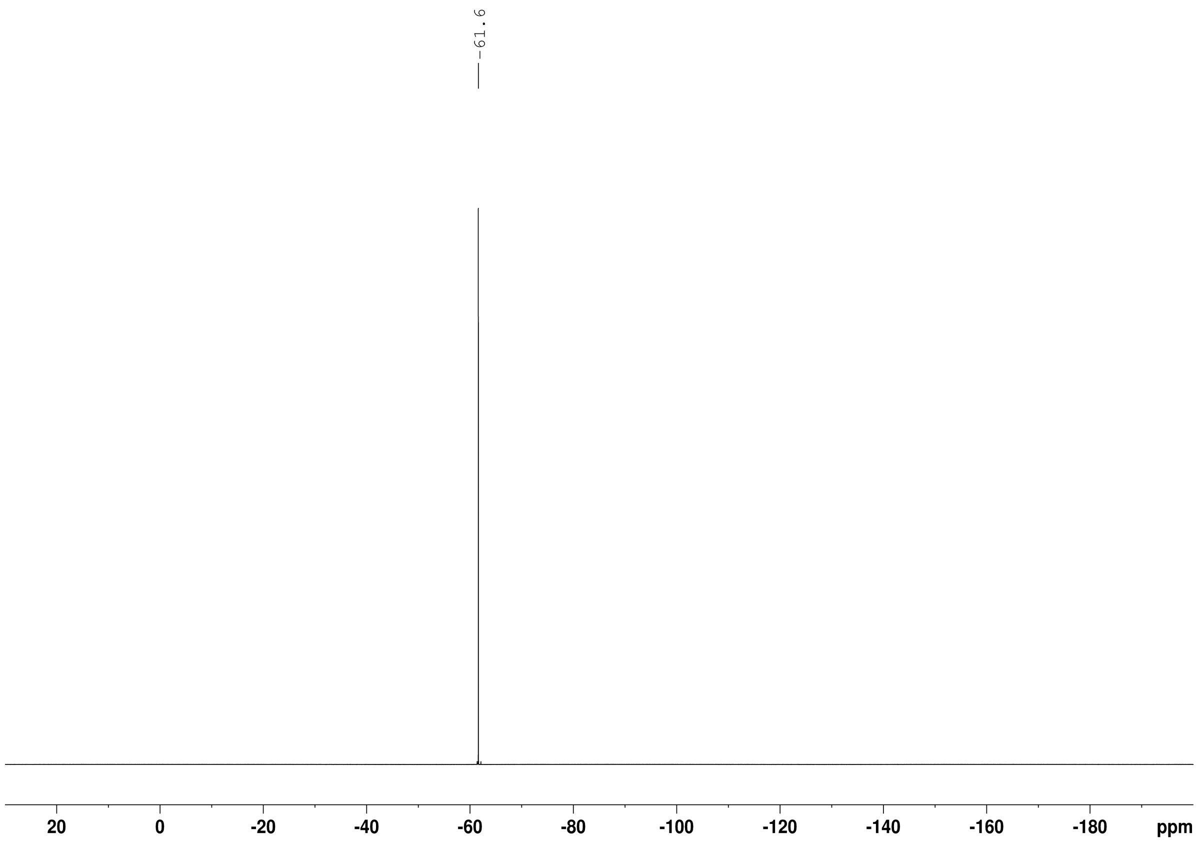

^{19}F NMR spectrum (376 MHz, DMSO-d_6) of **15**.

Tetrabutylphosphonium nitrotriazolate (16)

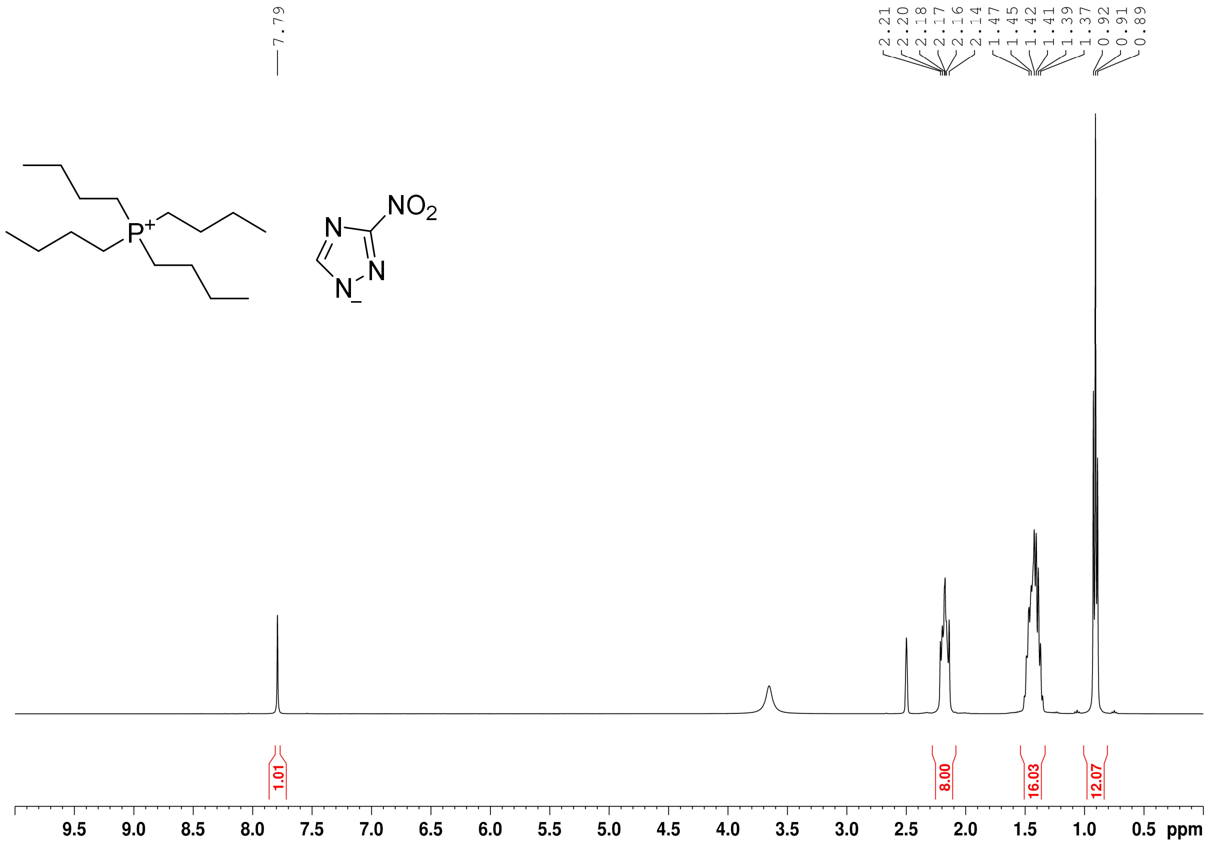

^{1}H NMR spectrum (400 MHz, DMSO-d_6) of **16**.

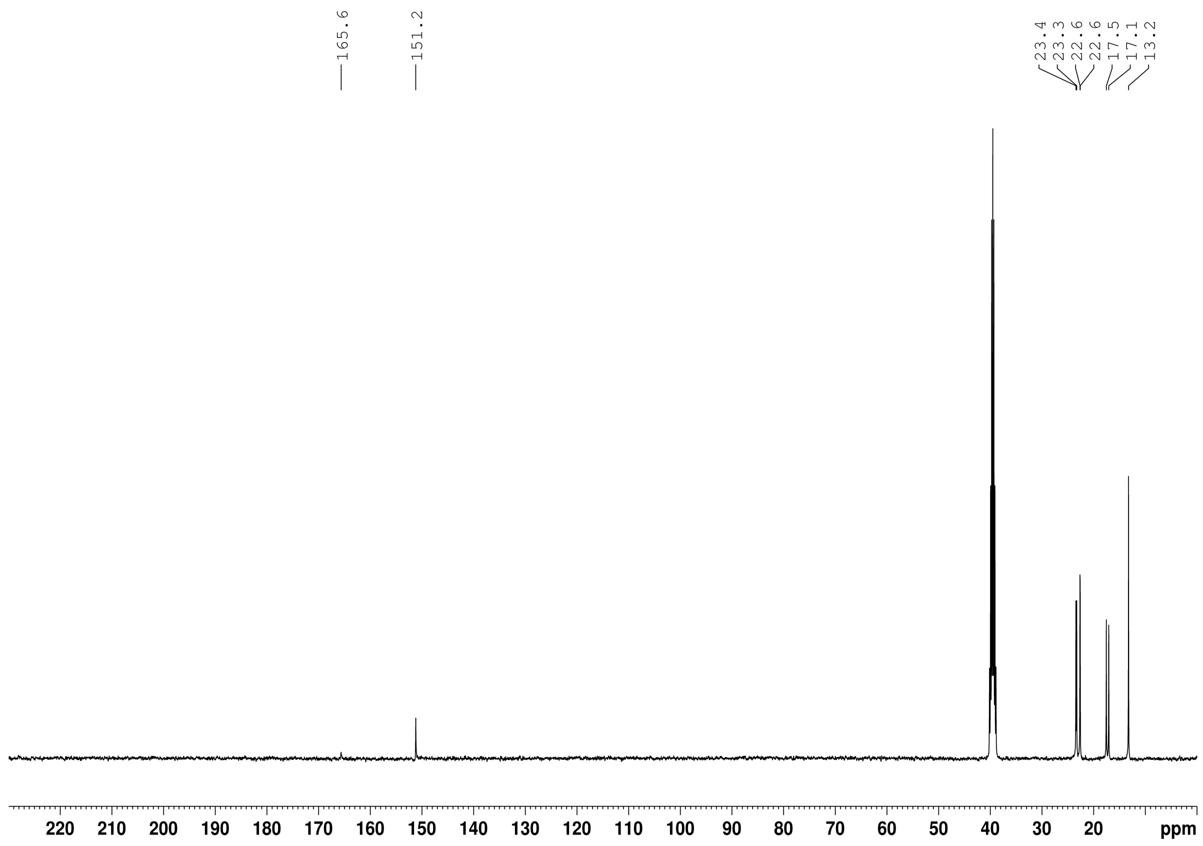

^{13}C NMR spectrum (100 MHz, DMSO-d_6) of **16**.

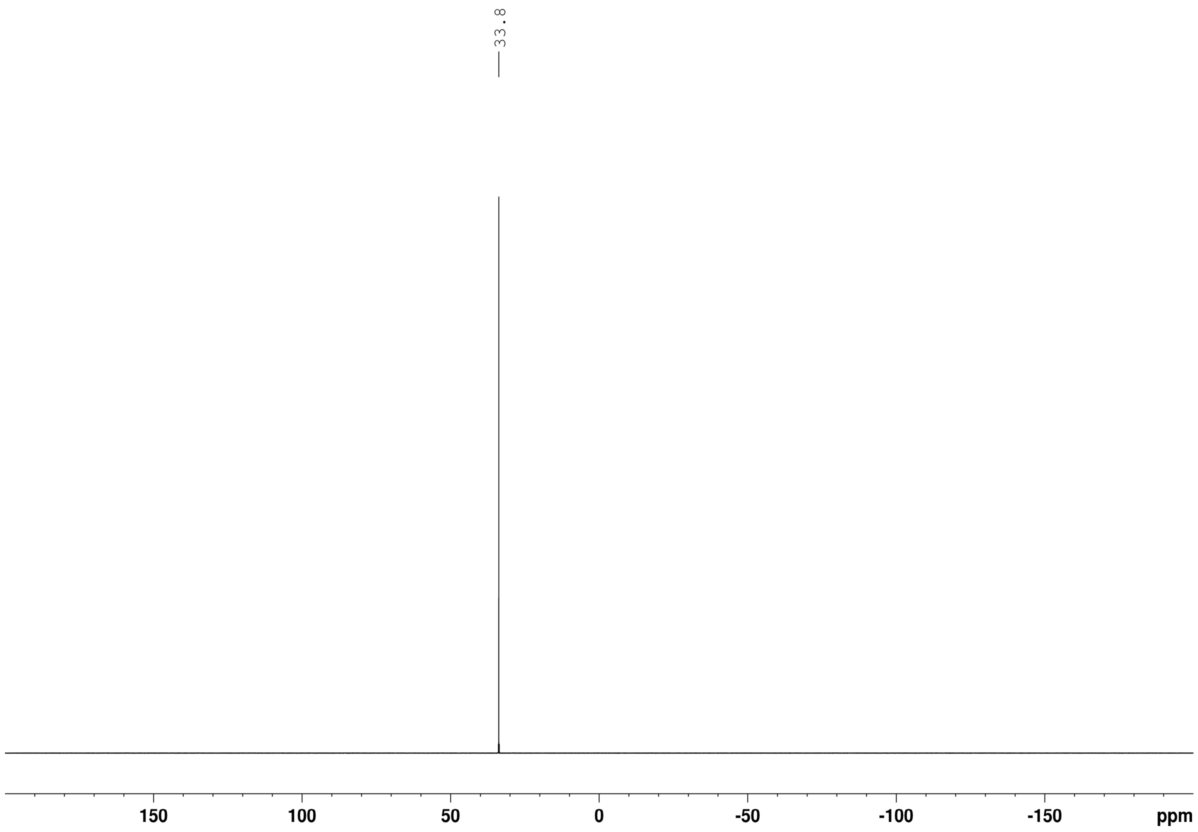

^{31}P NMR spectrum (162 MHz, DMSO-d_6) of **16**.

Tetrabutylphosphonium triazolate (17)

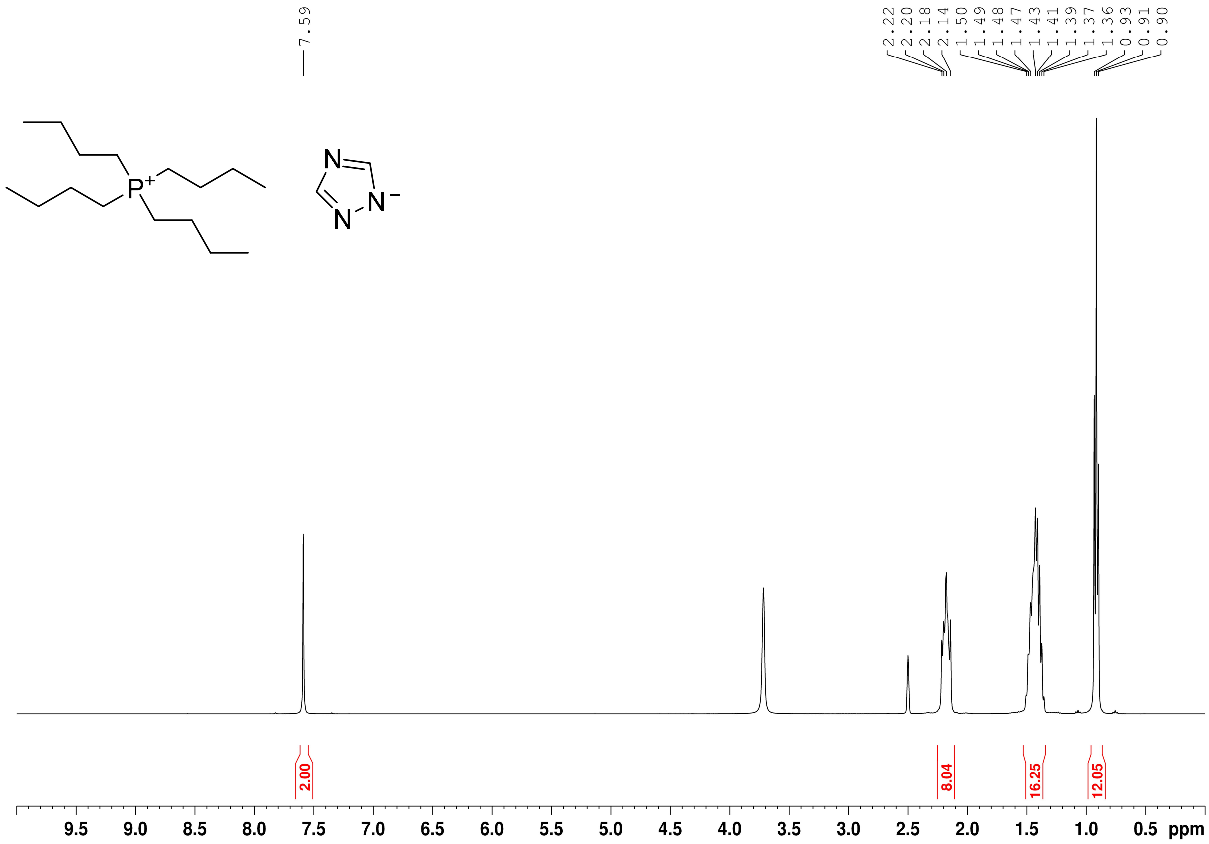

^{1}H NMR spectrum (400 MHz, DMSO-d_6) of **17**.

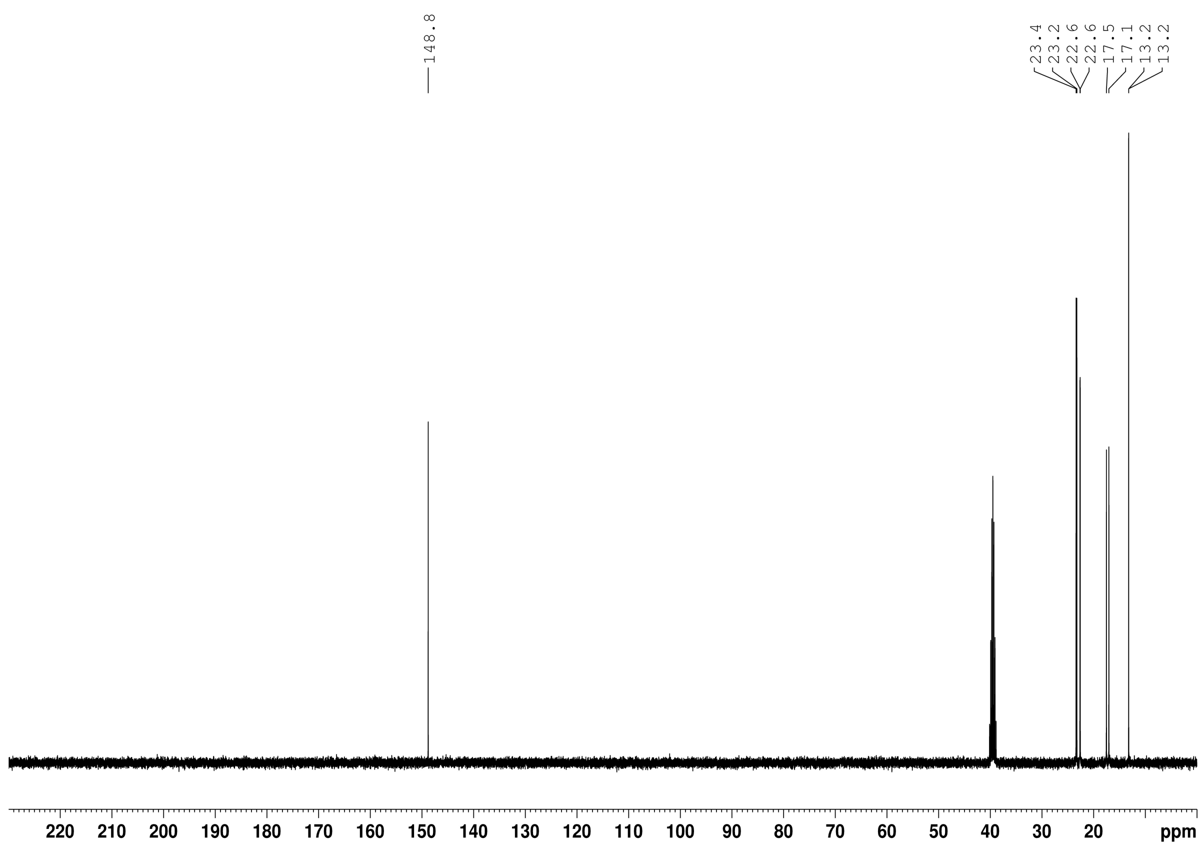

^{13}C NMR spectrum (100 MHz, DMSO-d_6) of **17**.

Tetrabutylphosphonium benzoate (18)

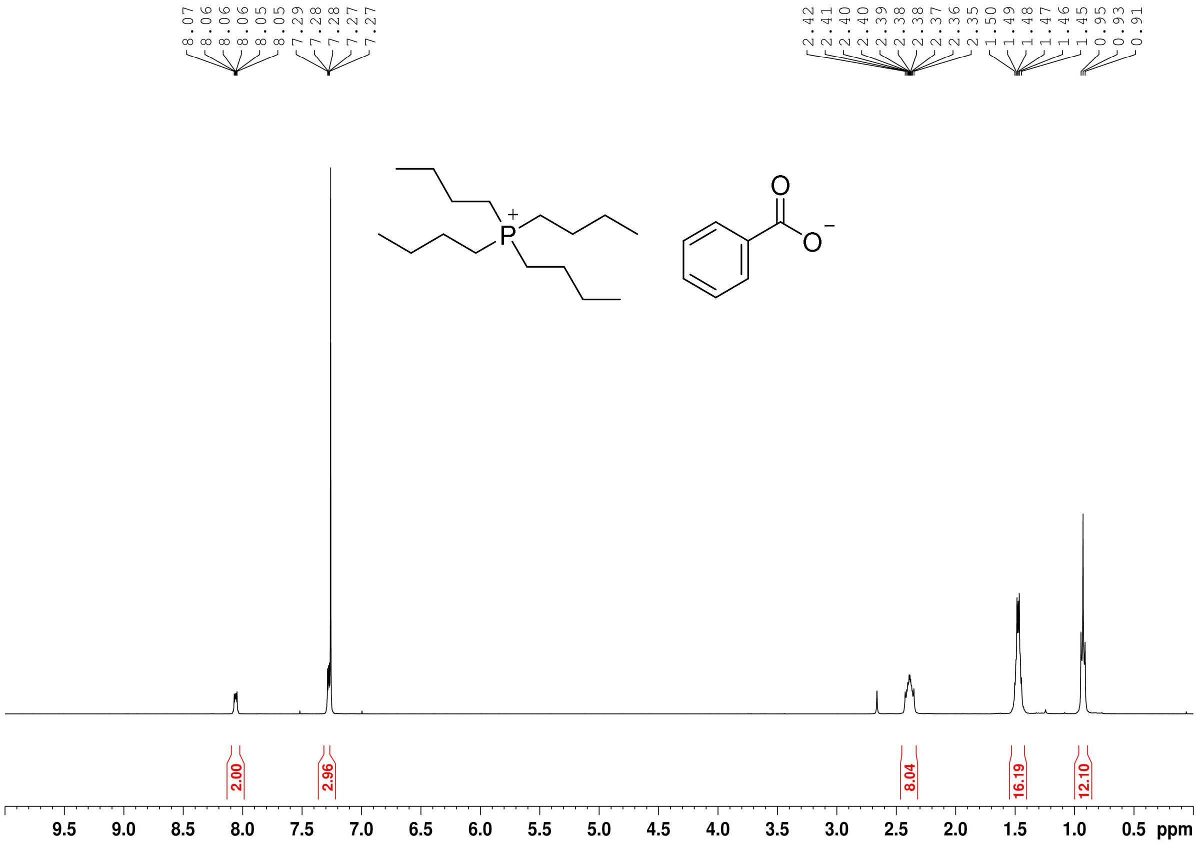

^{1}H NMR spectrum (400 MHz, $CDCl_3$) of **18**.

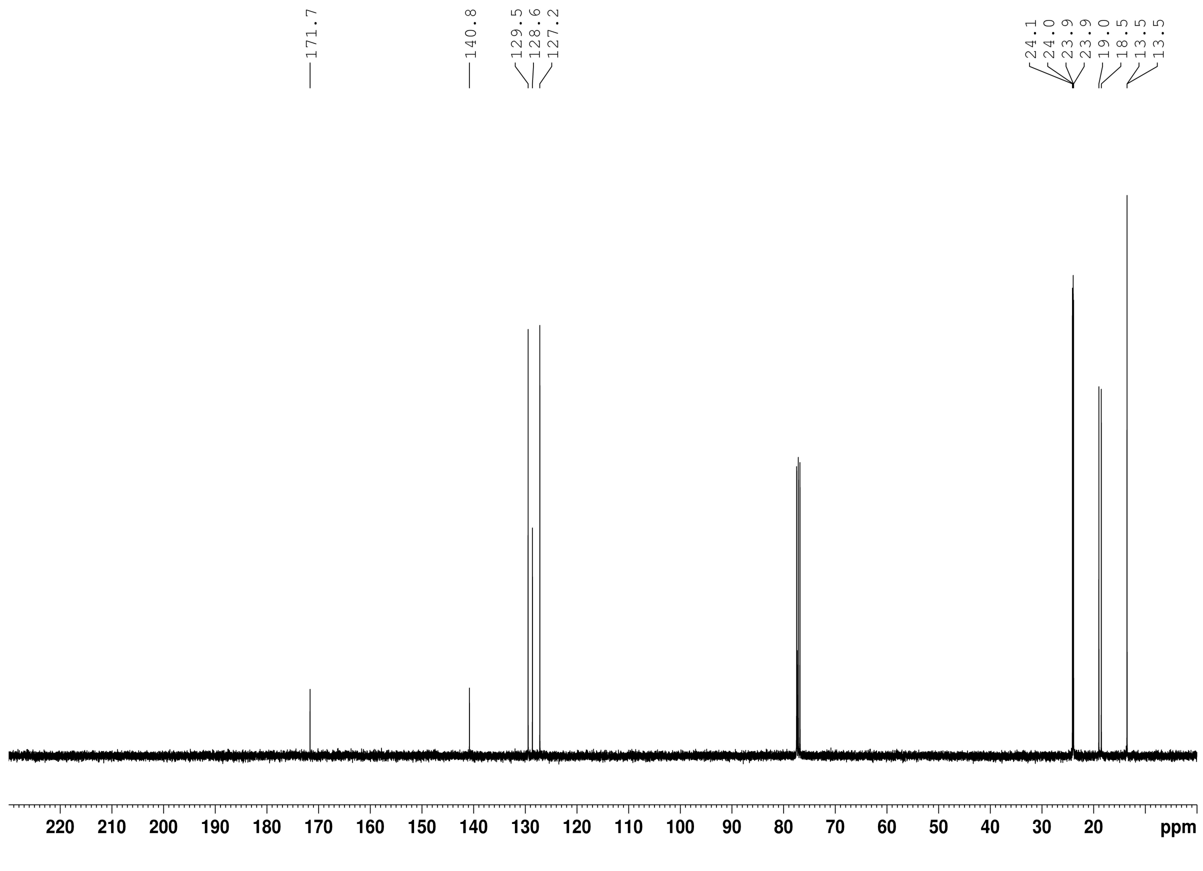

^{13}C NMR spectrum (100 MHz, $CDCl_3$) of **18**.

Tetrabutylphosphonium 4-methoxybenzoate (19)

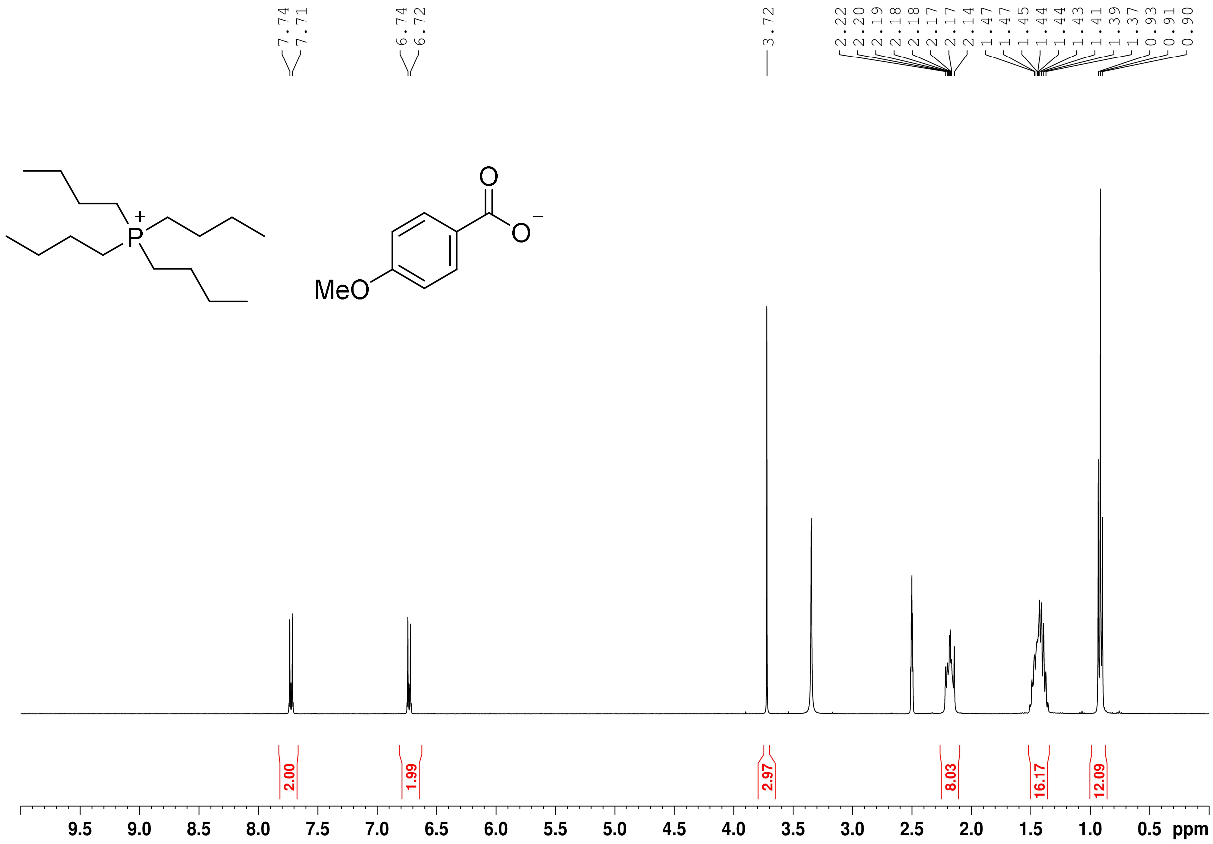

^{1}H NMR spectrum (400 MHz, DMSO-d_6) of **19**.

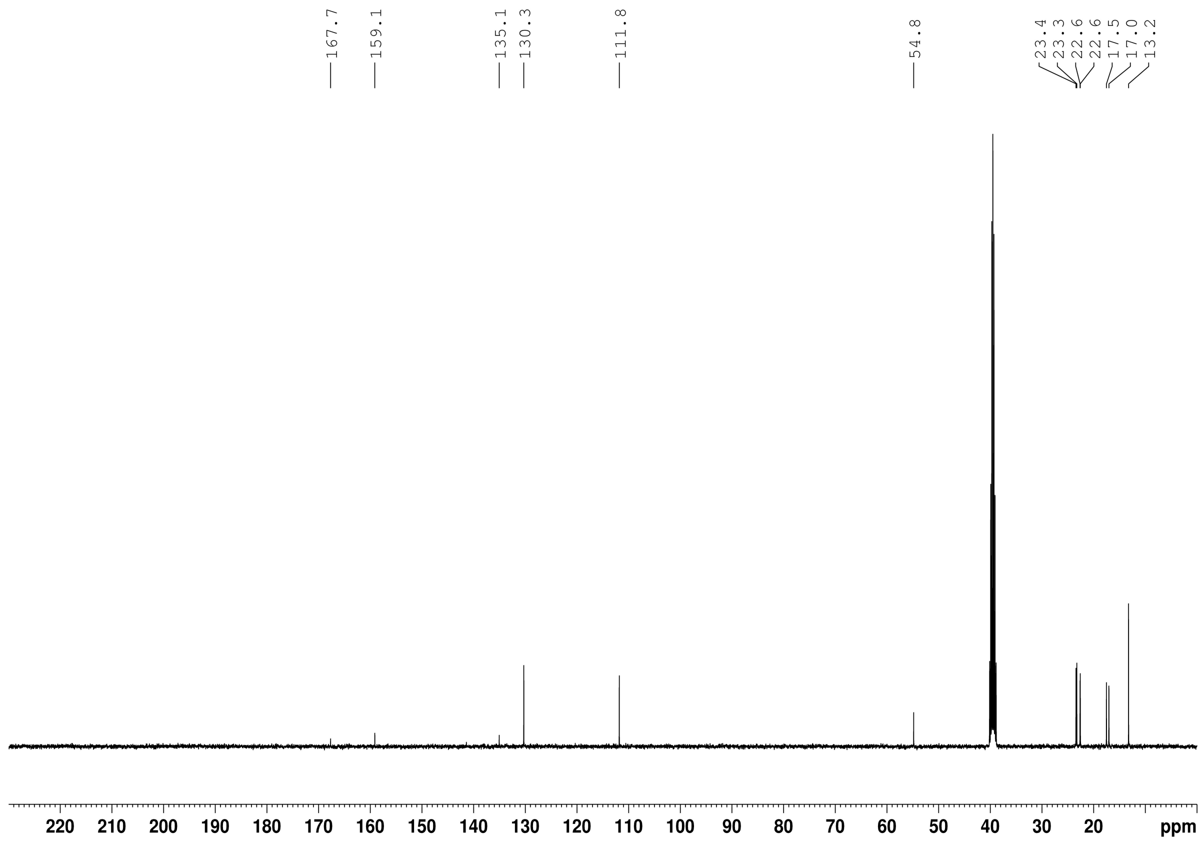

^{13}C NMR spectrum (100 MHz, DMSO-d_6) of **19**.

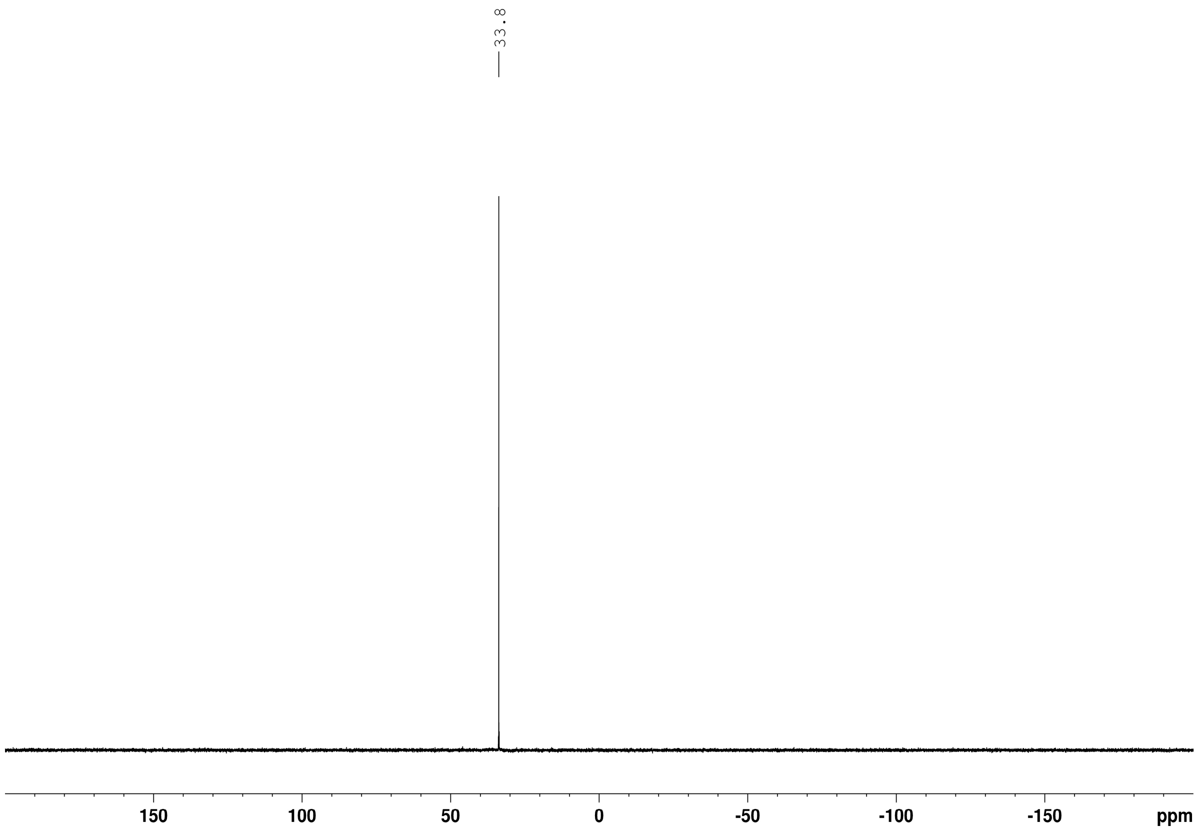

^{31}P NMR spectrum (162 MHz, DMSO-d_6) of **19**.

Tetrabutylphosphonium 4-trifluoromethylbenzoate (20)

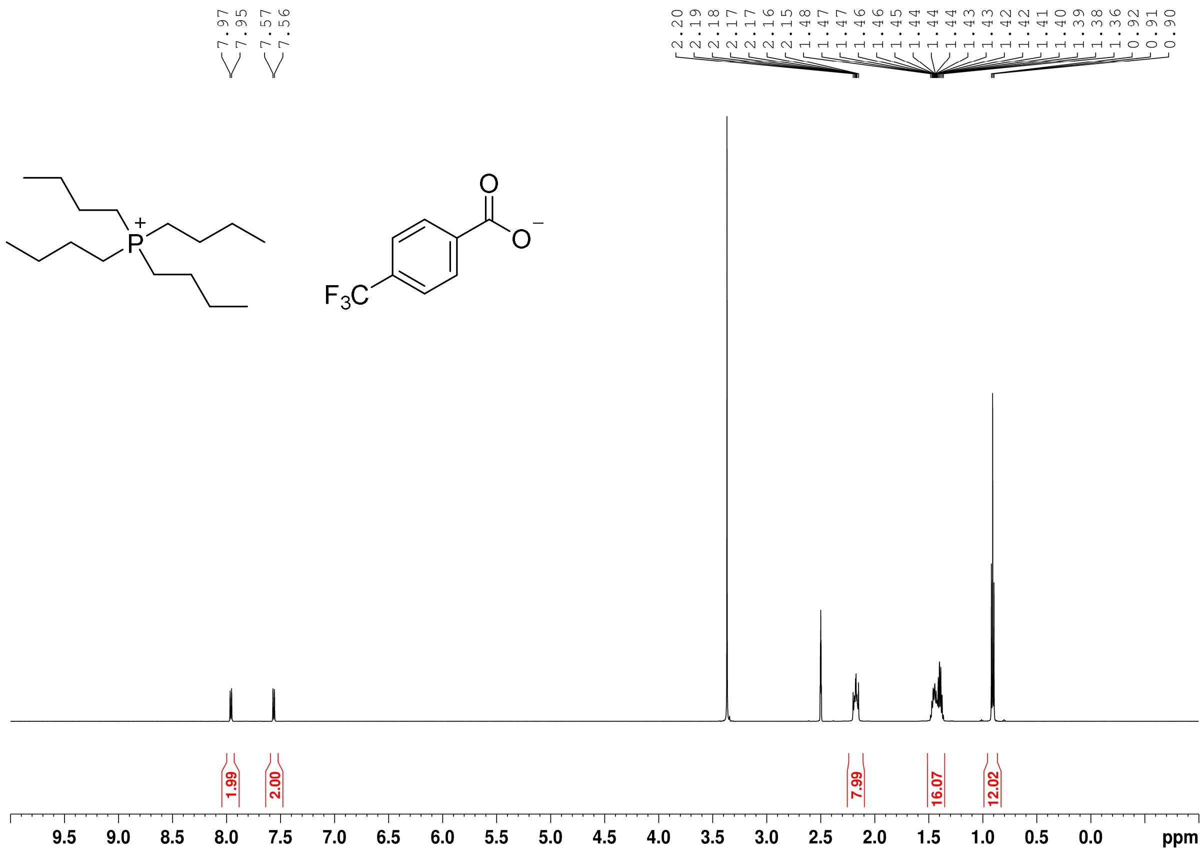

^{1}H NMR spectrum (600 MHz, DMSO-d_6) of **20**.

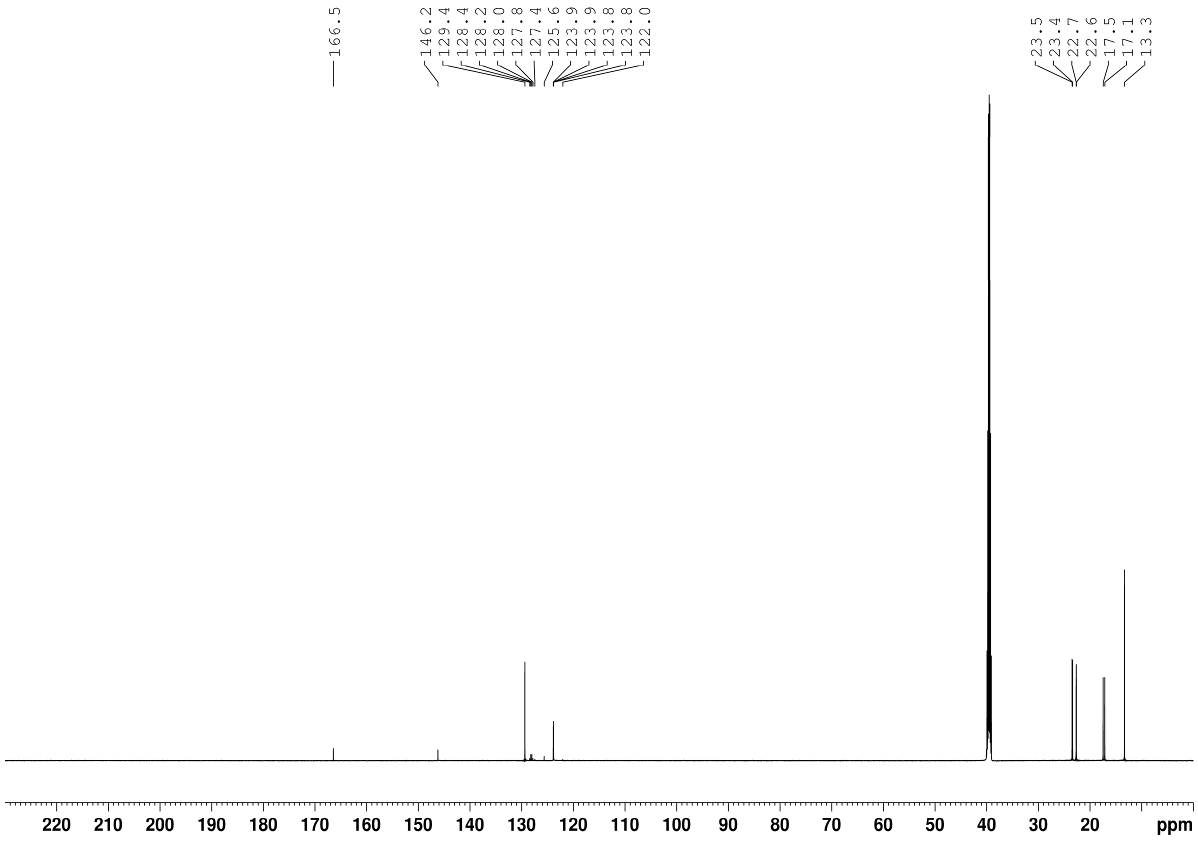

^{13}C NMR spectrum (150 MHz, DMSO-d_6) of **20**.

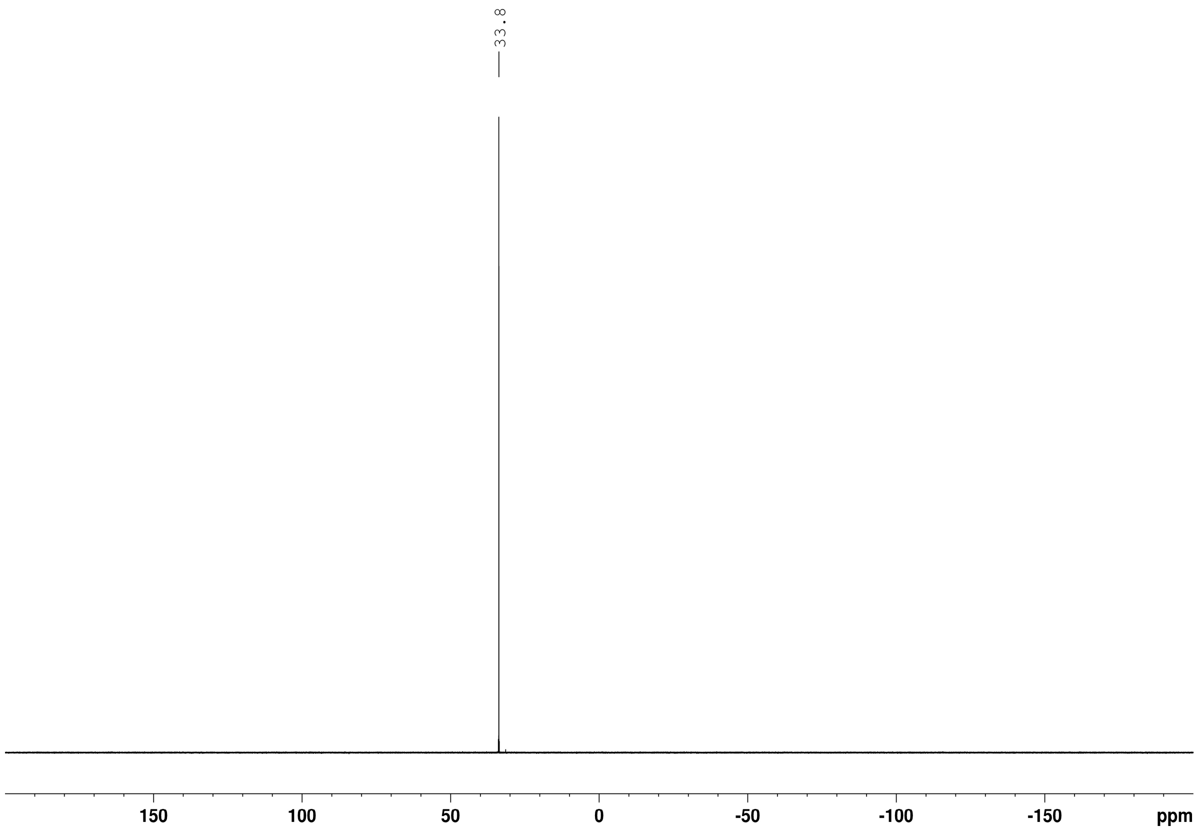

^{31}P NMR spectrum (162 MHz, DMSO-d_6) of **20**.

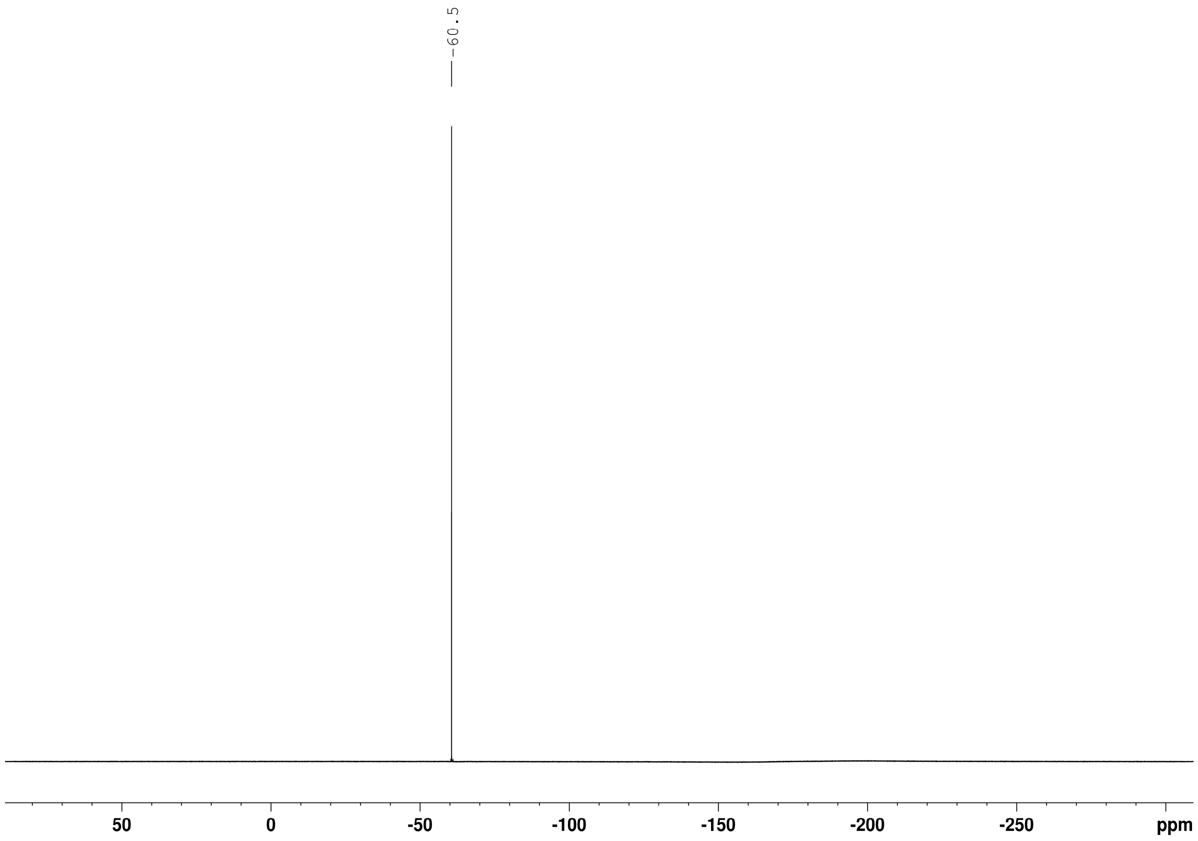

^{19}F NMR spectrum (377 MHz, DMSO-d_6) of **20**.

Tetrabutylphosphonium cinnamate (21)

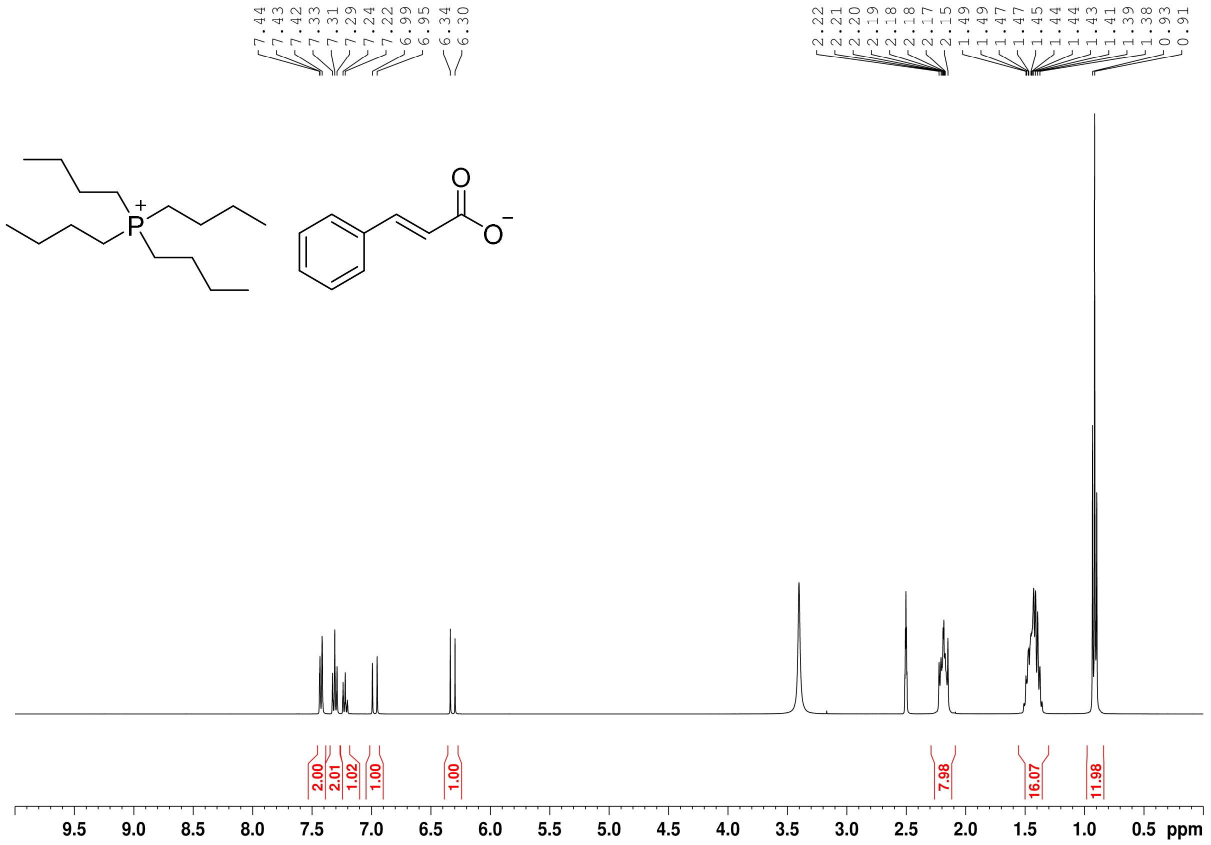

^{1}H NMR spectrum (400 MHz, DMSO-d_6) of **21**.

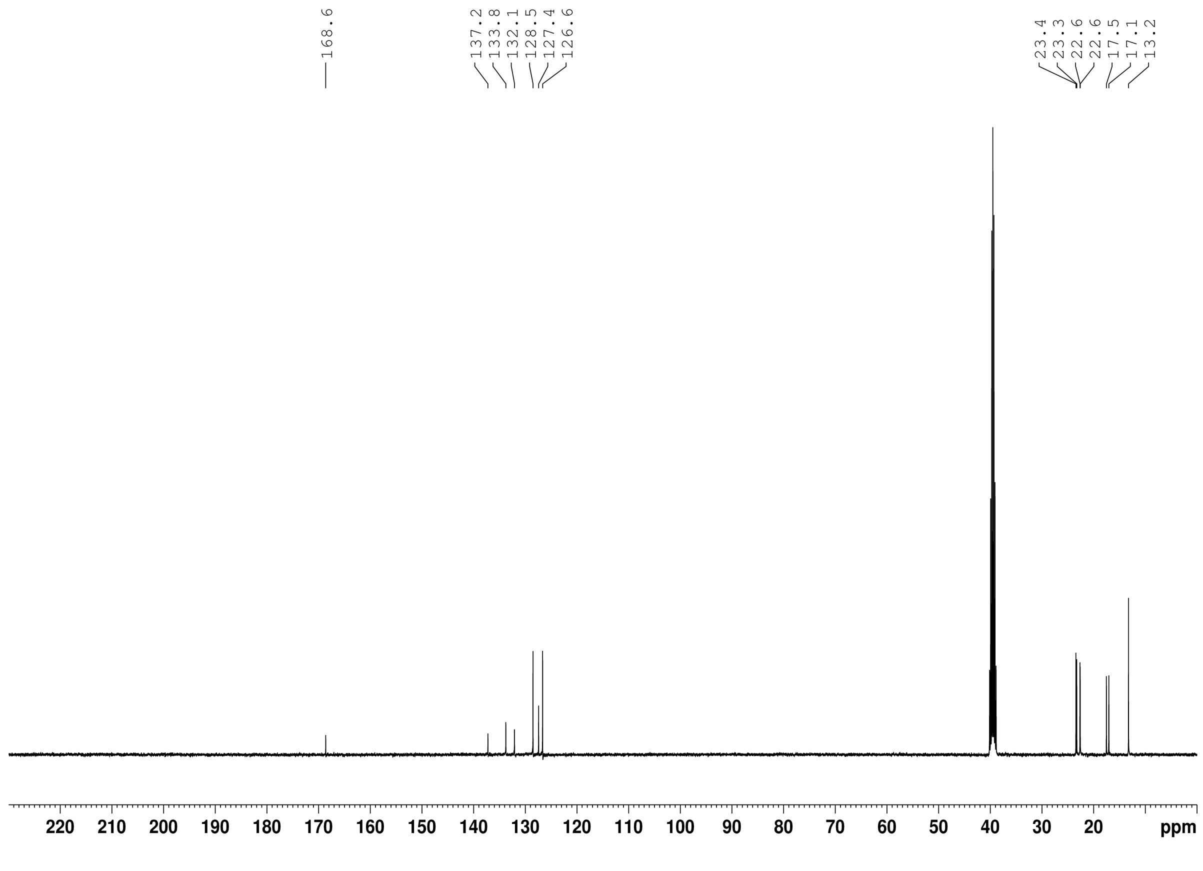

^{13}C NMR spectrum (100 MHz, DMSO-d_6) of **21**.

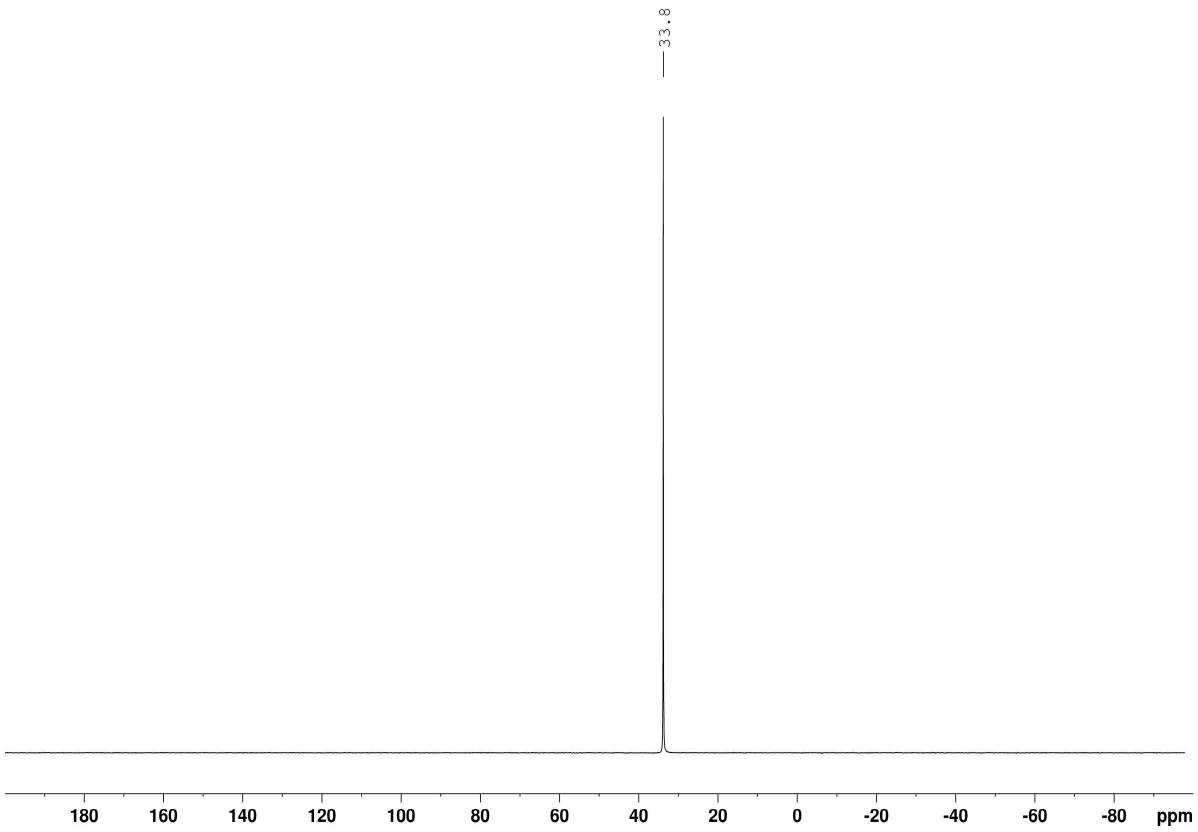

^{31}P NMR spectrum (162 MHz, DMSO-d_6) of **21**.

Tetrabutylphosphonium 2-naphthoate (22)

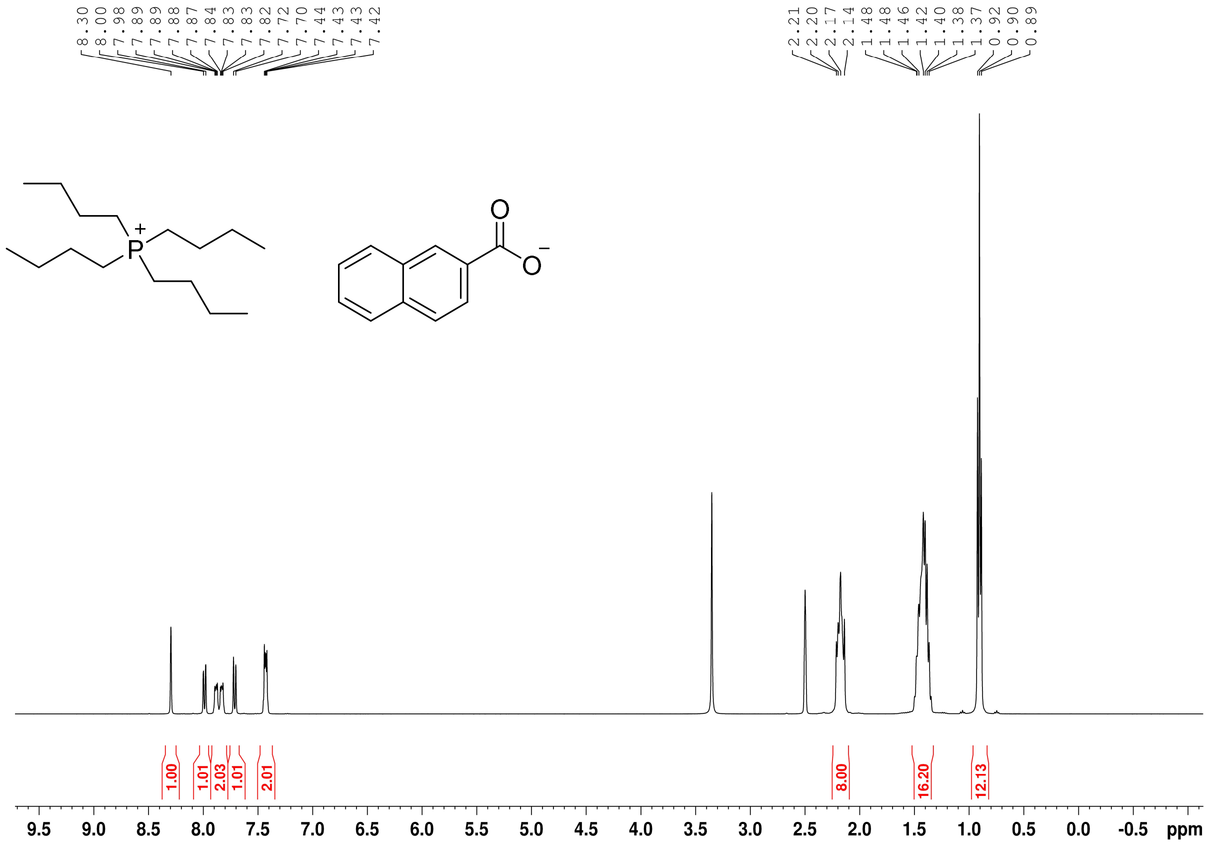

^{1}H NMR spectrum (400 MHz, DMSO-d_6) of **22**.

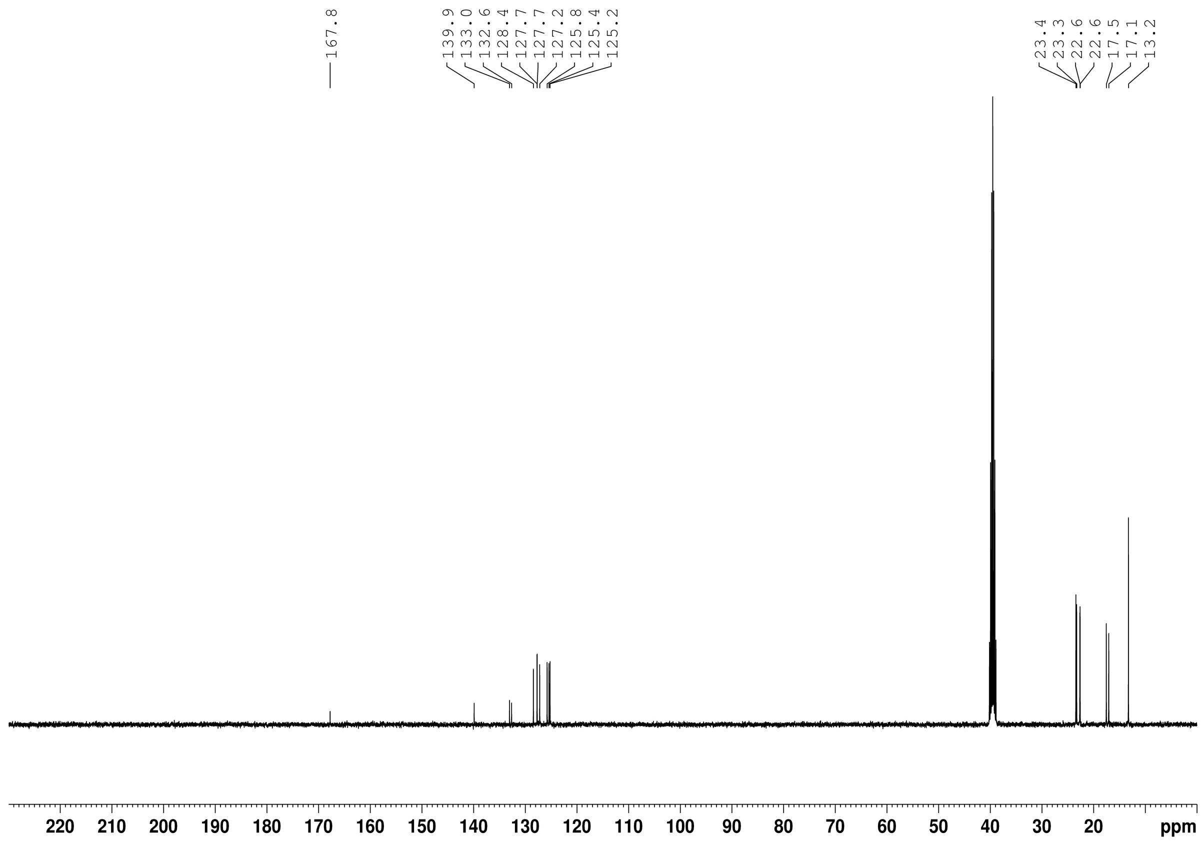

^{13}C NMR spectrum (100 MHz, DMSO-d_6) of **22**.

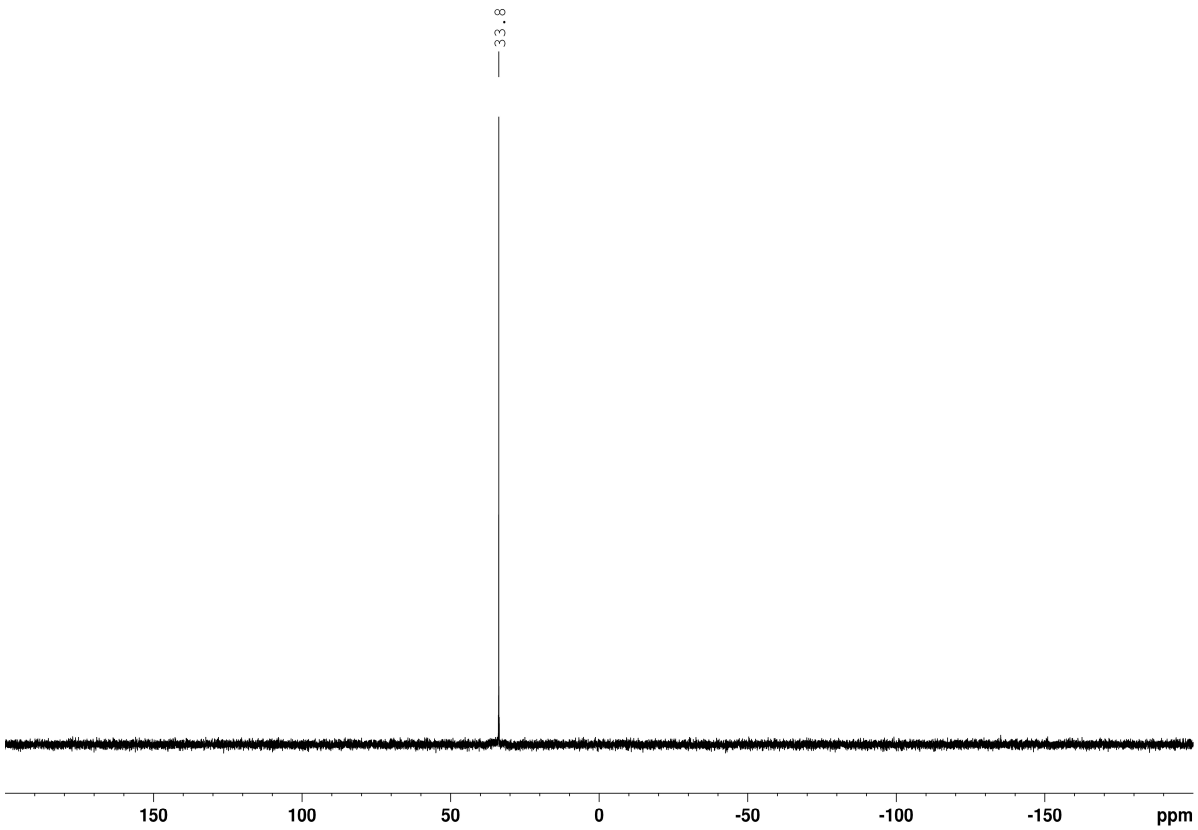

^{31}P NMR spectrum (162 MHz, DMSO-d_6) of **22**.

Tetrabutylphosphonium salicylate (23)

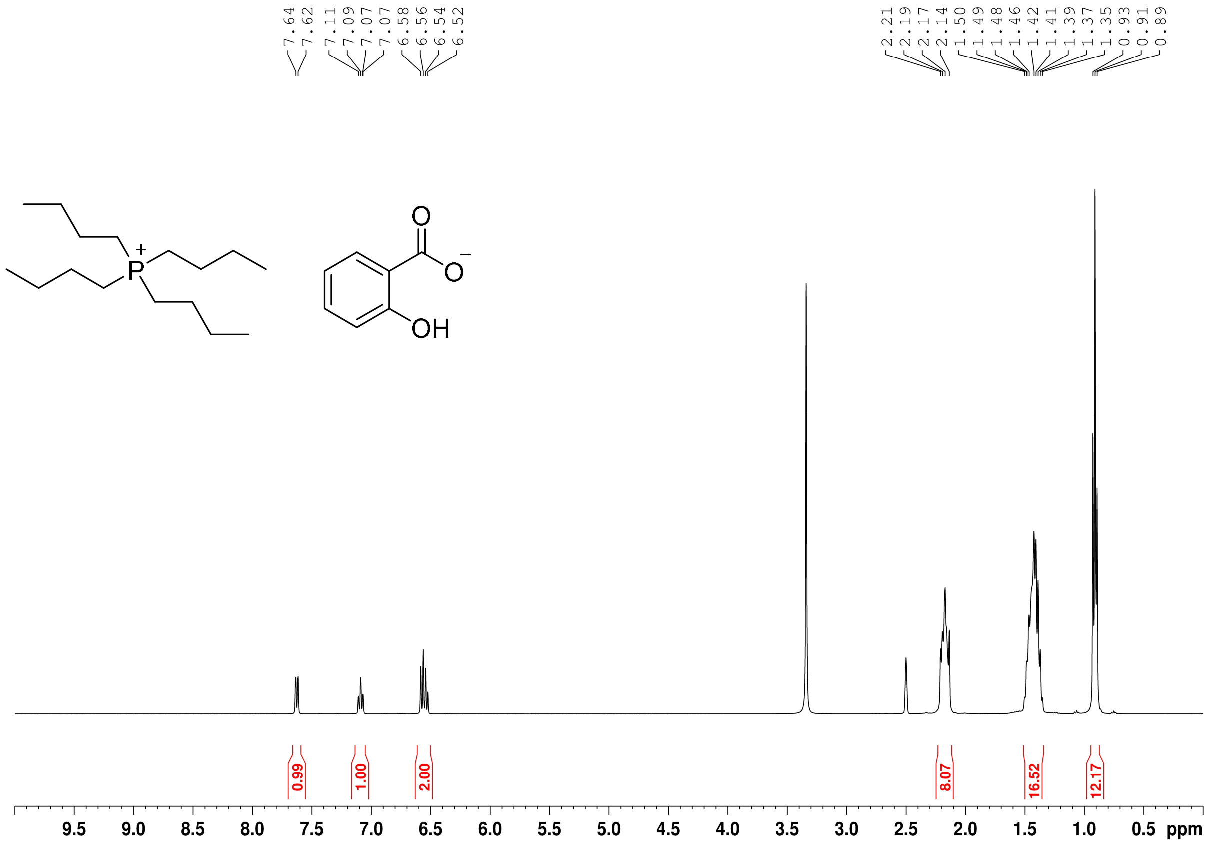

^{1}H NMR spectrum (400 MHz, DMSO-d_6) of **23**.

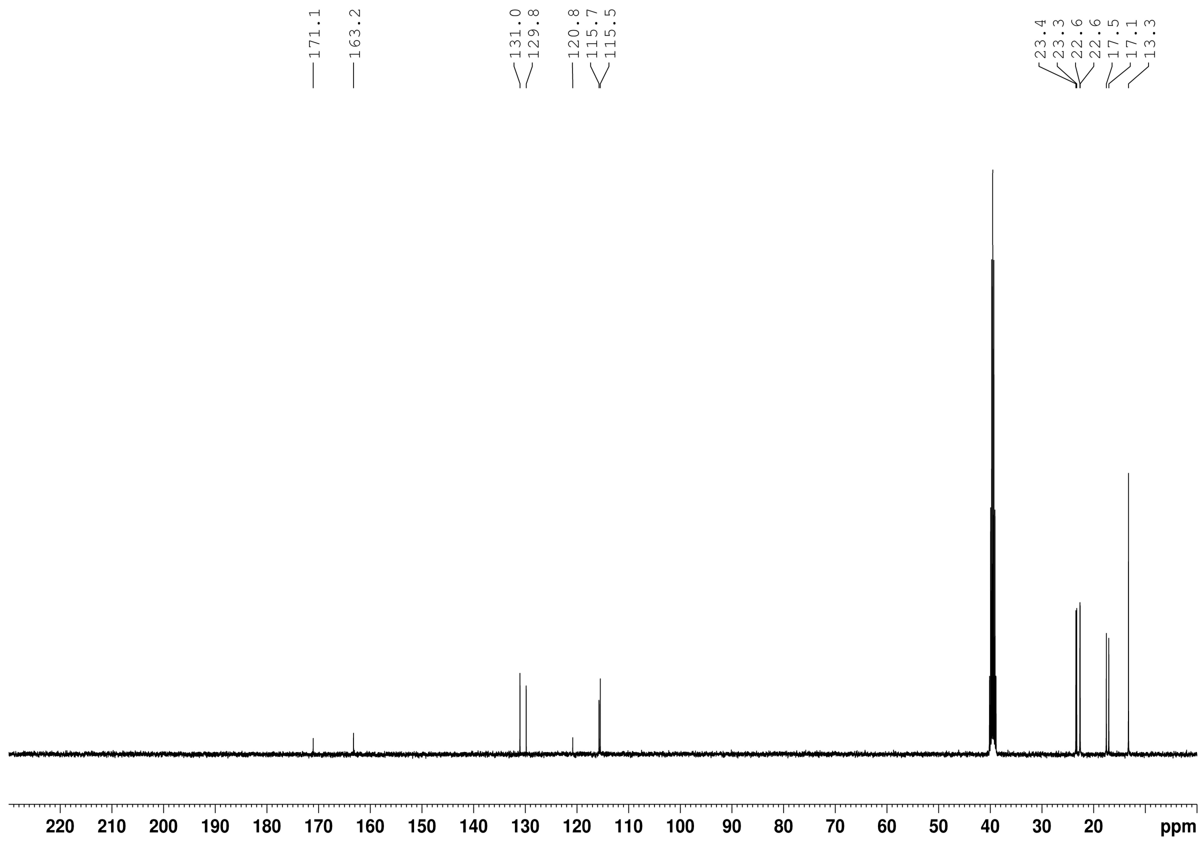

^{13}C NMR spectrum (100 MHz, DMSO-d_6) of **23**.

Tetrabutylphosphonium difluoroacetate (24)

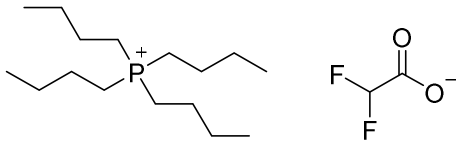

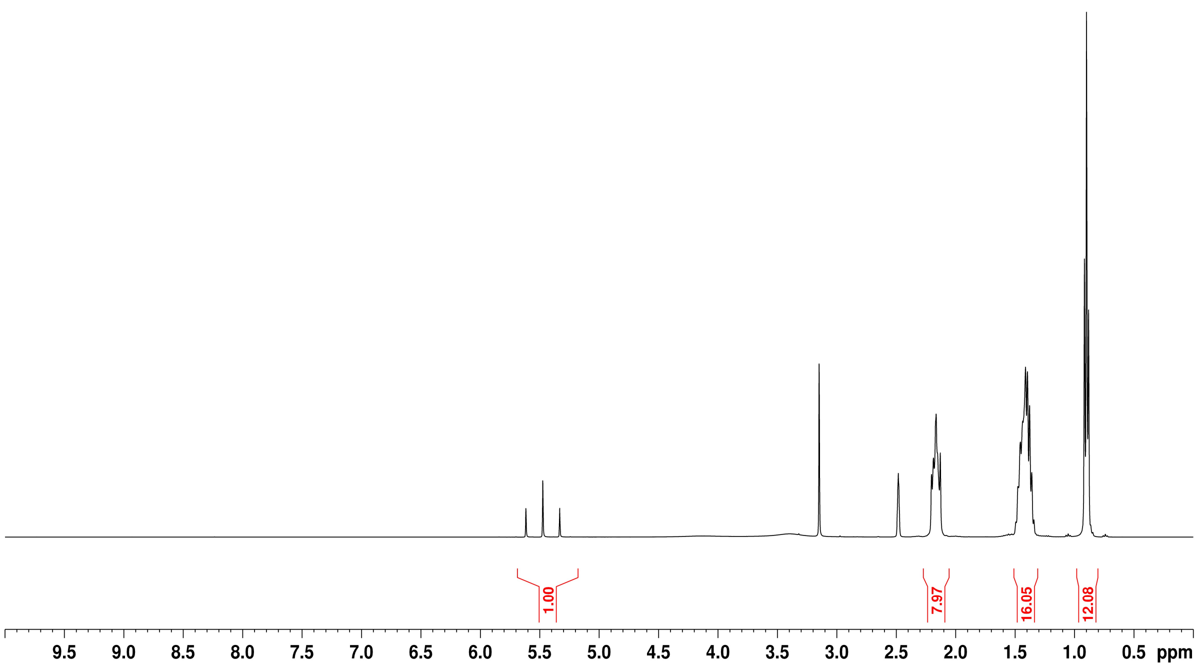

^{1}H NMR spectrum (400 MHz, DMSO-d_6) of **24**.

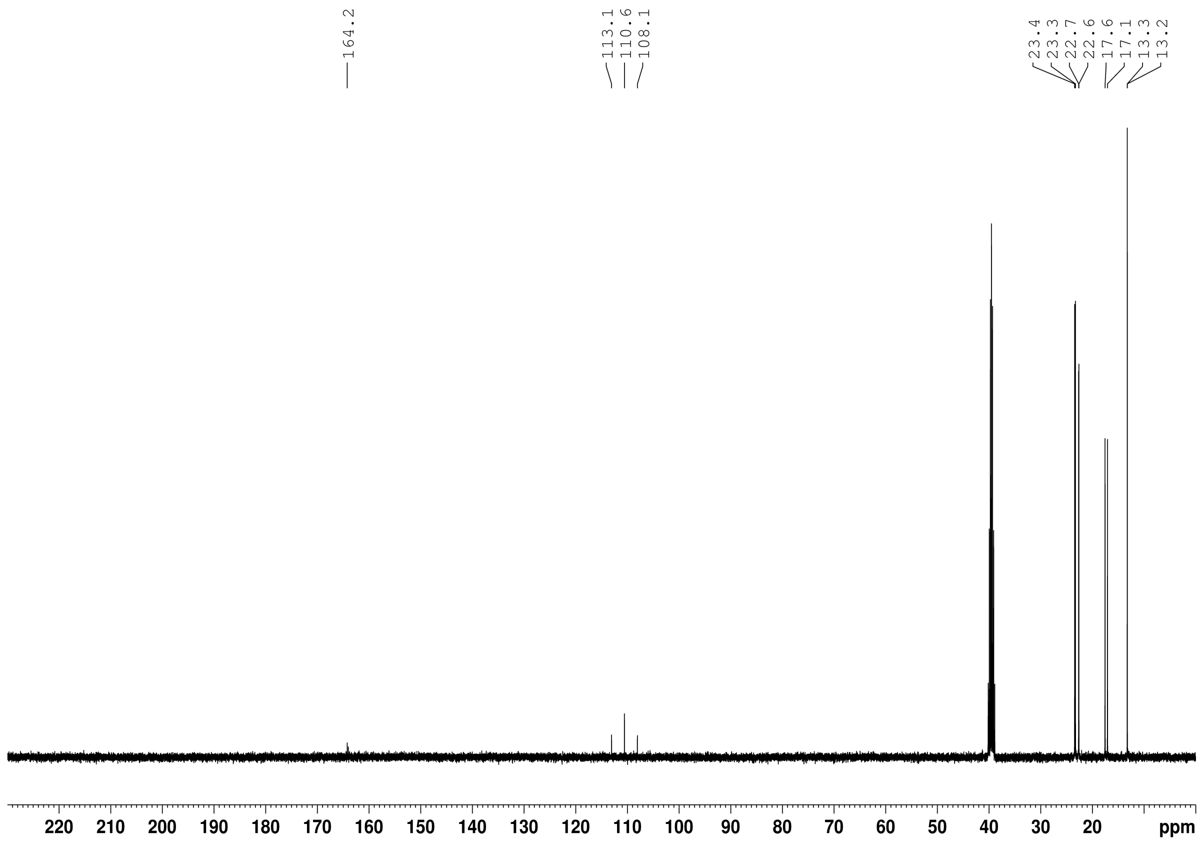

^{13}C NMR spectrum (100 MHz, DMSO-d_6) of **24**.

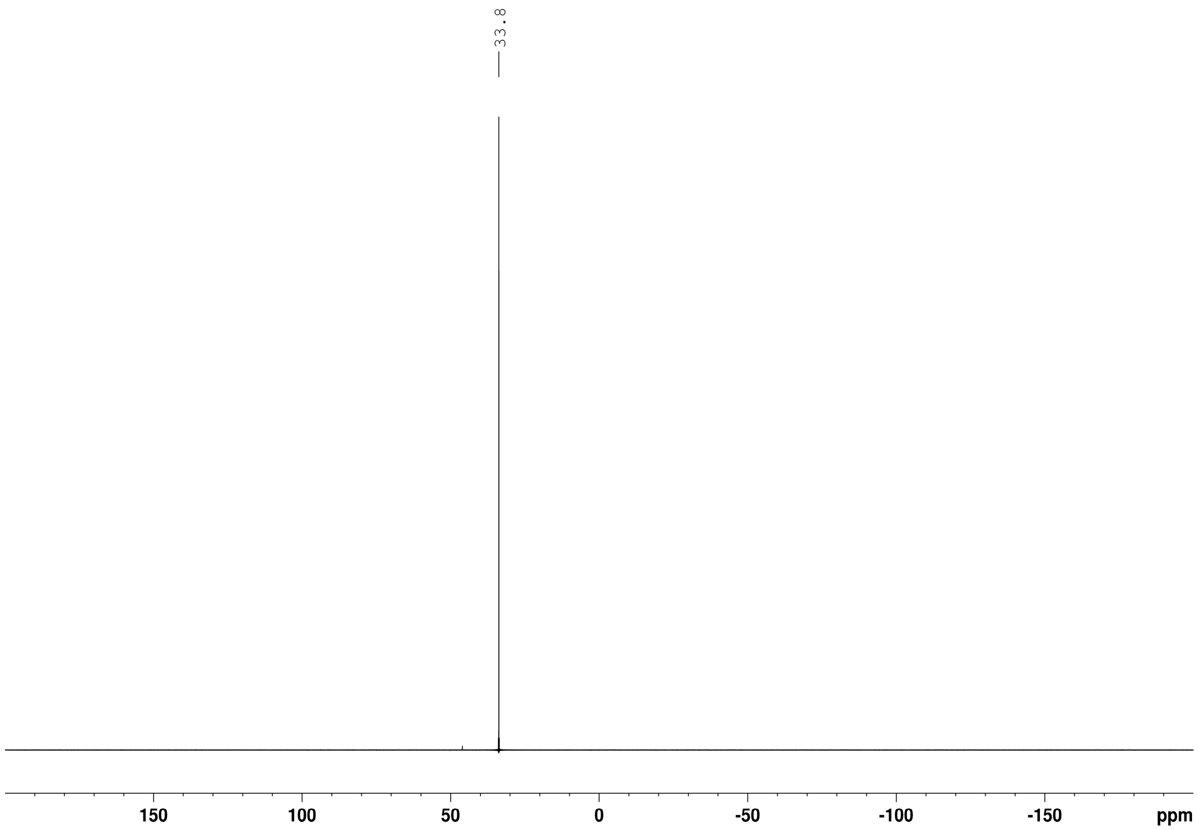

^{31}P NMR spectrum (162 MHz, DMSO-d_6) of **24**.

Tetrabutylphosphonium pivalate (25)

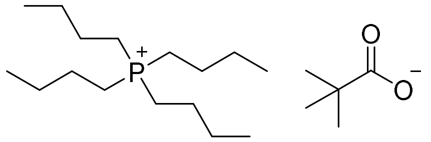

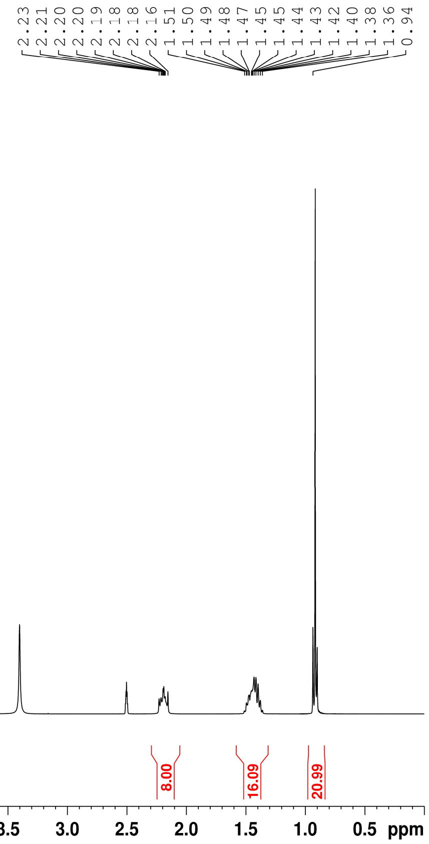

^{1}H NMR spectrum (400 MHz, DMSO-d_6) of **25**.

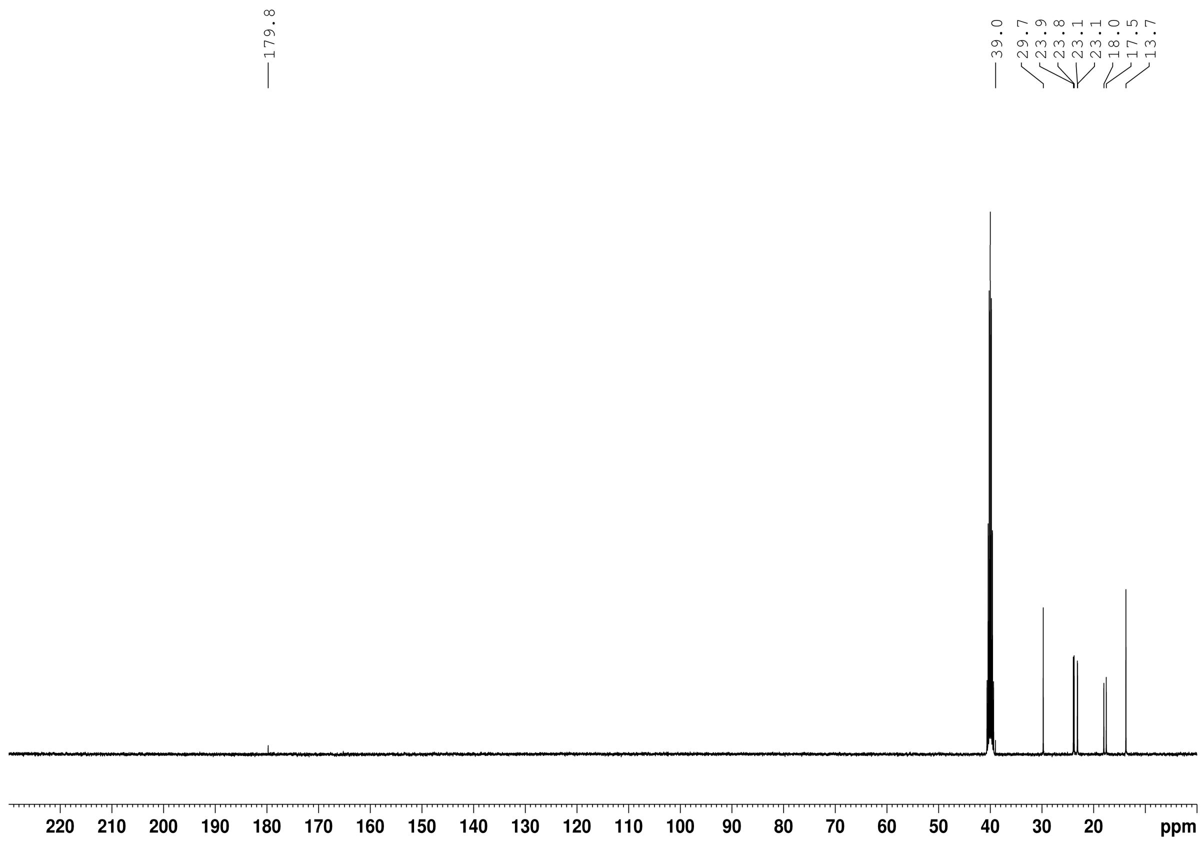

^{13}C NMR spectrum (100 MHz, DMSO-d_6) of **25**.

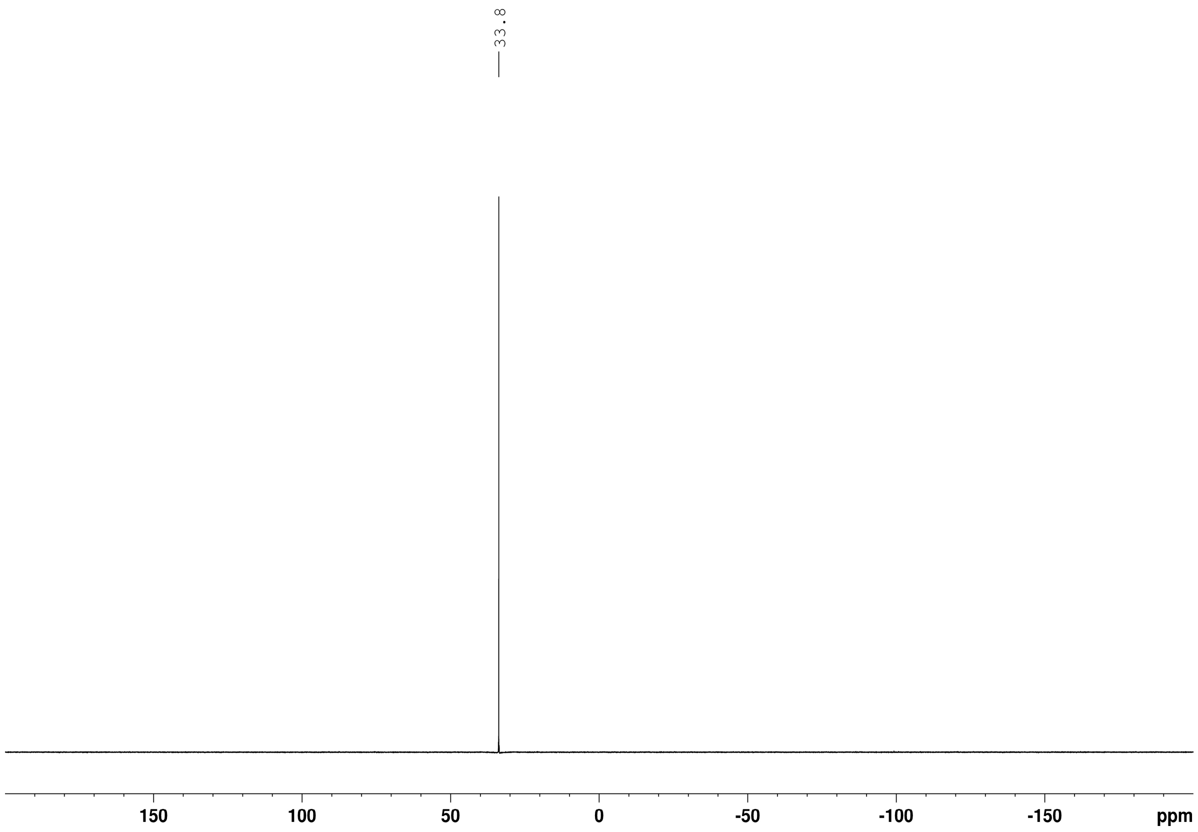

^{31}P NMR spectrum (162 MHz, DMSO-d_6) of **25**.

Tetrabutylphosphonium stearate (26)

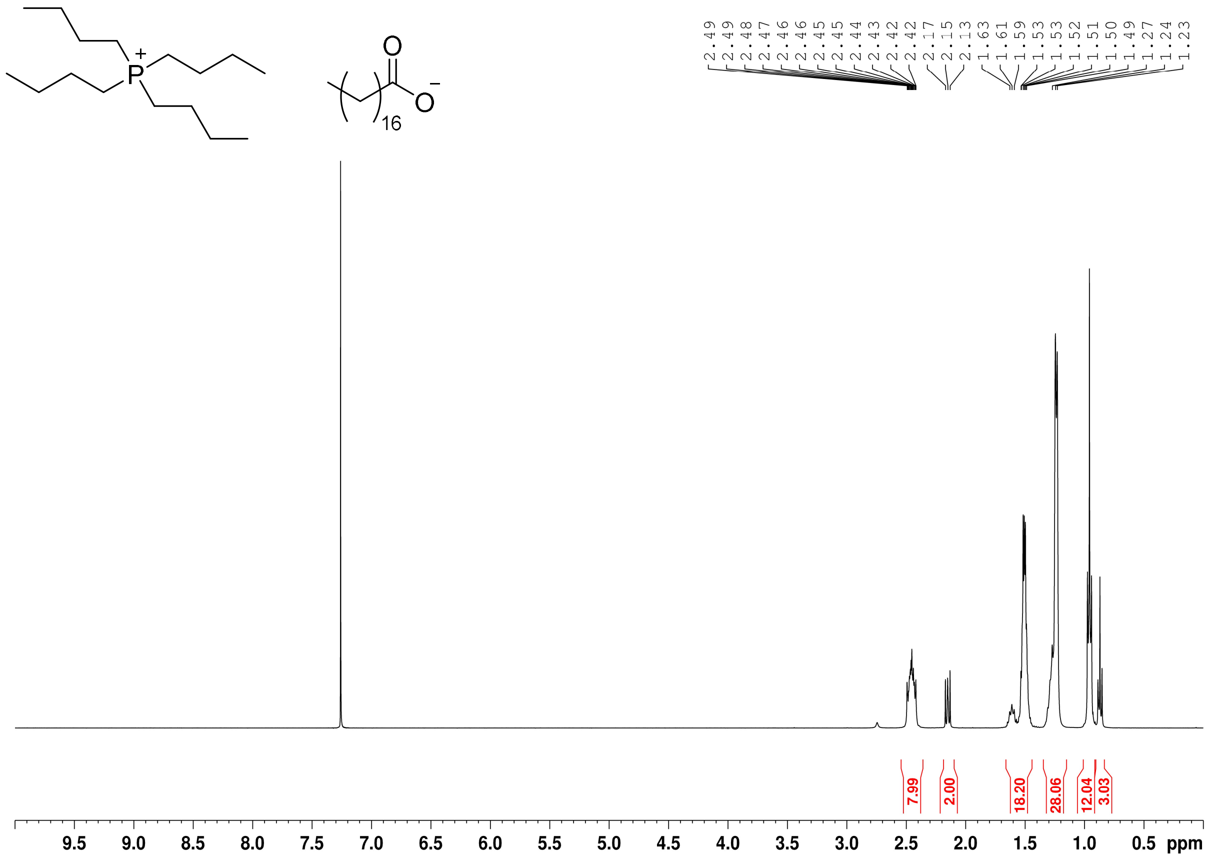

^{1}H NMR spectrum (400 MHz, $CDCl_3$) of **26**.

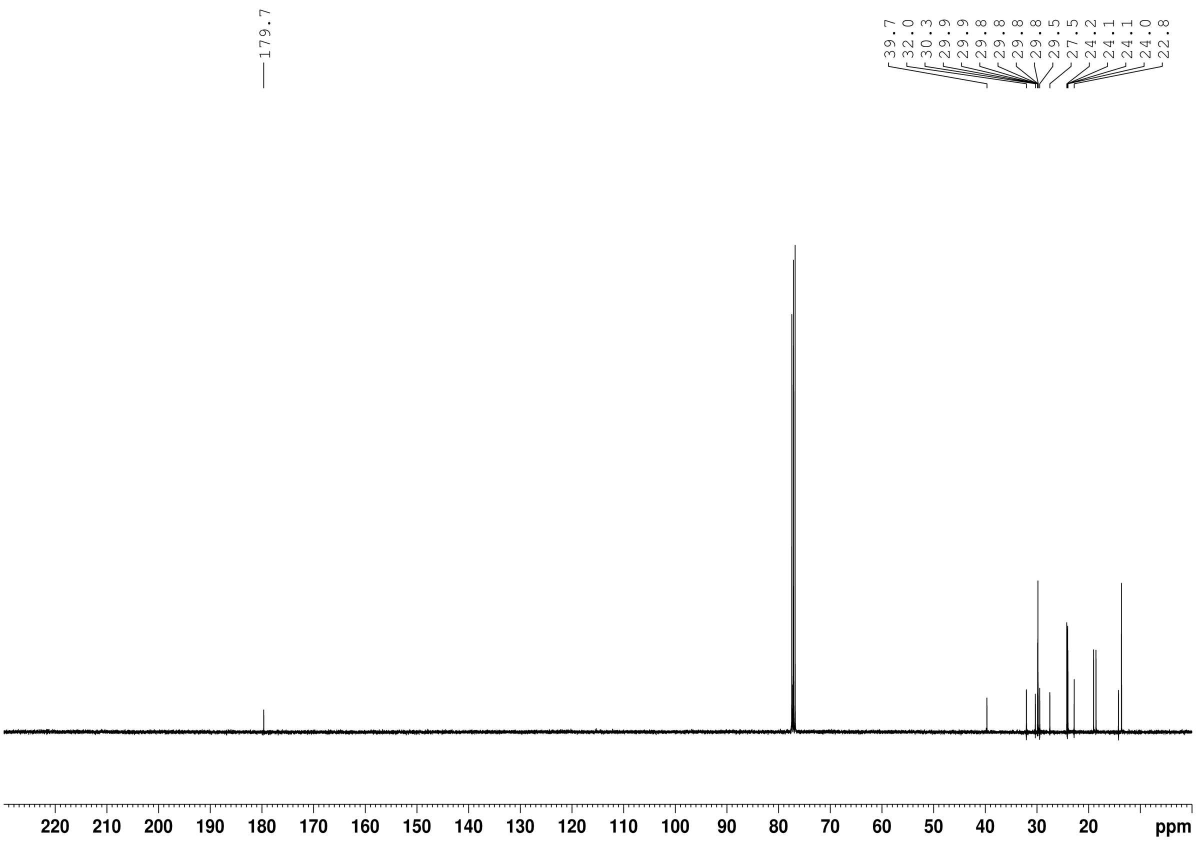

^{13}C NMR spectrum (100 MHz, $CDCl_3$) of **26**.

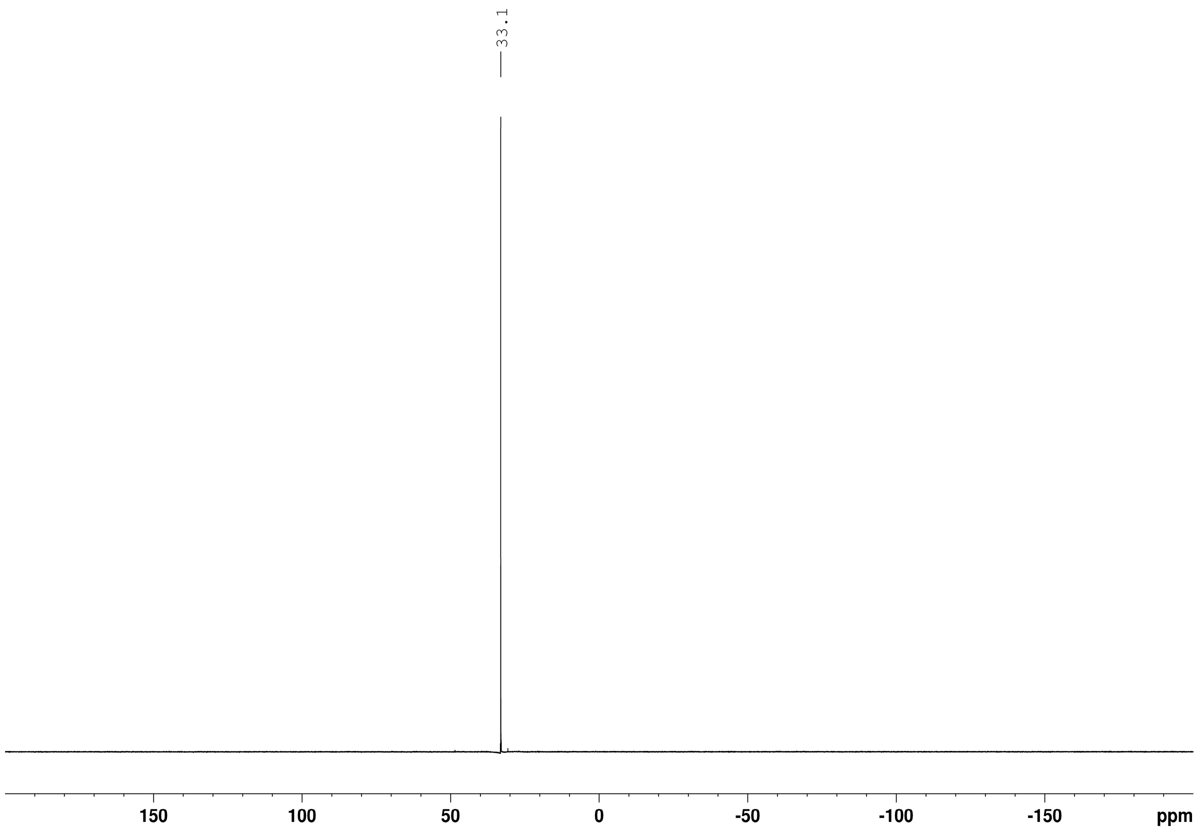

^{31}P NMR spectrum (162 MHz, $CDCl_3$) of **26**.

Tetrabutylphosphonium phenylacetate (27)

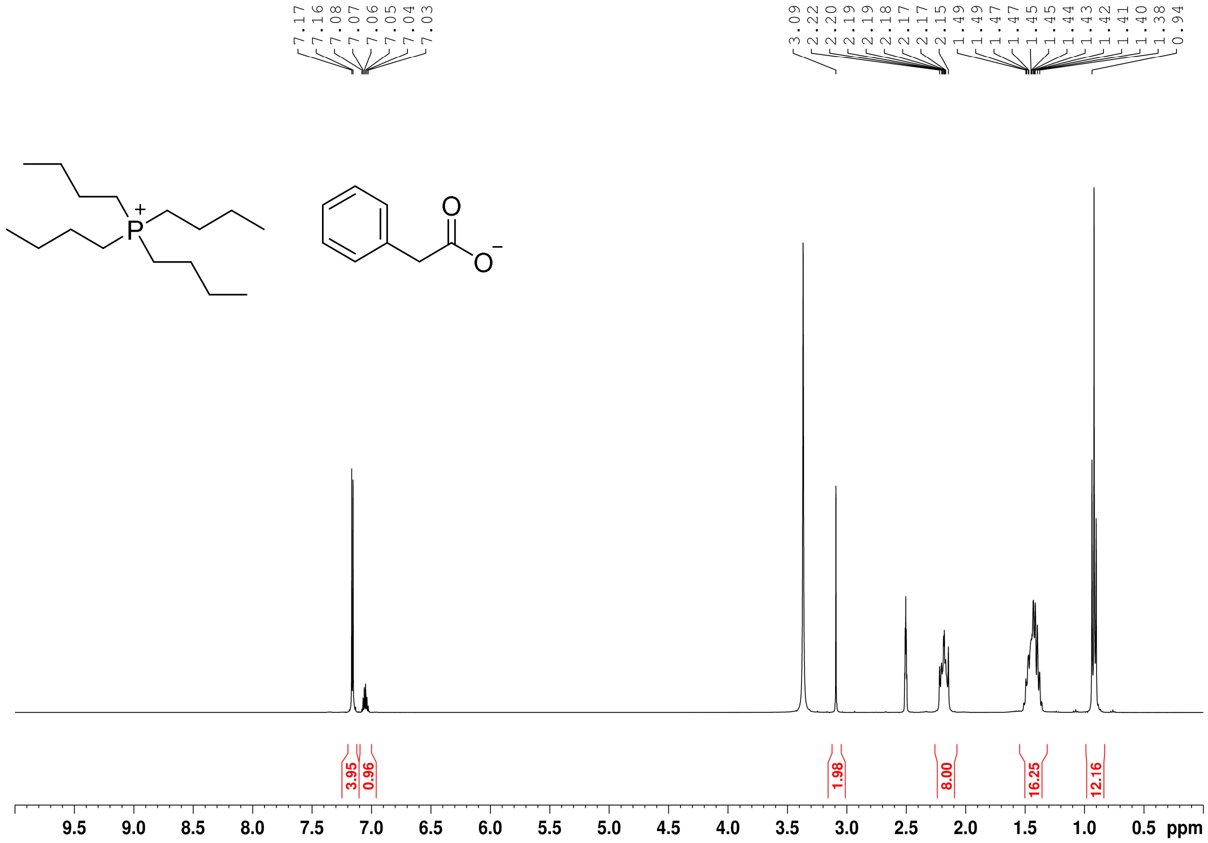

^{1}H NMR spectrum (400 MHz, DMSO-d_6) of **27.**

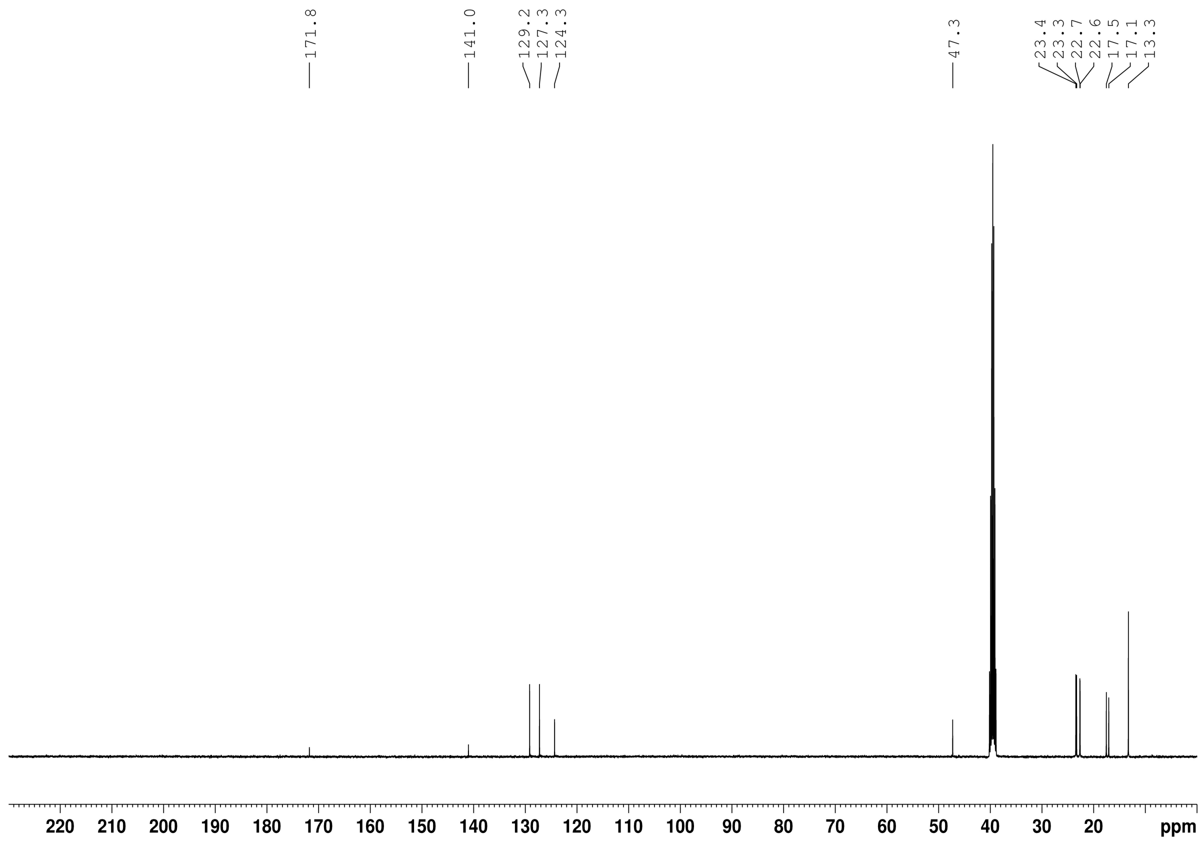

^{13}C NMR spectrum (100 MHz, DMSO-d_6) of **27**.

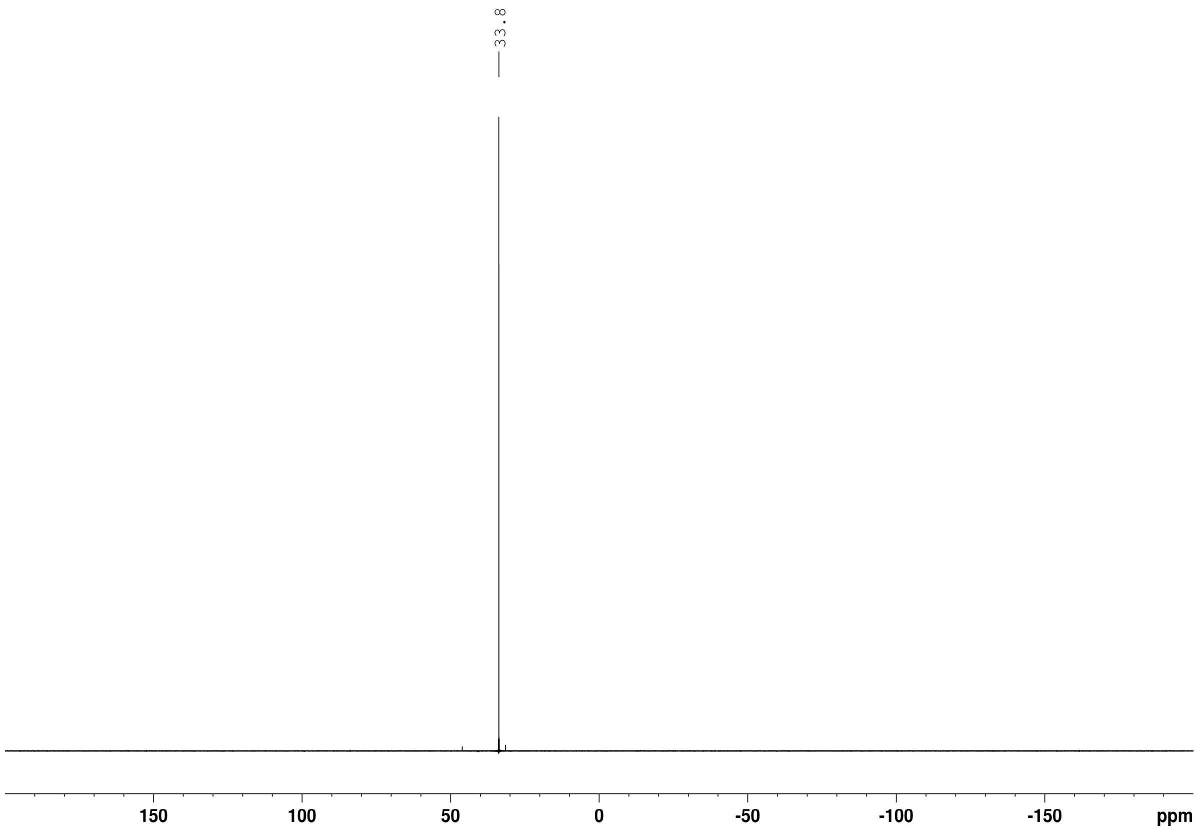

^{31}P NMR spectrum (162 MHz, DMSO-d_6) of **27**.

Tetrabutylphosphonium hydrocinnamate (28)

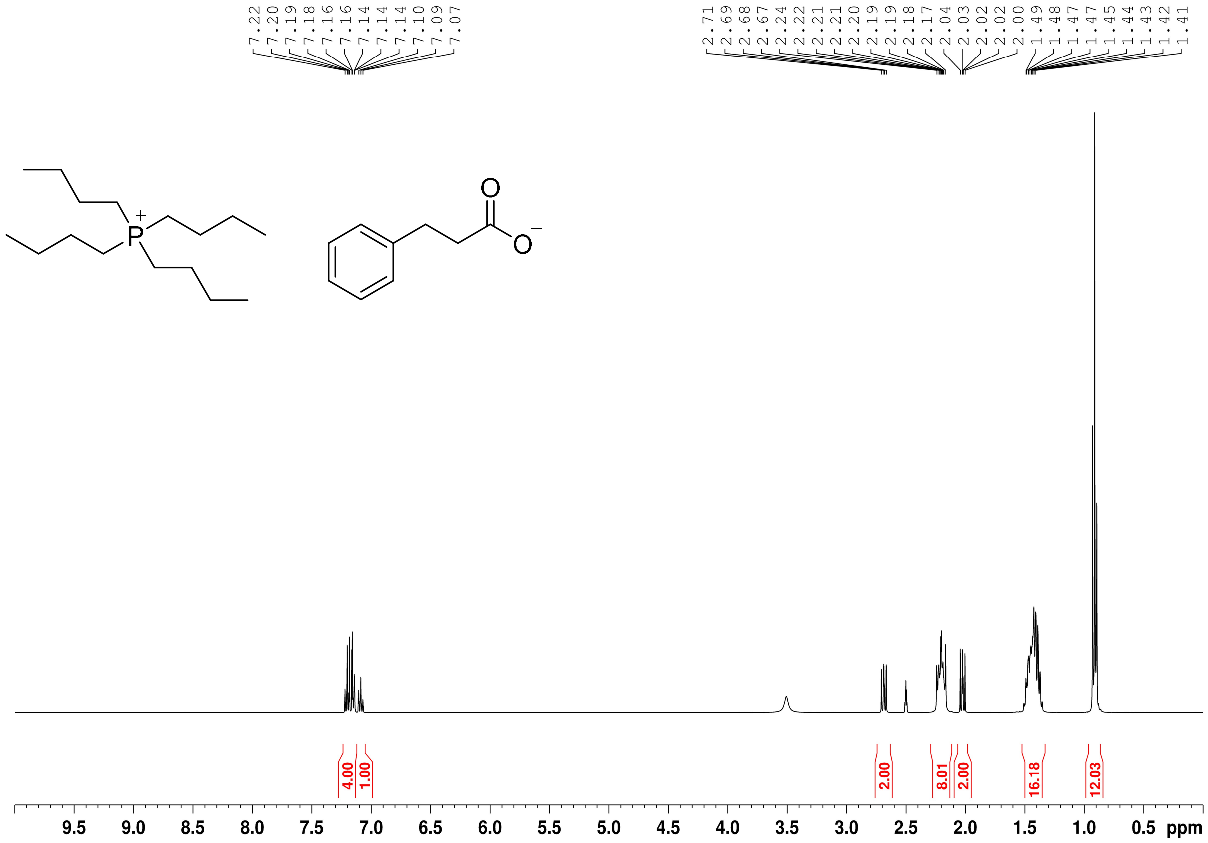

^{1}H NMR spectrum (400 MHz, DMSO-d_6) of **28**.

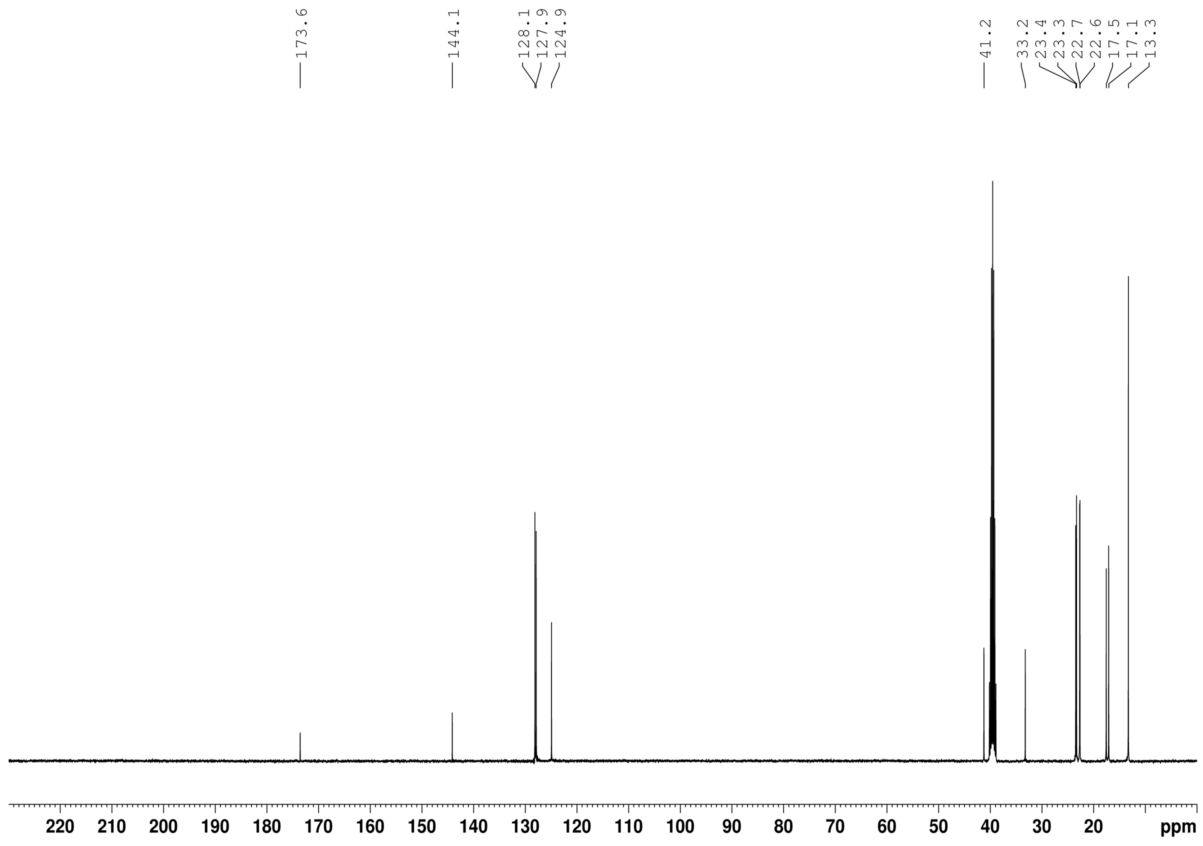

^{13}C NMR spectrum (100 MHz, DMSO-d_6) of **28**.

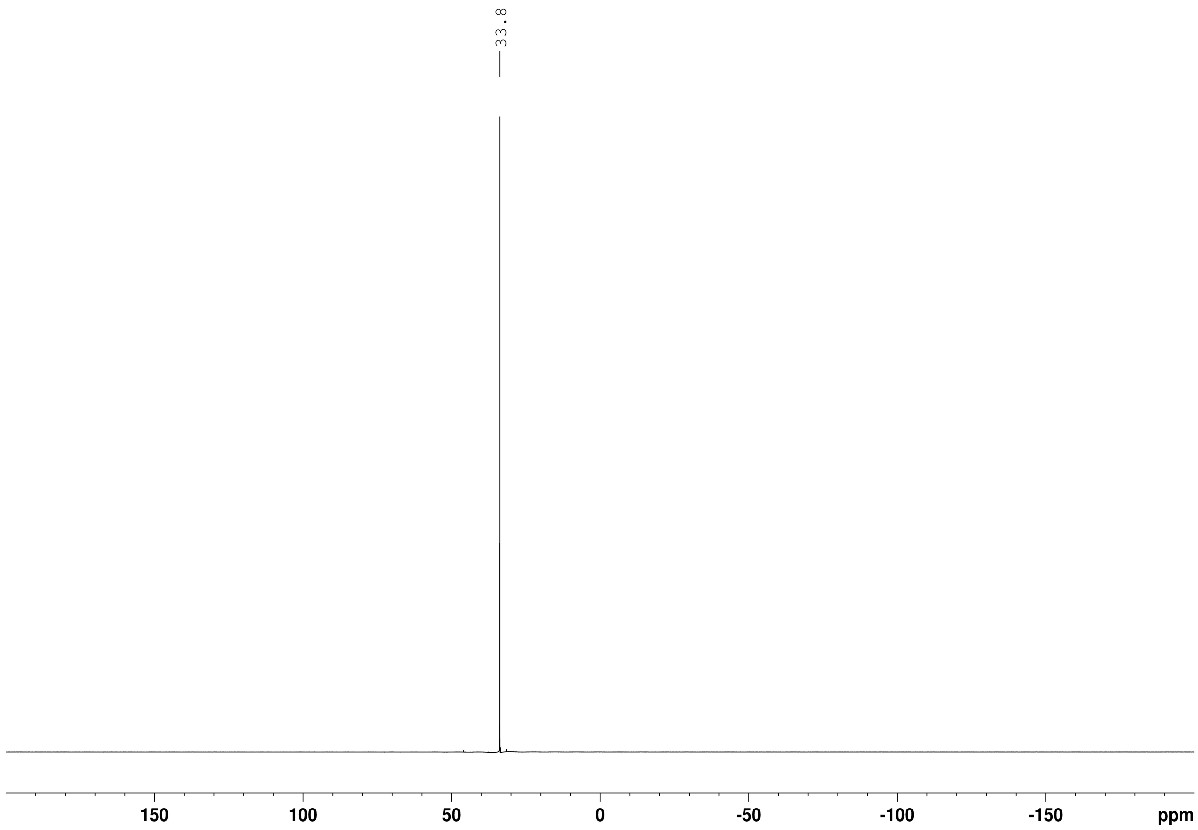

^{31}P NMR spectrum (162 MHz, DMSO-d_6) of **28**.

Tetramethylphosphonium hydrocinnamate (29)

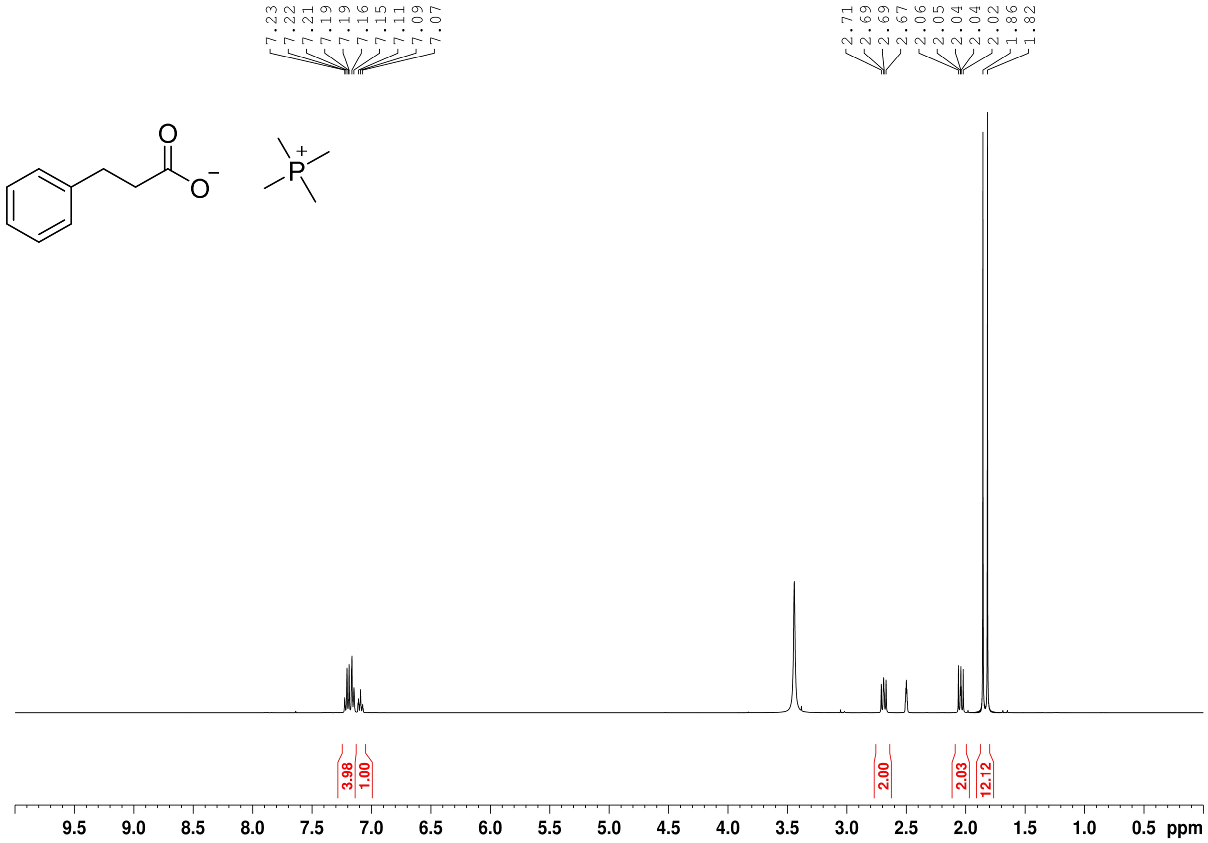

^{1}H NMR spectrum (400 MHz, DMSO-d_6) of **29**.

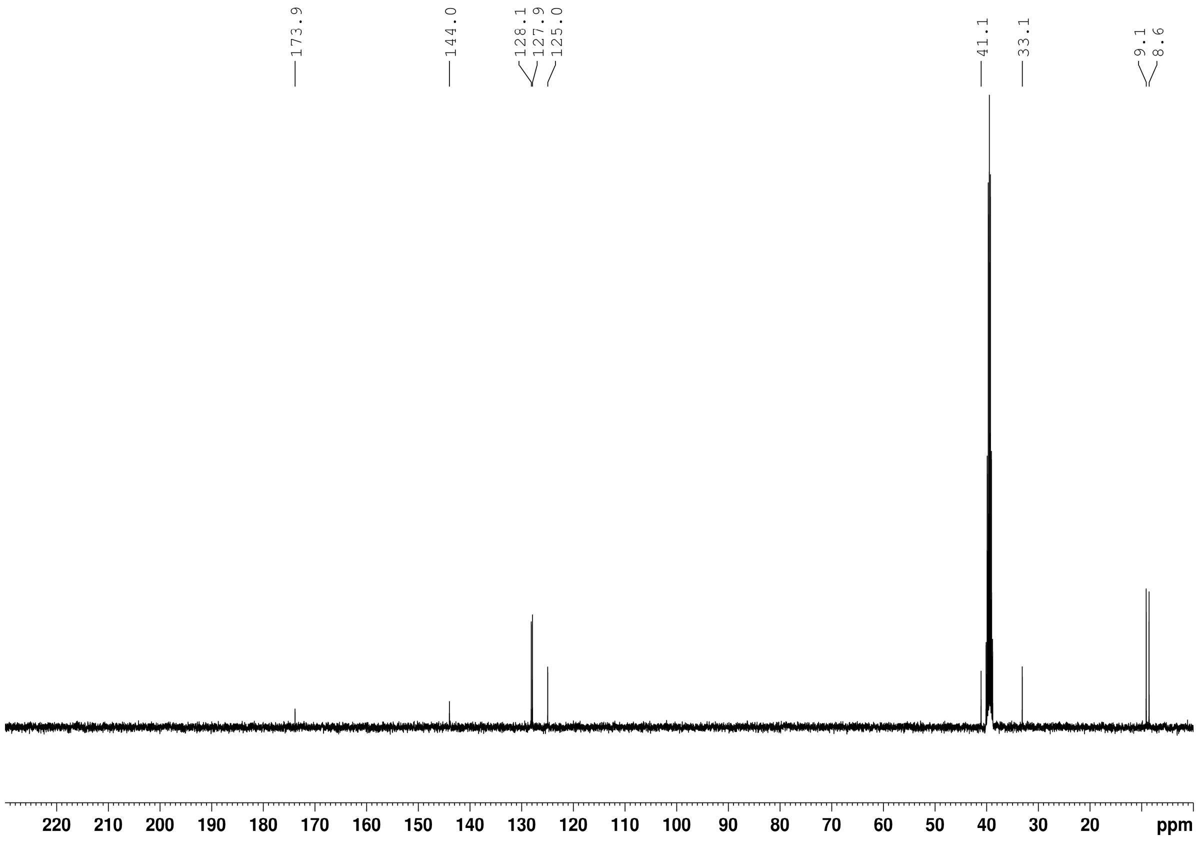

^{13}C NMR spectrum (100 MHz, DMSO-d_6) of **29**.

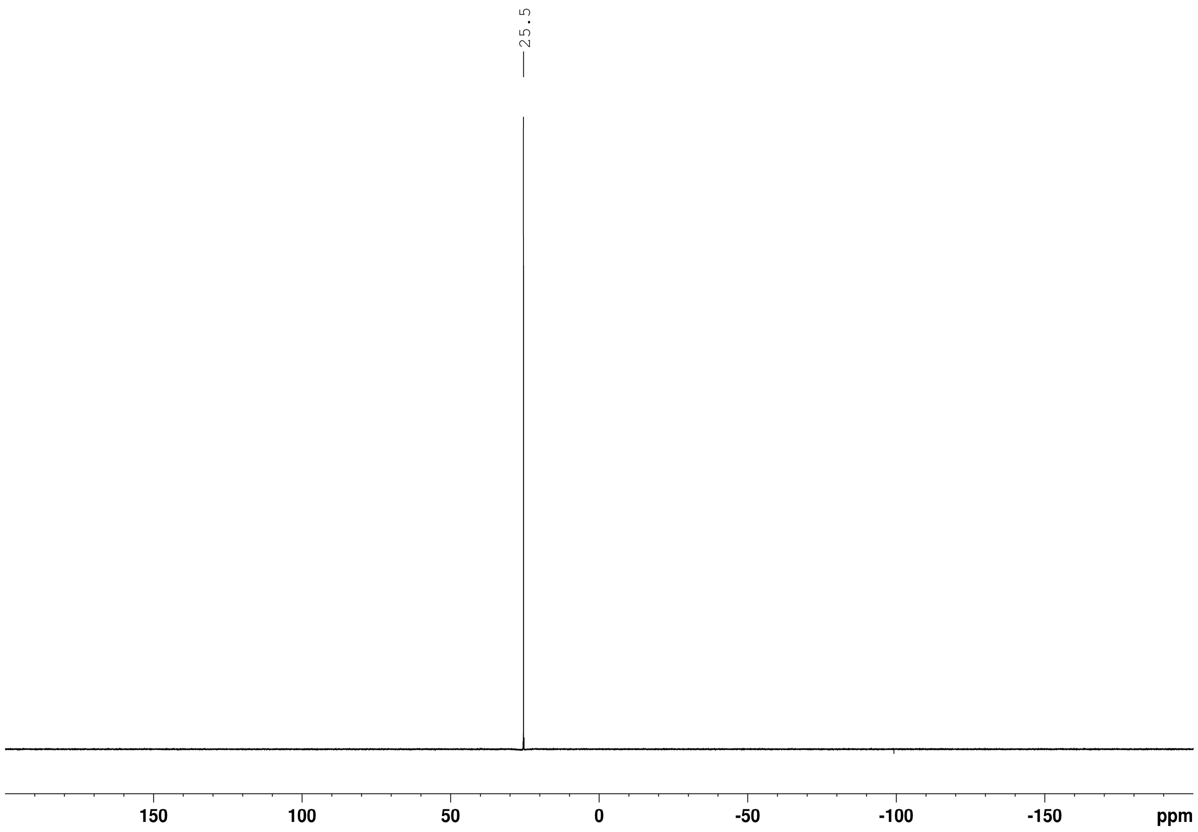

^{31}P NMR spectrum (162 MHz, DMSO-d_6) of **29**.

Tetraoctylphosphonium hydrocinnamate (30)

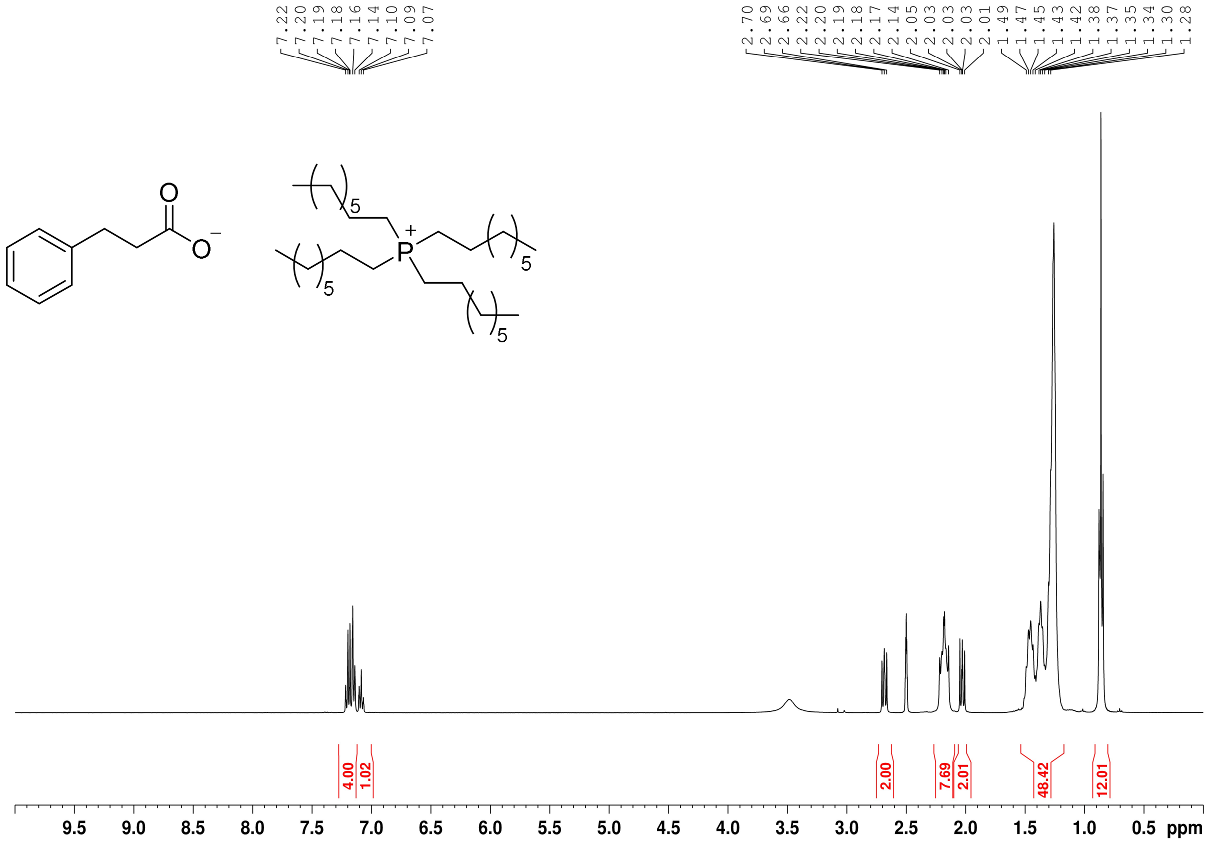

^{1}H NMR spectrum (400 MHz, DMSO-d_6) of **30**.

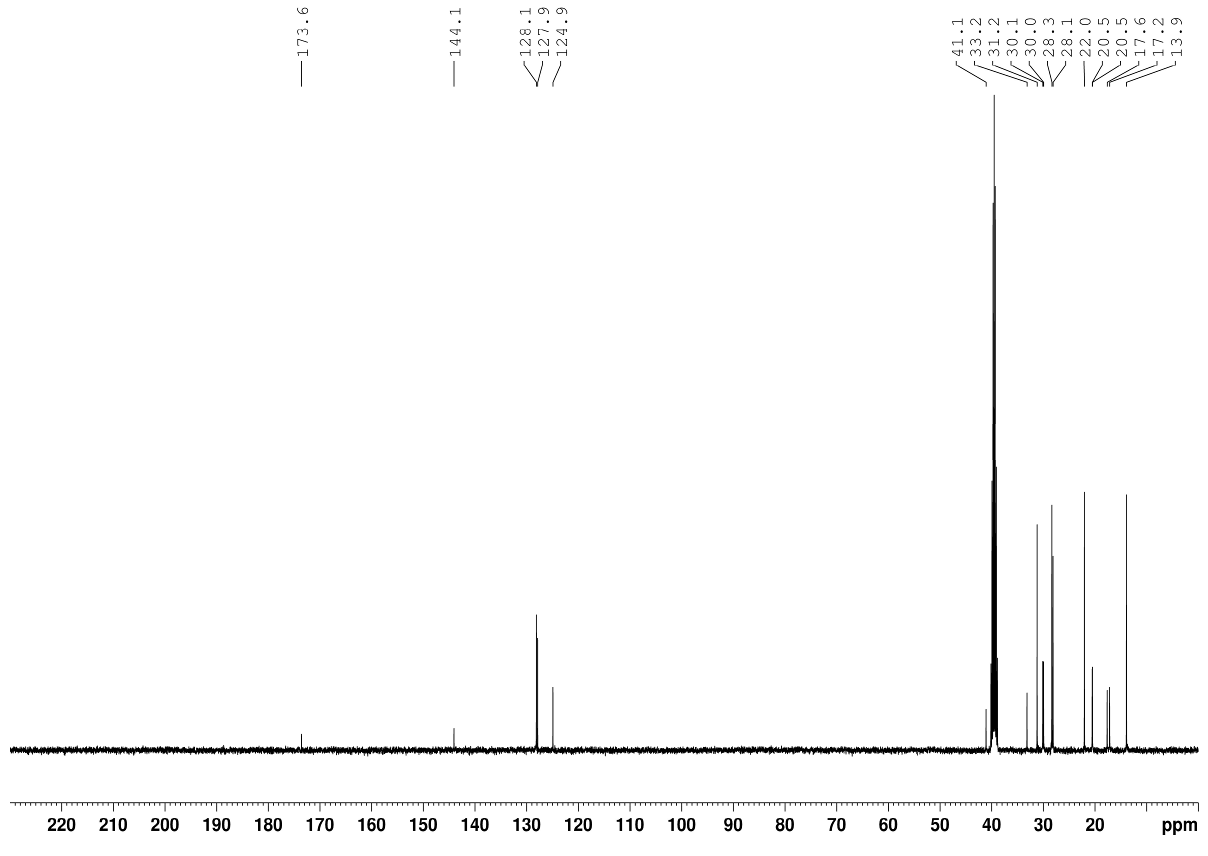

^{13}C NMR spectrum (100 MHz, DMSO-d_6) of **30**.

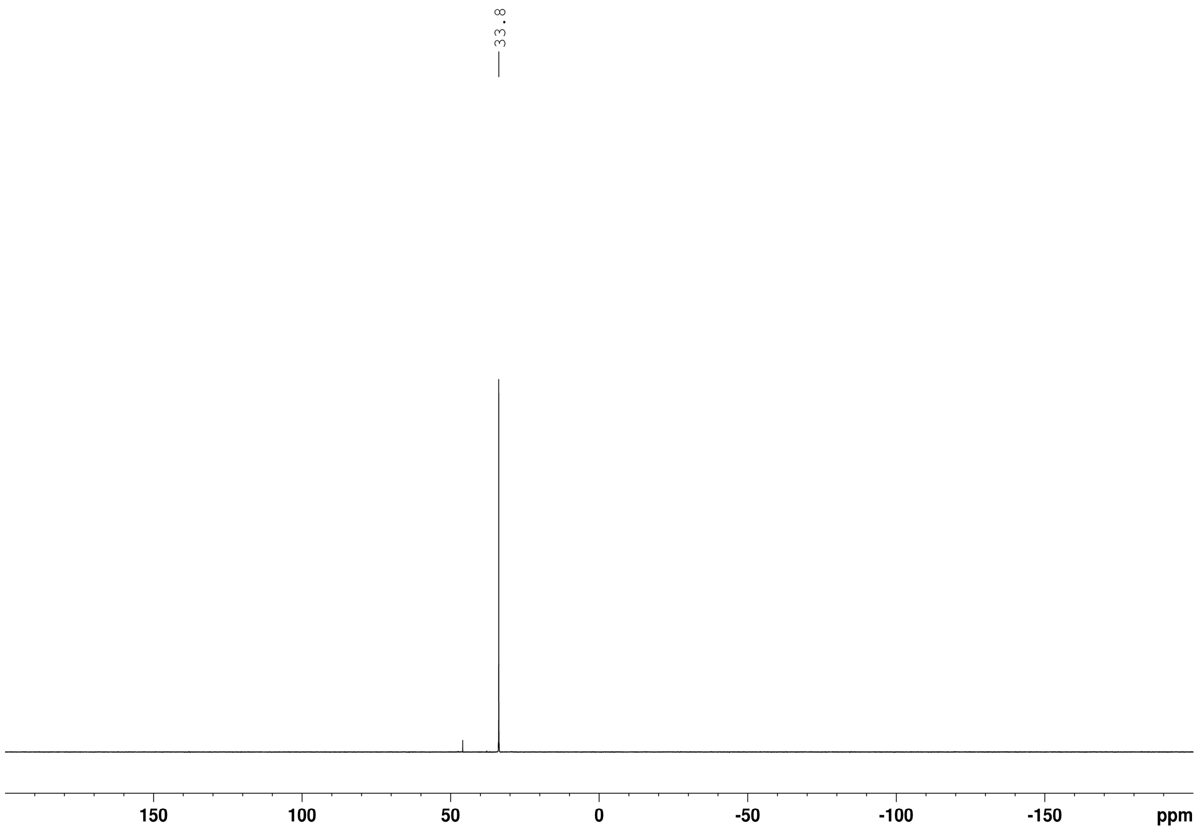

^{31}P NMR spectrum (162 MHz, DMSO-d_6) of **30**.

Trihexyltetradecylphosphonium hydrocinnamate (31)

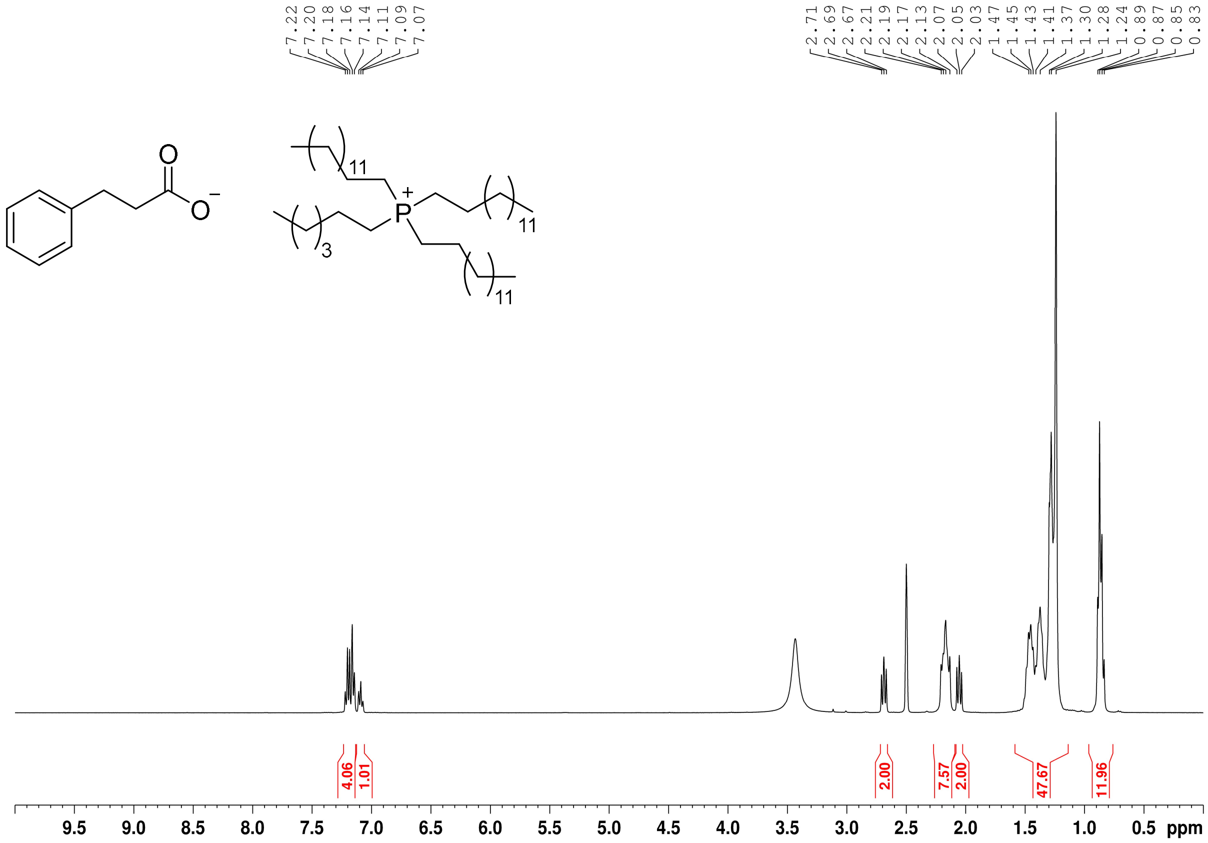

^{1}H NMR spectrum (400 MHz, DMSO-d_6) of **31**.

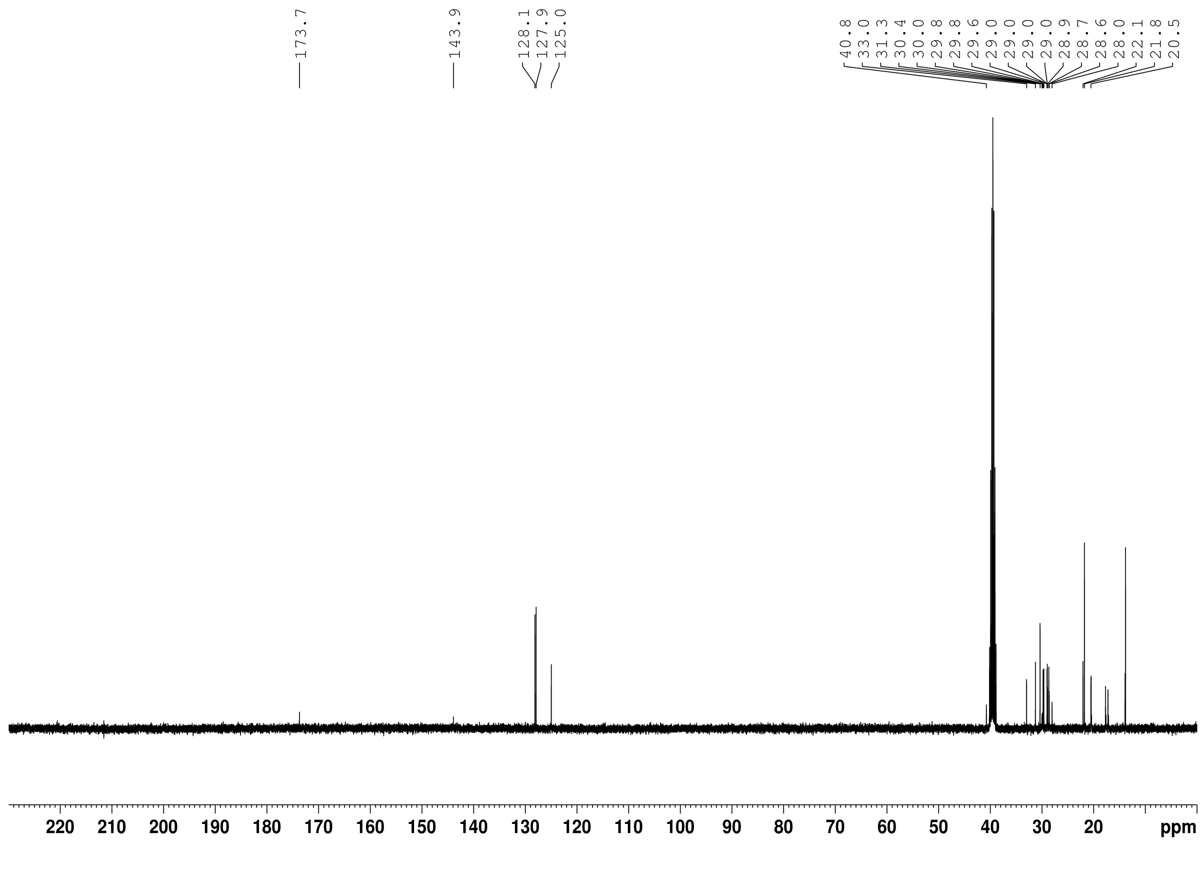

^{13}C NMR spectrum (100 MHz, DMSO-d_6) of **31**.

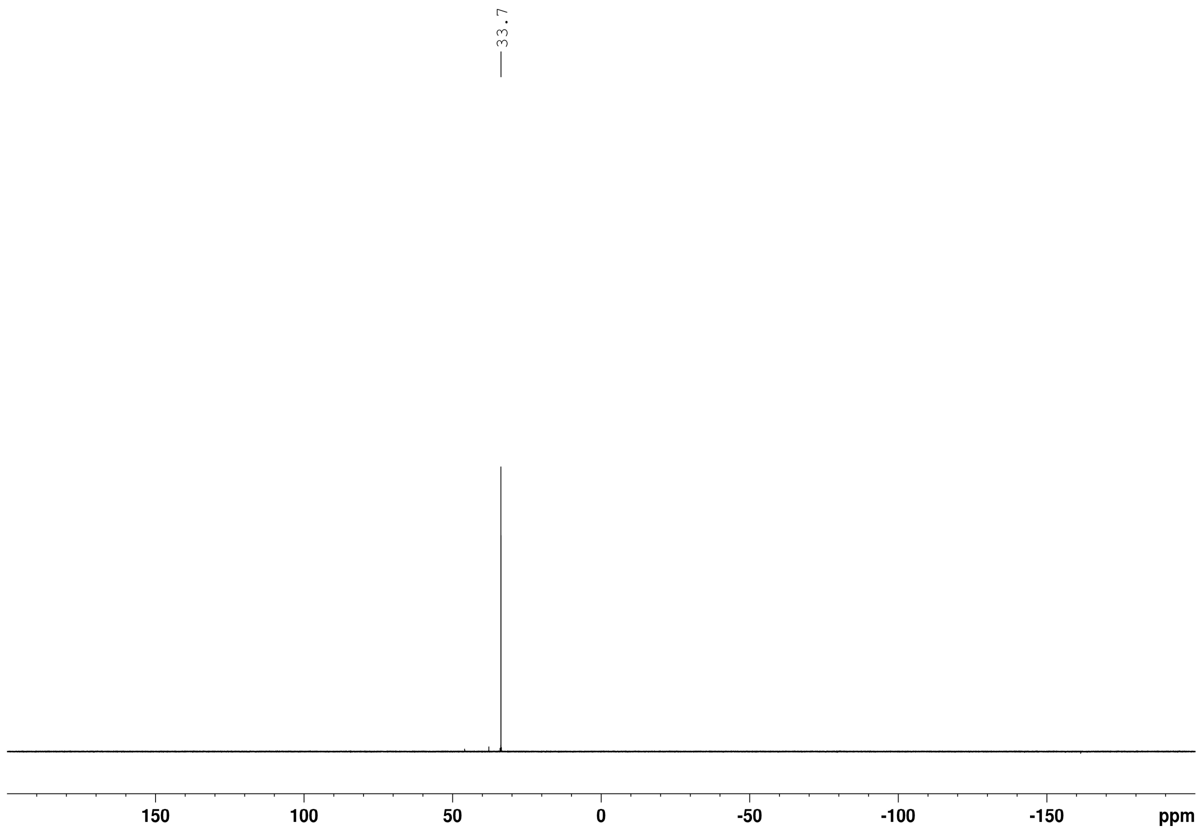

^{31}P NMR spectrum (162 MHz, DMSO-d_6) of **31**.

Methyltrioctylphosphonium iodide (S1)

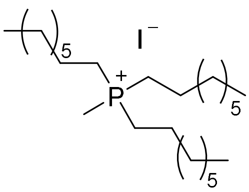

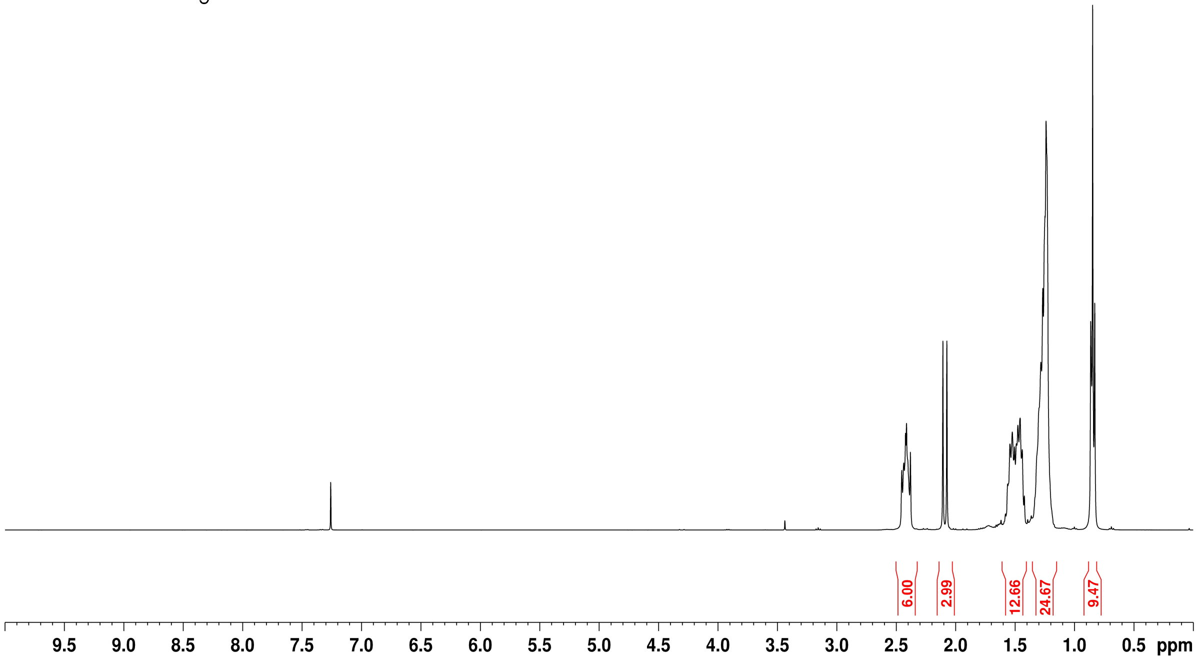

^{1}H NMR spectrum (400 MHz, $CDCl_3$) of **S1**.

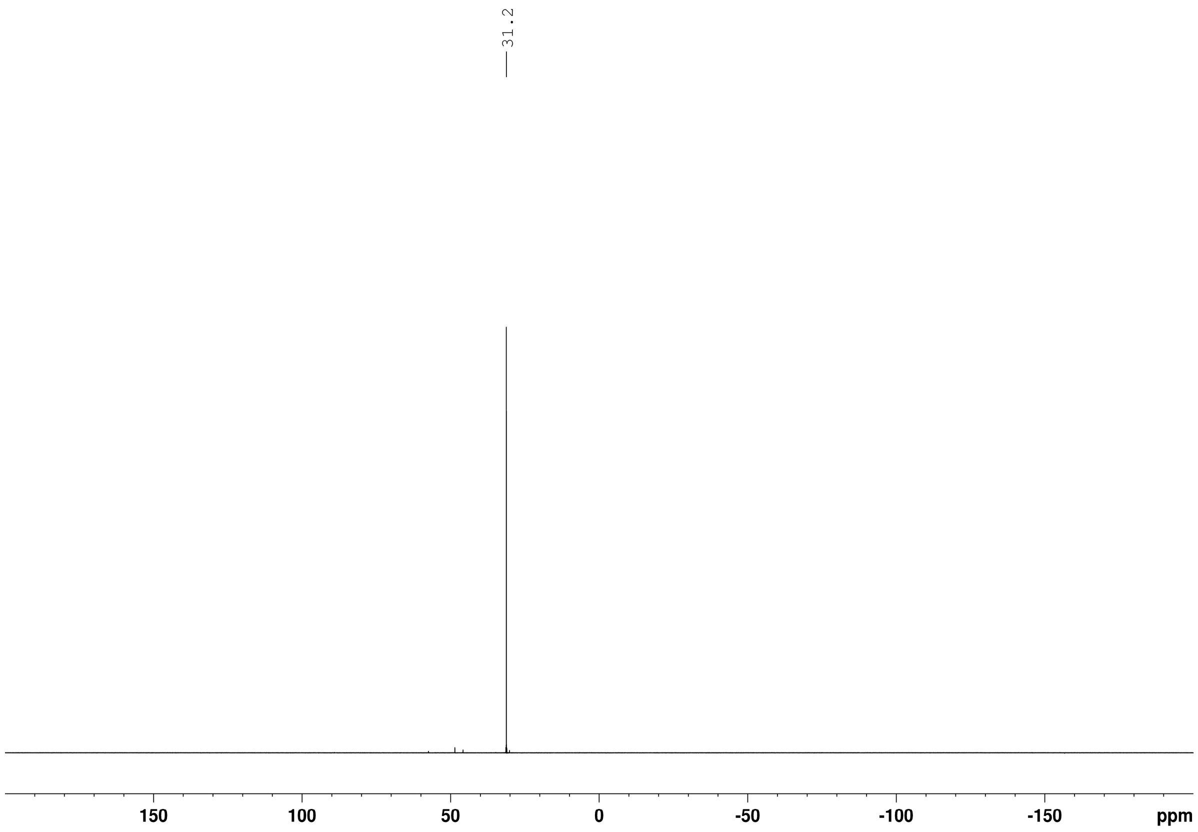

^{31}P NMR spectrum (162 MHz, $CDCl_3$) of **S1**.

Methyltrioctylphosphonium hydrocinnamate (32)

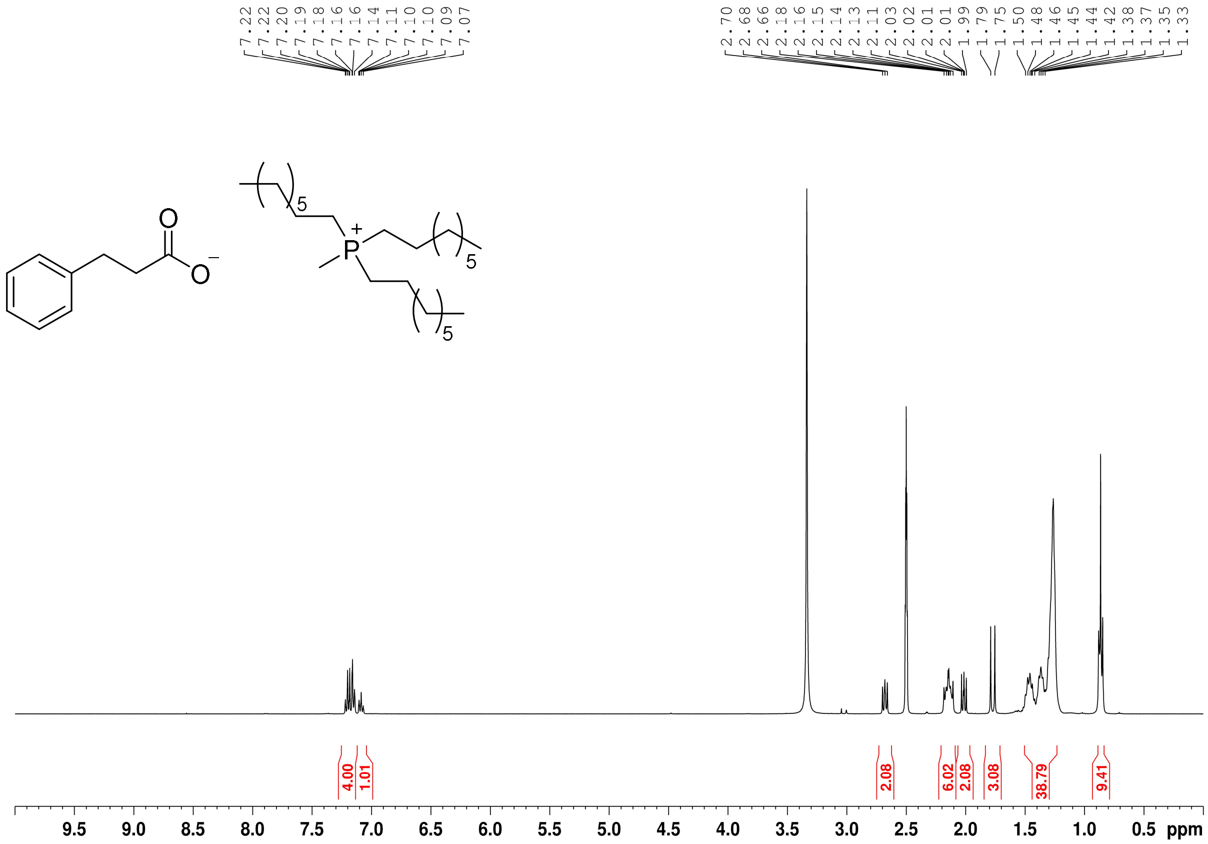

^{1}H NMR spectrum (400 MHz, DMSO-d_6) of **32**.

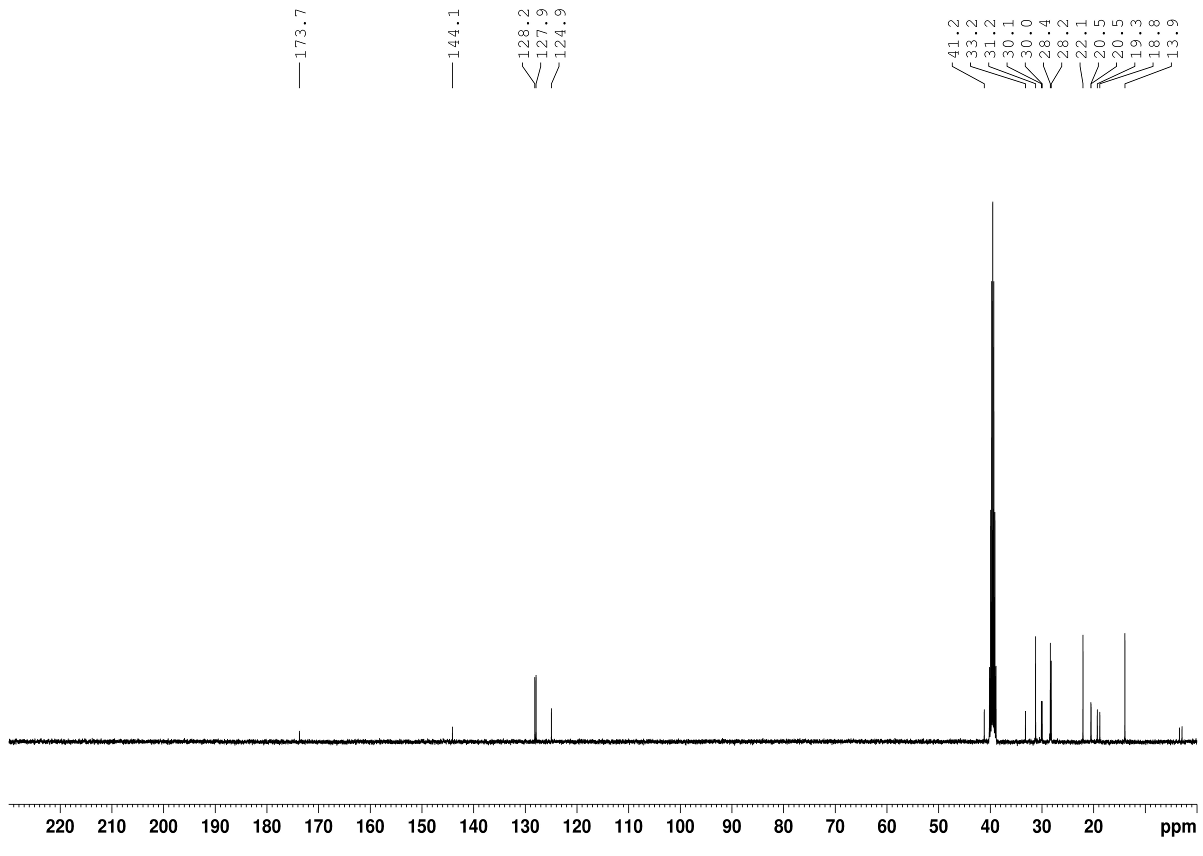

^{13}C NMR spectrum (100 MHz, DMSO-d_6) of **32**.

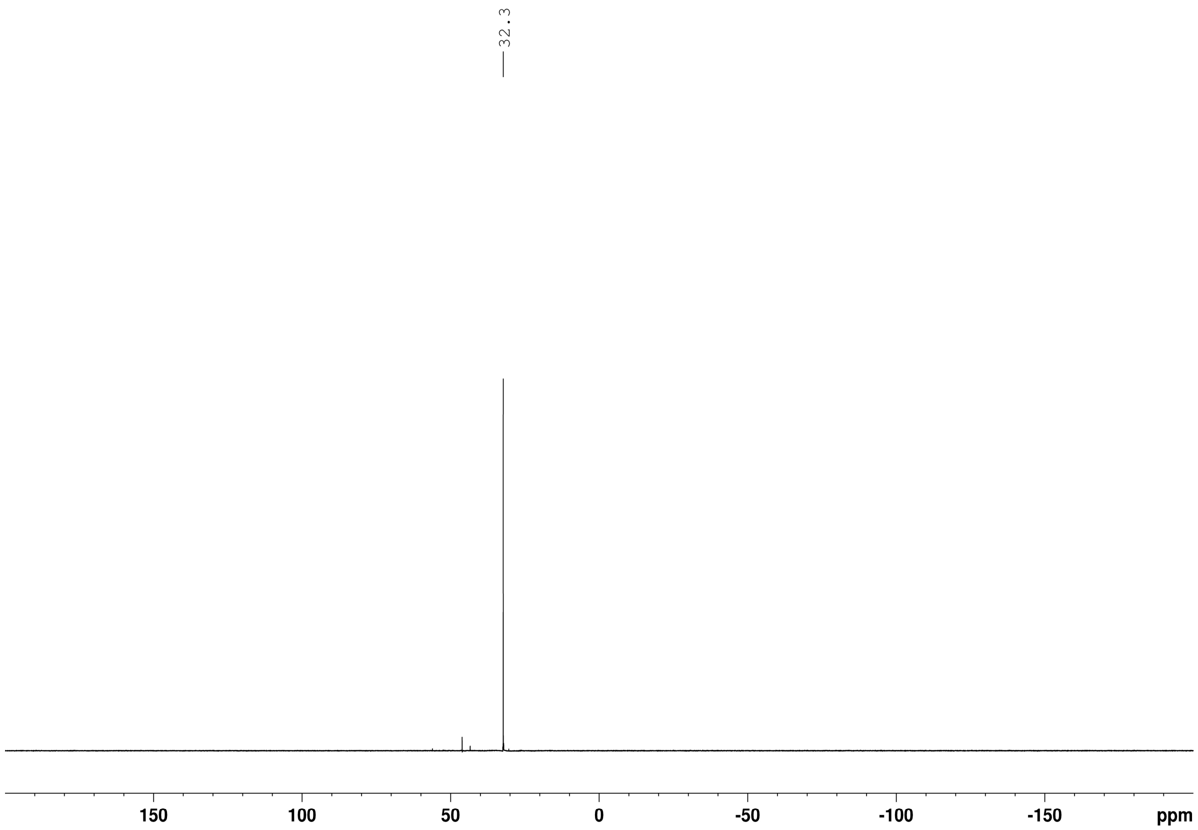

^{31}P NMR spectrum (162 MHz, DMSO-d_6) of **32**.

Tributyl-3-phenylpropylophosphonium bromide (S2)

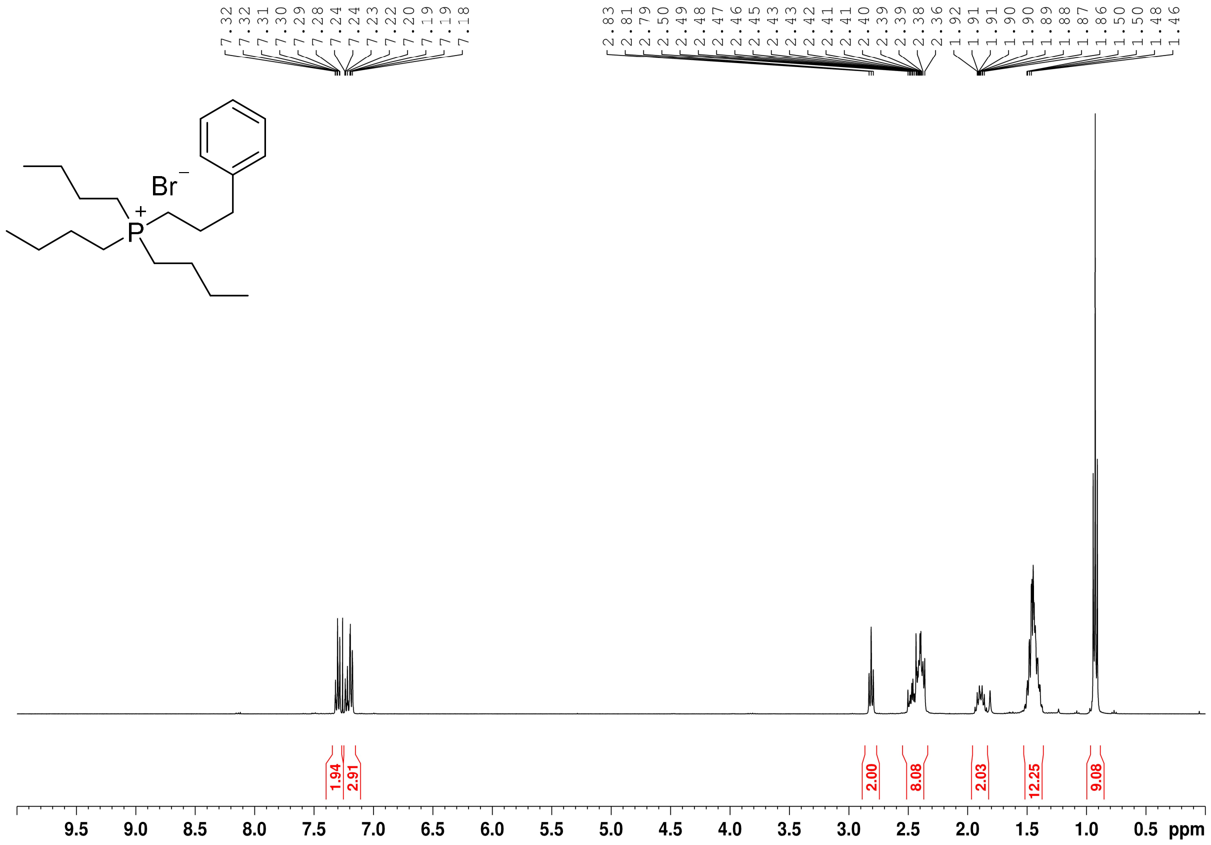

^{1}H NMR spectrum (400 MHz, $CDCl_3$) of **S2**.

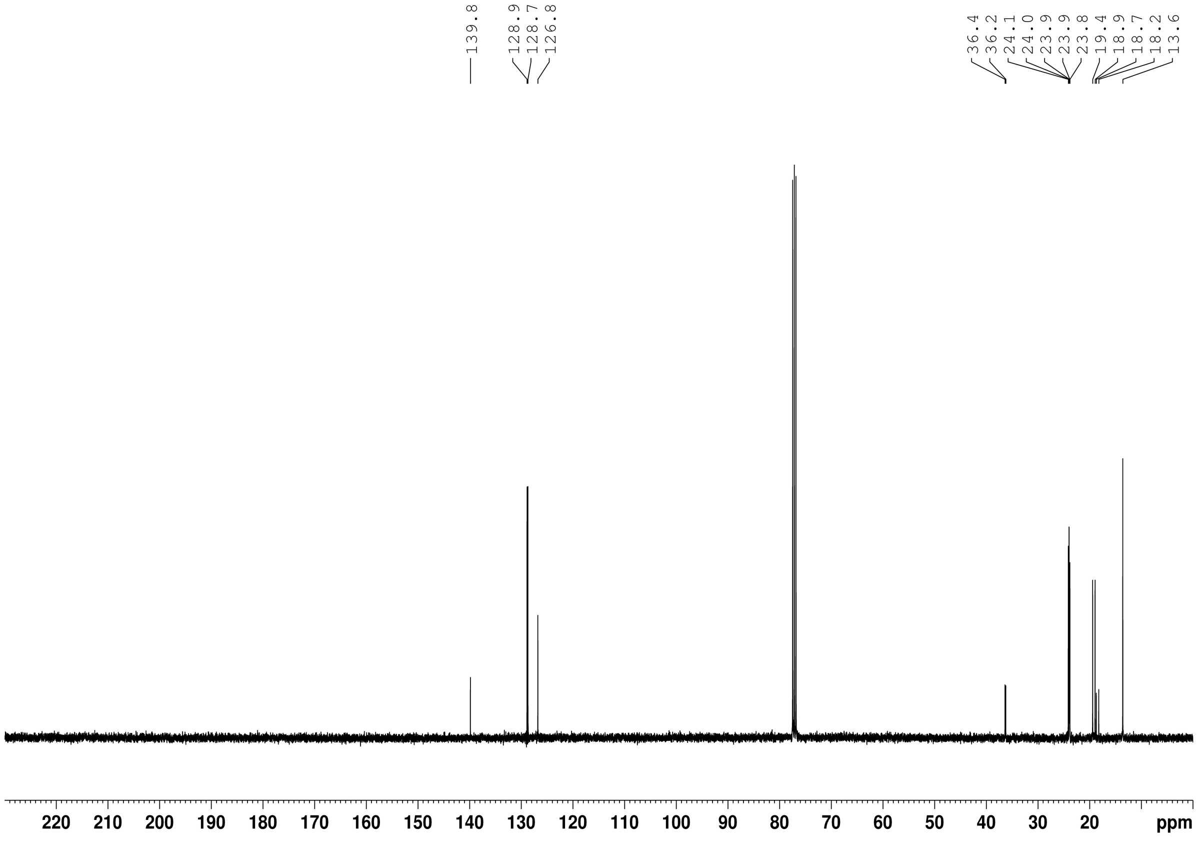

^{13}C NMR spectrum (100 MHz, $CDCl_3$) of **S2**.

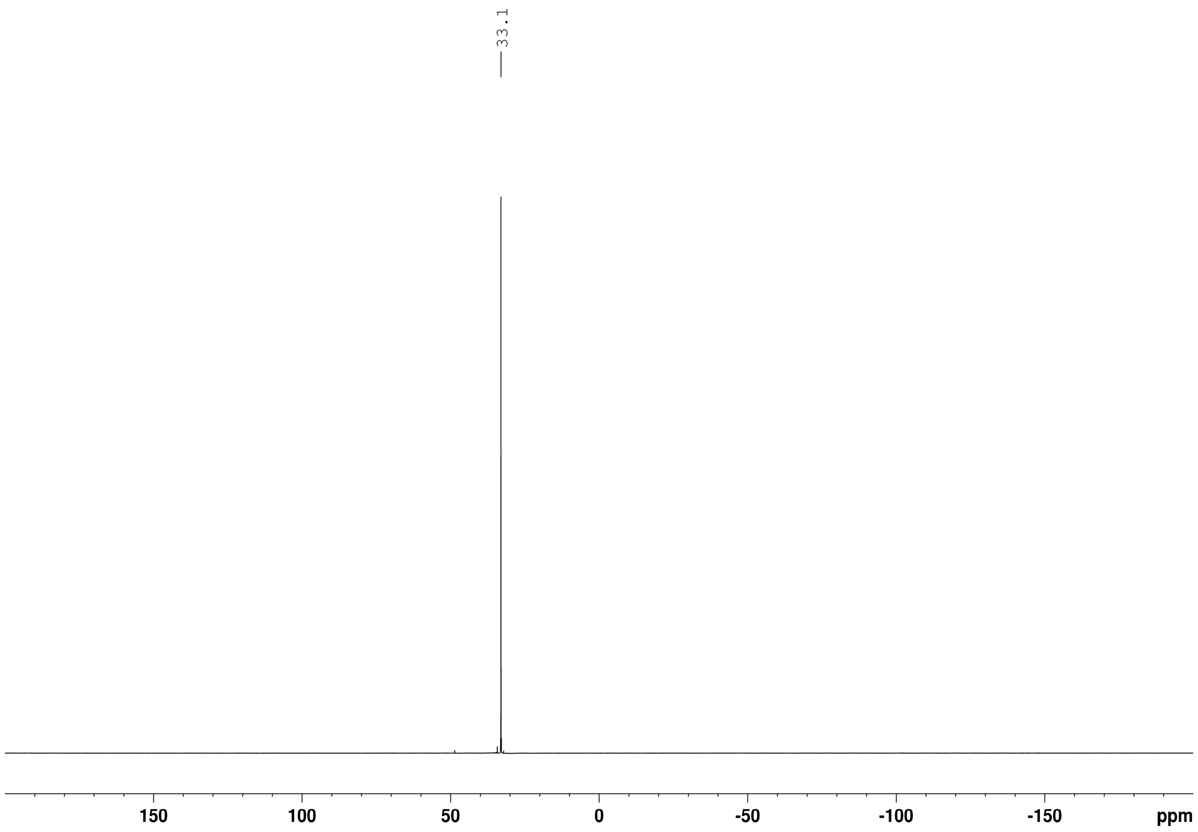

^{31}P NMR spectrum (162 MHz, $CDCl_3$) of **S2**.

Tributyl-3-phenylpropylophosphonium hydrocinnamate (33)

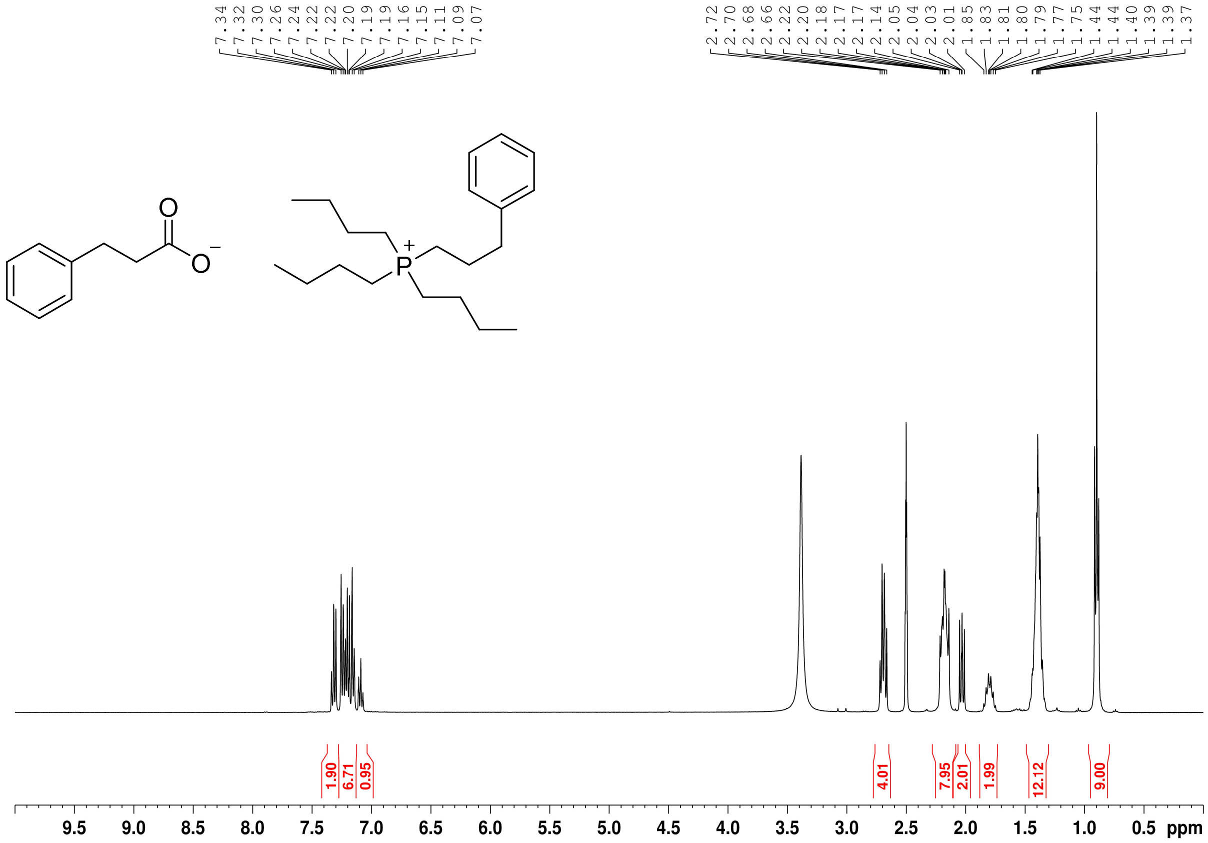

^{1}H NMR spectrum (400 MHz, DMSO-d_6) of **33**.

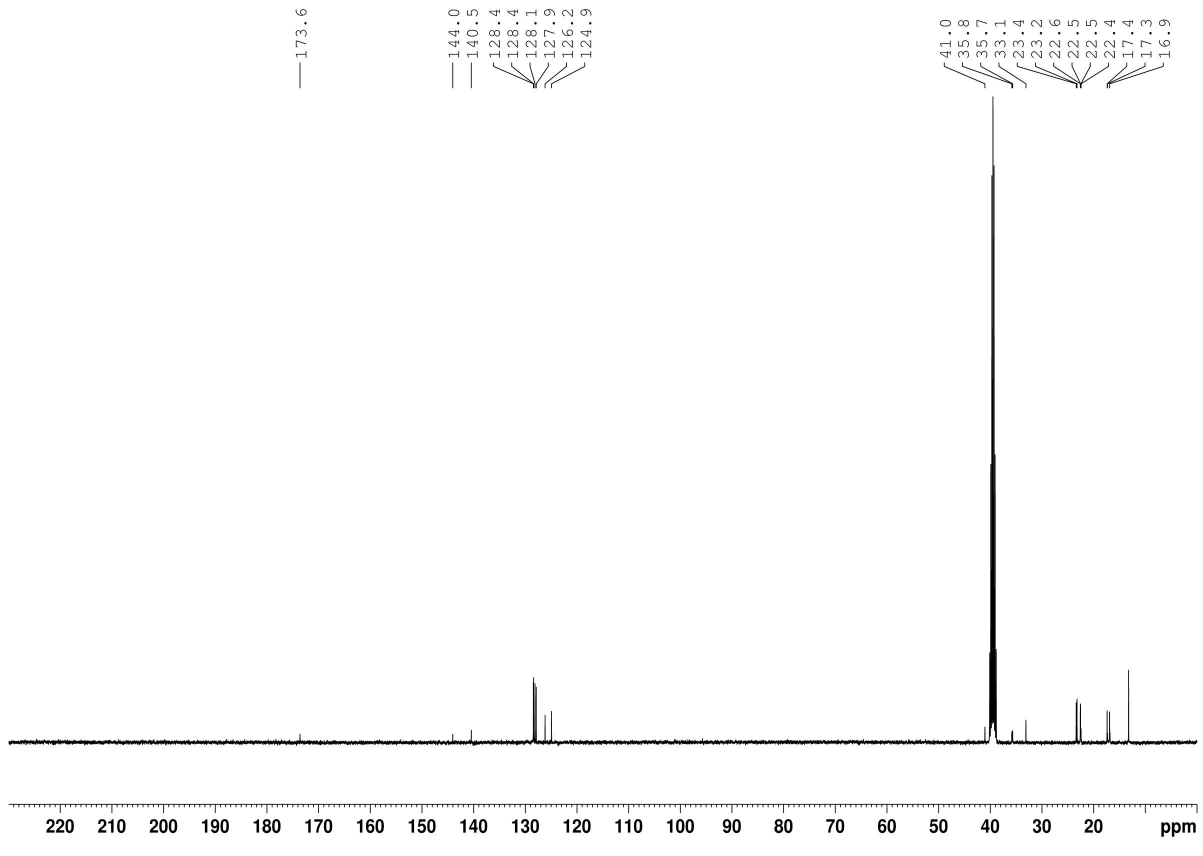

^{13}C NMR spectrum (100 MHz, DMSO-d_6) of **33**.

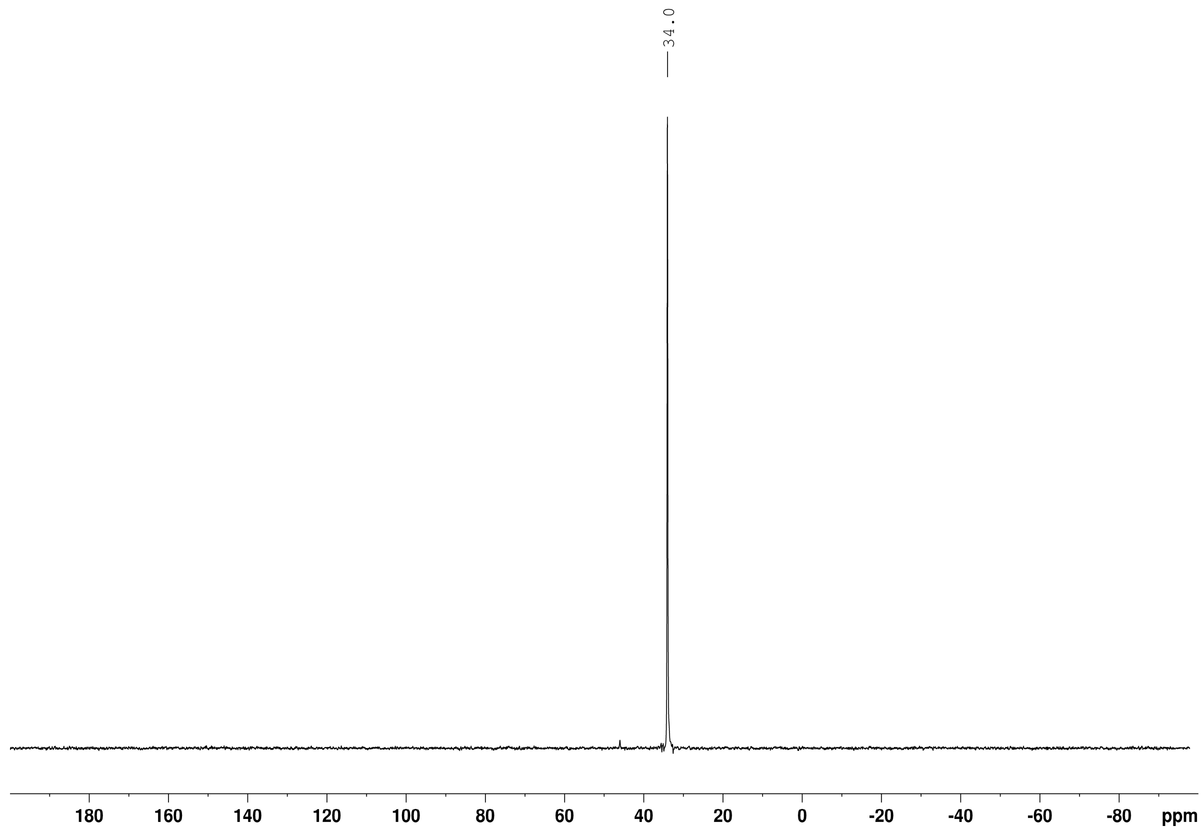

^{31}P NMR spectrum (162 MHz, DMSO-d_6) of **33**.

Benzyltributylphosphonium bromide (S3)

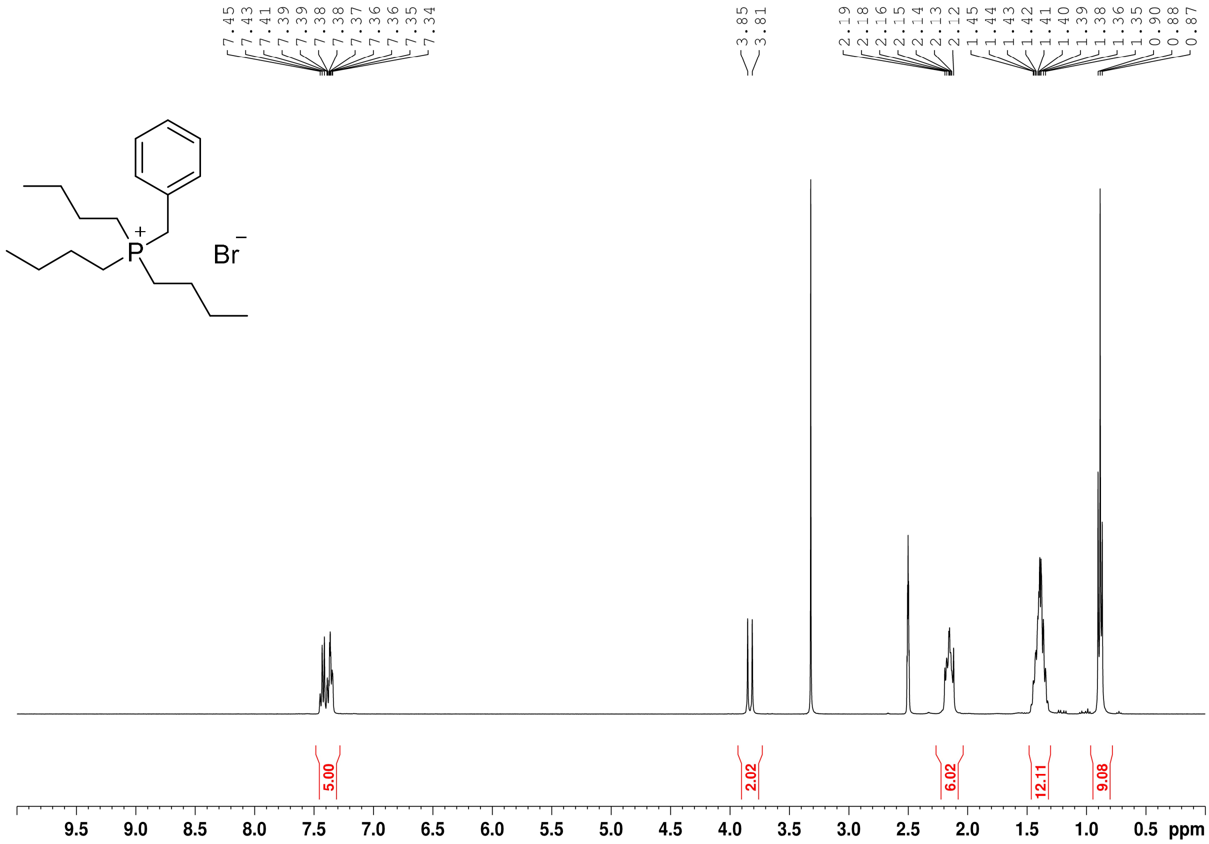

^{1}H NMR spectrum (400 MHz, DMSO-d_6) of **S3**.

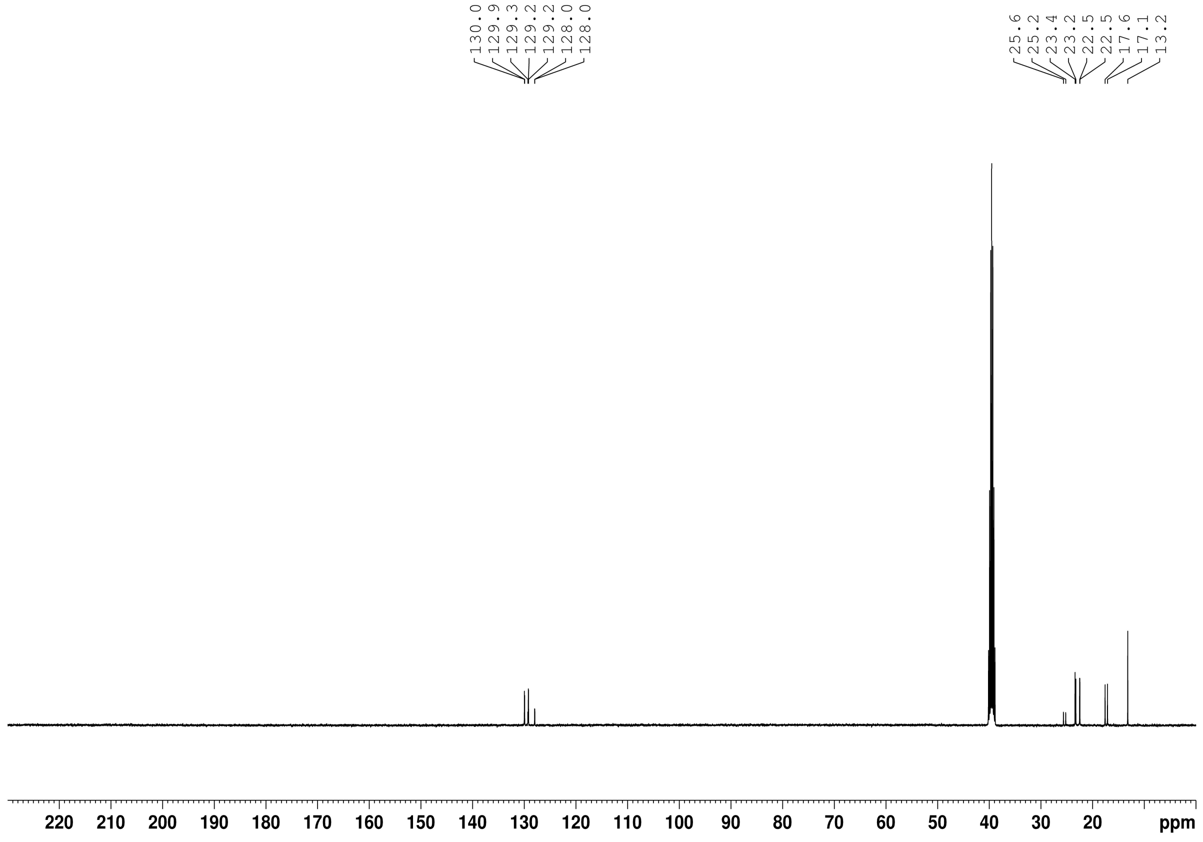

^{13}C NMR spectrum (100 MHz, DMSO-d_6) of **S3**.

Benzyltributylphosphonium hydrocinnamate (34)

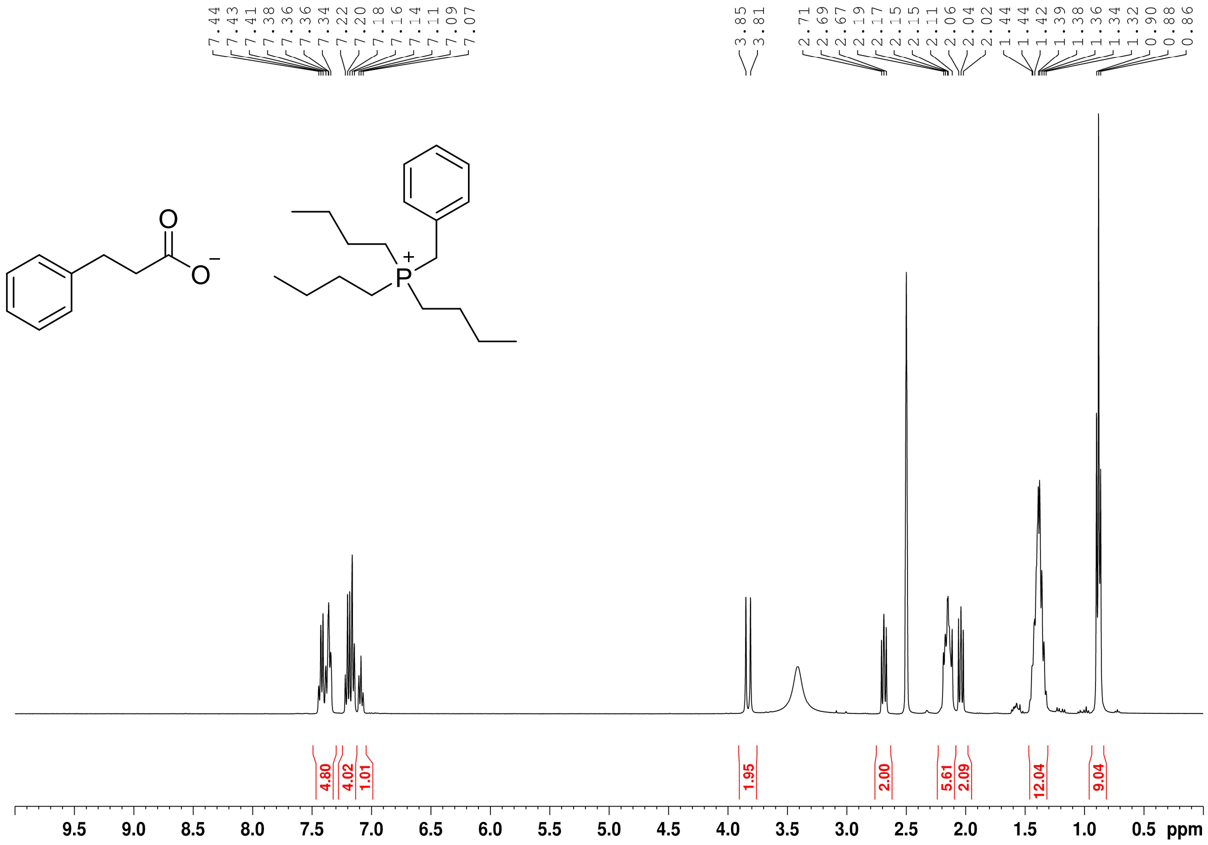

^{1}H NMR spectrum (400 MHz, DMSO-d_6) of **34**.

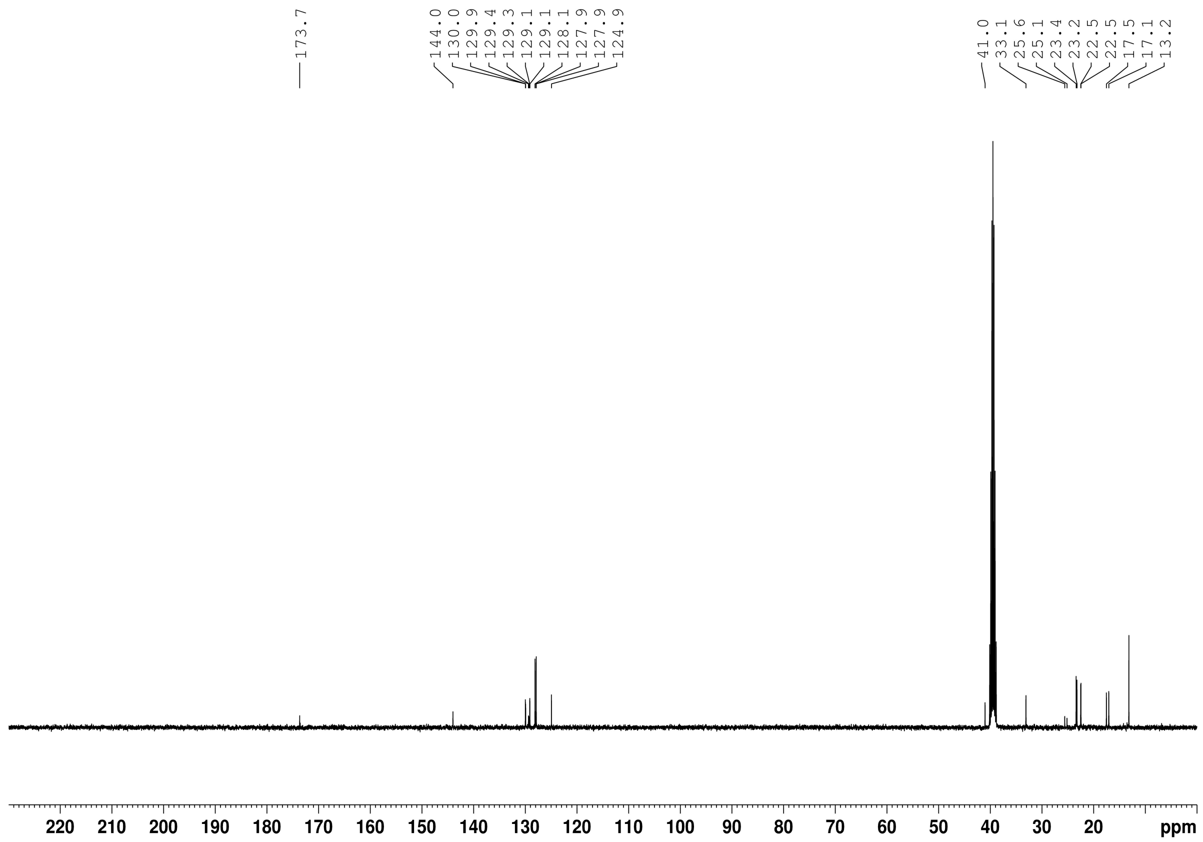

^{13}C NMR spectrum (100 MHz, DMSO-d_6) of **34**.

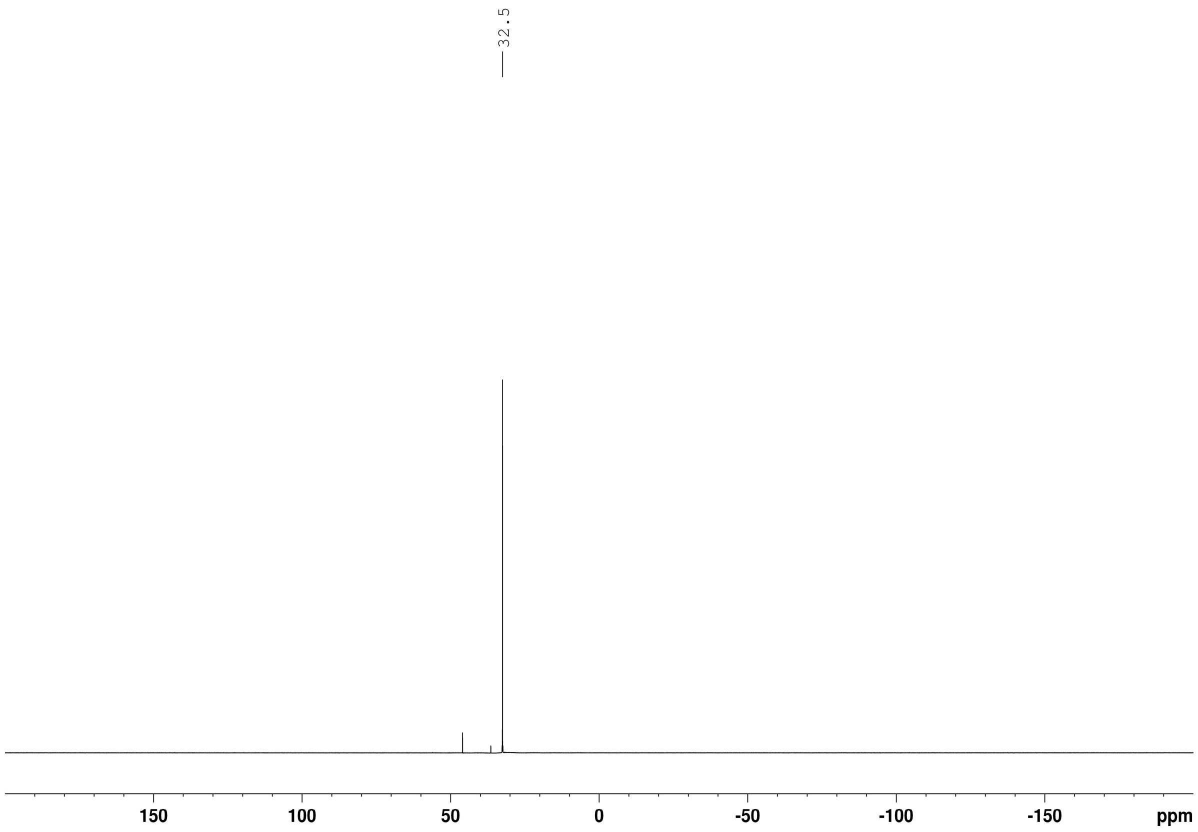

^{31}P NMR spectrum (162 MHz, DMSO-d_6) of **34**.

Tributyl(naphthalen-1-ylmethyl)phosphonium bromide (S4)

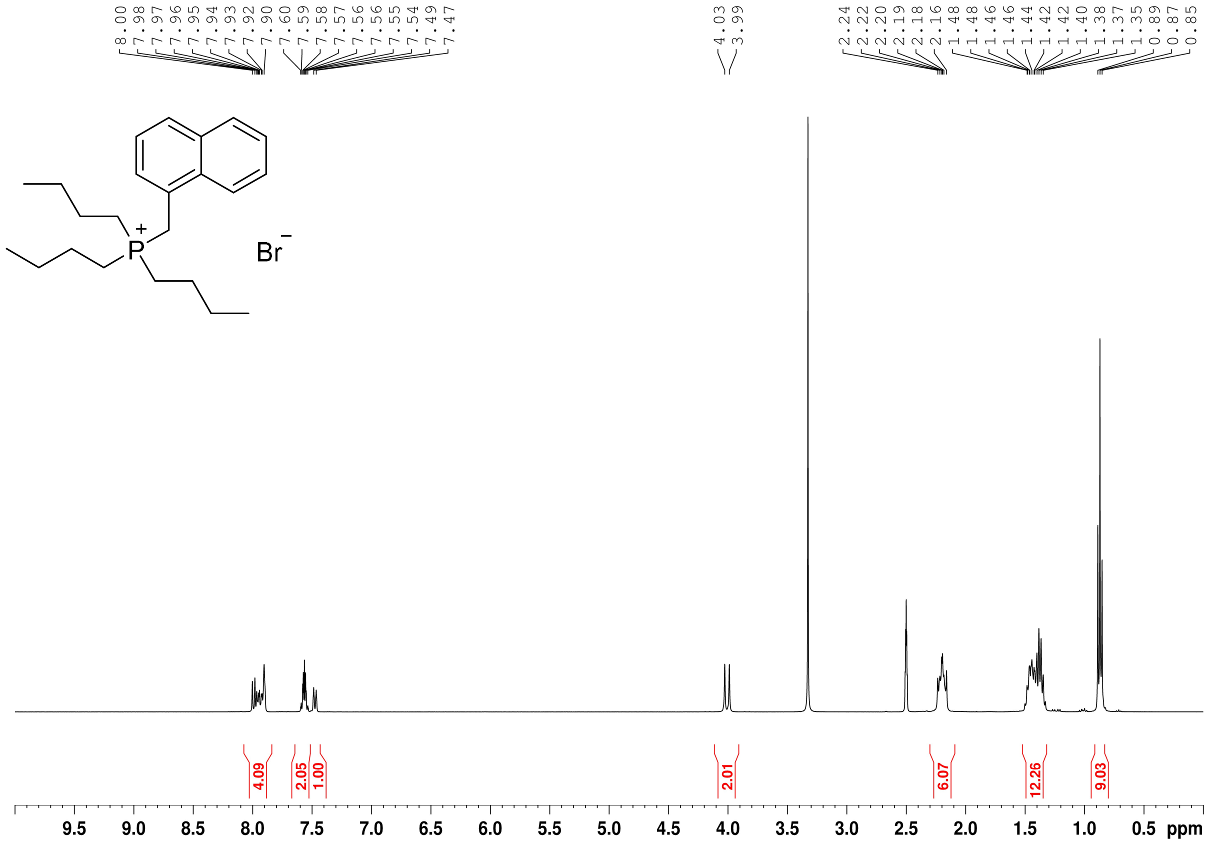

^{1}H NMR spectrum (400 MHz, DMSO-d_6) of **S4**.

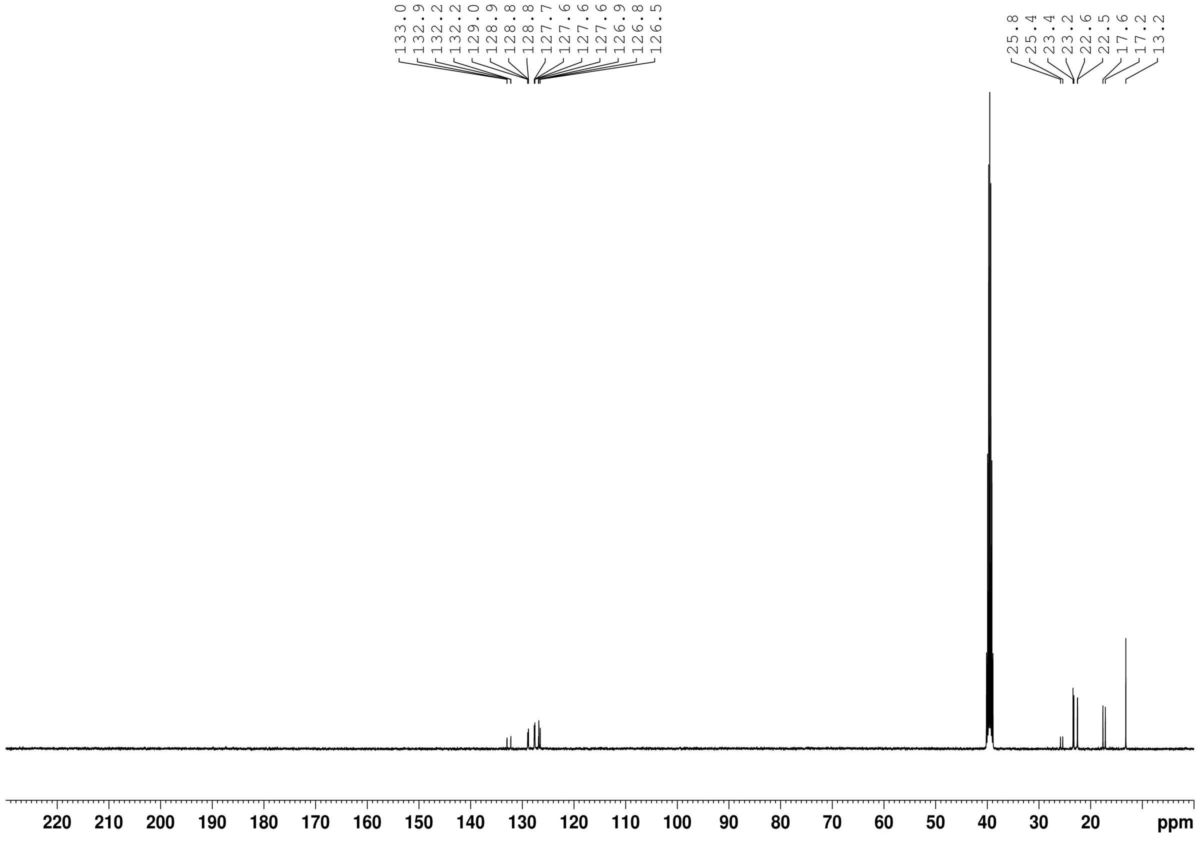

^{13}C NMR spectrum (100 MHz, DMSO-d_6) of **S4**.

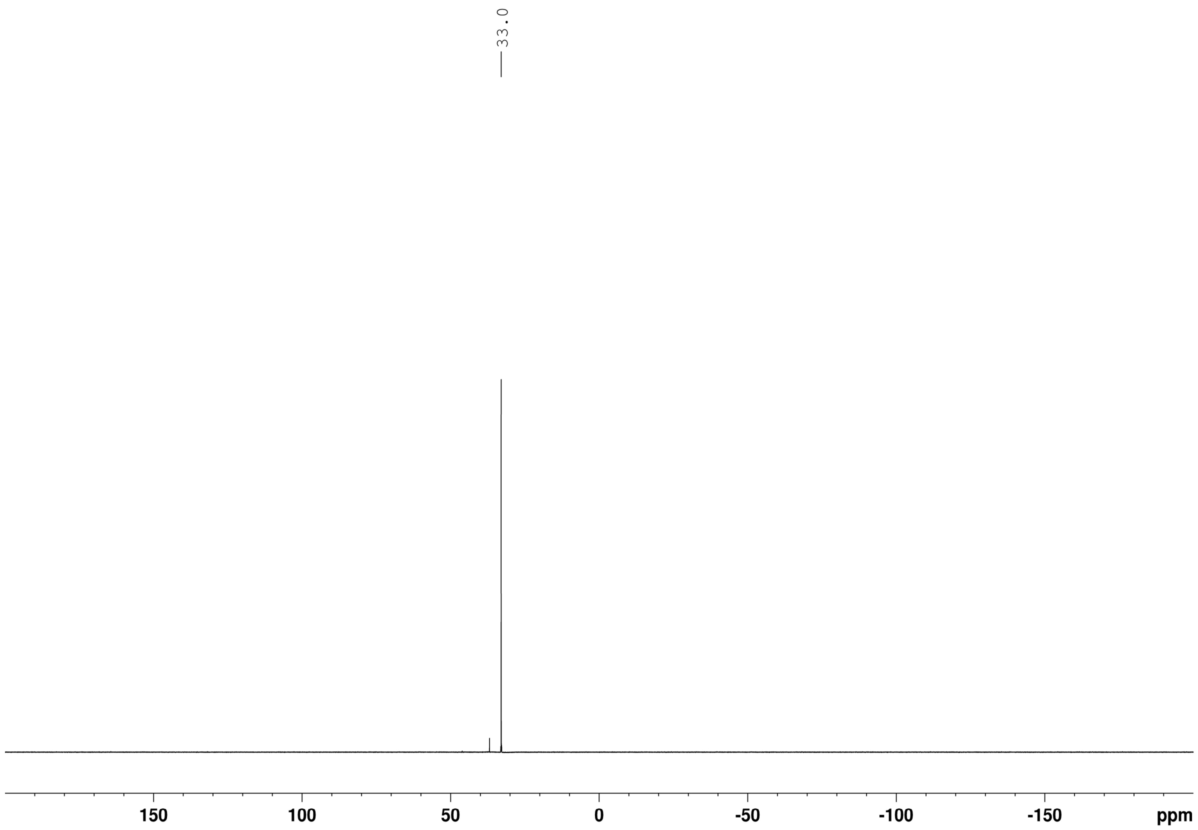

^{31}P NMR spectrum (162 MHz, DMSO-d_6) of **S4**.

Tributyl(naphthalen-1-ylmethyl)phosphonium hydrocinnamate (35)

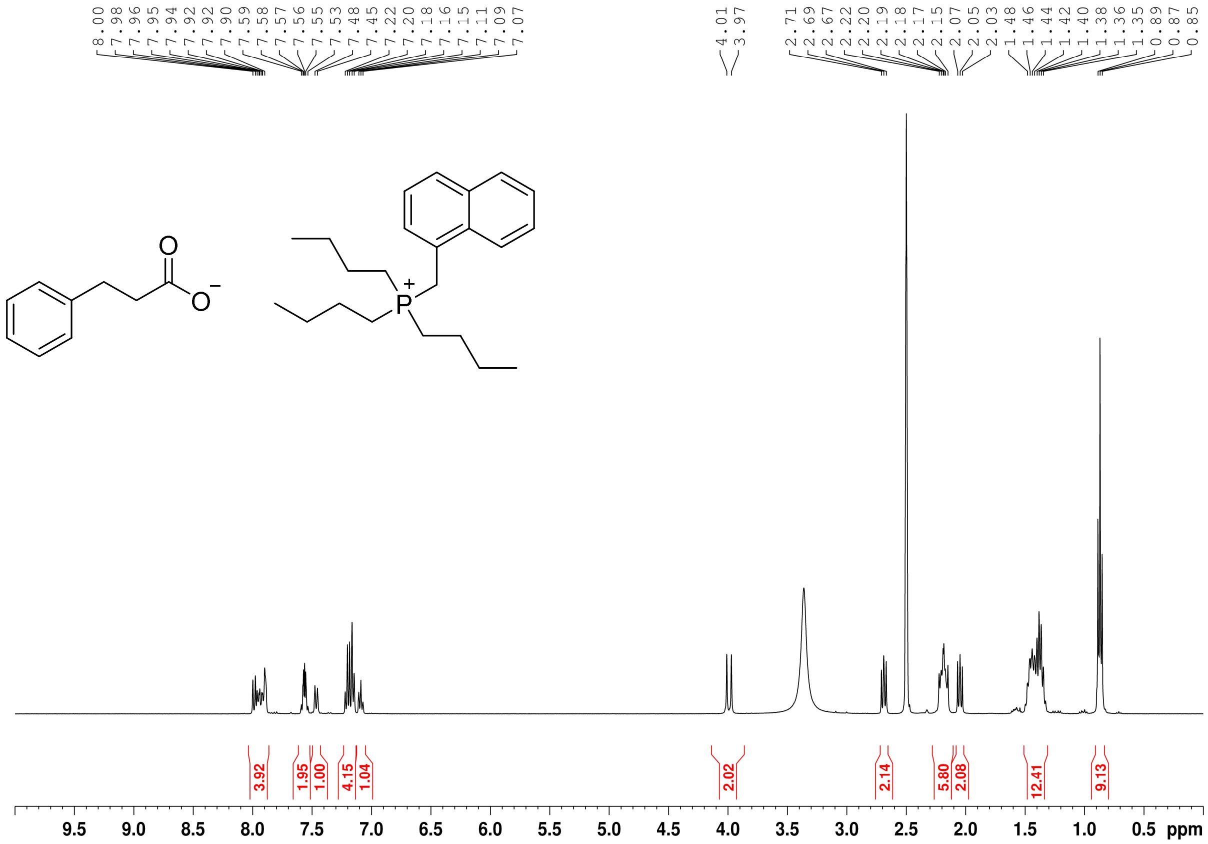

^{1}H NMR spectrum (400 MHz, DMSO-d_6) of **35**.

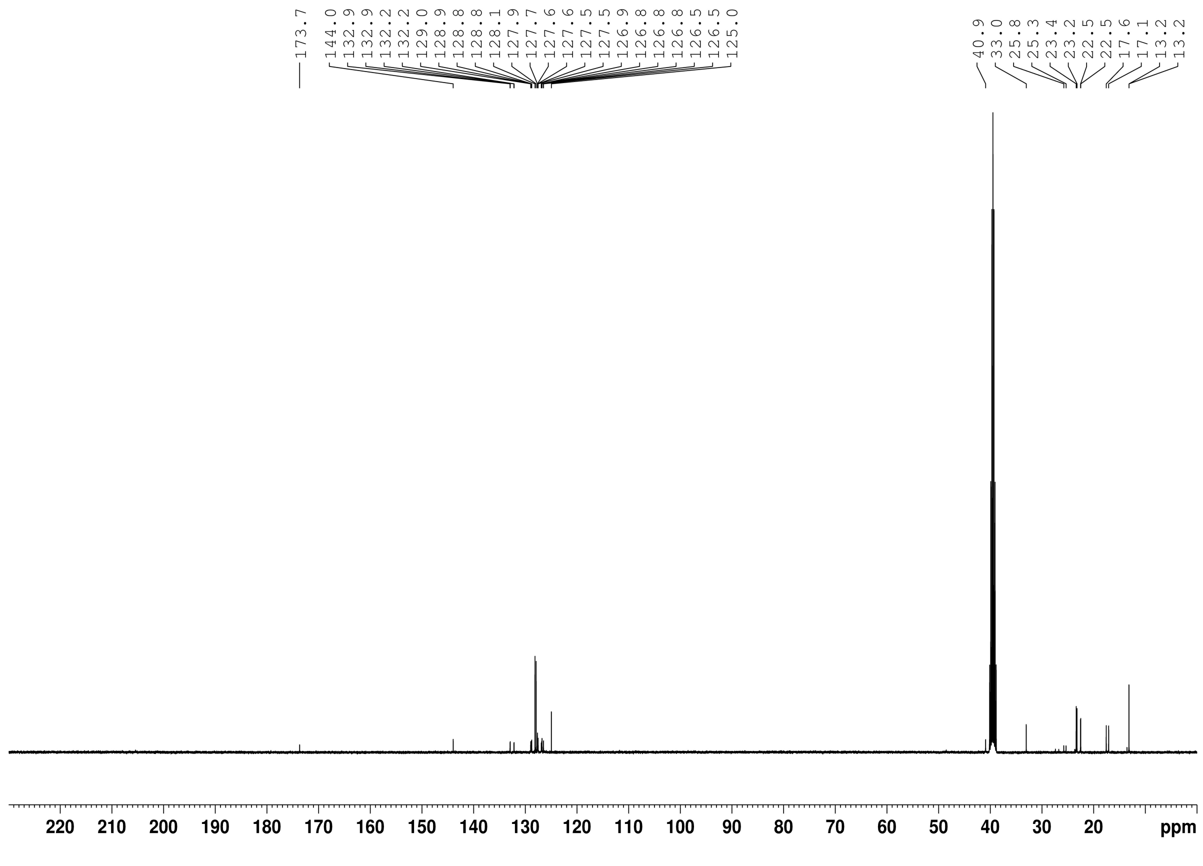

^{13}C NMR spectrum (100 MHz, DMSO-d_6) of **35**.

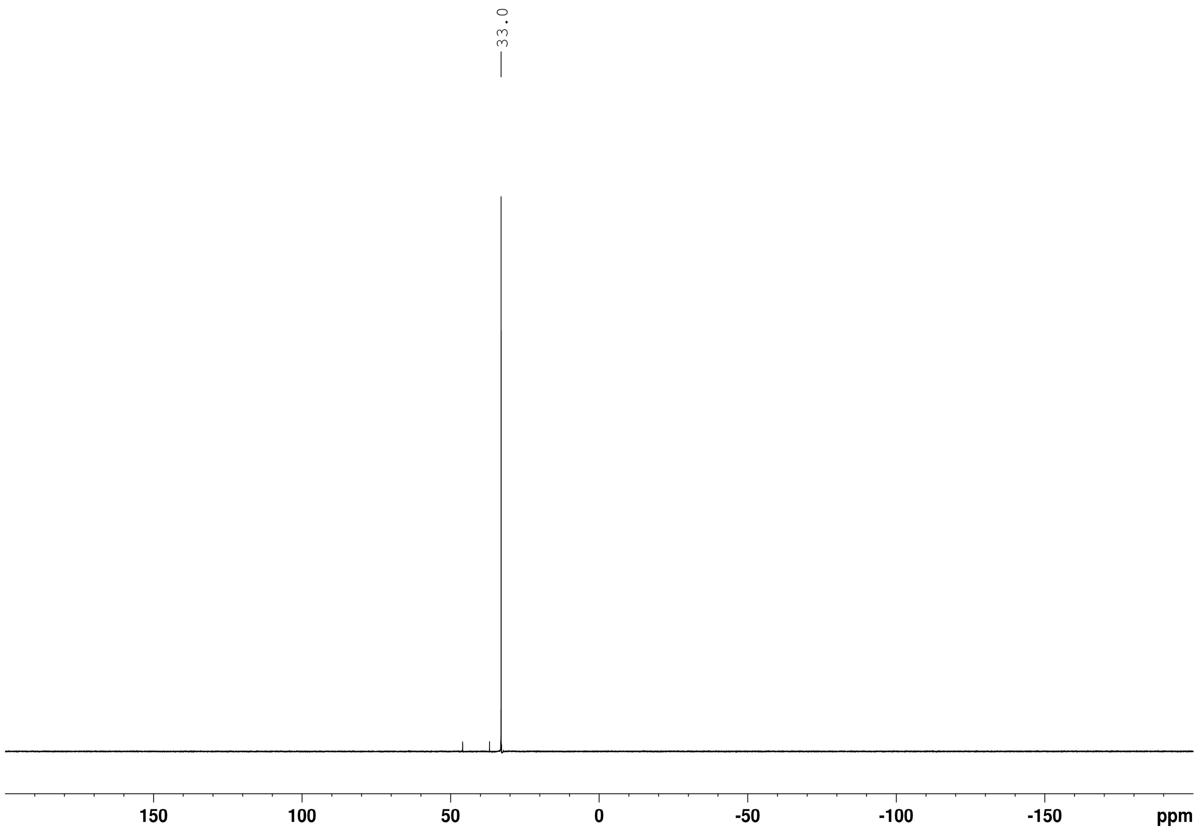

^{31}P NMR spectrum (162 MHz, DMSO-d_6) of **35**.

Tetramethylammonium hydrocinnamate (36)

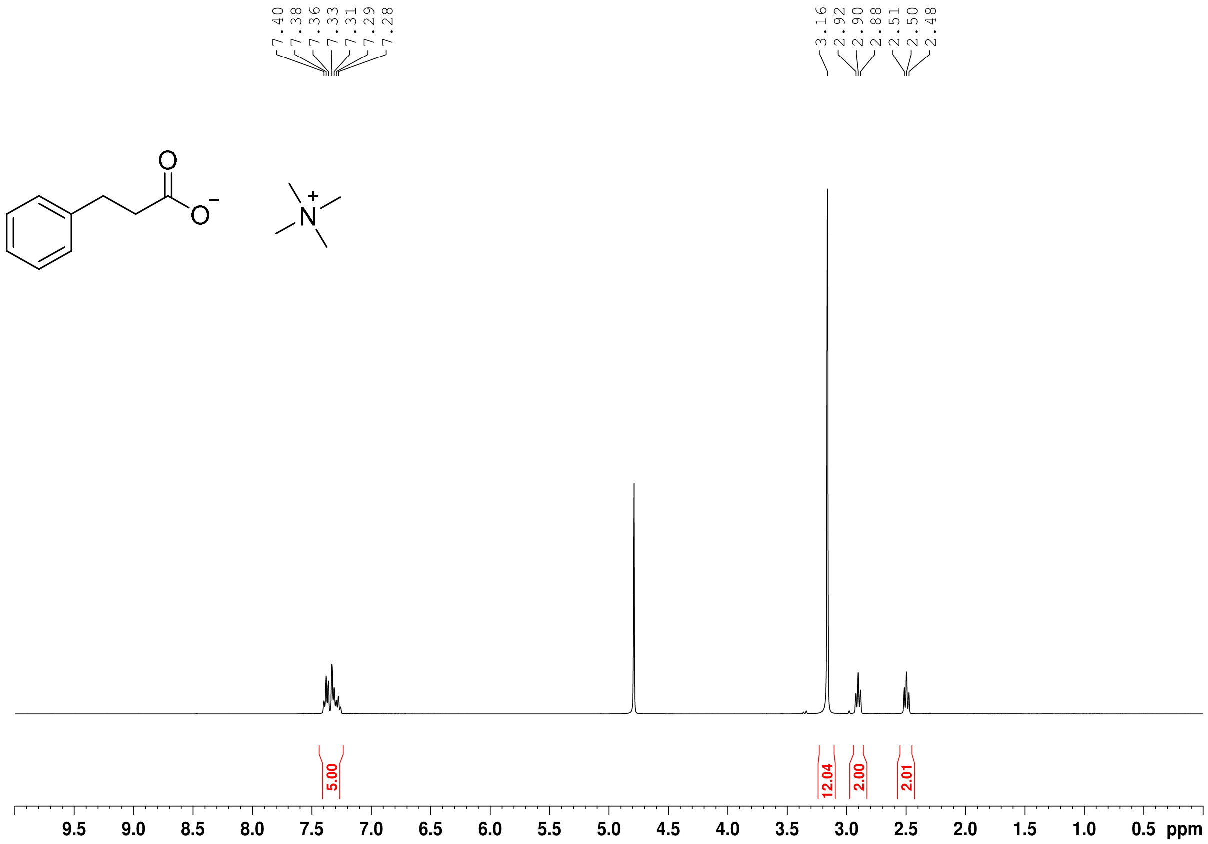

^{1}H NMR spectrum (400 MHz, DMSO-d_6) of **36**.

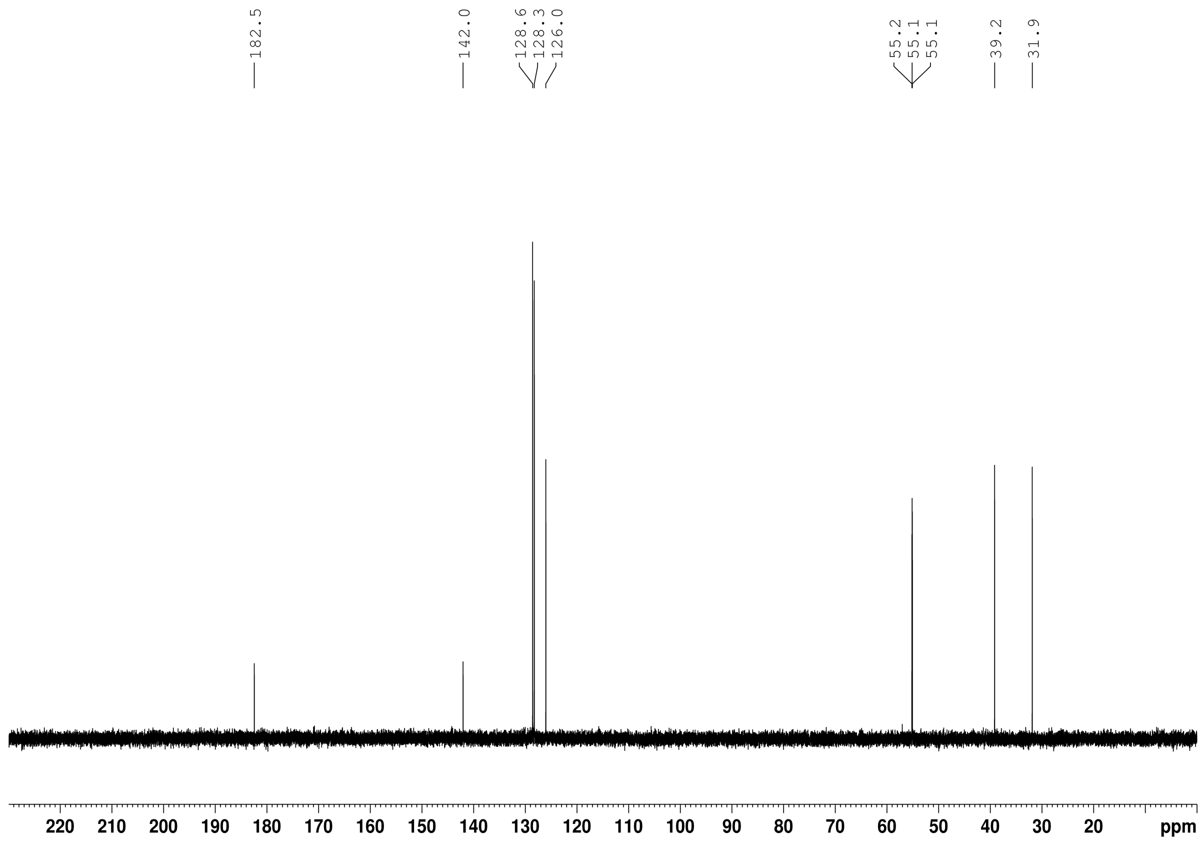

^{13}C NMR spectrum (100 MHz, DMSO-d_6) of **36**.

Tetrabutylammonium hydrocinnamate (37)

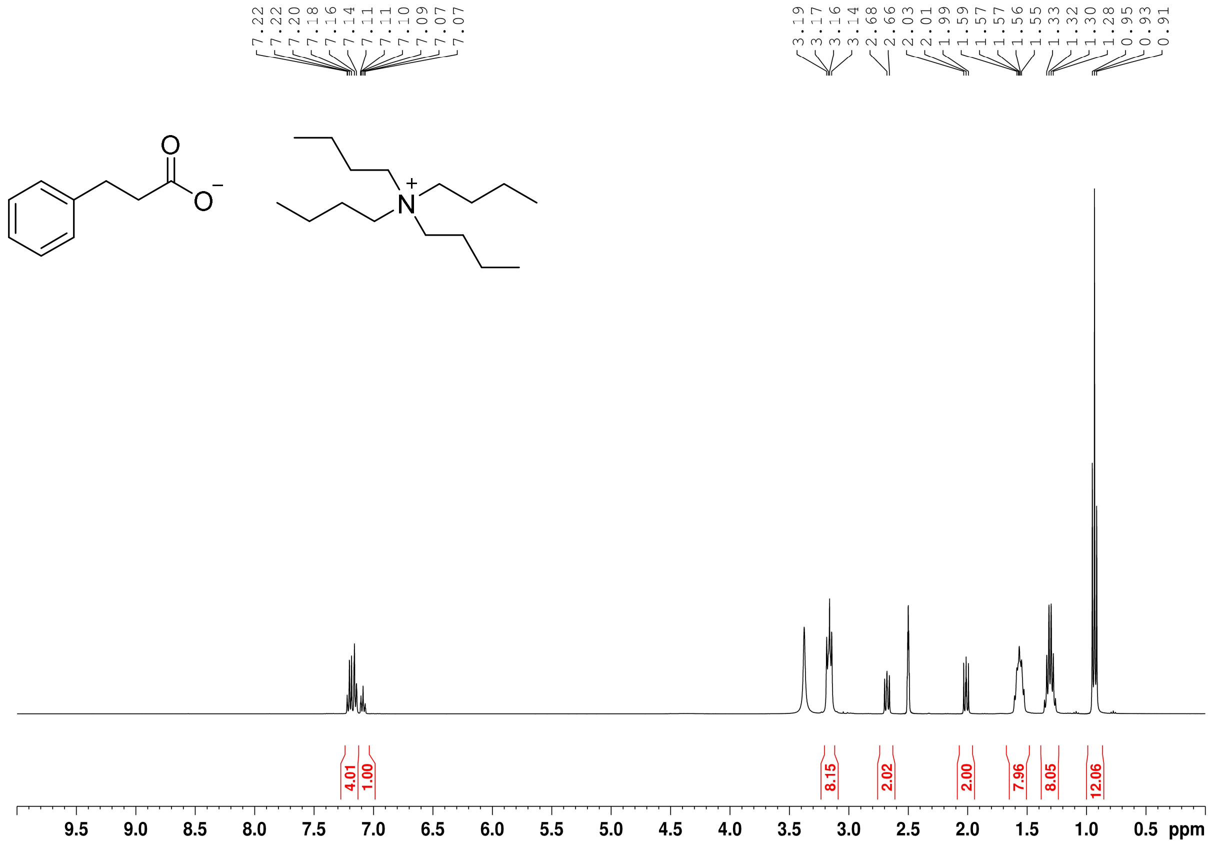

^{1}H NMR spectrum (400 MHz, DMSO-d_6) of **37**.

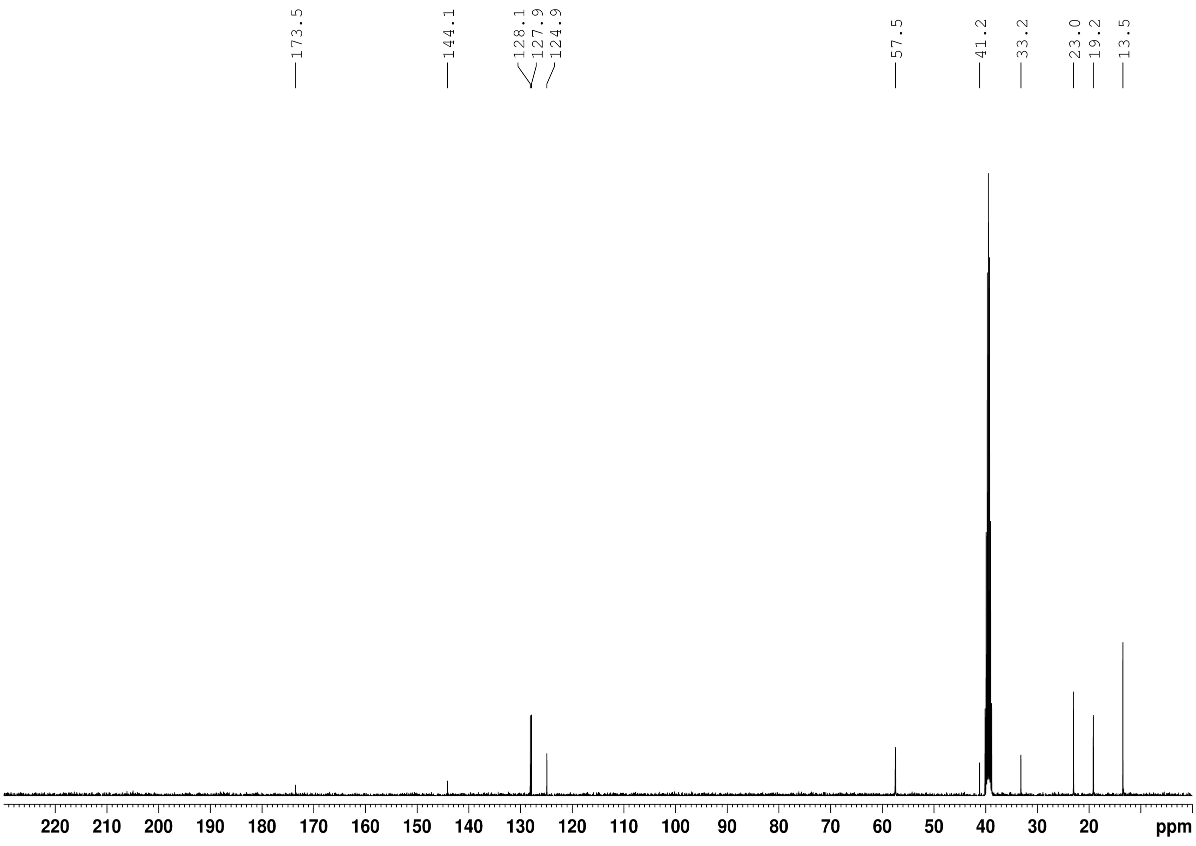

^{13}C NMR spectrum (100 MHz, DMSO-d_6) of **37**.

Tetrahexylammonium hydrocinnamate (38)

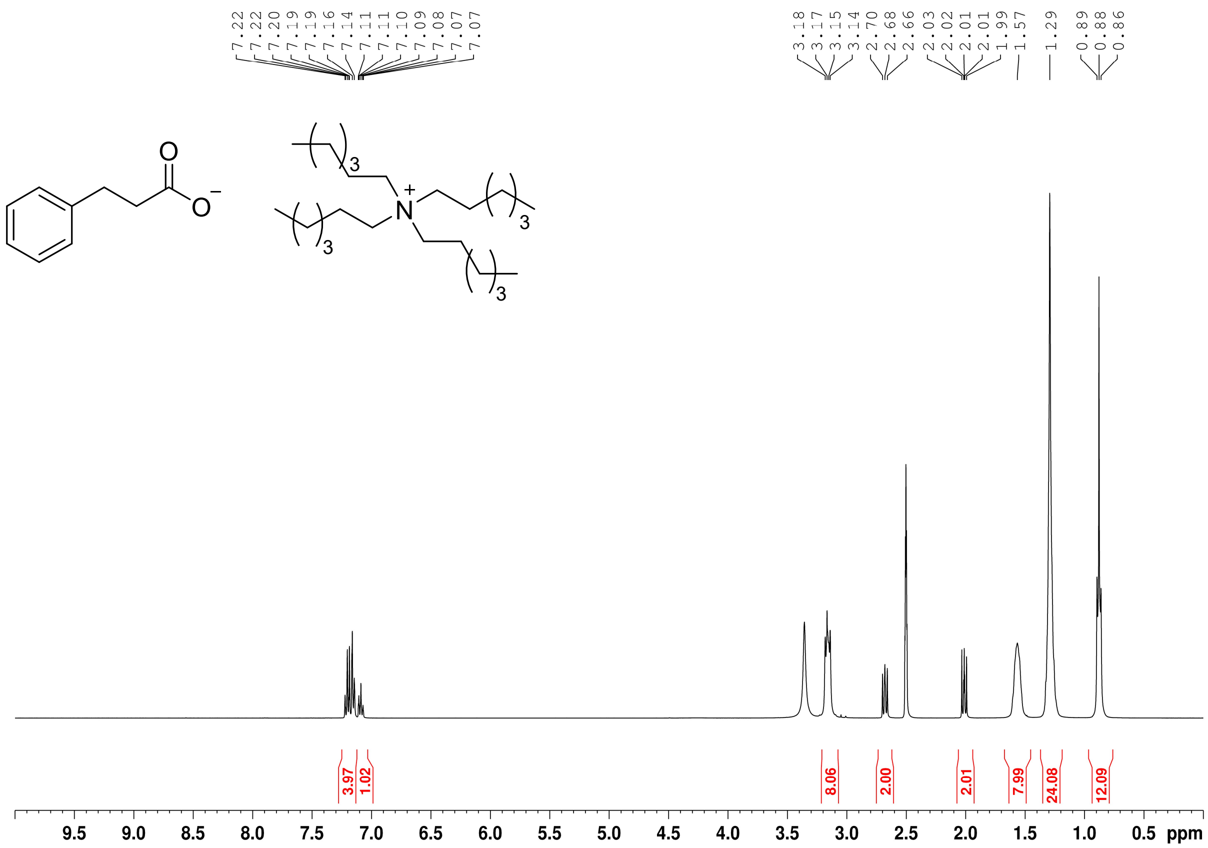

^{1}H NMR spectrum (400 MHz, DMSO-d_6) of **38**.

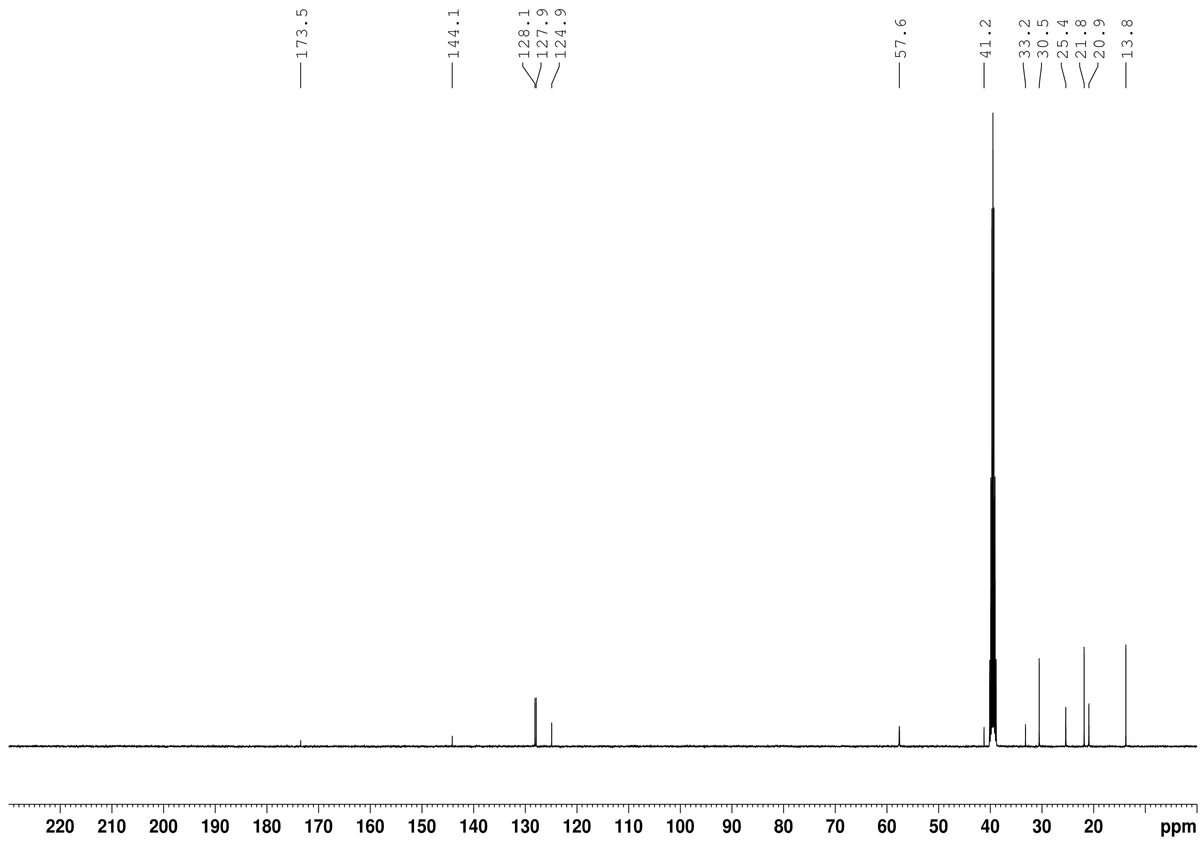

^{13}C NMR spectrum (100 MHz, DMSO-d_6) of **38**.

Tetraoctylammonium hydrocinnamate (39)

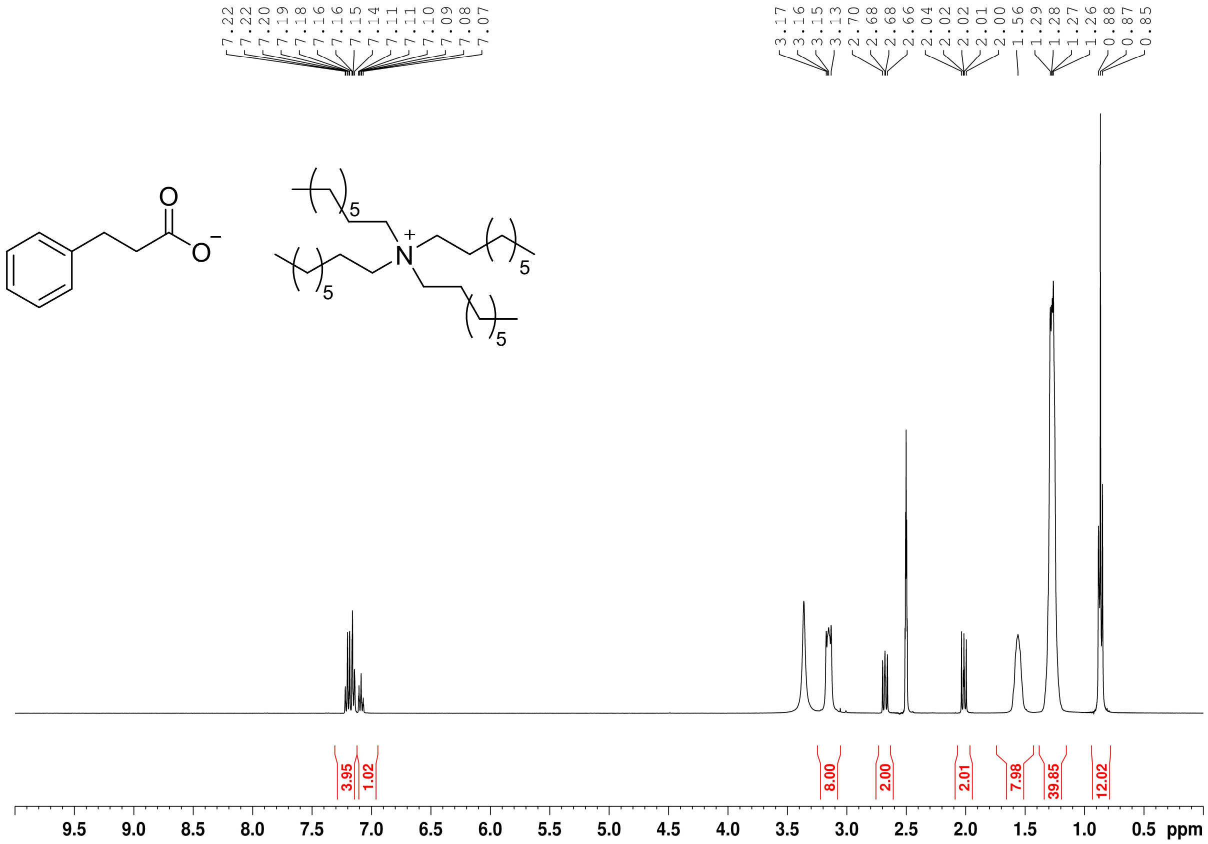

^{1}H NMR spectrum (400 MHz, DMSO-d_6) of **39**.

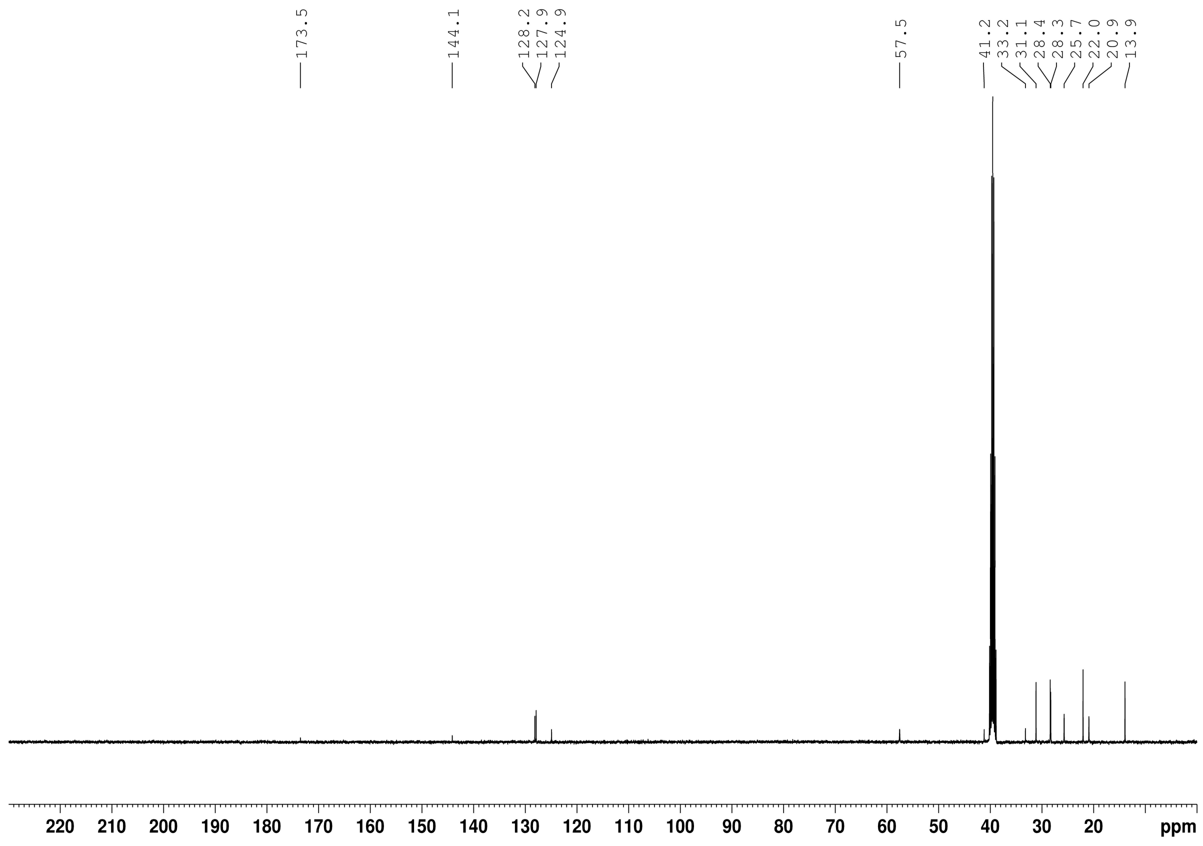

^{13}C NMR spectrum (100 MHz, DMSO-d_6) of **39**.

Methyltrioctylammonium hydrocinnamate (40)

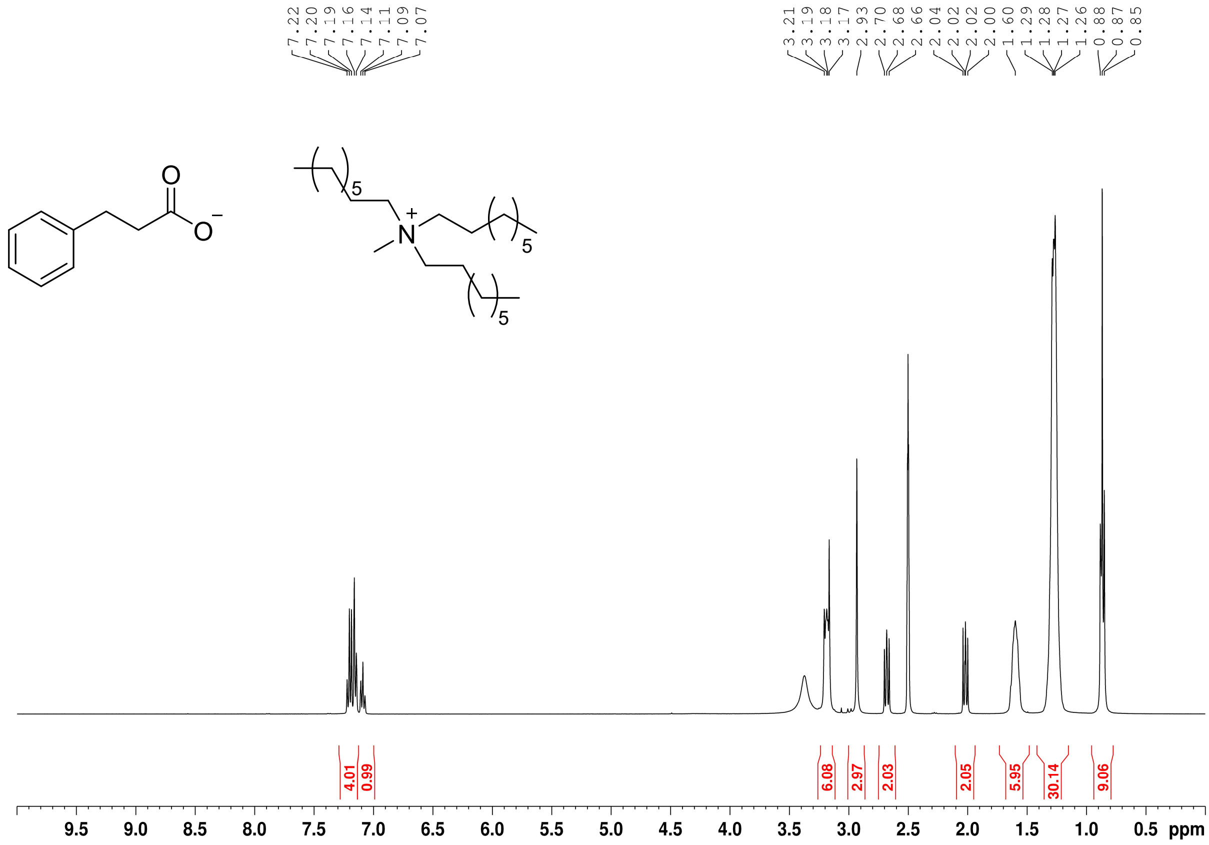

^{1}H NMR spectrum (400 MHz, DMSO-d_6) of **40**.

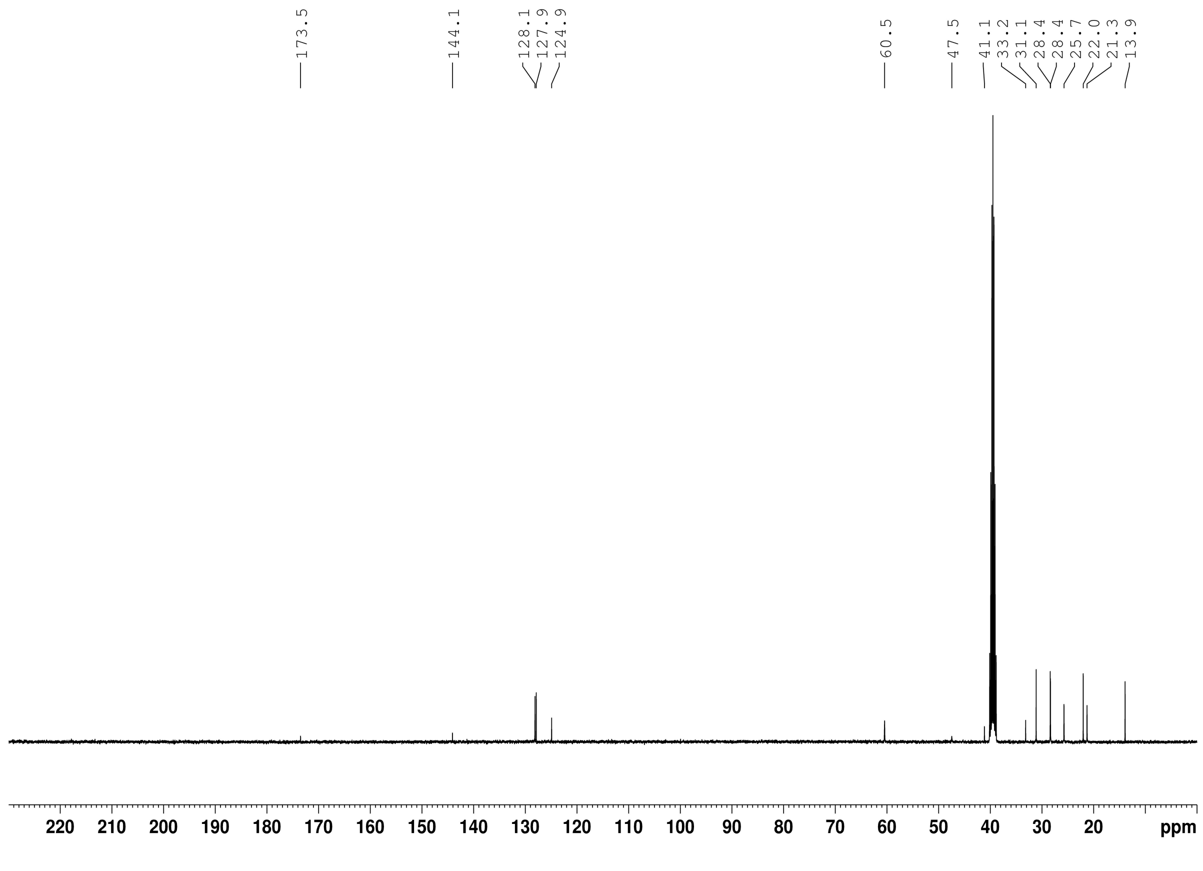

^{13}C NMR spectrum (100 MHz, DMSO-d_6) of **40**.

_N,N_-dimethyl-_N,N_-diundecylammonium bromide (S5)

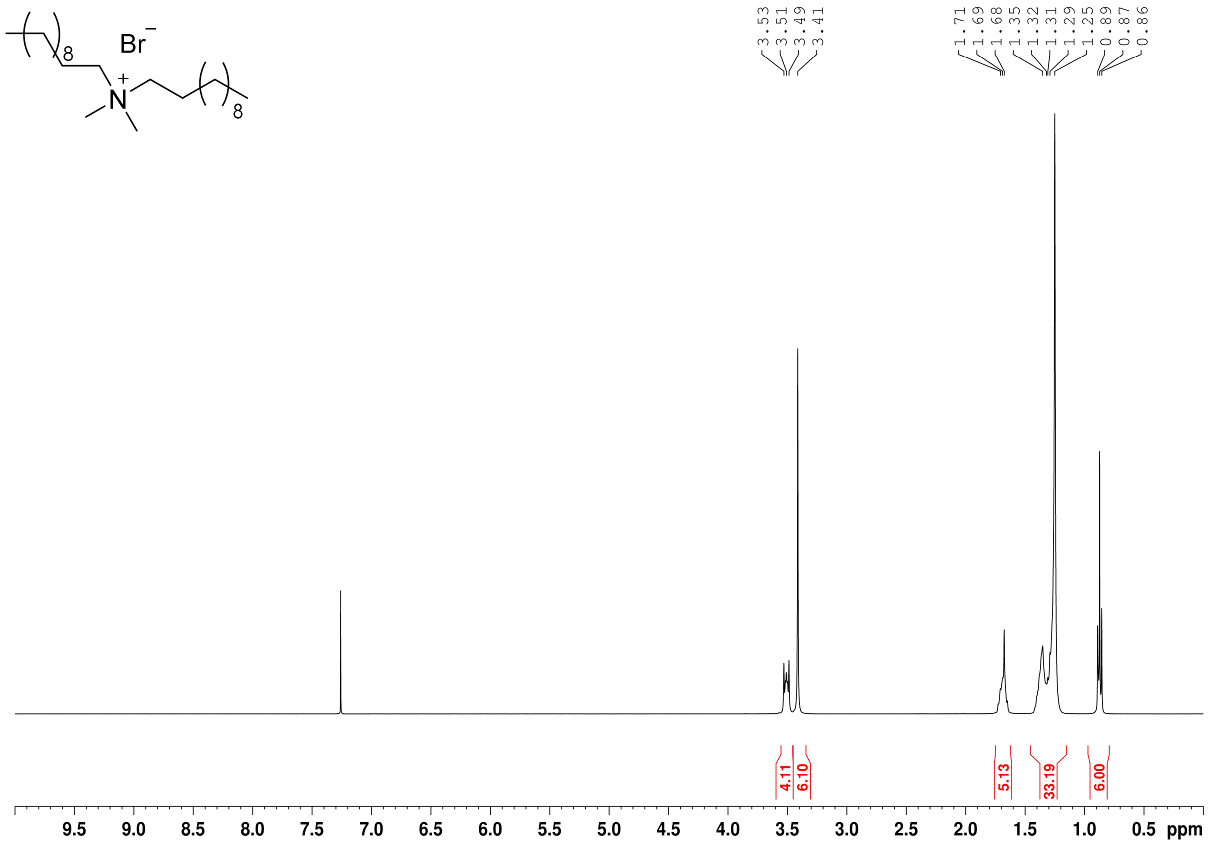

^{1}H NMR spectrum (400 MHz, $CDCl_3$) of **S5**.

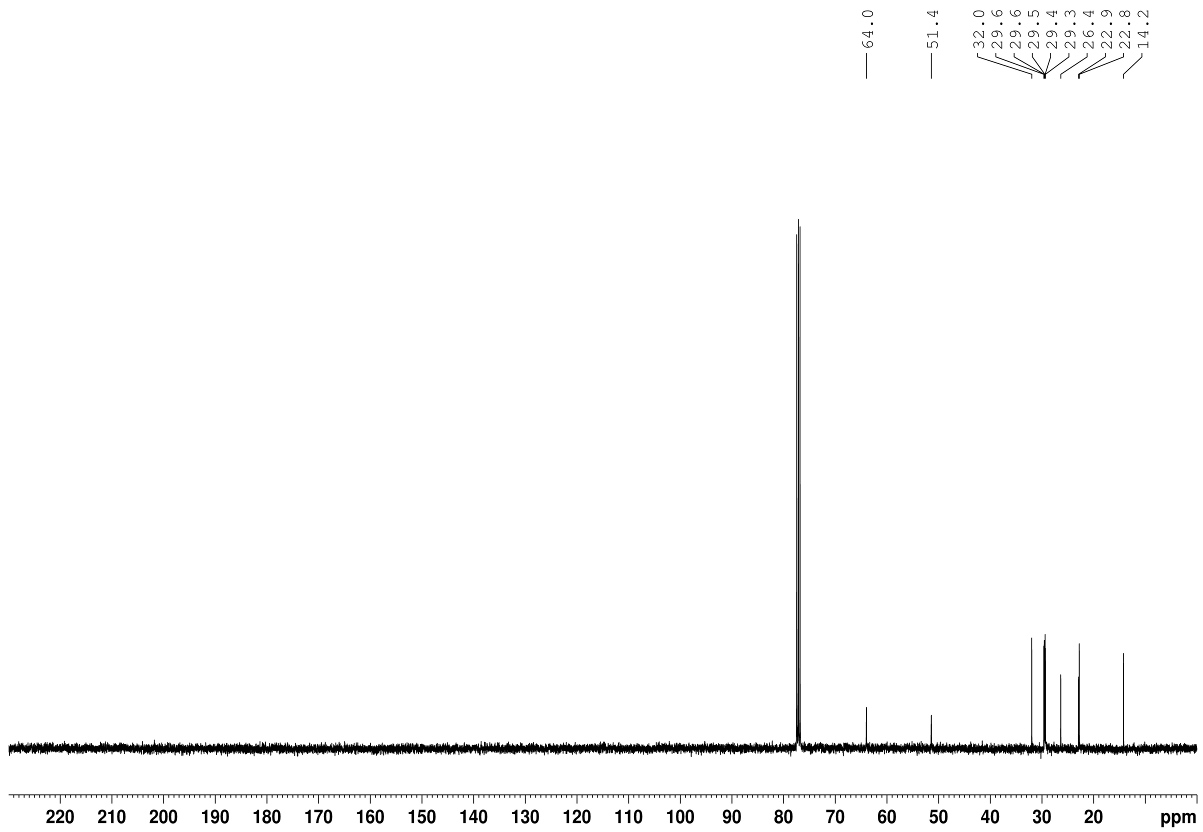

^{13}C NMR spectrum (100 MHz, $CDCl_3$) of **S5**.

_N_,_N_-dimethyl-_N_,_N_-diundecylammonium hydrocinnamate (41)

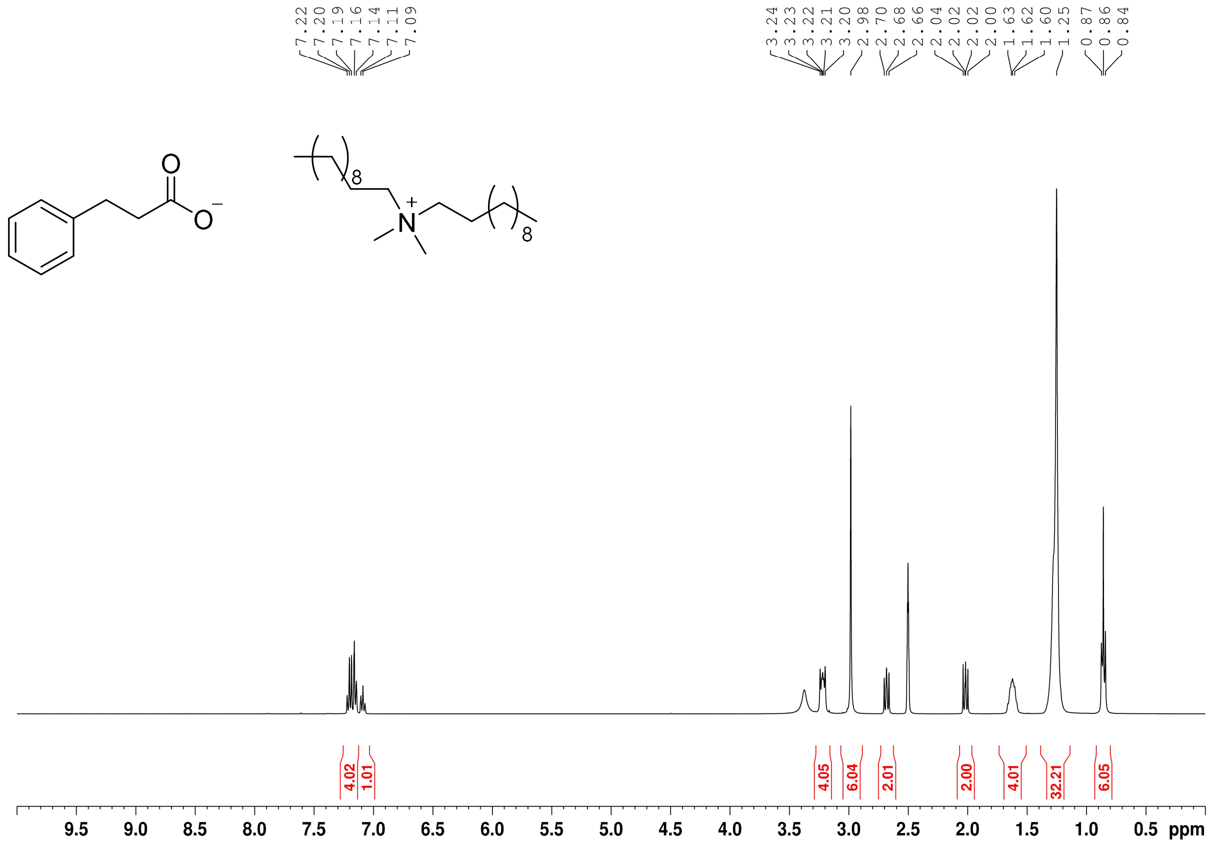

^{1}H NMR spectrum (400 MHz, DMSO-d_6) of **41**.

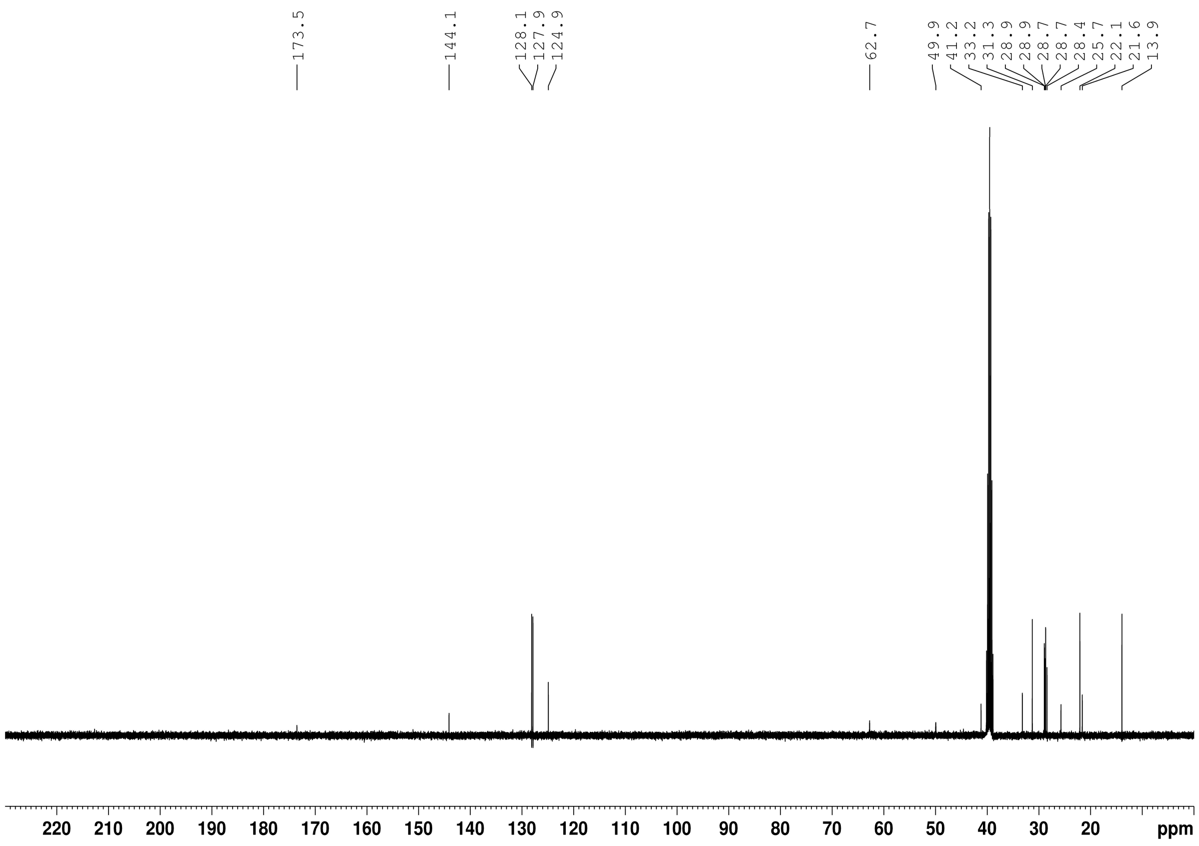

^{13}C NMR spectrum (100 MHz, DMSO-d_6) of **41**.

N-Methyl-*N*-octylpyrrolidinium bromide (S6)

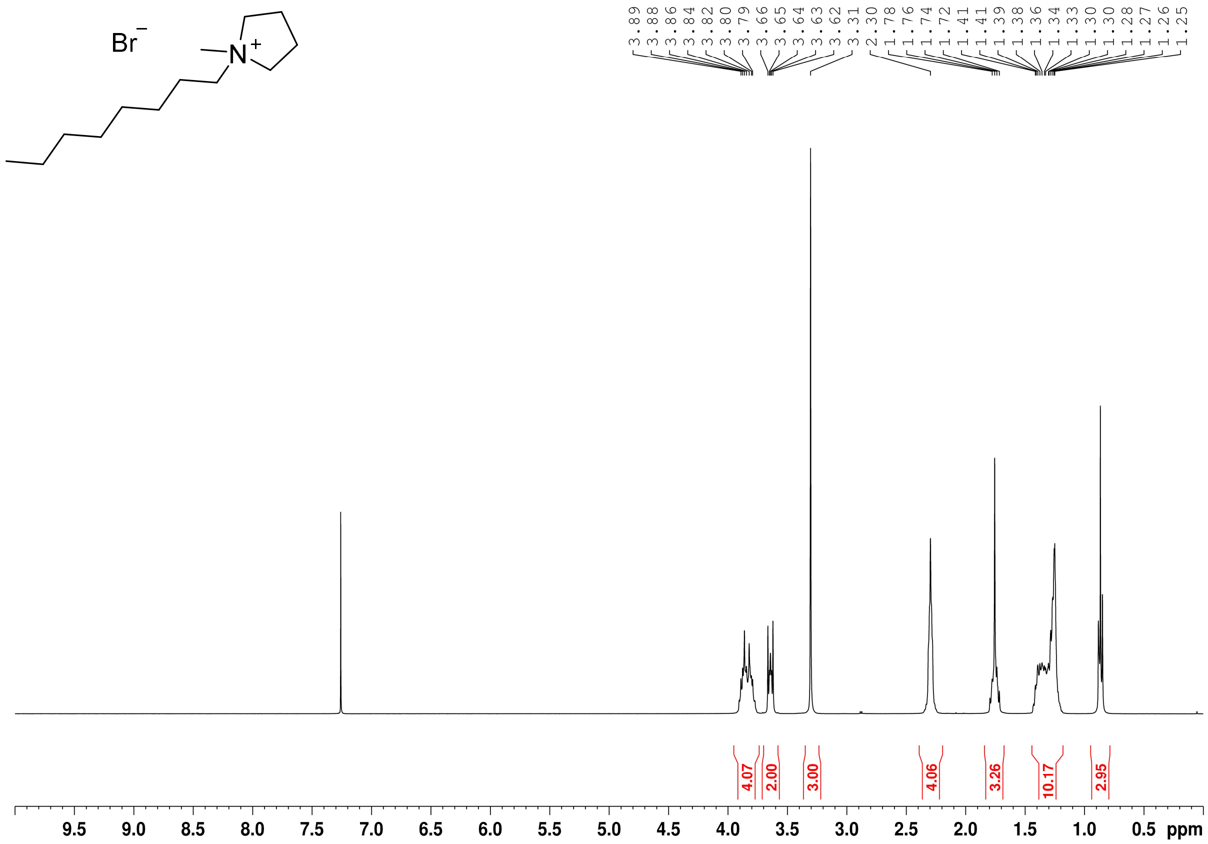

^{1}H NMR spectrum (400 MHz, $CDCl_3$) of **S6**.

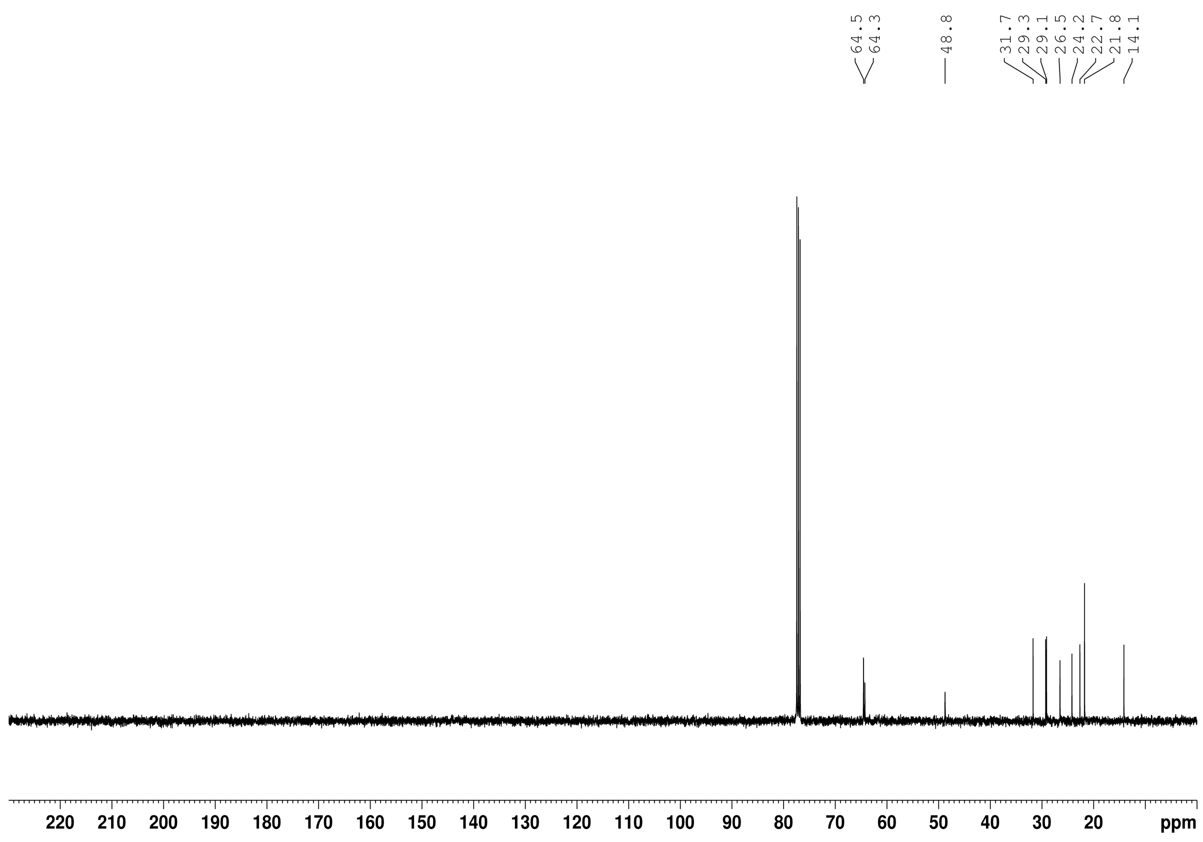

^{13}C NMR spectrum (100 MHz, $CDCl_3$) of **S6**.

1-Methyl-1-octylpyrrolidinium hydrocinnamate (42)

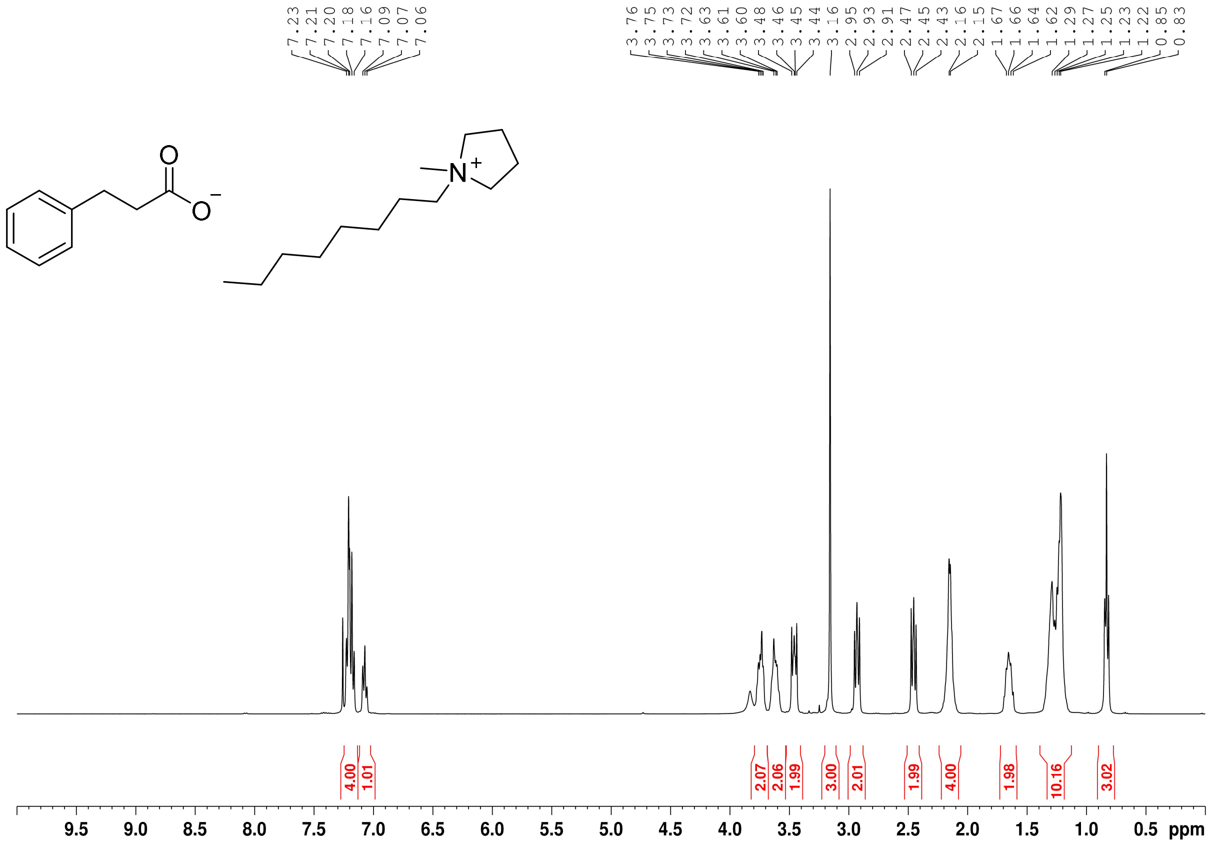

^{1}H NMR spectrum (400 MHz, DMSO-d_6) of **42**.

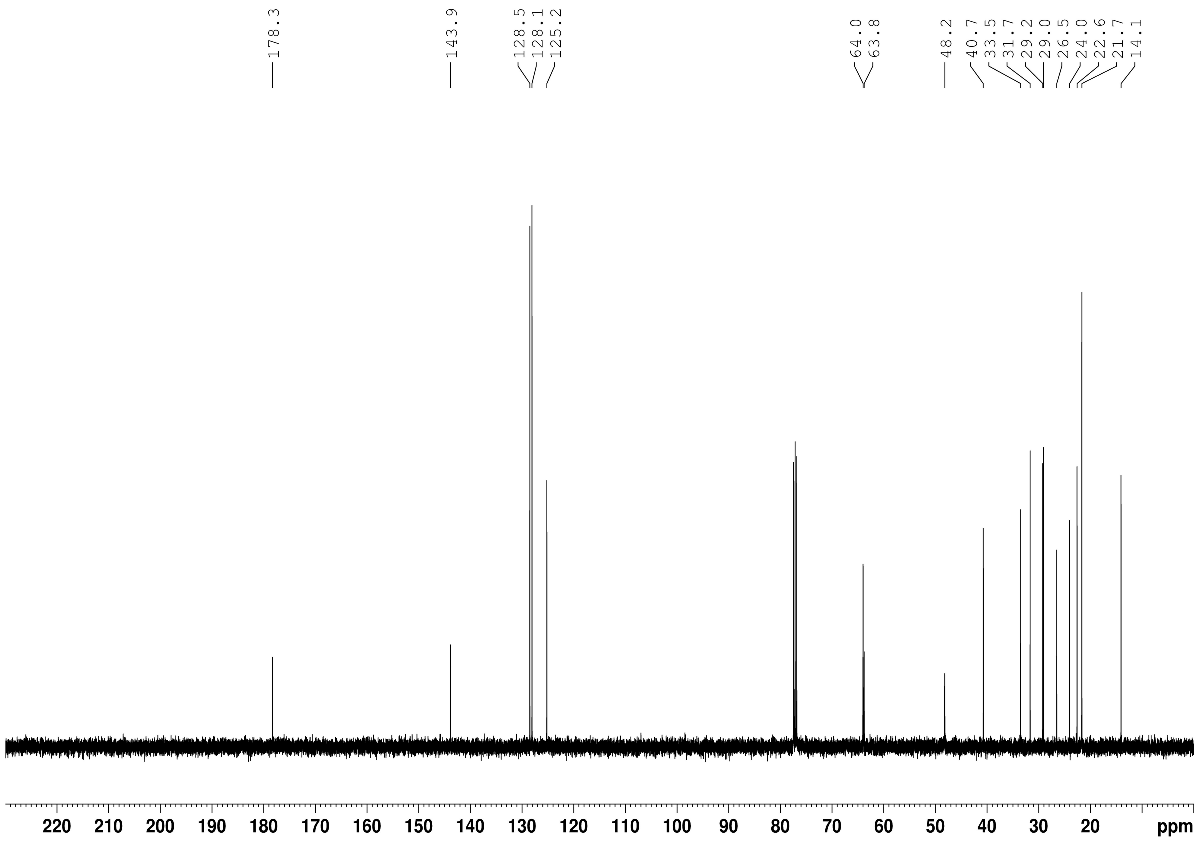

^{13}C NMR spectrum (100 MHz, DMSO-d_6) of **42**.

Methyloctylpyrrolidinium benzoate (43)

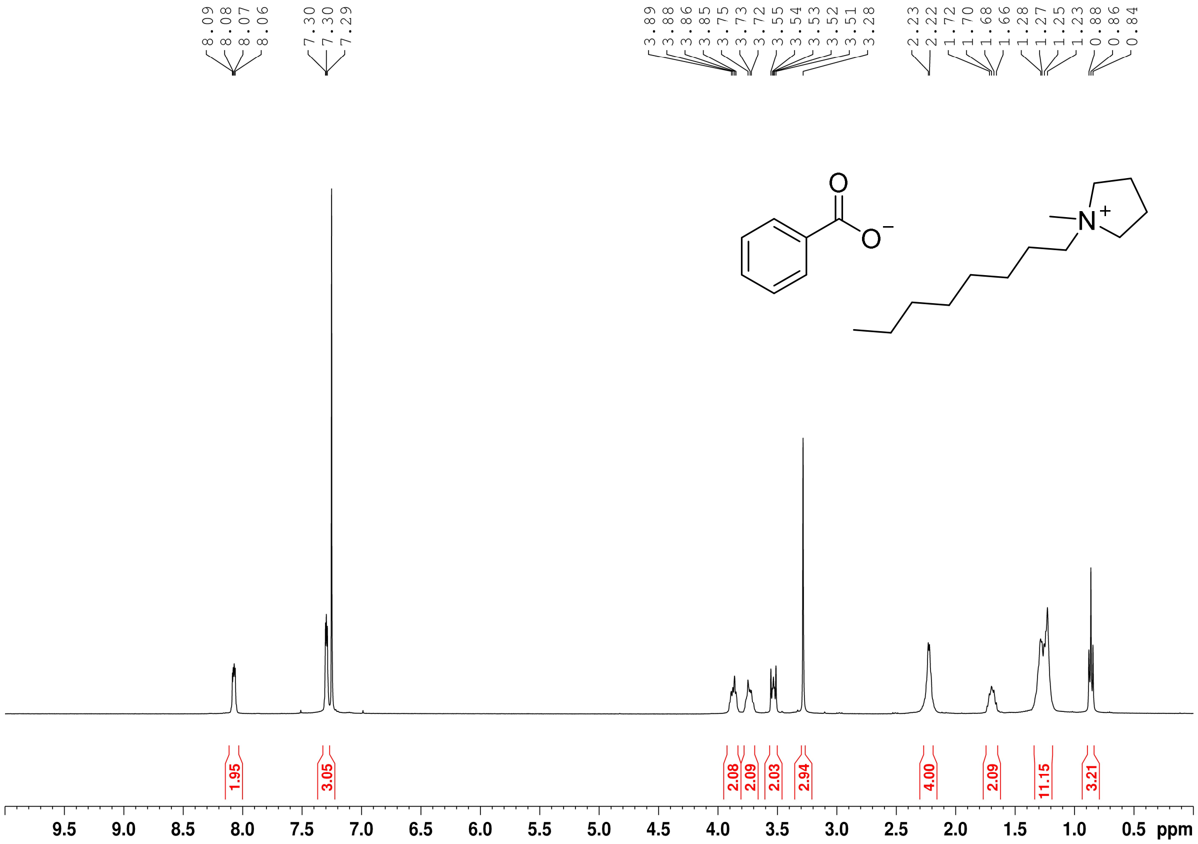

^{1}H NMR spectrum (400 MHz, $CDCl_3$) of **43**.

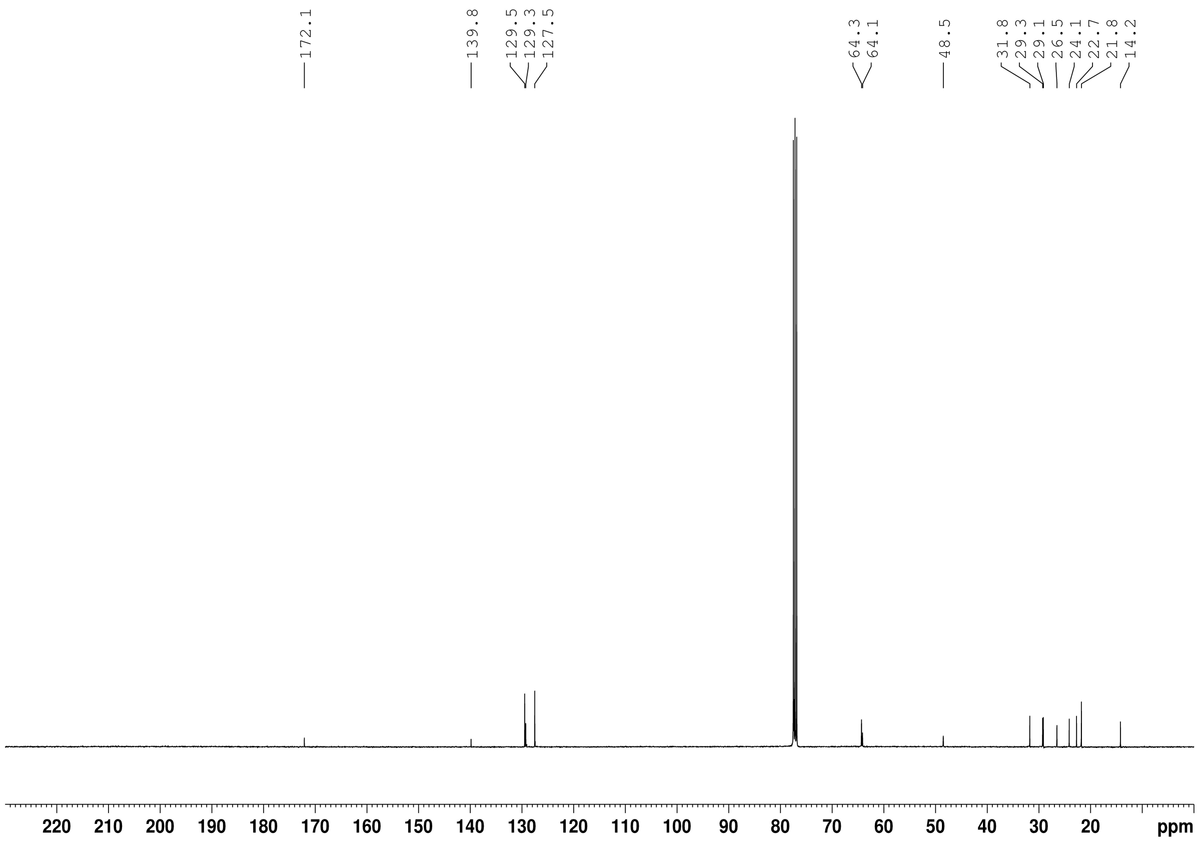

^{13}C NMR spectrum (100 MHz, $CDCl_3$) of **43**.

Dimethyldidecylammonium oleate (44)

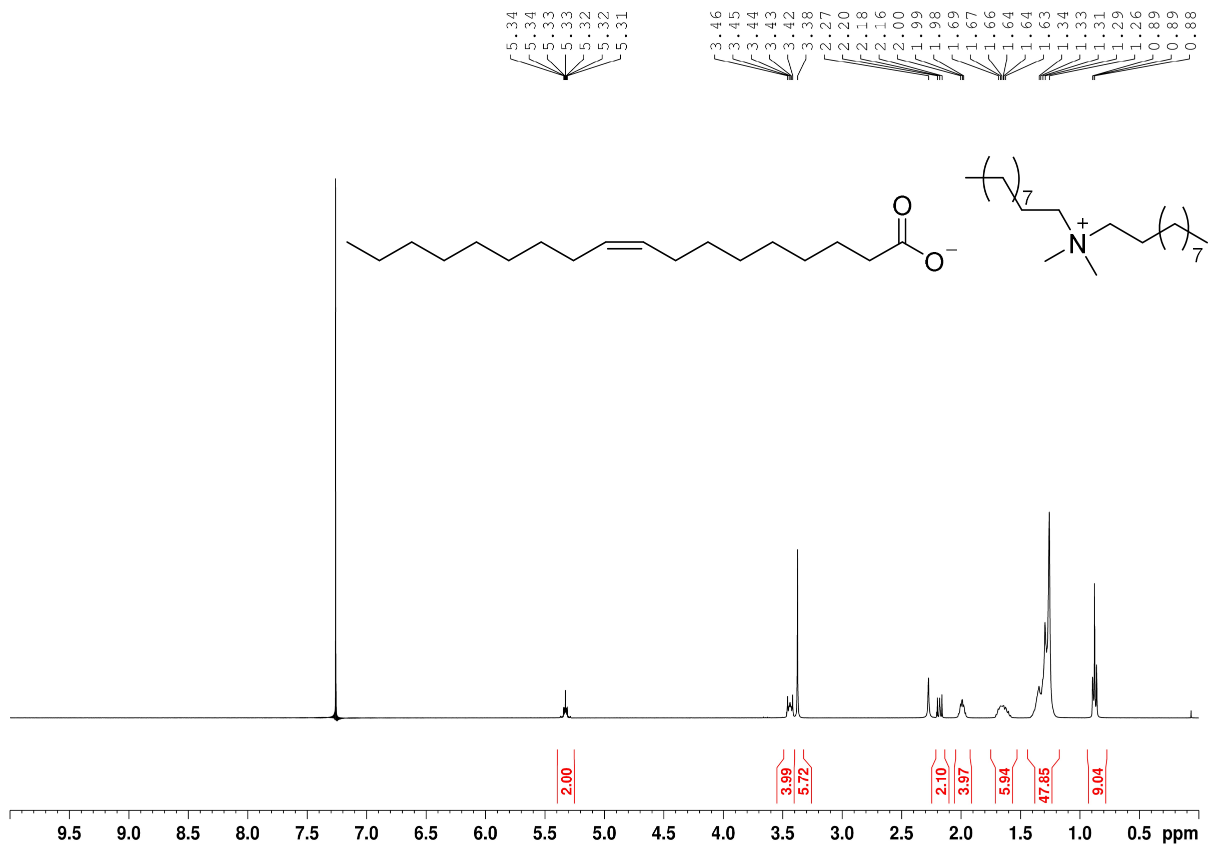

^{1}H NMR spectrum (400 MHz, $CDCl_3$) of **44**.

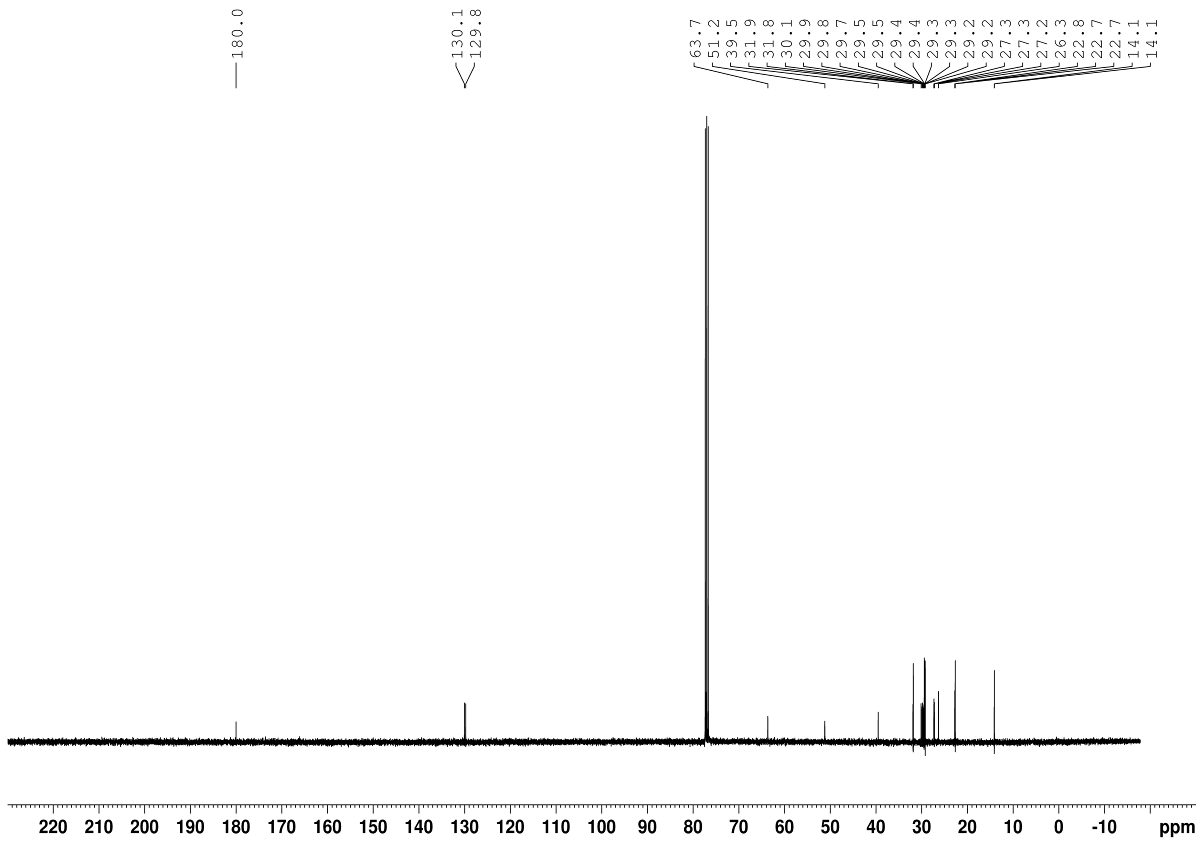

^{13}C NMR spectrum (100 MHz, $CDCl_3$) of **44**.
